# The Pastoral Origin of Semiotically Functional Tonal Organization of Music

**DOI:** 10.3389/fpsyg.2020.01358

**Published:** 2020-07-23

**Authors:** Aleksey Nikolsky

**Affiliations:** Independent Researcher, Austin, TX, United States

**Keywords:** tonal organization, animal communication, honest signal, semiosis, aspects of expression, domestication, kulning vs. yodel, motherese

## Abstract

This paper presents a new line of inquiry into when and how music as a semiotic system was born. Eleven principal expressive aspects of music each contains specific structural patterns whose configuration signifies a certain affective state. This distinguishes the tonal organization of music from the phonetic and prosodic organization of natural languages and animal communication. The question of music’s origin can therefore be answered by establishing the point in human history at which all eleven expressive aspects might have been abstracted from the instinct-driven primate calls and used to express human psycho-emotional states. Etic analysis of acoustic parameters is the prime means of cross-examination of the typical patterns of expression of the basic emotions in human music versus animal vocal communication. A new method of such analysis is proposed here. Formation of such expressive aspects as meter, tempo, melodic intervals, and articulation can be explained by the influence of bipedal locomotion, breathing cycle, and heartbeat, long before Homo sapiens. However, two aspects, rhythm and melodic contour, most crucial for music as we know it, lack proxies in the Paleolithic lifestyle. The available ethnographic and developmental data leads one to believe that rhythmic and directional patterns of melody became involved in conveying emotion-related information in the process of frequent switching from one call-type to another within the limited repertory of calls. Such calls are usually adopted for the ongoing caretaking of human youngsters and domestic animals. The efficacy of rhythm and pitch contour in affective communication must have been spontaneously discovered in new important cultural activities. The most likely scenario for music to have become fully semiotically functional and to have spread wide enough to avoid extinctions is the formation of cross-specific communication between humans and domesticated animals during the Neolithic demographic explosion and the subsequent cultural revolution. Changes in distance during such communication must have promoted the integration between different expressive aspects and generated the basic musical grammar. The model of such communication can be found in the surviving tradition of Scandinavian pastoral music - kulning. This article discusses the most likely ways in which such music evolved.

## Tonal Organization and Musical Mode

Since antiquity, scholars have been puzzled by the origins of music. Their quest still remains largely unanswered—impeded by the shortage of available data. The current consensus holds that some kind of musilanguage (Brown, 2000) must have preceded the bifurcation of music and language, marking the emergence of behavioral modernity in humans (Cross, 1999). Pitch orientation is seen as the primary structural marker of music, followed by rhythmo-metric organization (Brown, 2017)^1^. This unnecessarily oversimplified view can and should be expanded, since in reality music is organized not in two but in eleven *aspects of expression* (AEs^2^), each providing its autonomous information channel (Table 1):

**TABLE 1:** AEs of music.

**Acoustic domain**	**Aspects of expression in music**	**Physical substrate of an aspect**	**Perceptual substrate of an aspect**	**The overall range of expression of an aspect**	**Discrete increments within an aspect’s range of expression**	**Gradual inflections within the continuum of an aspect**
Frequency	1. Melodic pitch (consecutive “linear” i.e., “horizontal”)	Changes of the fundamental frequency (FF) between the consecutive tones within the same timbral register and voice/part.	Relation of the intervallic size of a frequency change to the Temporal Coherence Boundary^*I*^ and the segregation of audio streams that result from it^*LVI*^.	Minimal change—about 100 cents, maximal change—about 1,200–1,600 cents (leaps over an octave characterize a few specific genres^*XLVIII*^ (e.g., lamentation) or music systems (e.g., anhemitonic pentatonic).	The ambitus of a melody is divided into “degrees” based on the permanence in tuning (stability of pitch level) of tones of the same register that execute the same or similar melodic function within a phrase—the resulting intervals between the degrees define the “interval classes” in a musical mode^*II*^.	A degree can fluctuate in its frequency within a certain range of values that is usually equal or smaller than the interval between the adjacent degrees^*III*^—either in a form of a temporary alteration of that degree (syntactic inflection) or the portamento gliding between the adjacent tones (pragmatic inflection that traditionally constitutes the subject of intonation in Western music theory)^*XLVII*^.
	2. Harmonic pitch (concurrent “vertical”)	Consecutive changes in the relations of harmonics between the harmonic series of the concurrently sounding tones.	The number of harmonics that share the same frequency values in the harmonic series of each of the concurrently sounding tones^*IV*^.	Minimal matching of harmonics—when the distance between the FF of the concurrent tones is about or below 100 cents, maximal matching—when 1,200 cents apart.	The extent of matching and mismatching of harmonics varies between the degrees of a musical mode, forming a progressive “scale” of harmonic interval classes afforded by that mode, from the most “consonant” to the most “dissonant” interval^*V*^.	Each harmonic interval can slightly fluctuate in their exact tuning depending on the context of melodic and harmonic relations between the constituent tones within the same musical mode in a music piece, especially major and minor intervals—as a part of expressive tuning^*XXX*^.
	3. Musical texture	A specific type of arrangement (vertical and horizontal) of all musical sounds within a musical work	The number and relations of familiar conventional structural components in grouping of tones, themselves forming stereotypes of arrangement specific to certain genres^*XLIII*^; varying along 3 axes: density, range^*XLIV*^ and functions^*XLV*^.	The simplest case of texture is a monodic melody where grouping is restrained to shifts in melodic direction and leaps^*XLV*^ without rhythmic contrasts (e.g., Paganini—Moto perpetuo); the most complex is the great number of numerous parts/voices that are diverse in their function (e.g., Debussy—La Mer)^*XLVI*^.	Each texture breaks into a number of “stream segments” at its surface level of perception^*XLVII*^; forming discrete components—“textural cells” used as bricks in constructing a texture by vertical (chords) and horizontal grouping (motifs) of various complexity, functionality and hierarchic relations—ascribed specific semantic values^*XII*^.	N/A
	4. Musical form	Changes of the thematic material—a complex of musical structures consecutively ordered within a music work^*XXXIX*^.	Repetition, variation, contrast or recapitulation (i.e., the return of a thematic material after some other material) of a specific thematic material, identified by some salient feature(s)^*XXXVIII*^.	The simplest form is an exact reproduction of the same material (AA). The most complex is the “unveiling form” based on the ongoing contrast (ABCD…)^*XXXIX*^.	Changes in thematic material break a music work into discrete sections, with hierarchic relations between the changing phrases (A-B) and their changing constituent motifs (a-b), forming different hierarchic levels (e.g., A-B-A = ab-cd-ab).	Each section of a musical form can employ thematic materials of various salience, ranging from highly concentrated (symphony) to highly dispersed (prelude)^*XLI*^; the transition from the concentrated to the dispersed state can occur within the same section gradually^*XL*^.
Time	5. Rhythm	Relative duration of consecutive tones is quantized according to a certain division ratio (2,3…).	Grouping of the consecutive tones based on their perceived proportions of duration and position within a group^*VI*^.	Minimum—according to the Western classical music theory, semihemidemisemiquaver, or, 128th note (=6th division of a whole note), maximum—brevis (=2 whole notes)—relative to tempo; in absolute terms, from 20 msec^*VII*^ to 1,800 (2,000) ms^*XXXI*^.	Rhythmic proportions are estimated in terms of binary or ternary divisions that produce a set of standard durations—i.e., “time classes” (=rhythmic values) engaged in a composition—usually 3–5 divisions, one or two of which are most frequently used, forming a “metric grid” employed to round up the actual duration of a tone to a valid rhythmic value.	Rhythmic values can fluctuate in their actual duration in the so-called “expressive timing”^*VII*^ that exaggerates rhythmic contrasts by prolonging anchored tones while shortening tones in passages, ornaments, or short tones in those rhythmic figures that consist of contrasting durations—e.g., overdotting in the so-called “punctured” rhythm^*VIII*^; such flections overtake the normative ratios that govern the rhythmic divisions^*VII*^.
	6. Meter	Number of unstressed beats, grouped together with the stressed beat, before the occurrence of the next stressed beat—perceptually and statistically prevailing in a musical movement.	Grouping of beats based on the perceived periodicity of stresses generated by longer and louder tones as well as changes in melodic direction and harmony^*VI*^.	Minimal size group—1, i.e., “spondaic” pulse (every single beat is stressed), maximal size group—24 (compound ternary pulse, made by 4 divisions in 3-level hierarchy 3:6:12:24)^*XXXII*^; in absolute terms, periodicities from 100 to 6,000 ms^*IX*^.	Metric grouping is estimated in binary or ternary increments of an entire group that can be isochronous or non-isochronous^*IX*^, so that a metric pulse within a meter can proceed by symmetric or asymmetric increments (e.g., common time can switch between 1/1, 2/2, 4/4, 8/8 and 16/16 pulses, or 3/8 + 3/8 + 2/8, 3/8 + 2/8 + 3/8 etc.)^*L*^.	Metric stress can fluctuate within a metric group, making the metric increments inside it acquire or lose metric weight: this is achieved by placing the anacrusis on a different position within a metric group (e.g., in 4/4 pulse, placing the anacrusis on the fourth beat shrinks the group to three beats—such shift alters the metric pulse without replacing it by another pulse^*LV*^.
	7. Tempo	The average pace of beat within a span of musical movement, which retains a specific character of motion (e.g., hasty or lazy).	An overall impression of a characteristic movement of a certain type (e.g., walking, jogging, running, hopping) akin to a gait—estimated based on the interaction of the pace of beat and the rhythm which determines the choice of tempo by a performer^*IX*^ enabling an “absolute tempo” (i.e., the optimal pace for a given music piece)^*X*^ whose importance is reflected by the invention of the metronome^*XI*^.	Minimal metronome value—usually, 40 bpm, maximal metronome value—208 bpm, traditionally tied to heartbeat and gait rates that characterize a particular form of locomotion and its related affective state^*XXII*^, in absolute terms, within the range of the beat value from 300 to 900 ms^*VII*^– inferred based on the density of pitch changes per metric unit of time^*XXXIII*^.	Each tempo is defined by a specific velocity and a character of musical movement (e.g., *presto* rushes, while *allegro* does not), so that every musical culture works out a set of standard tempi—for classical music of the Common practice period it is a 12-tempi system^*XII*^ where a tempo, optimal for a given music piece, is defined as a range of bmp values within which the “feel” for that tempo remains the same^*XIII*^—narrow enough to consider a “perfect tempo.”^*XXXIV*^	Velocity of each tempo can be adjusted without changing its character—music practice often generates rules for temporary minor fluctuations, reflected by a set of modifier terms: e.g., for Western classical music these are *meno mosso*, *ritenuto*, *rallentando*, *piu mosso*, *stretto*, *accelerando*; tempo inflections can also be canceled (*tempo giusto* = strict time) or added (*tempo rubato* = constant slowing and speeding within the same phrase)^*XIII*^; the velocity curves for such inflections seem to be fixed by convention in reference to the cultural standards of locomotion^*XXXV*^.
	8. Articulation	The manner of attaching/detaching of successive tones within the same register and part/voice.	Shortening of the nominal rhythmic value (akin to the plucking sound production of mandolin or xylophone)—or, extending it (akin to echo) by the overlap of the end of one tone with the onset of the following tone (thereby generating a momentary harmonic interval in a monophonic line).	Minimal use of articulation—*non-legato*, maximal detaching—*staccatissimo*, maximal attaching—*legatissimo*^*XIV*^; these concepts do not seem to follow any absolute criteria.	Performance practice generates styles of consecutive rendition of textural elements (e.g., melody, chords, figurations) that fill a range from the most abbreviated to the most extended articulation in a gradient manner: *staccato*, *marcato*, *mezzo staccato*, *non-legato*, *portato*, *tenuto*, *legato*^*XIV*^—their contrast often generates groups (e.g., a 2-tone legato-tenuto or a 2-tone legato-staccato)^*XLII*^.	Many articulation styles form a continuous range of shortening or extending a rhythmic value, depending on the musical context—which establishes the performing conventions^*XV*^; a common case of contextual influence is adjusting the exact extent of legato^*XVI*^ and staccato^*XVII*^ depending on how high or low the register to which an articulated tone belongs is placed in the ambitus—the most common axes in flexing the articulation styles are connectedness, discreteness and compactness^*XLII*^.
Amplitude	9. Dynamics	Changes of amplitude between consecutive or/and concurrent tones within the musical texture.	Relative increase or decrease in intensity of a particular tone, textural element (e.g., melody, bass), component (chord), segment (accompaniment) or the entire musical texture.	Minimal dynamics—*pianissisimo* (*ppp*), maximal—*fortissisimo* (*fff*); these concepts do not seem to follow any absolute criteria, yet are present in many if not all music cultures, from Ancient Greece on^*XXXVII*^.	Music practice generates dynamic distinctions that generally correspond to the extent of affective intensity of the music^*XVIII*^ and form a “scale” of dynamics increments: *fortissimo*, *mezzo forte*, *forte*, *mezzo piano*, *piano*, *pianissimo*^*XIX*^—each featuring a range, narrower for experienced music users, highly variable in reproductions of the same music, but stabler per person^*XX*^.	Dynamics also uses gradual changes, usually to support climaxes and intensify contrasts^*XXXVII*^; such flections can be graded: positive—*piu forte*, *poco crescendo, molto crescendo*, *rinsforzando*; and negative—*meno forte*, *poco diminuendo* or *molto diminuendo*, *morendo*; although these terms appear in Western tradition only in the 19th century, similar notions seem to exist in implicit non-Western music theories to support flections of the expressive timing^*XXI*^ that is most crucial for phrasing^*XIV*^.
Timbre	10. Register	Contrasting changes in tonal quality between timbrally homogenous groups of tones within the ambitus of a music work.	Registral position in music is evaluated similarly to pitch—in terms of gradation in higher/lower placement within the ambitus that is employed in a music work—while accounting for timbral similarity in sound quality between adjacent pitches (e.g., registers that are darker/lighter or thicker/thinner in sound)^*XXII*^.	The lowest register forms one pole in the range of musical tones possible for vocal and instrumental production, while the highest register forms the opposite pole; for vocals and such instruments as flute or clarinet, the highest register is the strongest and most vibrant, whereas the lowest—the weakest and dullest; for brass, in contrary, the lowest register is the strongest and the most vibrant^*LI*^.	Human voices and musical instruments have break-up points in their tessitura, where the tonal quality noticeably changes, breaking in a few registers, each distinguished by its own coloration; thus, clarinet has four registers: somber “chalumeau” (E4-E5), dull “transition” (E5-B5), bright “clarino” (B5-C7), piercing “altissimo” (C7-A7)^*XXIII*^; 4 (3) registers are typical for most singing voices^*XXIV*^ and musical instruments^*XXV*^, but different cultures adopt different attitudes to registers: some smoothen registral contrasts, while others increase these contrasts^*XXVI*^.	Some musical traditions cultivate an overlap between the neighboring registers, extending the span of each register, thereby increasing its continuity—e.g., the countertenor can sing as baritone or bass^*XXVII*^; additionally, musical instruments usually develop an arsenal of performing devices to diversify their timbre providing “flections” of their “principal” timbre—e.g., *pizzicato*, *col legno*, *con sordino*, *sul ponticello* and *sul tasto* on string instruments^*XXVIII*^; similar devices are used by vocalists (parlando, aspirare, fioco); yet another common source of “flexing” the timbre and register is to stress a particular harmonic in the instrumental sound, thereby recoloring its timbre^*XXIX*^.
	11. Instrumentation	Selection of a type of musical instrument and vocals most suitable for a specific expression	Timbres of individual instruments and vocals can blend into a new timbre (e.g., oboe and clarinet), remain discrete yet complement each other (e.g., flute and oboe), or repel (e.g., harp and horn), depending on similarity and synchrony of spectral centroids and attacks^*XLIX*^; and salience of individual harmonics^*XXIX*^.	The simplest instrumentation is sustaining a single timbre per piece of music (solo); the most complex is the combination of orchestra and choir that features multiple foreground and background layers, changing over time, with contrasts between tutti, soli, and orchestral and/or choral groups^*LII*^.	Each type of musical instrument and vocals constitutes a specific tone color in a palette of a music-maker^*LI*^; certain combinations of instruments (string trio, wind quintet, orchestra)^*LIII*^ and vocals (duet, quartet, choir)^*LIV*^ form stable settings used to create music of certain semantic content depending on the tonal quality and technical capacities of the instruments^*LII*^.	N/A

*This table summarizes the structural characteristics of each of the principal AEs according to the treatises on music theory and relevant psychoacoustic research during the last 70 years. In the footnotes to the table, I have provided the relevant sources for those readers who are interested to find out more. All AEs feature incremental organization. Most AEs are quantifiable: melodic and harmonic intervals, textural elements and components, themes, metric pulses, rhythms, and articulation groups. Of 11 AEs, 9 (melody, harmony, form, rhythm, meter, articulation, dynamics and register) exhibit gradual inflections that are systemically used to increase the expressiveness of music. ^*I*^(Noorden, 1975, 40–67); ^*II*^(Nikolsky, 2015b); ^*III*^(Garbuzov, 1948); ^*IV*^(Benson, 2007); ^*V*^(McDermott et al., 2010); ^*VI*^(Jones, 2016); ^*VII*^(Clarke, 2007); ^*VIII*^(Fabian and Schubert, 2008); ^*IX*^(London, 2004); ^*X*^(Levitin, 1994); ^*XI*^(Fallows, 2001); ^*XII*^(Nazaikinsky, 1972); ^*XIII*^(Garbuzov, 1950); ^*XIV*^(Keller, 1973); ^*XV*^(Jerkert, 2003); ^*XVI*^(Repp, 1995); ^*XVII*^(Repp, 1998); ^*XVIII*^(Dean et al., 2011); ^*XIX*^(Berndt and Hähnel, 2010); ^*XX*^(Garbuzov, 1955); ^*XXI*^(Todd, 1992); ^*XXII*^(Drabkin, 2001); ^*XXIII*^(Miller, 2014); ^*XXIV*^(Titze, 1988); ^*XXV*^(Patterson et al., 2010); ^*XXVI*^(Yemelyanov, 2000, 46); ^*XXVII*^(Ravens, 2014); ^*XXVIII*^(Garbuzov, 1956); ^*XXIX*^(Nazaikinsky and Rags, 1964); ^*XXX*^(Rags, 1980); ^*XXXI*^(Fraisse, 1982); ^*XXXII*^(Harding, 1983); ^*XXXIII*^(Madison and Paulin, 2010); ^*XXXIV*^(Gabrielsson, 1999); ^*XXXV*^(Friberg and Sundberg, 1999); ^*XXXVI*^(Chew, 2001); ^*XXXVII*^(Thiemel, 2001); ^*XXXVIII*^(Réti, 1951); ^*XXXIX*^(Mazel, 1979); ^*XL*^(Kholopov, 2006); ^*XLI*^(Val’kova, 1992); ^*XLII*^(Braudo, 1961); ^*XLIII*^(Huron, 1989); ^*XLIV*^(Benward and Saker, 2009); ^*XLV*^(Skrebkova-Filatova, 1985); ^*XLVI*^(Berry, 1987); ^*XLVII*^(Cambouropoulos, 2010); ^*XLVIII*^(Rags, 1999); ^*XLIX*^(Sandell, 1995); ^*L*^(Kholopova, 2002); ^*LI*^(Meyer, 2009); ^*LII*^(Banshchikov, 1997); ^*LIII*^(Kendall and Carterette, 1993); ^*LIV*^(Kreitner et al., 2001); ^*LV*^(Rothstein, 1989); ^*LVI*^(Cambouropoulos, 2008).*

• Melodic contour,
• Harmony,
• Texture,
• Form/thematicity,
• Tempo,
• Rhythm,
• Meter,
• Articulation,
• Dynamics,
• Register,
• Timbral quality (instrumentation)^3^.

The problem is that in investigation of music, cognitive scientists rely on “standards” of Western musical theory, produced by Western civilization and therefore specific to certain historic periods and geographic regions. Although Western music system has proved to be the widest spread and the oldest surviving tradition, with its theoretic foundation rooted in the 3rd millennium BC (Dumbrill, 1998; Mathiesen, 1999; Jorgensen, 2003; Christensen, 2008; Crickmore, 2009; Nikolsky, 2016), nevertheless, there are other civilizations that abide by their own musical theories, explicit or/and implicit, documented or/and orally transmitted (Nettl, 2005). The need to formulate a “meta-theory” applicable to all varieties of musics has been realized only in the 1890s and dealt with by the discipline of systematic musicology (Bader, 2018). However, this discipline too inherited the framework of Western “classical music,” which is just one of many (Nikolsky, 2015b, 2016, 2020; Nikolsky et al., 2020). Since this framework is tailored to incremental frequency changes, the pitch-related AEs have been prioritized in Western musicology, covered by the dedicated disciplines of harmony, counterpoint, and musical form (Christensen, 2008). The other AEs have only recently received attention, after the traditional discipline of musical form was approached semiotically (Bobrovsky, 1978; Mazel, 1979; Ratner, 1980; Nazaikinsky, 1982, 1988, 2013; Lerdahl and Jackendoff, 1985; Berry, 1987; Ruwet and Everist, 1987; Beliayev, 1990b; Molino, 1990; Nattiez, 1990; Aranovsky, 1991, 1998; Monelle, 1992, 2000, 2006; Narmour, 1992; Tarasti, 1994, 1995, 2012; Kholopova, 2002; Arom, 2004; Bonfeld, 2006; Medushevsky, 2010; Tagg, 2012; Turino, 2014; Benjamin et al., 2015; Yust, 2018). Cross-examination of syntactic, pragmatic, and semantic use of conventional musical idioms has revealed that they break into 11 different AEs (Table 1). Nine of them are used in monophonic music (without harmony and texture)^4^. Each AE is distinguished by its unique perceptual substrate and idiomatic expressions.

Interspecific comparison of human music to vocalizations of different animal species along these aspects promises a better understanding of the qualitative leap in the emergence of music. The Moscow school of “integrative analysis”^5^ presents a methodology for such interspecific analyses, which I have adapted to identify those typological patterns in AEs of human music that contrast *animal calls* (ACs). These contrasts should be examined to reveal what exactly in human cultural evolution could be responsible for the emergence of new AE patterns that are unique to humans.

Human music is distinguished by its incremental structure (Bresin and Friberg, 2011)—requiring the ability to discriminate changes in at least 9 AEs (Table 1). Their categorization into “classes” seems to be modeled after pitch. A music-maker breaks the range between the lowest and the highest pitch classes (i.e., ambitus) within a music work into “degrees,” forming a set of pitch classes to construct music. Similarly, other AEs divide the continuum between their marginal values into step-like increments, the assortment of which can structurally characterize a musical work. Pitch-class sets receive their analogs in sets of the following classes, intuitively selected by a music-maker for a particular expression per composition:

• “time-classes” (number of rhythmic values i.e., “divisions”),
• “pulse-classes” (number of periodicities in a metric grid),
• “tempo-classes” (number of musical movements)^6^,
• “articulation-classes” (number of styles of connecting consecutive tones),
• “dynamics-classes” (number of dynamic gradations),
• “register-classes” (number of zones of different tonal coloration),
• “texture-classes” (number of textural components),
• “form-classes” (number of themes).

Such discrete classes coexist with gradual inflections for each class (Table 1). Evidently, music is designed to integrate multiple AEs in a complex admixture of their patterns of expression. Music defaults to the *integration* of concurrent tones in contrast to the *segmentation* tendency of speech (Bregman, 1994)—people can sing together, yet when speaking, they always take turns (Brown, 2007). Here, AC sides with music rather than speech, evident in the widespread animal chorusing. Integrative power of music makes the concept of “musical mode” indispensable for understanding the rise of music. “Mode’s” reduction to “scale,” adopted by some researchers (i.e., Pfordresher and Brown, 2017) constitutes a fundamental error in confusing the purely quantitative and formalistic concept of “scale” with the qualitative and content-oriented concept of “mode” (see Nikolsky, 2015b). Musical mode is more than a mere set of pitch-classes selected to make music—it also encapsulates the rules for their interconnection and the semantic range of suitable expressions (Wulstan, 1971; Alekseyev, 1976; Kholopov, 1976, 2005; Bytchkov, 1987, 1997; Lester, 1989; Beliayev, 1990a; Porter et al., 2001; Powers and Wiering, 2001; Straehley and Loebach, 2014; Winnington-Ingram, 2015).

In essence, “mode” constitutes the generalization of a particular melodic typology, characteristic for a given musical genre, which supplies that mode with semantic denotations (Nazaikinsky, 2013). Nothing similar exists in speech. Music is unique in its holistic appreciation of sounds *per se* (Patel, 2010). Hence, the idea of *euphony*—pleasant concordance of sounds in specific expressions—is quintessential for “mode,” as emphasized by Russian theorists.

The same principles apply to “rhythmic modes,” conceptualized within Western (Roesner, 2001) and some non-Western civilizations (Clayton, 2000). Rhythmic divisions, utilized in a composition, complement one another in expression of musical movement and in combinatory rules. A rhythmic modus in Western medieval theory, Arabic maqam, Iranian dastgah, or Indian raga incorporates not only a specific progression of rhythmic values but a specific “ethos”— an abstracted emotional quality projected by music on society at large (Shestakov, 1975). Each rhythmic modus in the abovementioned music systems is characterized *semantically* by its affiliation with a certain ethos and *structurally* by certain proportions between the duration values used in a music work. Rhythmic modus resembles pitch modus by incorporating a set of rules. Just as pitch-classes are allowed to follow or not follow one another, or require an alteration for ascending or descending motion, rhythm-classes are restricted to certain ratios which can be altered in a certain way (e.g., a dotted rhythm can be “over-dotted” in a suitable context).

The idea of concordance and appreciation that underlies the overwhelming majority of known traditional music cultures justifies the conceptualization of each AE as a carrier of its proprietary “mode.” Every musical piece can be defined by identifying its melodic, harmonic, rhythmic, metric, tempo, articulation, textural, and timbral modes.

Together, these modes constitute “tonal organization” (TO) in music. Conceptualized by François-Joseph Fétis (1840), TO is a method of joining musical tones together according to the sensibility of music-users (Fétis, 1994, XXV). Unlike tonemes of tonal languages, musical TO affects *all* tones, generates *complex* functional relations between them, and involves rhythmo-metric, dynamic, articulatory, and registral arrangements. Speech might also use similar arrangements (Patel, 2006). But music requires a special analytic attention where changes in the melodic contour are quantized into pitch-classes that are continuously cross-compared—unlike the linguistic “vowel pitch” (Walker, 1997, 322–3). Such syntactic pitch-parsing is as imperative for music as word-parsing is for language. Semantics provides yet another distinction: verbal syntax specializes in conveying referential meaning, whereas music specializes in emotional expression^7^ (Gabrielsson and Lindström, 2001; Juslin, 2001, 2005, 2011, 2013; Cook, 2002; Krumhansl, 2002; Gabrielsson and Juslin, 2003; Dissanayake, 2008; Johnson-Laird and Oatley, 2010; Trainor, 2010; Perlovsky, 2012; Altenmüller et al., 2013b; Eerola and Vuoskoski, 2013; Eerola et al., 2013; Peretz, 2013; Nikolsky, 2015a, 2020; Schiavio et al., 2016). Such distinction has been fundamental for the musical practices and theories of most musical traditions before Western classical music was swept away by the 20th century modernistic “revolution.” This distinction became revived after emotion and music attracted intense neuro-psychological research in the 1980s.

Music’s social nature—evident in entrainment^8^ (Tarr et al., 2014)—and emotionality—evident in chills (Altenmüller et al., 2013a)—are critical for distinguishing music: neither entrainment nor chills characterize verbal communication. And both are closely related through emotional contagion (Trost et al., 2017). This music/language distinction must have been already present in musilanguage, since in AC referential and motivational information is coded differently (Manser, 2010). However, music differs from ACs by encoding affective information according to the conventional modes of numerous AEs, as we shall see. Hence, the structural definition of music should be:

TO of multiple AEs that *entrains* listeners and performers and *transposes* performers’ intentions to emotionally stir listeners through vocal and/or instrumental performance.

Pitch contour, rhythm/meter, and dynamics (the most salient AEs) together constitute the principal structural criteria of music.

## Emic and Etic Approaches to Tonal Organization

The proposed definition is instrumental for engaging an additional source of evidence in the quest for the origins of music—the comparative structural analysis of world’s archaic indigenous musics, earliest forms of music-making by human infants, and animal vocalizations. The modern advances in computer science support the acoustic and statistical analyses of vast datasets unavailable before. Such investigation could radically update the evolutionary theory while resolving the current situation in comparative ethnomusicology that is nothing short of a crisis (Savage and Brown, 2013).

Many cognitive scientists remain unaware of the profound ideological shift in Western ethnomusicology that occurred during the last half-century. In essence, the study of “text” became replaced by the study of “people” (Zemtsovsky, 1997)^9^. The turning point was marked by Gourlay (1982) at the 1979 Oslo Conference of the IFMC by a call for “humanizing ethnomusicology” to abandon “the pretense of objectivity.” Timothy Rice reflected this departure in his influential article “Remodeling Ethnomusicology” (Rice, 1987). At the heart of this transformation lies the emic/etic antithesis, introduced by Pike (1967) in 1957 to oppose the “insider’s” versus the “outsider’s view” in the researcher’s position toward an object of study. Ever since, this opposition has grown into a schism between Western social and cognitive scientists (Headland, 1990). Harris (1964) adapted Pike’s approach for social sciences, conceptualizing “emic” as a specific culture, *mentally* “native” to an “insider,” whereas “etic”—as cultures, experienced not mentally, but *behaviorally* due to their “foreignness” to an “outsider.” Hence, Harris’ claim that an outsider is capable of only grasping the superficial behavioral patterns through direct observation. Harris’ followers wanted to *abstain* from any “mentalization” of observed facts to avoid their misrepresentation (Harris, 1990). Pike’s followers, in contrary, interconnected mental and behavioral aspects, holding that etics and emics present respectively physical and cultural aspects of analysis, so that an outsider *can* learn to analyze like an insider, and vice versa (Pike, 1990).

For ethnomusicology, emic/etic problem was discussed at the 32nd ICTM Conference, 1993, Berlin. The consensus recognized that insider and outsider perspectives were inseparable and complementary to each other: emic data was to be fit into etic categories, disregarding whether they were actually recognized by the insiders (Baumann, 1993). However, in the following decade Western ethnomusicology became progressively politicized against a supposed “Western bias”—equated with any form of etic evaluation. Some authorities went as far as viewing cross-cultural scientific investigation of music as “cultural colonialism” (see Agawu, 2003).

The purist emic approach replaces the scientific method of investigation with the insider’s description of a native culture in a social context (Myers, 1993, 222–3). The reason for this is that the scientific method by itself is a product of Western civilization (Messner, 1993). Thus, Gourlay (1984) explicitly defies any objective inquiry about music by means of scientific investigation^10^. Becker (1986) declares musical systems as being “incommensurable,” and any scientific study of non-Western music as being “immoral.” She insists that each musical culture should be investigated only in its own native terms and *not* evaluated against another culture—the only way for a researcher to study music is to merge with the indigenous community, learn its language and jargon, and collectively make music. In effect, this utilitarian ethno-unilateral approach to music precludes the study of its origins (Dobzhanskaya, 2012). No wonder, in the West, comparative musicology became abandoned, musical universals denied, and music history fragmented into a bunch of disconnected “histories” (Savage and Brown, 2013). Unfortunately, despite its severe shortcomings, the “emic bias” has penetrated into psychoacoustics (i.e., see Parncutt and Hair, 2011)^11^.

Certainly, not all Western ethnomusicologists abstain from the musicological analysis (Arom, 2010) and deny the validity of objective etic approach (Alvarez-Pereyre and Arom, 1993). Nevertheless, the anti-analytical trend^12^ has taken its toll, establishing a conviction that any research of structural universals is inevitably ethnocentric and inadmissible for ethnomusicology (Nattiez, 2012). Disregarding musical text in sake of musical behavior is symptomatic of a shift away from comparative musicology to fractured sociomusicology of isolated musical communities (Nettl, 2010, 70–92). Many contemporary American ethnomusicological papers are published without a single example of structural analysis to support the author’s claims, basing their claims on entirely behavioral, and not musicological, data—paradoxically conducting *musicological* research without looking into *music per se* (Zemtsovsky, 2002)^13^. Consequently, cognitive scientists interested in comparative music theory and musicological analysis have no choice but to rely on the old publications in English and new ones in other languages (especially those coming from Eastern Europe and Asia, where the influence of politicization is weaker).

The summary of etic/emic arguments, crucial for investigation of TO, demonstrates that proponents of emic approach strongly overvalue it while writing off its fundamental flaws (Table 2).

**TABLE 2:** Pros and cons (P/C) of purely etic, emic, and combined “etic + emic” approaches to analyzing music structures.

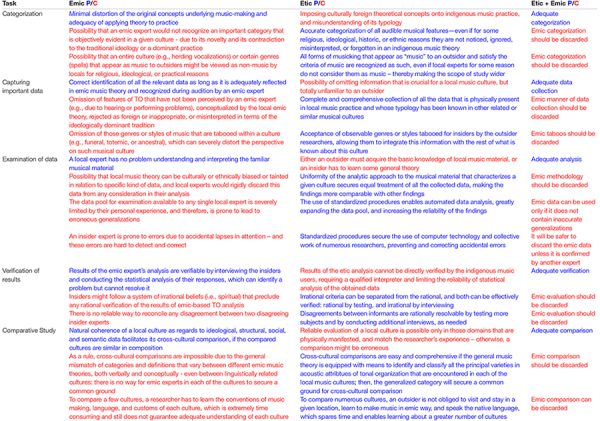

*Pros are colored blue, and cons red. The number of cons for the emic approach (11) doubles the number of cons for the etic approach (5). Emic cons are more detrimental for the outcome of the analysis. The etic approach, even at its worse, still allows the researcher to infer valid principles of TO in a sufficiently large pool of samples of musical styles/genres - which, in the long run, secures correction of mistakes by subsequent researchers. At its worse, the emic approach precludes any comparative study and invalidates the study of TO in an isolated music culture where its members do not regard certain sound production as “music” (i.e., incantations, spells, herding vocalizations). The combined etic/emic approach effectively corrects the shortcomings of a purely etic approach, but in most cases, it fails to correct the shortcomings of a purely emic approach.*

TO is identifiable based on the etic information alone, and its few potential shortcomings are easily amendable by emic references (Dasen, 2012). Purely etic approach has been a status quo in organology, where musical instruments are identified according to etic principles, disregarding emic views (Baumann, 1993). And there is no reason why the entire field of ethnomusicology should not be treated in the same way. The etic approach is unique in enabling a “progressive” accumulation of knowledge where the mistake of one researcher can be corrected by another. Etic self-sufficiency is evident in the fields of ethology and developmental psychology. Neither human babies nor animals can provide emic information—which by no means invalidates the acoustic analysis of their communication.

In light of this, studying TO is paramount for establishing the objective ground for interdisciplinary scientific research of the evolution of music across the synchronic and diachronic varieties of music systems. TO’s role for musicology is comparable to the role of phonology in linguistics: TO specifies a set of acoustic attributes and their oppositions to encode and convey information. Together, they form the “surface level” that underlies the musical syntax and semantics, and provide the material base for any music culture (Cambouropoulos, 2010).

## Tonal Organization Distinguishes Human Music From Animal Communication

The very ability to enjoy “harmonious” sounds most likely emerged as a byproduct of satisfying the need to bring individual emotions in accordance with the interests of a social group (Panksepp and Bernatzky, 2002). Musical anhedonia in humans is exceedingly rare, indicating that music evolved as a direct auditory pathway toward the emotional reward centers in the brain (Loui et al., 2017). Music is probably a human invention that came-into-being to shape important brain functions through the hedonistic effect of appreciating sounds (Patel, 2010). Patel’s (2008) theory of “transformative technology of the mind” reconciled the adaptionist (Darwinian) and the non-adaptionist (Spencerian) approaches, based on the latest cognitive research, and provided the foundation for the theory of “mixed origins of music” (Altenmüller et al., 2013b) that explains how human affective signaling system has transformed the human brain and created music. Emotive specialization and emergence of “musical emotions” must have followed the formation of human auditory-affective circuitry (Altenmüller et al., 2013a).

Centrality of affective signaling brings animal communication closer to music than to speech (Fitch, 2006). Animal signals usually express affective states according to their innate “vocabulary,” are volitionally produced, and are actually felt (Fitch, 2010, 179–81). TO shares more similarities with animal vocalizations than with phonetics, since consonants, crucial for verbal parsing, are unique to human speech—unlike vowels that are more similar to singing and ACs (Kolinsky et al., 2009). Vowels determine verbal prosody which is the primary means of conveying emotions through speech.

Most likely, the musilanguage’s TO resembled the model of vocal production, common for primates and human infants—a reflex-like vocalization (e.g., pain-shrieking), triggered by specific stimuli, and hard-wired for animals but modifiable for humans (Jürgens, 1995). Humans start developing the repertory of cries by differentiating timbral and contour features just a few months after birth (Wermke and Mende, 2009), whereas for most animals, call structure is not modifiable by acoustic experience (Hauser, 1996, 315). Call-learning occurs in a few songbird species, but for most birds, songs are innately encoded, and life experience only activates their retrieval (Marler, 1997).

A call serves as the basic unit in animal communication^14^ and usually conveys specific affective information (Hauser, 2000). Different calls are combinable in “mixed bouts” that are different from “pure bouts” (single call) by triggering a sequence of emotion-based behavioral responses in other animals. Each call’s significance is hard-bound to its acoustic structure. Despite their superficial similarity with music, “mixed bouts” lack transposability of intentions: each call comes only in response to the actual stimulus present in the environment (Zuberbühler, 2017). Transposability is the landmark of music—the same structural pattern is intended to express the same idea across different instances of use, without which musical genres would be impossible: e.g., most lullabies are recognized cross-culturally by their set of structural features (Trehub et al., 1993). Genres are based on reproduction and transposability, and usually form genre systems to support important social practices (Samson, 2001), which enables music to reflect perceptual reality. Animal-learned vocalizations miss such comprehensiveness and generalization. They are limited to:

• display of fitness (Naguib and Riebel, 2014),
• a single season and gender (Slater, 2011),
• mating or defending situations (Slater, 2001).

Syntactically, AC overall lacks a combinatorial organization^15^. It resembles the one-word holophrasic communication of human infants by depending on a directly observable context and on an “analog” signal-emotion correspondence (Johansson, 2005). The same applies to animal “*phonocoding*”^16^ (Marler, 2001): it excludes categorical perception, rhythm, hierarchical structure, and adjacent transitional probabilities (Yip, 2006).

Indispensable for speech and music, compositionality completely eludes ACs—along with listener’s capacity to continually (re)-organize behavior as the song unveils. Non-human communication, as a rule, employs a “one-ended” system: a signaling animal emits a signal unconsciously, not for any specific receiver but as a physiological reflex conditioned to a particular type of stimuli (Hauser, 2000). Such intention-free transmission precludes semiosis^17^ —since sender and receiver must share signs and codes to actually transmit information.

A cumulative “two-ended” semiosis, where the receiver signals in response to the sender and vice versa, is unique to humans, and emerges as a result of technological complexity of human life. Dennett (1983) called this “second-order intentionality”—i.e., the receiver’s beliefs and desires about the sender’s beliefs and desires—in distinction from the “first-order intentionality” that is limited to the receiver alone.

• *First-order* intentionality is characterized by a one-ended conscious processing of unconsciously emitted signal—here, the unintended signaling receives an intentional interpretation.
• *Second-order intentionality* requires a two-ended premeditation of a signal: the signaler has to consider the receiver’s competence, and the receiver must be looking for information while considering the signaler’s circumstances.

Subsequently, the state of knowledge is changed on both ends of such communication, which, so far, has not been found in any non-human animal. Most common for ACs is *zero-order intentionality*—the signaler does not consciously intend to convey a piece of information, but *instinctively* engages a specific signal structure, triggering a similarly automatic response of the receiver.

Two-ended communication generates an unlimited diversity of structure due to infinite recombinations of a finite set of discrete elements that do not carry meaning on their own—what Abler (1989) calls “particulate principle.” It is peculiar to human language and music, finding only embryonal equivalents in a few animal species (Hauser, 2000). Complexity, comparable to human, is evident in some birdsongs, but serves to impress mates and intimidate competitors rather than conveying a specific message (Marler and Slabbekoorn, 2004)—likely forming a parallel (not prototype) to human evolution (Fitch, 2010, 184).

The structural criterion for emergence of the *Semiotically Functional TO* (SFTO)^18^ in music is therefore manifested in the introduction of particulate organization in *phonocoding*.

## The Timeframe of Tonal Organization Obtaining Full Semiotically Functional Capacity

The current consensus holds that music was gradually formed since the appearance of Homo heidelbergensis about 600,000 BP, leading to an artistic “explosion” circa 40,000, when the earliest bone “flutes”^19^ were produced “en masse” (Morley, 2013, 219–25). Although flutes prove the existence of TO in the Aurignacian culture, this tells nothing of whether their sounds served a one- or two-ended communication. In all likelihood, TO did not *communicate* musical emotions but merely *accompanied* the behavioral display of actual real-life emotions—as it happens in reflex-driven animal vocalizations (Seyfarth and Cheney, 2017). Their acoustic form is shaped by the physiological impact of emotion on the vocal organs plus Pavlovian-style priming.

Semiosis originates in an ongoing interaction between signalers and receivers within the reference-framework of the *same* environment—forging communication rules through the dialectics of ritualization and devaluation (Wiley, 1983). Ritualized signals establish conventions via encoding/decoding interaction between the acquainted individuals. Once established, convention becomes “devalued”—abused by “bluffing calls” of the unacquainted signalers trying to take advantage of the established reactions of the receivers. Increase of dishonest signaling causes the signaler to substitute the signal or modulate it along a single acoustic dimension until an “evolutionary stable strategy” is formed, marking a stationary equilibrium within the population—which ultimately fixes the convention (Maynard-Smith, 1976). Here, “signaling efficacy” obtains its formative power: as natural selection optimizes a signal to support the signaler’s visual display, successful decoding starts relying on whatever the receiver finds most comfortable to detect, discriminate, and remember (Guilford and Dawkins, 1991). Together, strategic design and efficacy determine the ultimate structure of a signal.

The road from animal call to musical phrase goes through the ritualization of innate physiological and behavioral cues that animals use to exchange information (Maynard-Smith and Harper, 2003)^20^. Ritualized signals differ from cues by being more conspicuous, redundant, stereotypical, and containing alerting components (p. 72). Nevertheless, they remain “concrete” (bound to a single context) like cues (Fitch, 2010, 184) and unlike “transposable” music. For ritualized signal to evolve into musical phrase, its meaningful features must be abstracted to become non-signal-specific and form an AE of TO—a conventional dimension of gradient change along some axis.

**FIGURE 1:**
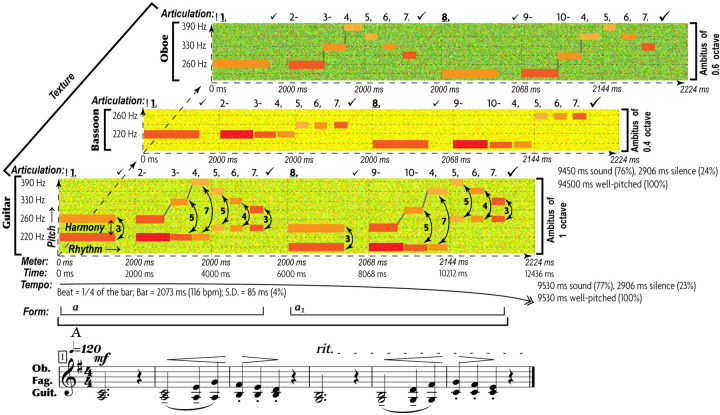
11 AEs in a musogram of classical instrumental music. At the bottom of the figure, the conventional musical notation represents the same content as the three musograms above it. The lowest musogram (guitar) contains all the AEs marked out and named. Its horizontal axis (horizontal dashed arrow) represents time, vertical axis (vertical dashed arrow) frequency, depth axis (diagonal dashed arrow) the aspect of **texture**. The latter joins all three musograms. Small colored rectangular bars indicate tones. Their vertical relation represents **pitch**, with dash guidelines referencing frequency values. The changes in distance between the concurrent (superimposed) rectangles indicate **harmony**. The rectangular length represents **rhythm**. The breaks and the gray lines that connect the consecutive rectangles as well as the numbers above the frequency grid comprise an aspect of **articulation**. Each tone is numbered, checkmarks indicate pauses (the bigger the pause the larger the checkmark), and punctuation signs reflect the grouping of tones. Dashes mark the connected tones (legato), commas—disconnected tones within the same phrase, periods—the end of a phrase, and exclamation marks—the phrasal opening. Bold and underlined numbers indicate anchor-tones (stressed by duration, dynamics, and frequency of occurrence). The gray lines represent connectivity: discrete pitches are connected by vertical lines, whereas portamento pitches by tilted lines. The coloring of rectangles represents **dynamics:** from the loudest in yellow to the softest in blue. Thin vertical dashed lines indicate **meter**—inferred from well-articulated occurrences of anchor-tones and longer rests. **Tempo** averages all metric units, expressed in msec and beats-per-minute. The standard deviation shows how flexible the tempo is. A solid arrow with a double arrowhead reflects the tempo changes: ascending for accelerations, while descending for decelerations. **Form** reflects the thematic organization of the material, indicated by horizontal brackets and letters: thinner brackets and lowercase letters for motifs, and thicker brackets and uppercase letters for phrases. Each new material is marked by a new letter, and variation—by a subscript number. **Register** is represented by the coloration of the grainy filling of the ambitus: from a deeper green for the darkest timbre to yellow for the lightest timbre. In this example, oboe uses its darkest register, bassoon—its faintest register, whereas guitar—its medium register. **Harmonicity** (see Table 3) is indicated by the relative thickness and the geometric shape in representation of tones: the greater the harmonic richness, the thicker the rectangular bars, whereas the noisier the sound, the more irregular the fuzzy shapes (not present in this particular example). For thorough explanation of this method of visualization see Appendix 1 in Supplementary Material.

The end result of such abstraction is the multifactorial nature of music communication (Figure 1): each emotional/motivational state is represented not by a *dedicated signal* but by the configuration of *numerous AEs* (Juslin, 2005). Conventional musical notation is poorly suited for incremental representation of AEs other than rough indications for melody/harmony, rhythm/meter, and form. Waveforms display rhythm and dynamics in finer detail, but miss other AEs. Spectrograms decently represent melody, rhythm, articulation, register, harmonicity, and dynamics, but miss harmony, tempo, meter, and texture. This necessitates the use of a special notation—such as *prosogram*, developed by Mertens (2004) for analyzing speech. Although applicable to monophonic vocal music in visualizing pitch, rhythm, articulation, dynamics, harmonicity, and register, prosogram ignores harmony, tempo, meter, texture, and form. To overcome these limitations, I propose a similar approach to music—“*musogram*^21^.” Its advantages over conventional notation in capturing 11 AEs are demonstrated in the simplest case of classical music (Figure 1). It introduces the conventions, necessary to read the upcoming figures.

Multifactorial visualization reveals the expressive contribution of all AEs. Each AE features structural patterns representing specific emotional states across cultures, genres, and styles—at least for basic emotions (Table 3)^22^. Configuration of such patterns distinguishes one emotional expression from another. If multiple expressions share the same pattern of AE (e.g., legato characterizes both sadness and tenderness), the combination of a few aspects (e.g., “articulation + meter”) differentiates them.

**TABLE 3:** The configuration of structural patterns for each AE, typically used to express five basic emotions.

**Acoustic domains**	**Aspects of expression in music**	**Range of the expression of an aspect**	**Happiness’ acoustic markers**	**Sadness’ acoustic markers**	**Anger’s (aggression) acoustic markers**	**Fear’s (anxiety) acoustic markers**	**Tenderness’ (love) acoustic markers**
1. Frequency	1. Melodic pitch (consecutive “linear”)	High/low relation of tones	Prevalence of ascending contour within a diverse set of contours, wide ambitus, leaps, sharp zigzags, and sharpened intonation	Prevalence of descending and wave-like contours, narrow ambitus, mainly stepwise motion, flat and sliding down intonation	Prevalence of ascending contour, with little diversity of other contours, frequent leaps with sharpened melodic contours and tendency to short motifs	Prevalence of ascending contour, of little variation, wide ambitus, many interruptions, frequent leaps, including extreme, use of angular and wave-like intonations	Fairly narrow ambitus, prevalent steps with occasional leaps, rising intonation, wave-like shapes
	2. Harmonic pitch (concurrent “vertical”)	Concordant/discordant combination of tones	Major, diatonic, prevalence of medium size perfect intervals of fourth and fifth	Minor, chromatic, dissonance, prevalence of small intervals	Minor, chromatic, strong dissonance (up to atonal), large intervals, esp. major seventh, augmented fourth	Minor, strong dissonance, diverse intervals	Major, diatonic, general consonance
	3. Form (thematicism)	Sameness/diversity	Relative simplicity	High complexity	High complexity	Relative complexity	Quite low complexity
2. Time	4. Tempo	Fast/slow metric pulse	Mostly fast, with very restricted use of rubato	Slow, with strong rubato and prevalence of ritenuto	Fast, with minimal rubato and general tendency to use accelerando	Fast, with strong rubato and many abrupt changes	Slow and moderate, strong rubato but no abrupt changes
	5. Rhythm	Short/long relation of tones	Sharp contrasts of tones, yet smooth succession of groups of tones	Smoothened contrasts of tones, yet firm patterns, many long tones	Very sharp contrasts of tones, complex patterns with sudden changes, many short tones	Abrupt changes of tones and rhythmic groups, with overall diversity in rhythm, many short tones	Smoothened contrasts of tones, yet with rhythmic diversity, many long tones
	6. Meter	Short/long periodicity of stressed beats	Strong regularity with minimal deviations	Tendency to irregularity	Tendency to syncopation and irregularity	Pronounced irregularity and variability	Strong regularity with moderate variability
	7. Articulation	Styles of attaching/detaching of successive tones	Prevalence of staccato, with overall great diversity of styles	Prevalence of legato, with little diversity of styles, many pauses	Prevalence of staccato, with moderate diversity of styles, occasional legato	Prevalence of staccato (stressed), with great diversity of styles, many pauses	Prevalence of legato, with little diversity of styles, many pauses
3. Amplitude	8. Dynamics	Loud/soft relation of tones	Prevalence of loud and medium loud, with limited crescendo and diminuendo	Prevalence of soft and medium soft, with medium crescendo and diminuendo	Prevalence of very loud, with very little dynamic change, accents tend to fall on unstable tones	Prevalence of soft and medium soft, yet with diverse dynamic changes, mostly abrupt	Prevalence of medium soft, few dynamic changes, accents tend to fall on stable tones
4. Timbre	9. Register	Relation of homogenous groups of tones in their tonal quality	Prevalence of bright register, raised singing formant, brightness	Prevalence of bright register, low singing formant, dullness	Bright register with little changes, raised singing formant, harshness	Prevalence of bright register with abrupt registral changes, general mellowness	Prevalence of dark register, lowered singing formant, general mellowness
	10. Harmonicity, attack and vibrato	Periodic/non-periodic spectral content	Harmonic richness, fast attack, medium vibrato with mid-fast rate	Harmonic scarcity, slow attack, small vibrato range with slow rate	Harmonic richness, much spectral noise, fast attack and decay, large vibrato range, mid-fast rate	Contrasts of harmonic richness and scarcity; gentle attacks; small irregular vibrato with fast rate	Harmonically moderate, slow attacks, small vibrato range with mid-fast rate

*This table is compiled based on a number of meta-reviews of experimental research on emotional responses to listening to music (Gabrielsson and Lindström, 2001; Gabrielsson and Juslin, 2003; Juslin and Laukka, 2003; Juslin, 2005). The data is categorized according to the musicological nomenclature: all acoustic attributes are broken into 10 AEs across 4 acoustic domains. The aspect of texture is missing, because it was not controlled for in the experimental studies of the acoustic structural patterns that characterize “musical emotions”. The aspect of harmonicity constitutes an organic part of the aspect of instrumentation, listed in the beginning of this paper. This potentially confusing mismatch occurs as a result of the discrepancy in musicological and psychoacoustic scholarships: as a rule, musicians are ignorant of harmonicity, while psychoacousticians are ignorant of instrumentation. Harmonicity can be defined as the extent to which the spectrum of a complex tone is made of its component frequencies that are integer multiples of its fundamental frequency (FF). This is usually measured as the ratio of harmonics to noise. Slow attack and great vibrato generally tend to reduce harmonicity in a monophonic tone.*

Multifactorial particulate semiosis shapes musical signs—each AE features SFTO, which enables “natural selection” for the most effectively communicated expressions. AC can be multifactorial but lacks particulate semiosis. Verbal semiosis is particulate but mostly unifactorial: phonetic organization is its primary source^23^.

Basic emotions can be recognized across musical cultures (Mohn et al., 2010) and can be acoustically described (Eerola and Vuoskoski, 2013). Therefore, at least some of their musical markers share biological roots with mammalian ACs (Zimmermann et al., 2013). The birth of SFTO is trackable by comparing the multi-cultural markers of typical musical expressions of basic emotions to equivalent AC expressions and by inferring their differences and commonalities (Table 4). Common traits indicate music’s inheritance from ACs, whereas contrasting traits—innovations brought about by cultural evolution.

**TABLE 4:** Acoustic attributes of typical animal vocalizations used by different species to display their affective state, grouped according to AEs of human music.

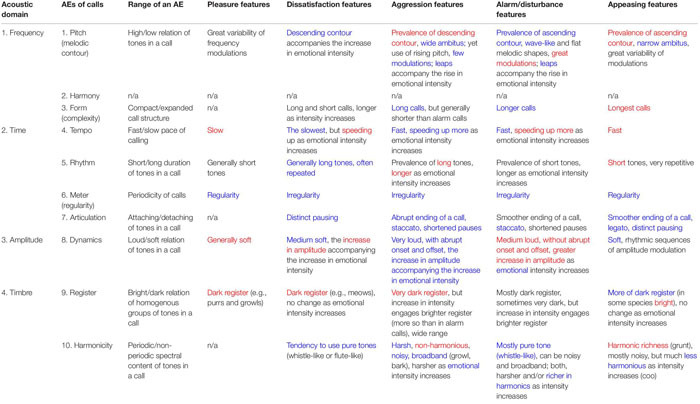

*The data for this table is compiled from numerous meta-reviews (Morton, 1977; Peters, 1984; August and Anderson, 1987; Snowdon, 2003; Briefer, 2012; Altenmüller et al., 2013; Zimmermann et al., 2013; Snowdon et al., 2015). According to the classification scheme of Brudzynski (2013), human and animal affective states are equated in the following ways: human “happiness” is equated to animal “pleasure” (satisfaction), human “sadness”—to animal “dissatisfaction” (social isolation from a bonded party), human “anger”—to animal “aggression” (agonistic behavior, conflict with display of threat or combat), human “fear”—to animal “alarm/disturbance” (anxiety at the presence of threat or intimidation by a novel environment), human “tenderness/love”—to animal “appeasing” (affiliation—physical contact without agonistic behavior, e.g., grooming, and play). Those acoustic features that agree between human and animal expressions of the same affective state are marked blue, whereas the disagreeing features—red. Features that are not covered in research literature are marked “n/a.” The aspect of “harmony” is clearly not applicable to animal vocalization. The aspect of “form” bears only distant relation to “musical form”: AC’s compactness loosely corresponds to simplicity of structure, whereas lengthiness—to complexity. Aspect of “meter” also finds only partial correspondence in regularity or irregularity of call units in the AC bouts. The timbral coloration is reflected by the aspect of “harmonicity” rather than “instrumentation” that manages timbre in human music.*

Music and ACs have in common only regularity/irregularity and articulation. They both find a perfect match between human music and AC (5 out of 5 emotional states). The next closest match (4 out of 5) is “harmonicity.” That is why these two aspects of TO (articulation and harmonicity) must be the most ancient, possibly retained from the pre-human times. In contrary, “register” shows a nearly perfect mismatch, testifying that humans cardinally reorganized the use of registers in music. The rest of the AEs display mixed results. If to generalize by emotional states rather than by expressive aspects, then none of the emotions display a full match or a full mismatch. Evidently, coding of emotions in human music has developed its own proprietary acoustic attributes. This confirms that ACs are mostly conspecific. Heterospecific^24^ generalities support only a rough distinction between “positive” versus “negative” emotions (Snowdon et al., 2015). Human communication inherits from ACs just 2 general semiotic oppositions: (1) positive/negative affectation and (2) low/high intensity of an affective state (Brudzynski, 2013). High-intensity “strong emotions” (Grewe et al., 2005) have evolved into chill-like experiences of music—in contradistinction to the “mundane” use of language (Silvia and Nusbaum, 2011). However, “strong emotions” *per se* could not support musical semiosis because the stimulus-response relationship between chill and music structure has not been experimentally reproducible—music chills seem to occur intermittently (Altenmüller et al., 2013a).

Both incremental and gradual changes in multiple AEs (Table 1) are peculiar to human music, whereas holistic tempo, dynamics, rhythm, and melodic contours are mutual for music and ACs. Musical meter, articulation, and harmony are also traceable to, respectively, ACs’ regularity/irregularity, pausing/continuing, and periodicity/harshness.

However, the cross-examination of TO in expression of 5 basic emotions in music versus ACs reveals that many AE’s patterns are unique to music (Table 5). Moreover, humans completely invert the acoustic characteristics of animal’s affective states:

**TABLE 5:** The acoustic attributes of typical expression of 5 basic emotions in human music that find no correspondences in animal communication (based on Tables 3, 4).

**Acoustic domains**	**AEs of music**	**Range of an aspect**	**Happiness features**	**Sadness features**	**Anger (aggression) features**	**Fear (anxiety) features**	**Tenderness (love) features**
1. Frequency	1. Melodic pitch (consecutive “linear”)	high/low relation of tones	prevalence of ascension in the overall diverse contours, leaps, zigzags, sharpened intonation, wide ambitus	smooth contours, mainly steps, wave-like shapes, flat and falling intonation, narrow ambitus	prevalence of ascending contour, with sharpened shape of melodic contours	little variation in contours, interruptions, use of angular shapes, wide ambitus	prevalence of descending contour, mostly steps with occasional leaps, rising intonation, wave-like shapes
	2. Harmonic pitch (concurrent “vertical”)	concordant/discordant combination of tones	major, diatonic, prevalence of perfect 4th and 5th	minor, chromatic, dissonance, mostly small intervals	minor, chromatic, strong dissonance, wider intervals (major 7th, aug. 4th)	minor, strong dissonance	major, diatonic, general consonance
	3. Form (complexity)	sameness (simplicity)/diversity (complexity)	relative simplicity	high complexity	n/a	n/a	low complexity
2. Time	4. Tempo	fast/slow metric pulse	fast, with very restricted rubato	strong rubato and prevalence of ritenuto	minimal use of rubato	strong rubato and many tempo changes	slow and moderate, with moderate rubato
	5. Rhythm	short/long relation of tones	sharp contrasts of tones, yet smooth succession of groups	smoothened contrasts of tones due to frequent use of rubato	very sharp contrasts of tones, complex patterns with sudden changes	abrupt changes of tones and groups, general prevalence of diversity	smoothened contrasts of tones due to rubato use
	6. Meter	periodicity of beat grouping	minimal deviations	n/a	tendency to syncopation	pronounced variability	moderate variability
	7. Articulation	styles of attaching/detaching of successive tones	prevalence of staccato, great diversity of styles	prevalence of legato, with little diversity of styles	moderate diversity of articulation, occasional legato	stressed staccato (marcato), great stylistic diversity, many pauses	little diversity of styles
3. Amplitude	8. Dynamics	loud/soft relation of tones	prevalence of loud and medium loud, with limited crescendo and diminuendo	prevalence of soft, with medium crescendo and diminuendo	mostly loud, with very little dynamic changes, accents tend to fall on unstable tones	prevalence of soft and medium soft, yet with diverse dynamic changes, mostly abrupt	few dynamic changes, accents tend to fall on stable tones
4. Timbre	9. Register	bright/dark relation of homogenous groups of tones	prevalence of bright register, raised singing formant, brightness	prevalence of bright register, lowered singing formant, dullness	prevalence of bright register, with little change, raised singing formant, harshness	prevalence of bright register with abrupt registral changes, mellowness	prevalence of dark register, lowered singing formant, mellowness
	10. Harmonicity, attack and vibrato	periodic/non-periodic spectral content of tones	harmonic richness, fast attack, medium vibrato with mid-fast rate	slow attack, little vibrato with a slow rate	harmonic richness, fast attack and decay, large vibrato with a mid-fast rate	gentle attacks; little and irregular vibrato with a fast rate	slow attacks, little vibrato with a mid-fast rate

*These attributes constitute a stock of TO features developed in the process of evolution of human music from hominin musilanguage. This includes changes in vertical harmony, in metric pulse, and in complexity of musical form; contrasts in melodic contour, in directionality of melodic intervals (sharpening for ascending, flattening for descending dyads), and in thematic material; diversity of rhythm, articulation and tempo; and ambitus size. Animal vocalizations do not seem to engage these categories in meaningful differentiation of calls.*

• Ascending/descending pitch (anger-tenderness),
• Fast/slow tempo (happiness-tenderness),
• Soft/loud dynamics (happiness-fear),
• High/low register (happiness/sadness-anger/fear),
• Harmonicity/inharmonicity (tenderness-anger).

This indicates massive remapping of the instinctive vocal encoding of affective states, achieved throughout the cultural evolution of Homo.

What could have caused such changes?

For many AEs, their cultural origin is obvious: metric pulses usually break into a default binary pulse (Potter et al., 2009), following the left/right paradigm instituted by bipedalism (London, 2004). Rubato patterns (ritenuto/accelerando) also relate to bipedal locomotion (Honing, 2003), so as tempo which is synchronizable to gait or heartbeat (Fraisse, 1982). Melodic intervals follow another locomotive paradigm of stepping/leaping (Nikolsky, 2015b)—each successive tone either “stands” (unison), “steps” (2nds and fast 3rds), or “leaps” (>3rd)—unlike harmonic intervals that are factored by consonance/dissonance relations (a much later historic semiotic development). Articulation grouping relies on yet another biological factor—the breathing cycle (Alekseyev, 1976, 130). Taking a breath terminates a phrase, imposing a “clausal structure” on the melody (Fenk-Oczlon and Fenk, 2009b). The “breath group” prototypes the “articulation group” via a “breathing pulsation” (Etzel et al., 2006). Noteworthy, breathing pulse takes over metric control in ametric forms of music-making (Wallin, 1983). Locomotive and respiratory AEs must have formed long before Homo.

The rhythmic aspect of music possibly emerged from the quantification of verbal rhyming, following the language development (Kharlap, 1972)^25^. Melodic contours also relate to verbal prosody. The timeline of language formation remains controversial: the “saltational” scenario regards language as a sudden mutation 50–100 kya, whereas the “gradual” scenario qualifies it as part of evolution throughout millions of years (Hillert, 2015). Paleoneurology points to the Middle Pleistocene as a birthtime of language (Quam et al., 2017). Since musical rhythm and melodic contours rely on fine vocal control, their addition to TO must have followed the accumulation of extensive lexic vocabulary within a phonological organization of language (Tallerman, 2013). This ties the emergence of multifactorial TO (which is hardly possible without engaging melodic contour and rhythm) to Homo sapiens and the Upper Paleolithic, as indicated by the proliferation of bone “flutes.” During 1995–2009, over 120 bone pipes were recovered across Europe, dated 36–30 kya and concentrated up to 3 “flutes” per cave (Conard et al., 2009). Evidently, melodic music suddenly became popular in the Aurignacian.

Discreteness of pitch is evident in the construction of Paleolithic “flutes”: holes are drilled in particular spots in order to generate sound of a particular pitch, and there is evidence of common patterns in the intervallic distances between the placement of the holes, suggestive of the commonality of certain melodic intervals in Aurignacian music-making (Nikolsky, 2015b, Appendix II). Discreteness of pitch was very likely to have been accompanied with the discreteness of rhythm, since stressing a pitch as a rule relies on extending its time-value relative to other pitches. Pitch hierarchy is supported by rhythmic contrasts between shorter timing of modally insignificant pitch-classes as well as longer timing of modally important pitch-classes (Krumhansl, 1990).

However, Aurignacian music most certainly lacked SFTO—semiotization of rhythm and directionality requires an extensive period of exploration. This is obvious in the acquisition of musical skills throughout infancy: infants babble—engage in meaningless play with melodic contours—before learning to compose musically *expressive* vocalizations (Moog, 1976; Dowling, 1984; Swanwick et al., 1986; Holahan, 1987; Hargreaves, 1996). Most children pass through a music-babbling stage when 12–18 months old (Gembris, 2006). Universality of babbling suggests the universality of prolonged sensorimotor trials in music-making before semiotic rules are formed. Babbling abstracts melodic directions and intervals, allowing an infant to master particulate semiosis. Similarly, early humans had to long experiment with meaningless melodic play for the SFTO conventions to emerge.

## Cross-Cultural “Scripts” in the Formation of Semiotically Functional Tonal Organization

Tool-making technologies (Ambrose, 2001) and “social scripts”—i.e., fixed generalized patterns of social behavior (Aiello, 1998)—most likely served as syntax precursors by providing explicit models for combining numerous elements into a structured sequence (Wildgen, 2004). Paleolithic proxies for syntactical language include composite tools (Ambrose, 2010), fire (Brown et al., 2009), knot-making (Camps and Uriagereka, 2006), cooperative hunting (Chase, 2006, 52), symbolic behaviors (Mcbrearty and Brooks, 2000), and burials (Mellars, 2004). The same proxies apply to syntax-related features of musical TO. All the AEs of music listed above (perhaps, except harmonicity) are engaged in the syntactic organization of music. Phrasal ends are usually marked by descending pitch, lower register, more concordant harmony, slowing of tempo, longer rhythmic value(s) placed on metrically strong time, reduction in loudness, and clear caesuras in articulation which separate the end of one formal unit (phrase, sentence) from the beginning of the following unit. In addition, there is evidence of a link between structures of tonal and social organization in indigenous societies (Blacking, 1967; Davidson, 1970; Lomax, 1977; Berliner, 1993; Arom and Voisin, 1997; Kubik, 1999)—which indicates that social structures might have also served as proxies for music syntax.

Making bone “flutes” was extremely tedious, demanding skills and expertise (Münzel and Conard, 2009). Why to invest into a “pitch toy” rather than to merely vocalize?

Cave-inhabitants must have supported flute-makers in the same way as they supported cave-artists—their exquisite labor required narrow specialization, precluding participation in hunting/gathering. In animistic ideology, depictions linked hunters to prey, providing means to benefit the outcome of hunting (Hauser, 1999, 1–4). Magic—not aesthetics—governed rock art, turning depiction into a shamanic occupation^26^. Shamanic music resembles shamanic depiction by cross-linking the signified to the signifier (Hubbard, 2003). In northern shamanic traditions, both melodic and pictorial contours are believed to affect the corresponding real objects (Novik, 2004, 67–85). Archeological evidence also links most resonant locations in caves with rock art in Paleolithic sites, suggesting the combined ritualistic use of images and music (Reznikoff, 2008; Morley, 2013; Mills, 2016). Hence, a Paleolithic “flute” was most likely a talisman used in rituals (Marshack, 1990). Its manufacturing from the bone of a particular animal (Wyatt, 2016) must have carried more significance for Aurignacians than the pitches it produced.

For melodic semiosis to occur, rhythm and directionality must first be abstracted into AEs. Abstraction of directionality probably followed rhythm: *salience of the melodic direction depends on rhythmic values, but not vice versa*. Tracking the melodic contour within the tonal “grid” constitutes the backbone of melodic organization (Deutsch, 2013), just like tracking the rhythmic grouping within the metric grid supports the temporal organization (Large, 2008). Reference to tonal hierarchy interferes with rhythmo-metric perception by biasing the attention toward pitch (Prince et al., 2009). Their conflict indicates that users of non-Western music discriminate rhythmo-meter better than users of Western tonality (which agrees with the observations of ethnomusicologists). This suggests that *frequency reference-frame* emerged later than *rhythmo-metric*.

Developmentally, acquisition of rhythmic hearing usually precedes melodic hearing (Shatkovsky, 1986). Infants seem to acquire rhythm-discrimination skills earlier than pitch-discrimination (Trehub and Hannon, 2006)^27^. The perceptual foundations of rhythm/meter are manifested just a few days after birth, as a part of developmentally crucial rhythmic interaction between infants and caregivers, occurring spontaneously and requiring little experience—reflecting its evolutionary importance for bonding (Trainor and Hannon, 2013). In verbal acquisition, rhythm too obtains semantic functionality earlier than prosodic contour (Shvachkin, 1948). According to the vast data collected through administration of early musical education in USSR, rhythmic hearing lays the foundation for vocal musical skills—followed by learning to reproduce melodic contours (Kirnarskaya et al., 2003, 168–170). Impressions that not only rhythm can influence melodic perception by directing the attention to longer tones, but that melodic features carry the reverse influence onto rhythm, are based on the misnomer between rhythm and meter (McAuley, 2010). Melodic intervals, contours, and “tonal accents” help to infer meter, but play no major role in identification of rhythmic values. On the contrary, judgments of melodic similarities are significantly affected by rhythm, especially in folk music (Eerola et al., 2001)^28^. Even for experienced Western musicians the distinction between rhythms is more salient than the distinction between pitches (Monahan and Carterette, 1985)^29^.

Important Upper Paleolithic cultural proxies promote the abstraction of rhythm—not of melodic contour. Metric pulse is transposable from bipedal gait into such a common Paleolithic activity as stone-knapping. Each knapper prefers his own tempo and rhythm (Whittaker, 1994, 81)—quite similar to individual gait preferences (Whittle, 2007). Knappers’ heartbeat provides a metric reference (Zubrow and Blake, 2006). Two knappers might have accidentally discovered the expressive capacity of rhythm through their entrainment, thereby forming the world’s first musical instrument (Montagu, 2004). Group “musical” knapping was observed amongst Aboriginal women in Queensland (Duncan-Kemp, 1952, 27). Rock slides and gongs are drummed across the globe in rituals related to fertility cults (Fagg, 1997, 38). The ritualistic context provides feeling of contentment or awe, abstractable into a semantic value for the knapping/grinding sound, turning its rhythm into a sign—and the archeological evidence for collective stone-knapping is present in Neolithic sites at Sanganakallu-Kupgal, India (Boivin et al., 2007). Even earlier, stationary lithophones were drummed in Solutrean-Magdalenian caves (pecked rock surfaces were found in Africa)—suggestive of the existence of portable lithophones (Blake, 2011). The weird-sounding cave echo might have prompted specific affective connotations (Cross and Watson, 2006).

Unlike rhythm, *pitch directionality* finds no proxies in the Paleolithic^30^. A set of meaningful pitch contours could have originated in verbal prosody, but paleolinguists connect the development of the fully phonemicized semantic languages to population growth *after* the Last Glacial Maximum (Robb, 1993). Deeply social, language is imperative for accumulation of knowledge, which depends on population density to avoid “bottlenecks” due to climate changes and extinctions. Cultural evolution stabilized only after 50 kya—most certainly, because of the advancement of language (Klein, 2009). In all the prehistory, the transition to Holocene stands out as the grand leap in innovation, called to subsist an ever-growing population (Richerson et al., 2009). Powell et al. (2009) developed a demic model to estimate the critical population density capable of sustaining the innovation growth to offset the innovation loss: for Europe it was 45 kya. Prior to 20 kya, prehistory consisted of a chain of major discontinuities in cultural transmission (d’Errico and Stringer, 2011). Technically, the archeological concept of “culture” applies only starting from the Neolithic (Probst, 1991, 227).

The first archeological symbolic “culture” of pan-European scale is the Gravettian, whose common trans-European traits are both socio-economic and spiritual, with regional differences confined to the material techno-complex (Kozłowski, 2015). The continent-wide cultural unity is evident in the omnipresence of “Gravettian Venuses” over most of Europe (Soffer et al., 2000)^31^. Denser population turns language from means of inter-group cooperation that compensates for local ecological deficits into a life-long ethnic marker, akin to the cranial configuration (Robb, 1993). Personal ornaments in Gravettian burials manifest similar function of the “ethnic badge,” differentiating age classes across the puberty threshold (Zilhão, 2014).

Social restructuring by ethnos and age hardly occurred without the involvement of music, closely affiliated with funeral and puberty rites. The Gravettian funerary practice strongly suggests the existence of burial rituals regulating the emotive interaction between the group’s members, the dead, and the landscape as part of a greater ritual system, underpinned by cosmological beliefs (Pettitt, 2010). The remnant of such socio-eco-cosmological interconnection with TO, providing its semantic foundation, is the ancient doctrine of **ethos**^32^ —renowned in Hellenic civilization (Mathiesen, 1984), but certainly much older (Farmer, 1965) and geographically wider (Manuel and Blum, 2011). The roots of ethos must lie in the Gravettian trans-European spiritual unity.

## Contribution of Multi-Dimensional and Multi-Emotive Semiosis to the Evolution of Music

Human melodic universals remap animals’ universals. Animal anger is characterized by descending contour, whereas animal appeasing—by ascending contour. Music reverts the registers for happiness, sadness, fear, and anger from low to high. Why?

*Music contributes to the conservation of knowledge* by bonding social groups and incentivizing linguistic communication. This capacity came in play after the Younger Dryas (11 kya), when global warming enabled colonization of Eurasia. Widely dispersed populations created a few flexibly bounded “social territories^33^,” developing the dialect continuums by linkages among groups due to intermarriages during population shortfalls (Robb, 1993). Population growth and sedentism accompanied rapid neolithization, promoting ethnogenesis and thereafter fissioning language into language families as regional cultural differences cumulated (Robb, 1991). Such line of development benefited from the social bonds established by music.

The absence of music-like particulate emotional communication must be one of the reasons why chimpanzees do not accumulate cultural traditions. Some chimpanzees acquire a culture of tools but due to the lack of transposability and abstraction cannot transmit it (Whiten, 2011). However, it is music, not language, that engages reproduction, transposability, and abstraction of idiomatic patterns of each of its AEs.

Human remapping of pitch encoding most probably originates from the continuous practice of:

• **Frequent rotation between aesthetic emotions:** ACs prioritize negative emotions due to greater urgency of their triggers (August and Anderson, 1987). Human music is balanced between negative and positive expressions because of the mentalization of aesthetic emotions (Juslin, 2013). Expression of negative emotions can be pleasurable whenever it occurs in a non-threatening situation, is aesthetically appealing, and seems somehow useful or appropriate (Sachs et al., 2015). Thus, abstraction of emotions enables older children to learn to appreciate sad music (Schubert and McPherson, 2015), whereas at 5–7 months, infants overwhelmingly prefer happy to sad music (Nawrot, 2003). By 4 years, children start intentionally expressing positive and negative emotions in singing (Welch, 2006), distinguishing happy/sad and angry/fearful musics (Eerola and Vuoskoski, 2013). This line of development is also applicable to cultural evolution. In both cases, changes of musical emotions sharpen *contrasts in patterns of their musical expression*—resembling *phonemic oppositions in phonology*.
• **Multifactorial musical semiosis:** Zero- and first-order intentionality separates animal signals from second-order intentionality of humans (Seyfarth and Cheney, 2017). Although non-human primates can coordinate the produced signal with the listeners’ response, modulating the acoustic features of their calls accordingly, modulation usually engages a single parameter—falling short of the complex multidimensional nature of emotional communication in verbal prosody and music (Filippi, 2016). Simultaneous interactive control over *multiple AEs* is peculiar to music alone. Thus, in expression of anger, prevalence of ascending contour and high register conveys physical strain, while the side-effect of their monotony is compensated by a diverse contrasting rhythm and spectral content, projecting agitation (Table 2). AC’s anger does not engage such interaction. It conserves a *unifactorial* timbral quality^34^ (Table 3).

**FIGURE 2:**
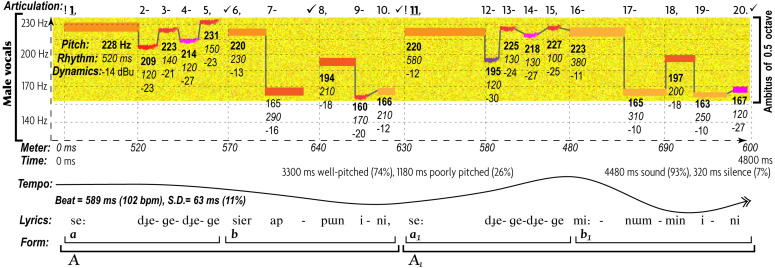
Characteristic patterns of AEs in expression of love in a Yakut traditional lyrical song “Sae Dyige” (may be auditioned at http://chirb.it/sNegG1). By Juslin’s (2005) classification this song fits the “love” music category—in agreement with its lyrics, describing how a woman is anticipating visits of her multiple lovers (Alekseyev and Nikolayeva, 1981, 86). The musogram follows the same conventions as Figure 1, with minor additions due to the less definite use of pitch in the purely vocal music. Tones of low spectral periodicity (noisy or spoken-like) are represented by fuzzy strips in contrast to high periodicity, represented by rectangular bars. The number under each pitch displays its frequency value in bold, its duration in italic, and its maximal amplitude (the highest value of any of its spectral constituents) in regular font. The lyrics are given in the phonetic transcription. There are two contrasting motifs: “a”—a sustained long anchor tone (tonic function), followed by rapid alternation of steps with rising intonation; and “b” —two descending intonations, the first of which leaps to the alternative anchor (dominant function to mark a cadence), while the second steps down and then gently rises. These two motifs make up a call-like phrase that is regularly repeated. Song is characterized by a narrow ambitus (half-octave), mid-low register, high harmonicity, low complexity, moderate tempo (102 bpm) with little rubato (11%), diverse rhythm (usage of four rhythmic values), regular meter, overwhelming legato (97%), and scarce dynamic changes. For more detailed discussion, see Appendix 2 “A Comparative Structural Analysis of Musograms.”

**FIGURE 3:**
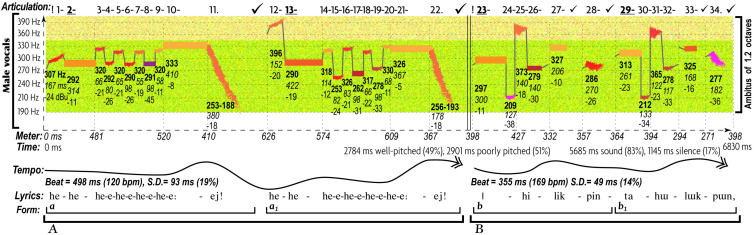
Characteristic patterns of AEs in expression of anger in a song of the underworld virgin from the olonkho “Djiribina Djirilatta” (http://chirb.it/sCq02k). This excerpt from the traditional Yakut epic expresses anger of the evil sorcerer toward the heroine, challenging her to a fight (Alekseyev and Nikolayeva, 1981, 35). Structural descriptors of most aspects of this song fall in the category of “angry” music (Juslin, 2005). The acoustic markers of all AEs contrast those in Figure 2. The ambitus is over twice wider. There are two registers instead of one: low singing and high “shouting”), both are higher than Figure 2. The share of well-pitched sounds in the overall duration of music is reduced by 34%. The share of staccato articulation is increased (by 142% in the duration of silence and 40% in the number of pauses). Tones are overall shorter and 50% more diverse in time values, with contrasts between rhythmic groups. The tempo contains abrupt switches, the fastest of which is 66% faster and 73% more variable (rubato) than Figure 2. Intonations feature wide leaps, on average 70% wider than Figure 2. Thematically, the music is more diverse and complex, using two contrasting materials, “A” and “B” (Figure 2 had only one). Timbre is harsh (a heightened larynx and intensified pressure).

All AEs differ in musical expression of love (Figure 2) and anger (Figure 3), as evident in musograms^35^ of indigenous Siberian songs that Russian theorists believe to represent the earliest forms of TO (Alekseyev, 1976, 1986; Brodsky, 1976; Zemtsovsky, 1983; Mazepus, 1993; Mazepus and Galitskaya, 1997; Novik, 1999; Zabolotskaya, 2009; Dobzhanskaya, 2011, 2016; Nikolsky, 2015b; Sheikin, 2017, 2002).

**FIGURE 4:**
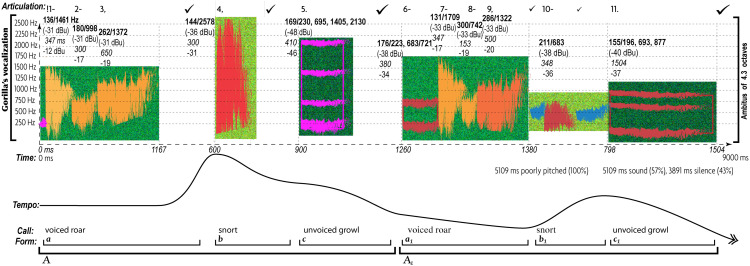
Characteristic patterns of AEs in expression of anger in gorilla’s calls (http://chirb.it/72g63y). Approaching primate’s vocalizations with the same multifactorial analytical method as human music reveals important differences in TO. The most noticeable is complete absence of harmonious sounds with clear FF and legato articulation. The share of silence doubles: 43% (versus 17% of Figure 3). The form is simpler—no motifs conjoin into a phrase. Calls (voiced roar, non-voiced growl, and snort) remain detached except for a few instances of joining snort and growl together. The same disconnectedness characterizes all temporal AEs. The onset of each of the calls exposes a sort of an irregular pulse. However, the rate of this pulse is more than twice slower than the angry human music (Figure 3) and its deviation from a regular pulse is nearly twice greater—exceeding even the slow and flexible “loving music” (Figure 2). In essence, it would be accurate to characterize these vocalizations as rhythmically irregular, ametric, and undifferentiated in pitch. None of the calls generate a clear pitch contour due to their very broad band (up to 4.2 octaves). The calls’ bandwidth was calculated by taking measurements of the frequency of that portion of the spectrum which stood out from the rest of the signal. Unlike music, gorilla’s call-motifs do not break the ambitus into registers but timbrally recolor the entire ambitus for each of the calls, thereby increasing their separation.

Unlike the expression of love, anger engages a wider ambitus, greater leaps, contrasting registers, harsh timbres, loudness, shorter and richer rhythms, reduced regularity and tonal stability, increased tempo fluctuations, staccato articulation, and thematic complexity (Figures 2, 3). However, gorillas express anger differently: “call-motifs” remain always isolated and slow-paced, featuring neither a clear melodic contour (due to its enormous bandwidth) nor rhythm (Figure 4).

If humans *consciously* manipulate numerous *learned* expressive parameters in music, animals *instinctively* “center” on a single biologically “*hard-wired*” parameter to reflect their emotional intensity. Human infants start their development at the same level where animal cubs start theirs, but quickly advance. Newborns employ just 2 vocalization types: negative and positive (Loewy, 1995). Cries of hunger, cold, distress come first as biological reflexes (Zeskind, 1985). However, the similarity of an infant’s supralaryngeal vocal tract to that of the primate cub’s does not stop the infants from trying to imitate his/her caretaker’s vocalizations (Lieberman, 1985)^36^. Infant cries start varying in temporal and frequency characteristics as the infant ages (Papoušek and Papoušek, 1995). Loudness, timbre, register, attack speed, FM range, and harmonicity are progressively mastered as markers of different cry-types (Golub and Corwin, 1985). An infant builds a repertory of melodic contours assigned to specific situations and used as building blocks to inform the caretaker about his/her state and to receive a desired treatment (Wermke and Mende, 2009). Such ongoing two-ended communication lies at the heart of musicality (Trevarthen, 2019).

Call/cry-repertory building appears to be universal in human development (Wermke et al., 2007), very likely paralleling the phylogenetic evolution of music (Foster, 1994). Similarities between the structure and function of human and non-human vocalizations were discovered in crying, motherese, and babbling (Snowdon, 2003). Fluent switching from one cry-type to another, corroborated by the caretaker’s response, prompts the cross-examination of the cries’ acoustic parameters. The intensity of temporal expression usually matches pitch expression (frequent leaps require faster tempo to convey excitement and emergency—otherwise the caretaker is not “convinced” to respond urgently enough). Together, the projection of feedback and memorization/cross-relation of cry-types establish the acoustic oppositions between AEs of common musical emotions.

What diverts music from AC is the radical change in communication framework. Animals communicate “face-to-face” in situations that demand immediate action, which selects signals effective in expressing rapidly changing motivational states, with clear gradations in their intensity (Morton, 1977). Such signaling prioritizes ease of detection, speed of interpretation, signal’s briefness, and a single salient gradient AE (Maynard-Smith, 1976). High redundancy and stereotypicity of selected signals often “fix” them (Simpson, 1997). This precludes combinability of AEs and calls, enabling “*dishonest*” calling.

Unlike animal calls, traditional indigenous music normally *never “lies”* (Nikolsky, 2016, Appendix III). A performer, as a rule, expresses emotions he actually feels—even when impersonating an epic protagonist or a spirit, the singer becomes temporarily “possessed” by them (Novik, 2004, 272). “Putting on an act” is a prerogative of post-Renaissance Western classical performance tradition, and even there the performance canon demands “method-acting” to convince the audience in the realism of musical emotions (Nikolsky, 2015a)^37^. A non-western traditional song usually appears “westernized” to the indigenous audience when “acted out” formally (Zemtsovsky, 1983). Folk “cover-songs” necessarily engage the performer’s “direct”—rather than “indirect” or “scripted” speech (Zemtsovsky, 1979)^38^.

Insincerity and falsehood in musical expression did not present a critical issue prior to the 1760s (Charlton, 2009). They both attracted public discourse as a systemic aberration peculiar to a specific class of music (rather than a “defective” sample) only after the entertainment industry became institutionalized (Dahlhaus, 1989, 314). Rise of mass production made “emotional faking” a norm for commercial popular music—explicitly codified in Irving Berlin’s composition standards (Suisman, 2009)^39^. So, music started as a decidedly “honest signal” (Levitin, 2009, 141–6) and only recently adopted “acting”—albeit, hardly enough to declare music fundamentally “dishonest^40^.”

Jointly, multi-dimensionality of music and emotional contagion make lying difficult. Music always integrates listeners and performers, and this togetherness promotes sincerity. The particulate structure of musical semiosis effectively reveals dishonesty: at least some of AEs’ insincere expressions are bound to contradict each other, prompting a resolving interpretation. But what in the cultural evolution could have spurred the inclination for aspect-matching?

## Domestication of Animals Sets the Need to Make Tonal Organization Semiotically Functional

**FIGURE 5:**
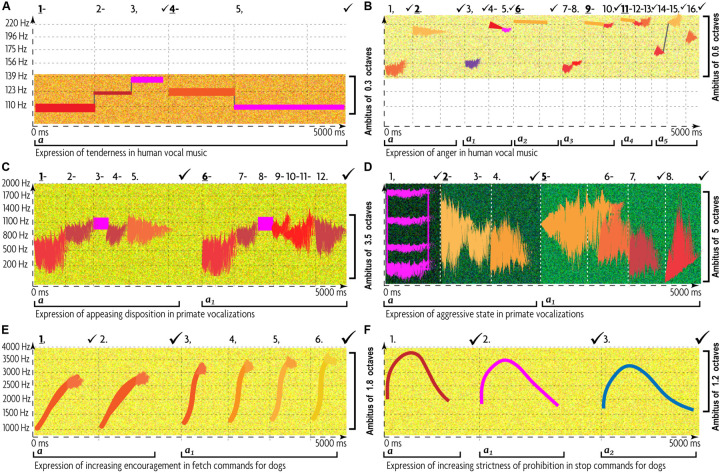
Hybridization of characteristic patterns of ACs and human music in encouraging and prohibiting commands by human trainers to their dogs (McConnell and Baylis, 1985; McConnell, 1990, 1991, 2002; Miklosi, 2015). **(A) Typical expression of tenderness in human music**. This diagram extracts the key features of Table 2 and Figure 2: very few pitch-classes with a low rate of change within a narrow ambitus, wave-like melodic contours filled by stepwise motion in the low register, slow tempo, with long tones and tendency to decelerate, and regular meter yet rhythmic diversity. Articulation is mostly legato, with occasional pauses. Dynamics is soft, stressing the anchor tones. **(B) Typical expression of anger in music** (according to Table 2 and Figure 3): many pitch-classes with high rate of change and wide ambitus, ascending contours, and leaping zigzagging motion in high register. The tempo is fast, with short tones, often accelerating, with irregular pulse, and strong rhythmic contrasts. Dynamics is mostly loud, and accents fall on metrically weak tones. **(C) Typical expression of appeasing disposition in primate vocalizations** (Table 3). Many pitch-levels have a high rate of change, following a gradually ascending melodic contour within a relatively narrow ambitus. Tempo is fast, with short tones and long groupings. These features strongly contrast **(A)**, whereas metric regularity, legato articulation, low registration, and soft dynamics resemble **(A)**. **(D) Typical expression of aggressive disposition in primate vocalizations** (Table 3 and Figure 4). There are relatively few pitch changes due to an extremely broad bandwidth, precluding frequent leaping. Long tones are embedded in fast motion with a descending contour in low register. These features oppose **(B)**, whereas meter, articulation, dynamics, and harmonicity resemble **(B)**. **(E) Typical expression of growing encouragement in fetch-whistles for dogs**. This expression combines a tender disposition of a human **(A)** with the appeasing disposition of a dog **(C)**. Therefore, fetch-command has to reconcile the contradictions between AEs’ expressions of **(A)** and **(C)**. To accomplish this, the ascending contour becomes steeper, each signal and the time interval between signals become shorter, the ambitus of each signal grows and reaches higher register, and the groupings grow in size (from 2 to 4). Temporal and pitch AEs are co-adjusted, merging traits from **(A)** and **(C)**. **(F) Typical expression of growing prohibition in stop-whistles for dogs**. This expression combines the display of human displeasure, like **(B)**, with the appeasing disposition of the dog **(C)**, while structurally and semantically opposing **(E)**. **(F)** subverts a single long tone to the contrasting gradual flections in pitch, where the descending portion receives the greatest significance. The increase in intensity of prohibition is signified by extending the time values and reducing the steepness of the descending curve—in contrast to **(E)**. Dynamics provides yet another axis of opposition: loud for **(E)** versus soft for **(F)**. Most importantly, the **(E,F)** opposition involves a compensatory interaction of the temporal, dynamic, and pitch patterns of AEs. Thus, whenever **(F)** is used in isolation, its softness, slowness, and ametricity might project the impression of passiveness—contrary to the categorical nature of a “stop” command. To avoid this, **(F)**’s melodic curve combines ascending and descending curves whose conflicting relation generates extra tension.

The need to command domestic animals underlaid the population explosion of both humans and livestock during the Neolithic Revolution. Animals benefited from human support, while humans benefited from animal produce. They both had to establish common patterns in their existing codes of vocal communication and adopt new patterns wherever the old patterns were deficient. Aspect-matching of pitch and rhythm was part of “bi-specific translation” of human commands (Figure 5). Rhythm reflects the “motion” pattern characteristic for a given “emotion” (Amaya et al., 1996), while pitch—the exertion/effort required by such motion—jointly defining a “sound gesture” (de Götzen, 2004). Perception of pitch and rhythm relies on the biological components mutual for mammals, thereby supporting heterospecific communication. There is fMRI evidence of shared emotional vocalization systems across species (Belin et al., 2008).

An account of pitch-rhythm interaction comes from dog-training. Long continuous low/descending pitch is universally used to stop a dog, whereas repetitions of short rhythmic high tones—to encourage it—which might comprise a mammalian generality (McConnell and Baylis, 1985). Dog trainers identify pitch contour, rhythm, repetition rate, and amplitude as AEs effective in dog’s commands.

Stop/fetch opposition reflects a multi-dimensional compensatory interaction of pitch, rhythm, and dynamics, mutual for both humans and canines. Some of the animal acoustic “universals” became appropriated into this bispecific communication, while others were overruled. Thus, across mammals, greater amplitude generally corresponds to a higher level of arousal (Briefer, 2012). However, it is only the fetch-command that follows this rule, whereas the stop-command, in contrary, adopts soft dynamics to subdue a dog (McConnell, 2002, 49–63). This overriding of the natural association between dominance and loudness highlights the fundamental difference between human and animal communications (Owren and Rendall, 2001):

• Human communication is “*receiver-centered*”—TO caters to information requirements of the listener;
• Animal communication is “*sender-centered*”—TO reflects the psycho-physiological state of the signaler, disregarding the listener.

Human-to-animal communication integrates both strategies:

• *Humans address animals*, treating them like humans, but *perfect the encoding* to secure the desired response. Thus, “doggerel” (Hirsh-Pasek and Treiman, 1982) constitutes dog-directed adaptation of human motherese (Mitchell R. W., 2001).

Pitch contour is a primary AE for most human cultures. Melody is the only aspect that differentiates between the basic musical emotions completely on its own (Table 2)^41^. In ACs, pitch does not provide such differentiation (Table 3). Pitch’s importance for music pushes human melodies *higher* in register. This is because the low frequencies appear softer (Oxenham, 2013)—making the low contours less salient than the high contours. The same applies to primate hearing and, possibly, other mammals (Stebbins and Moody, 2011). Domestic animals too should follow suit. This incentivizes humans to raise contours characteristic for basic emotions above 1 kHz, where pitch changes are more salient. The only exception is the affection/love signals. Intimacy requires close-distance communication where the softness of low-frequency poses no problems.

Social animals share affective signaling system with humans (Snowdon et al., 2015). This enables effective musical communication between humans and domestic animals—all of whom are “social” (Stricklin, 2001). SFTO in all likelihood evolved gradually, following the schemata of human-to-dog communication. The earliest archeological evidence of domesticated dogs dates back to 15 kya (Larson et al., 2012), but signs of domestication were found in a Gravettian site, at Předmostiì (Germonpré et al., 2012). The DNA analysis indicates that a dog-like 33 kya old fossil from Altai is closer to modern dogs than to wolves (Druzhkova et al., 2013). Dog domestication must have been slow, preceded by feeding dogs with leftovers in exchange that they would follow humans and alert them of approaching predators. Dogs are genetically adapted to digest starch, which constituted part of human diet (Axelsson et al., 2013). Similar adaptation occurred in dog’s communication system. *It adopted traits of human TO*. Compared to wolves, dogs use more vocal signals, especially bark-based—and barks feature co-modulation of two expressive aspects, amplitude and rhythm (Simpson, 1997). Alerting and territorial barking, both vary in intensity and rate depending on the distance of the dog from the conspecific or heterospecific intruder and the extent of the dog’s arousal. At near distances barks become louder and more rapid. Such signaling and the manner of its modification most likely evolved in response to human’s selective pressure on dogs to bark territorially at strangers (Simpson, 1997).

Human-to-dog communication most likely prototyped communication to later domesticates: cows, sheep, and goats. The surviving Nordic tradition of *kulning* provides the gist of the Neolithic pastoral music-making.

## The Scandinavian Tradition of Kulning as a Model of Neolithic Musical Semiosis

Animal husbandry in Scandinavia started ≈1800 BC and reached its “golden age” by 1200 BC. This is when owning larger stocks became prestigious while climate warming enabled outdoor animal maintenance almost year-long (Tesch, 1992). However, winter grazing was hard on bushes and trees, depleting local resources. This, along with subsequent climate cooling, brought about a new housing style, designed to shelter animals together with humans for winter—which characterized Scandinavian pastoralism (Armstrong Oma, 2013). Sharing the house with animals led to acceptance of animals as household members, equal to humans, and categorically as “clean”—even animal dung was used to make wattle and daub walls. Sharing is known to increase bonding. Human dependence on milk products, and animals’—on humans’ “room and board” promoted mutual trust and attraction (Armstrong Oma, 2010). From being “products,” animals turned into “producers” of dairy. This brought about *psychological “revolution”* in human-animal relationships, where music acquired the leading role.

Milking required concordance. An irritated animal or milkmaid reduced milk-yield, reducing human nutrition. Humans had to maintain mutual affection toward animals—evident in taboos on swearing/screaming at cattle, widespread across Eurasia (Plotnikova, 1999b). Music ritualized and fortified this union across different cultures (Shevtsov, 1988; Wallin, 1991; Alekseyev, 1995; Ivarsdotter, 1995, 2004; Novik, 1999; Dorina, 2004; Dissanayake, 2005; Kolltveit, 2008; Cheng, 2009; Yoon, 2018), especially evident in surviving traditions of milking songs (Nielsen, 1997; Pegg, 2001; Gioia, 2006b), animal lullabies (Kondratyeva, 1989; Kyrgys, 2002; Tchotchkina, 2003; Kan-ool, 2012), and spells (Kondratyeva, 1996; Kyrgys, 2002; Bordzhanova, 2007; Sodgerel, 2012, 2016; Tiukhteneva, 2017)—which all share the union of musicality and love/care that characterizes human motherese (Trevarthen, 2019).

Principal traits of such music can be extracted from the current practice of Scandinavian herder’s music-making. Its chief task is to control the behavior of the grazing livestock during the warm seasons at distant pastures (Ivarsdotter, 2004). The herder aims at influencing the animal’s emotional state over a range of distances, up to a few kilometers. Long-distance transmission requires a special vocal technique and musical instruments. The same musical signals convey different information to livestock and humans: commanding animals while informing animal-owners at the farmstead of their animal’s wellbeing. This dual communication has been faceted through a transhumance system known as *shieling* in England (Cheape, 1996), and *fäbod* in Scandinavia (Svensson, 2015)—emerging during the late Bronze Age in response to the scarcity of local winter fodder (Tesch, 1992). In Sweden, the shieling standard was set in Dalarna, and the alternative local traditions are considered its variations (Svensson, 2015). Traces of shieling are spotted across Europe, from the Hebrides to the Carpathians, becoming widespread by the Iron Age (Cheape, 1996). In Norway, the earliest fossil fields of lynchets show signs of cultivation during the late Bronze Age (Skrede, 2005), confirmed by palaeobotanic and archeological dating (Kvamme, 1988).

Shieling is characterized by seasonal migration to a summer station where herders spend their daytime supervising animals, preparing fodder for the coming winter, and produce dairy during evenings (Cabouret, 1984). Since milking, butter- and cheese-making traditionally constituted the women’s job, shieling and its music became female prerogatives in Scandinavia. There, milking could dishonor a man, and shieling was managed exclusively by young women (Svensson, 2015). In Ireland, shieling was a family business, whereas in Spain, France, and Switzerland dairy-work and herding were conducted by men.

The gender difference, undoubtfully, played a role in shaping the European pastoral musical traditions. Scandinavian, Icelandic, Alpine, Jurassic, Pyrenean, Apennine, Sardinian, Balkan, Turkish, and Caucasian mountains have sheltered singing styles that originated in the herding culture, and shared a peculiar singing technique based on a forceful high-laryngeal falsetto-like sound production (Wallin, 1991, 510). Wallin (pp. 511–23) summarizes the archeological, anthropometric, and genetic research to support the ethnographic findings of Carl-Allan Moberg (1971). Moberg outlines the core traits of the archaic *Fåbodväsendet* music: “head-voice” vocal technique, utilitarian function of long-distance signaling, and ideological roots in pagan magic.

The centerpiece of *Fåbodväsendet* tradition is its “maximal-distance” style—“*kula*”—that I distinguish from “kulning”—an umbrella-term for the entire *Fåbodväsendet*^42^. Local names for kulning (e.g., *lockrop*) imply the alluring of animals by magic properties of sound to suggest certain behavior to the herd, avert evil trolls and predator-animals—following shamanic tradition of maiden singing (Mitchell R. W., 2001). In Swedish mythology, forest spirits possessed their own cattle, and herdswomen (*kulerska*) learned kulning from *skogsrå*, “sirens of the woods” (Johnson, 1990). Suggestive power of kulning was deemed so high that women lived in *fåbods* alone without any weapons. Folk beliefs attributed this power to beauty. Indeed, well-ornamented high “warbling” register of distant female voice made men and women pause their work and enjoy the sounds (Ivarsdotter, 1986). For humans, kula clearly presented an aesthetic object despite bearing utilitarian status of “non-music” (Frödin, 1929)^43^. For animals, kula constituted a “safety call.” Both attitudes focus on *positive* rather than negative emotions—not only to keep the cattle under human control, preventing panic, but also to boost the *kulerska’s* confidence and alertness (Wallin, 1991, 420)^44^. SFTO must have emerged as a set of sonic attributes, perception of which was directly “wired” to reward circuits in brains of humans and domestic animals.

Wallin (1991, 420) rightfully stresses that matriarchy influenced early pastoralism: “the maternal instinct and care” instilled the social holding of attachment to stabilize and reinforce the animal-human affiliation. Distinctively female, *Fåbod* tradition must have prehistoric roots (Johnson, 1990). Motherese undoubtedly prototyped a close-range kulning. Animal-directed vocalizations acoustically and functionally resemble lullabies by commanding calmness/happiness—not just in Sweden (Wallin, 1991, 392) but also on the other side of Eurasia, in Altai (Kondratyeva, 1996). Common traits include prolonged singing, formulaic regularity, vocables, smooth contours, motherese-talking, and caressing (Tiukhteneva, 2017). In animistic societies, both infant-lulling (Kondratyeva, 1989; Farber, 1990; Tchotchkina, 2003; Gioia, 2006a; Milne, 2017; Garroway, 2019) and domestication rites for newborn cattle (Aksyonov, 1964; Johnson, 1990; Kondratyeva, 1996; Plotnikova, 1999b; Kan-ool, 2012; Tiukhteneva, 2017) are associated with magic, achievable by female “charms.”

Similar to lullabies are milking songs (Nielsen, 1997)—used across Eurasia, from Scotland to Mongolia (Gioia, 2006b, 71). Remarkably, when milking, Mongolian herdsmen switch to motherese-like “musical talk,” based on animal onomatopoeia (Yoon, 2018). Known cases of male pastoral calling engage falsetto to imitate the female model (Uttman, 2002). Similarly, in surviving pastoral traditions of Altai, lulling is reserved for women, and require throat-singing if sung by men (Tiukhteneva, 2017). Pastoral spells in Altaic tradition constitute female prerogative, but are occasionally performed by men (Kondratyeva and Kopytov, 2017), engaging throat-singing (Kyrgys, 2002, 64). Like falsetto, throat-singing emphasizes harmonics that make melodies appear registrally higher—closer to the female range—and, like female kula, resembling pure tones.

The same applies to whistling signals, used across Eurasia by herdsmen to stimulate and/or safe-guard animals (Levin and Suzukei, 2006, 134–40). Just like kulning, in pastoral societies whistling is associated with sorcery (Plotnikova, 1999a) and is thoroughly regulated by taboos (Dzenzelevskii, 1984). Acoustically, whistling comes closest to “kula” in distance-range, loudness, and tonal quality (Eklund and Mcallister, 2015). To command their animals, Altaic herdsmen produce whistles audible over 4–5 km, and throat-singing—3 km (Pegg, 2001, 236). Curiously, female “head voice,” required by kula, is called “whistle register” (Sundberg, 1987, 50). And *xöömii* (throat-singing) is considered a form of whistling in Mongolia (Pegg, 1992).

**FIGURE 6:**
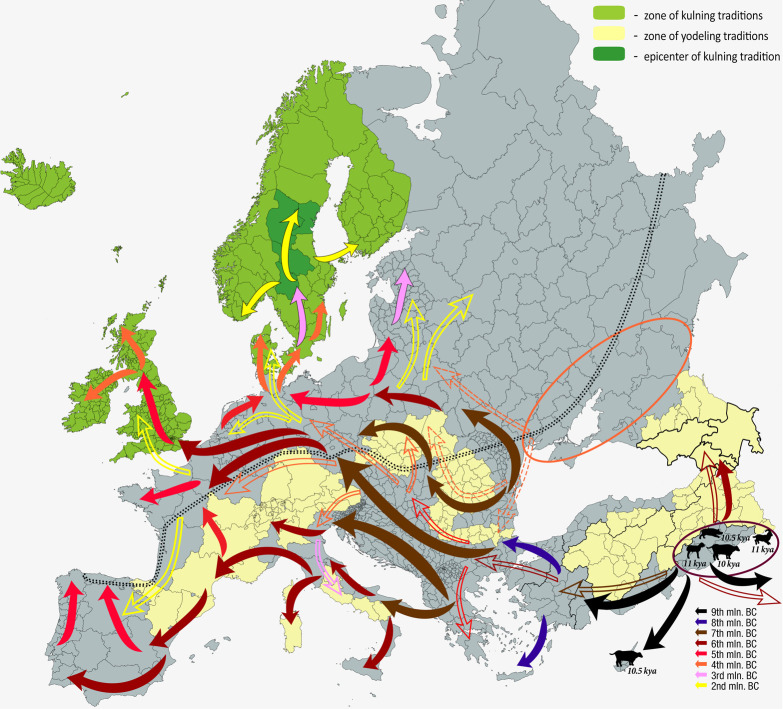
The earliest spread of pastoralism across Western Eurasia. This figure shows the approximate timeline and the geographic correspondences between locations of herding falsetto-like vocalization, the oldest areas of cattle-breeding and distribution of Indo-European languages. Light green color marks the territory of shieling pastoralism, dark green—the “core” Fåbod regions, and crème—the area where yodel-like vocalizations survived within pastoral cultures (Moberg, 1955, 1971; Baumann, 1976; Leuthold, 1981; Ivarsdotter, 1986; Wallin, 1991; Mitchell S. A., 2001; Uttman, 2002; Plantenga, 2004). The origin of the latter can be dated by the timeline of the spread of domesticates over Europe, which is well studied. Animal icons show the approximate place and time of origin of domesticated cow, goat, sheep, and pig, based on available archeological data (Zeder, 2008; Driscoll et al., 2009; Peters et al., 2017). Color-filled thick arrows show the timeline and main routs of dissemination of domesticated cattle during the Neolithic and early Bronze Age according to the archeological and genetic data (Caramelli, 2006; Lõugas et al., 2007; Zeder, 2008; Rowley-Conwy, 2011, 2013; Tresset and Vigne, 2011; Bläuer and Kantanen, 2013; Marciniak, 2013; Saña, 2013; Schulting, 2013; Sjögren and Price, 2013; Berthon, 2014; Cramp et al., 2014; Felius et al., 2014; Sørensen and Karg, 2014). The darker the arrow’s color, the older the date. The double-dotted black line approximates the border between the Northern and Southern European bovine genetic funds. Colored ovals and outlined arrows indicate the hypothetical origin and the spread of Indo-European languages according to the computational methods, based on Bayesian logic and phylogenetic analysis algorithms (Diamond and Bellwood, 2003; Gray and Atkinson, 2003; Atkinson et al., 2005; Atkinson and Gray, 2006; Bellwood, 2008; Gray et al., 2011; Anthony and Ringe, 2015; Chang et al., 2015; Heggarty, 2015). The brown oval marks the area of genesis of Proto-Indo-European language according to the “Anatolian hypothesis” (Renfrew, 1987), whereas the orange oval—to the earlier “steppe hypothesis” (Gimbutas, 1993; Anthony, 1995). The dashed outlined arrows show the earliest stages of dissemination of the Indo-European languages from the Yamnaya epicenter. Both hypotheses generally agree in defining the later stages (Gray et al., 2011)—represented by solid outlined arrows.

Wallin (1991, 523) sees shieling music as part of the prehistoric expansion of a novel herding culture northwest of Anatolia/Balkan/Caucasus toward Iceland, with its base in Jamtland (Figure 6). Jamtland’s “forest barrow” marked the end of tundra after the glaciers’ retreat, attracting hunters and supporting a mixed pastoral economy that survived at the coldest outskirt of Europe practically unchanged until the late Middle Ages. Geographic and chronological distribution of cattle-herding across Europe, quite well-studied, provides timing references for Wallin’s model. The outcome of this geomusicological^45^ correlation is presented in Figure 6.

Domesticated cattle spread East-to-West along the Mediterranean coastline, encapsulating most of “yodeling” territories ≈6000 BC. The South-to-North expansion took much longer—Central Sweden became pastoralized in the 2nd millennium BC. Dissemination of cattle and Indo-European languages went hand by hand. The Indo-European language family covers most of Europe—except for Finno-Ugric languages of Fennoscandia and Russia. Another notable exception is Turkey whose Indo-European languages (Hittite, Luwian, Palaic, Lydian) died out during Antiquity. Formation of each new Indo-European language seems to have followed the adoption of husbandry. The yodeling areas correspond to the earlier stages in expansion of the Indo-European languages, conserved by the mountain systems: Taurus, Pontic, and Armenian Highland in Turkey, the neighboring Caucasus, Balkan, and more remote Carpathian, Alps, Jura, Apennine, Sardinian, Corsican, and Pyrenean. The dissemination routes either curve around the mountains or cross them by riverbeds. The oldest routs ran by the Mediterranean coastline along the 40N latitude, supporting the conclusion of Diamond and Bellwood (2003) that the domesticates and languages spread faster to East-West than to South-North. This explains the divergence of pastoral music tradition into two types: Southern yodeling versus Nordic kulning and kulning-likes^46^, distinguished by different bovine genomes. Studies of Y-chromosomal variation have identified two primary taurine haplogroups in Europe, split in two homogenous regions alongside cultural, historic, religious, and linguistic boundaries between the pied or red cows of the Nordic and Baltic/Slavic lands, on the one hand, and the spotted yellow or brown breeds of Switzerland and southern territories, on the other hand (Edwards et al., 2011).

Kulning and yodel form respectively Northern and Southern “dialects” of a cattle-directed “language”—a satellite of the proto-Indo-European. The main role in the Indo-European “domestication package” belonged to cattle—the largest meat- and milk-source of all domesticates. The emergence of cattle-related mythology reflects the importance of cattle and explains the sudden proliferation of cattle burials across Northern Europe ≈3000 BC (Sjögren and Price, 2013)^47^. Symbolic elevation of cattle could characterize the entire Neolithic “revolution” in Eurasia, more noticeable in Scandinavia, where ox symbolism replaced red-deer symbolism after ox overtook deer as the most important food source (Tilley, 1996, 183–4). If wild deer opposed the human sphere as a *utilitarian* object of desire, domesticated ox was included into the human sphere as the *emotional* object of desire. And music is indispensable in supporting emotionality.

Divinization of music (Franklin, 2006) and ox (Campbell, 2017), so prominent in Indo-European tradition, could have a single origin in Indo-Iranian lands—bound to the concept of non-violence (Tull, 1996). Cattle sacrifice is depicted in prehistoric Sujanpura petroglyphs (Brooks and Wakankar, 1976). The ritual use of burnt cow dung is still common in Hinduism, traceable to the 3000 BC Ashmounds (Boivin, 2004). The Shiva-bull affiliation is evident in the Bronze Age Harappan “Proto-Shiva” (Hiltebeitel, 2011). Harappan symbolism clearly elevates the cattle over other domesticates, evident in the buffalo figurine amulets and seals that are likely to assimilate the west-bound Indo-Iranian cult of Mother Goddess, eventually forming the “Sacred Cow” concept (Lodrick, 2005). This corresponds to veal and cow-milk becoming primary foods during Rigvedic and Vedic times—there were people at that time who lived on milk alone (Prakash, 1961, 12). Milk products were used in rituals and offerings to gods, certainly accompanied by music, promoting the transformation of cow into the symbol of femininity and fecundity in Vedic literature (Brown, 1964). Consecration of cow gave it purity: even its urine and dung were used for healing and cleansing (Korom, 2000).

The cultural context of kulning and the tradition of home-sharing with cattle strongly resembles the Vedic cultural blend of non-violent femininity, cow-worship, and magic. It is not accidental that kula finds a nearly perfect match in Tibetan traditional pastoral songs with long rhythmically free undulating phrases, extremely tense timbre of high quasi-falsetto voice, generous ornamentation, and an ongoing variation (Stuart, 2008, XXIV). This is the most ancient of the three major forms of Tibetan music, peculiar to a nomadic pastoral culture, and originating from cattle calls (Crossley-Holland, 1967). Like kulning, it incorporates parlando and recitative for close-distance vocalization to animals, and also includes milking songs (Plantenga, 2004, 113).

Introduction of milk revolutionized the Neolithic lifestyle, supporting the psychological revolution in human-animal relations and bi-specific musical communication—especially in Northern Europe, where milk quickly replaced fish as the main food—manifested by the widespread adoption of milk-storing pottery (Cramp et al., 2014). The archeological evidence agrees with the genetic evidence of the time of emergence of lactase persistence^48^. Lactase persistence reflects the adaptation to diet (Hancock et al., 2010)—without which adults have lactose intolerance and nutritional loss (Campbell et al., 2005). Ill effects of malnutrition coexisted with milk-bound diseases during the adoption of the milk-based diet. Mycobacterium tuberculosis existed 40,000 years ago, but became pathological for humans only from 6200–5500 BC onward (Hershkovitz et al., 2015) - by the time when the spread of husbandry reached Central Europe. Seemingly “the same” milk could either kill or nurture life—which must have promoted new supernatural beliefs and rituals to “exorcize” milk-production in replacement of the earlier hunter/gatherer rituals. Music, so common for religious applications, most certainly supported this reform.

For Europe, geographic distribution of Indo-European languages^49^ (Heggarty, 2015) goes hand in hand with the distribution of taurine mtDNA that descends from the Fertile Crescent (Caramelli, 2006). And subdivision of the bovine European genetic pool into Northern/Southern genotypes (Edwards et al., 2011) matches the distribution pattern of lactase persistence: 40% of adults in Greece versus 90% in Scandinavia/England (Curry, 2013). Those populations that consumed more dairy have higher occurrence of lactase persistence (Bersaglieri et al., 2004). Evidently, milk dependence was more than twice higher in the North. The Indo-European expansion occurred through the farmers’ immigration and interaction with local foragers rather than by technological import alone (Rowley-Conwy, 2011). Greater lactase persistence in the North reflects the dairy’s effectiveness in providing nutrients, the convenience of its storage in cold climate, the insurance against bad harvests (Gerbault et al., 2013), and health benefits of increased vitamin D consumption in low-sunlight conditions (Flatz and Rotthauwe, 1973).

Kulning emerged to nourish the symbiotic co-dependence of humans and cattle in harsh Nordic conditions that demanded stronger bonding than those of more diverse pastoral economies of Southern yodel territories, therefore employing a **female pastoral model**.

The biggest contender for the Indo-European language family in Northern Europe—the Uralic family (Diamond and Bellwood, 2003)—relates to another domesticate: the reindeer. Reindeer hunting was essential for colonization of Eurasian Arctic/Subarctic (Gordon, 2003). However, reindeer domestication still remains in its early phase (Reimers and Colman, 2009). The distinction between reindeer-hunting and reindeer-herding remains vague—even reindeer owners often do not know if a particular reindeer is “wild” or “domestic” (Ventsel, 2006)^50^. Leading fences and corrals have been used for hunting wild reindeers and only recently have they become “domestic” accessories (Aronsson, 1991). Reindeer pastoralism emerged gradually from taming individual reindeers for transportation and decoy-hunting—compensating for the depletion of wild reindeer population (Vorren, 1973) that occurred during the 13–16th centuries (Hansen and Olsen, 2014, 175)^51^. Reindeer domestication must have started in parallel with cattle domestication in Norway/Sweden but lingered into the Middle Ages—absorbing cultural traits of human-to-cattle communication.

The principal psychological trait of kulning is the “humanization” and child-like patronizing of cattle. Similar attitude characterizes reindeer pastoralism: animal is treated like a family member whose life is valued and its attitudes are respected (Ingold, 1986). Kulning, yodel, and reindeer-communication should all be regarded as various “**languages of domestication**,” generated by borrowing “acoustic traps and snares”—i.e., onomatopoeic decoy calls—from hunters and syntactically reorganizing them into “animal-directed” words to control the herd, its leader, and the individual animals (Alekseyev, 1995).

Kulning and yodel are *Indo-European* musical “*cow-languages*,” later adapted for goats/sheep as they became personalized like cows^52^, whereas reindeer-vocalizations make a *Finno-Ugric* “*reindeer-language*.”

Kulning’s SFTO was forged by long-distance delivery of the desired subharmonic structure. Kula is characterized by dynamic maximization (80–100 dB SPL at 50 cm)^53^ while fixing 4 formants at FF, 1700, 3,000 and 4,000 Hz throughout all frequency changes, restraining vibrato, and raising the larynx above the resting position (Johnson et al., 1982). Elevating laryngeal position up to 4 cm increases the sub-glottal pressure tenfold as compared to talking (Ivarsdotter, 1986). Somehow, this causes no distortions, and kula’s “harmonic signature” remains virtually unchanged at close- and mid-distances (1–11 m)—contrasting the “classic” falsetto (Eklund and Mcallister, 2015). Harmonic conservation is still observable at 22 m in kulning, albeit varying between different performers (Eklund et al., 2019). Evidently, kula is designed to transmit *kulerska*’s harmonic and melodic “signatures” to the herd at distances common in herding (Rosenberg, 2014).

Long-distance spectral optimization is known in intergroup communication of some primates (Waser and Waser, 1977). However, optimization to preserve subharmonic structures is unique to kula.

Kula’s sounds are supposed to stand out in the environmental soundstage by featuring unnaturally hyper-periodic noise-free spectrum. Kula’s harmonicity aligns with “**pleasantness**”—following the cow-bell paradigm. Animal-bells were used in Scandinavia at least from 1–4th centuries (possibly, from the beginning of the Bronze Age) to repel evil spirits, mark a human-controlled territory, and decorate the herd’s leading animal (Kolltveit, 2008). For cattle, the bell signified human control, herd-leader’s authority, and a safety signal. Humans associated bells with nature, peacefulness, goodness, and protection, employing bells to “borrow” the land from the forest spirits (Emsheimer, 1991, 43). Therefore, overall harmonicity signifies strongly positive values—in line with kulning’s perceived beauty and safety/care. Across the animal world, too, harmonicity (pure-tonedness) and inharmonicity are meaningful along the friendliness/fear opposition (Morton, 1977).

Long-distance transmission requires high intensity and register. For 1 km, the most effective transmission occurs at ≈2 kHz (= C7) (Graf, 1980; Gray and Atkinson, 2003)—the range of a piccolo flute. Perhaps, whistling prototyped kula. Whistles are common in communication with dogs and the herd. And whistles exceed calling and yodeling in long-distance intelligibility: correct identification of whistles at 170 m distance is 95% versus 58% for yodeling and 70% for calling (Titze et al., 2018). *Bi-factorial* changes of rhythm/pitch-contour in whistling signals would pave the road for *tri-factorial* changes of rhythm/pitch/phrase-length in kula.

**FIGURE 7:**
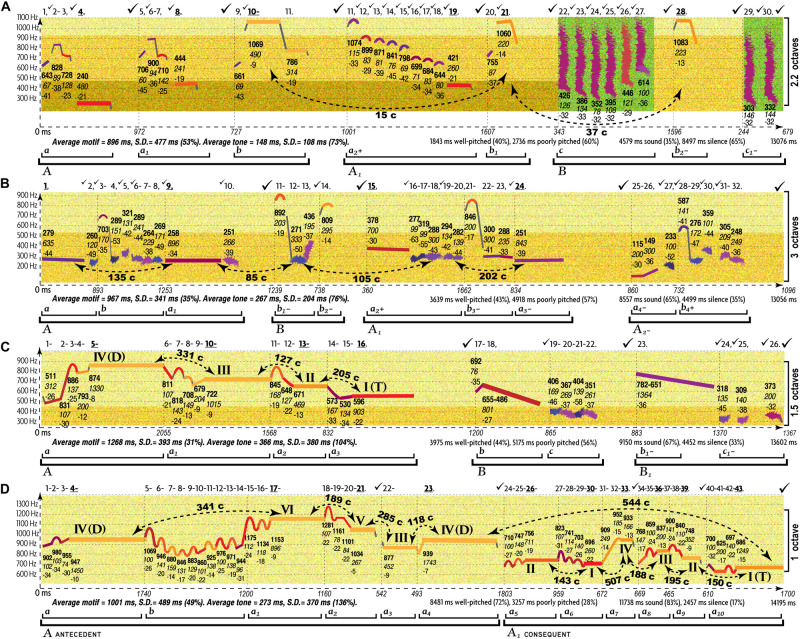
Patterns of TO in four main types of vocalization in the vocal tradition of kulning. Since kulning is essentially ametric and averbal (except for the closest range recitative), its analytic charts omit lyrics. Unlike the previous figures, the vertical dash lines indicate the onset of motifs. The colored arc-line symbol represents an ornamental melismatic shake. **(A) Stimulative medium-distance kulning: parlando (a), exclamation (b), and onomatopoeia (c) motifs** (http://chirb.it/ntIxfM). This style is designed to compel the entire herd to move in the desired direction and, most probably, sets a model of interaction with animals for the other three styles. The three motifs achieve stimulation, each in a different way, contrasting one another in register, harmonicity, rhythm, and articulation. Motif “a” alerts by its staccato zigzag leaping between two registers. Motif “b” combines stimulation (staccato leap up to the “shrieking” register) with relaxation (legato leap down to the long tone). The “shrieking” peak-tones maintain the same pitch level (melodic regularity)—reflected by the dotted double-arrows (numbers indicate the frequency discrepancy in cents). Motif “c” teases the cattle by imitating dog’s barking. The stimulative specialization of **(A)** is manifested in its prevalence of staccato, loud dynamics, three registers within a wide ambitus, exuberance of leaps, and briefness of motifs and tones. Noteworthy, the motifs “a_2_” and “c” resemble the “fetch-command” archetype (Figure 5E). **(B) Stimulative close-distance kulning: recitative (a) and motherese (b) motifs** (http://chirb.it/8K3Lqg). **(B)**, like **(A)**, is stimulative but dynamically gentler due to closer distance (≈9 dB softer). This allows for diverse motherese-like prosodic exaggerations in motivating individual animals. Motif “a” expresses love/care by greatly prolonging the “recitative tone,” sustaining its pitch and harmonicity. Motif “b” stimulates animals by briefly stressing the upper “head-voice” register with a shake-like embellishment, then sliding it all the way down to the low talking voice. Compared to **(A)**, **(B)** is smoother: fewer registers, less staccato, and longer motifs and tones. **(B)** tends to support a monotone (a predecessor of tonicity), most noticeable at phrasal ends. **(C) Inhibitive longer-distance kulning: simple kula (a), exclamation (b), and parlando (c) motifs** (http://chirb.it/n6f0sv). This style functionally opposes **(B)** by commanding the herd to stop grazing and to go home, implying that it is no longer safe to stay out. The chief function of “a” (kula) is to instill confidence in the herder’s control over the animals. “Kula” typically consists of a chain of motifs stitched together to form a characteristic shape of steep ascension to the crest point and thereafter a gradual fall-off. However, motifs might differ according to their phrasal functions: initiation, climax, decay, and cadence. The resulting kula receives a basic modal TO: anchor tones constitute “degrees” of the mode, forming a fifth between the marginal degrees and dividing it in wider upper and narrower lower parts. The Roman numerals indicate degrees (I = stable is marked as T = “tonic”). The “b” motif presents “exclamation”: a gradual sliding down (≈4th), softer than in **(A)**, and shaped like the “stop-command” (Figure 5E). Similarly shaped is the parlando “c” motif, much smoother than **(B)** due to its prevailing legato, freer rhythm, more homogenous registers, and longer motifs and tones. **(D) Tropotrophic maximal-distance kulning: exclusive use of complex kula sentences** (http://chirb.it/gpyC7t). Delivering signals over a kilometer requires taking multiple short caesuras throughout the span of the kula’s descending formula, which distinguishes **(D)** from **(C)** by making kula complex. Motifs make up phrases, and phrases—sentences, all of which create modal complexity: anchor-tones form intervallic relations that define degrees within a mode (usually, 5–7 degrees). Upper degrees open kula, forming an antecedent cadence (marked by letter “D”—“dominant” function). Lower degrees end kula with the consequent cadence (marked by “T”—“tonic”), providing resolution. Compared to **(C)**, sentences in **(D)** are longer, rhythmically freer, more homogenous (by maintaining legato, a single register, the narrowest of ambitus for all kulning styles, and no leaps). Relaxation, secured by modal resolution, is supported by beautification: exclusive use of legato in smoothly shaped phrases and exquisite ornamentation (shakes, trills). **(D)** differs from **(C)** by sacrificing dynamic shaping on a phrasal level and, instead, reproduces the same dynamic contour on a motivic level—the final long tone is almost always the loudest in a motif (i.e., stable). Increased homogeneity and melodic consonance (i.e., absence of leaps) are called to motivate the herd not to depart any further beyond the range of hearing kula.

Long-distance communication eliminates mimics and gestures from semiosis, making it rely exclusively on acoustic attributes and demanding long-term memory (Wallin, 1991, 390). Exclusion of visual cues promotes the prolongation of a musical expression to facilitate its recognition and memorization. Therefore, phrase length reflects the distance: longer distances require longer phrases (p. 391). Changes in distance generate musical syntax (Figure 7).

*Close distance* promotes short phrases of multi-registral motherese-like recitative where only the “reciting tones” are pitched, and exaggerated leaps employ legato and portamento (Figure 7B). Pitches have tendency to monotony in low register at phrasal ends, which generates tonicity. Vocalizations are mostly stimulating and diverse in their referential/propositional content.

*Middle distance* makes motherese inaudible, instead requiring a different approach. Vocalizations become euphonized: engaging “parlando” rather than recitative^54^, “smoothening” the leaps, increasing the share of pitched tones, and stressing rhythmic patterning and ordering. The calming effect of these adjustments, inappropriate for stimulating applications that are mostly common for mid-distance communication, is compensated by intensifying dynamics, structural contrasts, and staccato articulation (Figure 7A). Notwithstanding diversification, the highest-register “peak-tones” at motivic beginnings are often monotonous, prototyping the musical “leading-tone” by requiring some sort of continuation (as in a melodic resolution).

*Longer distance* further increases the share of musicality and pleasantness in herding vocalizations. They prioritize audition over visualization by engaging “call-phrases,” made of exclamatory imperatives and summoning, free from referential/propositional context (Wallin, 1991, 417). Verbalized vocalization is replaced by a wordless kula (p. 410). Simple phrase-sentences consist of motif chains akin to incipits, climaxes, and cadences of Gregorian tunes (Helmer, 1975). Each phrase is distinguished by a wavelike melodic-dynamic “envelop” with an abrupt quick rise and a gradual prolonged fall. Kula pushes vocalizations higher, squeezing their ambitus, homogenizing timbres and legato articulation, while loosening the rhythm (Figure 7C). This triggers the modal genesis: kula’s anchor-tones turn into degrees, with more-or-less sustained pitch values. The lowest degree becomes “tonic,” in contrast to the unstable upper degrees, thereby forming tetrachord-based modes.

**FIGURE 8:**
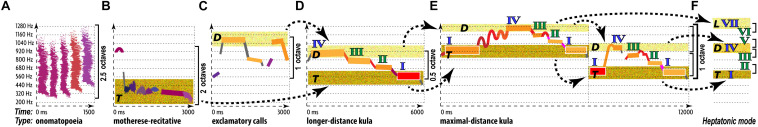
Genesis of SFTO in the vocal tradition of kulning. The set of six panels shows five excerpts representing five different vocalization types from Figure 7. They are placed on the same frequency grid to demonstrate how the registral position of phrasal tones evolves into a frequency range used to define a degree in a musical mode. Thin dashed vertical line indicates the phrasal ends. Thick curved dashed arrows show the genesis of “tonic” (principal stable) and “dominant” (principal unstable) degrees, eventually shaping a heptatonic mode. **(A) Mid-distance onomatopoeia (barking)**. This is the closest to ACs. A phrase repeats the same wideband aperiodic signal whose most intense part of the spectrum spreads over ≈2.5 octaves. **(B) Close distance motherese/recitative**. Low-register tones in such phrases tend to fall within the same narrow range of 250–290 Hz (257 c), marked by the darker grainy filling. Frequently repeated voiced vowels effectively refine the tuning of the “recitative tone” that adopts a tonic function (“T”) established by the common terminations of phrases. **(C) Mid-distance exclamatory calls**. High-register “shrieking” tones in such phrases are squeezed in a twice narrower range, marked by a lighter filling. These shrieking tones complement low tones in **(B)** in providing reference for pitch changes. Such tones prototype the “dominant” melodic function (“D”). Tones that fall in this register become imperfect “anchors” subdued to “tonic”—requiring a descending melodic “resolution” after them. **(D) Longer-distance kula**. Registral ranges of both “tonic” and “dominant” are further compressed into “degrees” of a simple 4-degree musical mode. Colored Roman numerals use blue for anchored tones, and green—for supporting tones (passing or auxiliary). Tonic function (stability) is shaped by the lowest degree terminating a phrase, whereas dominant function (instability)—by the highest degree initiating a phrase. This transformation is fueled by frequent stitching of **(B)**, **(C)**, and **(D)** phrases within the same musicking session as the distance changes. **(E) Longest-distance kula**. This type doubles the TO structure of the shorter distance kula—indicated by two thin vertical brackets encapsulated by one thick bracket. A similar tetrachordal structure is reproduced above the base-tetrachord. Both tetrachords are conjoined: the lowest stable degree (“T”) of an upper tetrachord becomes the highest unstable degree (“D”) of the lower tetrachord as kula descends from its opening phrase toward lower phrases, terminated by the lowest permanent “tonic.” Repeated use of such complex structure (common at distances over 1 km) is likely to turn it into a modal framework for the entire kulning, encompassing all its phrasal types. **(F) Heptatonic mode in complex kulas**. Frequent modulations between the conjoined tetrachords integrate both tetrachords into a single complex 2-tetrachordal mode with three axial degrees: the lowest *I*—a permanent tonic (“T”), the middle *IV*—an alternative temporary anchor that requires resolution (“D”), and the highest *VII*—a permanent unstable anchor, used to initiate sentences and/or build a climax—i.e., the “leading tone” (“L”) that always leads to more stable anchors (perfect and/or imperfect). These axial degrees enclose supplementary degrees, each of which is bound to the closest anchor, forming pairs.

*Maximum-range* communication complicates kula by introducing hierarchic structuring (motifs-phrases-sentences) and by engaging the contrasting phrasal functions (initiation/climax/interruption/termination). The stimulating effect of the increased syntactic contrasts, undesirable for maximum-range communication that focuses on keeping the animals calm, is compensated by greater melodic homogeneity: maximizing legato, sentence-length, and dynamics, while minimizing melodic-intervallic, rhythmic, and registral diversity (Figure 7D). Longer span necessitates inter-phrasal caesuras, marking multiple phrases within long sentences, joined by stereotypical declining inter-phrasal melodic and dynamic “envelops.” Melody relies on pentachordal skeleton, divided in upper major and lower minor 3rds, often supported with quartal/quintal infrafix (Johnson, 1979). Kula breaks in a series of antecedent-consequent sentences that engage different pentachord/tetrachord(s)—usually conjunct. This produces heptatonic modes (Figure 8).

The ongoing unveiling of musical structures makes kulning particulate by stacking up certain phrasal types while avoiding certain other combinations. This establishes syntactic rules and implicit music theory of TO for herders and herds. Herders perceive kulning as improvised “musical work in progress” (akin jazz improvisation) that elaborates a specific “theme” selected by the *kulerska* (Rosenberg, 2014). Herded animals probably perceive kulning as a series of programmed Pavlovian-conditioned routines. In both cases, compositionality promotes particulate semiosis: the meaning of a streak of phrases consists of the sum of the meanings of each of the constituent phrases. In effect, kulning tells a “continuing story” of the day, going through an elaboration of a musical theme (Rosenberg, 2014).

The herd’s daily movement generates SFTO by stitching/restitching phrases of 5 syntactic-semantic types (Table 6):

**TABLE 6:** Acoustic traits of main motif types and their semantic values in kulning.

**Acoustic domains**	**Aspects of music**	**Kula singing phrases and sentences**	**Exclamation calls**	**Onomatopoeic imitations**	**Parlando singing and calling**	**Motherese recitative**
1. Frequency	1. Melodic pitch (consecutive)	Prevalence of long descending stepwise motion; narrow ambitus of a 5th breaks in two portions: upper major. 3rd and lower minor. 3rd (with possible infrafix). Well-defined pitches, many melismas. The strongest melodic coherence. “Safety call” function –calming yet keeping alert, under control. Subject to aesthetic evaluation.	Prevalence of a zigzag pitch contour in a brief group of tones with a very short start, poorly defined pitches, medium long leaps, large ambitus of 0.5–1.6 octave. Optional but frequently employed shakes and melismas distinguish the stimulation use from inhibition, both engaged exclusively in the phatic function (no aesthetic value).	Short repetitions of the same imitation of an animal call: poorly pitched, very broad bandwidth, no melismas, melodic coherence is absent, stimulating function (to make the herd move in the necessary direction). Most likely, this is a derivative of a “fetch whistle” command. Utilitarian application (no aesthetic value).	Prevalence of a zigzag shape, like exclamation, but longer and stressing a huge descending leap (up to 1.6 octave), greater than an ascending leap (<7th). Both leaps relax toward the motif’s end. They stimulate or inhibit, based on the extent of such relaxation. Utilitarian application with aesthetic value.	Prevalence of drastic contrasts in pitch between flat monotonous pitched talking and extreme zigzag leaping (about 1.7 octave) that is usually embellished with melismas. Stimulating and motivating functions, supported with gestures and mimics. Only a few pitches are clear. Mostly utilitarian application.
	2. Harmony	n/a	n/a	n/a	n/a	n/a
	3. Form (complexity)	Greatest complexity	Simplicity	Greatest simplicity	Low complexity	Medium complexity
2. Time	4. Tempo	Slow, frequent ritenuto toward the phrasal end.	Moderate and/or slow tempos, possible ritenuto.	Moderate, possible accelerando.	Moderate or slow, frequent rubato.	Moderate and lively, with moderate rubato.
	5. Rhythm	Sharp contrasts of melismatic and anchor tones, the greatest rhythmic diversity, no increments.	Contrast of short-long pattern in grouping and totally arrhythmic “breath-groups.”	Prevalence of the same relatively short rhythm, grouped by pauses, clear increments.	Contrast of short upper versus long lower tones, and of groups of equal versus patterned rhythm.	Contrast of long initial versus short last tones, and of free “verbal” versus patterned “musical” rhythm.
	6. Meter	Most irregular, often totally “ametric,” a dragging feel.	Can contain regular fragments, usually iambic.	Mostly regular (spondaic).	Usually irregular (loose, free).	Irregular, as if always changing iambic-trochaic.
	7. Articulation	Absolute dominance of legato. Frequent and long caesuras between phrases that usually end on long tenuto tone and a descending glide.	Contrasted groups of staccato on ascending leaps and legato on descending steps. Frequent ending on a tenuto glide.	Prevalent non-legato provides ease and clarity of recognition for each of the imitations.	Contrasted groups of staccato for ascending leaps and legato for steps as well as descending leaps. Sometimes tenuto endings.	Syllables within a word usually are legato, while words or vocables are separated by pauses.
3. Amplitude	8. Dynamics	The loudest type. Mid-distance kula phrases are shaped diminuendo, while each long-distance kula sentence is shaped wavelike.	Rather intense dynamics with contrasts between the wavelike shape and the opposition of loud high versus soft low tones.	Only minor dynamic changes within mostly loud levels, copying the typical dynamic envelop of a typical animal call.	Moderate contrast of softer high tones and louder lower tones, in overall soft dynamics.	The softest type, yet with sudden accents, falling on a single syllable in those words that are marked by a zigzag leap.
4. Timbre	9. Register	Single, very high register—the longer the distance, the brighter the tonal quality (piercing or shrilling). Fixed high larynx position, constant brightness.	Multiple registers: shrieking for the highest tones, shrilling (kula-like) for high, “casual” for low tones. Variable larynx position. Recoloring of the tones within a motif.	Single register for each onomatopoeic imitation, usually broadband—in contrast to the narrow-band kula. Usually high larynx. Overall dullness.	Contrast of 2 registers: head-voice (kula-like) for high tones and throat or chest singing voice for low tones. Variable larynx position. Recoloring of the tones within a calling motif.	Contrast of 2 registers: head-voice (kula-like) for one syllable and normal speaking voice for the rest. Variable larynx position.
	10. Harmonicity, attack and vibrato	Clear harmonics (akin to pure tones), ascending portamento attack and descending termination (in longer kulas), minimal vibrato (only to embellish a tone such as a trill).	Clear harmonics (akin to kula) for the highest and longest tones only, ascending and descending portamento, no vibrato.	Prevalence of non-periodic spectrum, harsh, noisy sound, little voicing (only if present in the imitated model), no portamento or vibrato.	Clear and rich harmonics for the lowest and longest tones only (with some vibrato), ascending and descending portamento for leaps only.	Prevalence of non-periodic spectrum, as in speech, with pronounced frequency modulation and noise. No vibrato. Leaps engage portamento.

*Ten AEs (in rows) are used in five types of phrases (in columns), each characterized by a unique combination of AE patterns, the most distinctive of which are pitch, rhythm, articulation, dynamics, and register. Each type also is distinguished by its semantic specialization: kula—safety signal for grazing, exclamation calls—social “grooming talk,” onomatopoeia—playful teasing, parlando—commanding and convincing, and motherese—endearing and trusting. Except for the long-distance kula (whose sentences can reach up to 15 s), all other types are quite brief (usually, 0.3–2 s) and are intermixed with the same-distance or shorter-distance motif types (i.e., kula phrases can be included in motherese recitative, but motherese cannot be included in kula). The maximal distance squeezes the ambitus into an octave confined to a single highest register. This compresses degrees into steps of a smaller or a longer intervallic size, depending on their phrasal position. Climactic step tends to constitute the interval of major 2nd, whereas cadential step—of minor 2nd, to emphasize and facilitate resolution of tension (“major” for a peak in tension, “minor” for relaxation). The framework of a breath cycle sets the basis for traditional association of major with happiness (climax = inspiration = maximal power) and of minor with sadness (cadence = expiration = collapse). Mid-distance kula transposes heptatonic structures to lower registers, fitting them into a tetrachord (pentachord, if a climactic motif is added). Octave equivalence secures heptatony. Closer distances enable alterations, flattening or sharpening of the unstable degrees, and timbral recoloring. Stacking phrases of contrasting TO and semantic values, learnable by humans and domesticated animals, generates the SFTO. See a fuller version of this table in Appendix 2 “A Comparative Structural Analysis of Musograms,” in Supplementary Material.*

• Kula (tropotrophic),
• Exclamations (phatic),
• Onomatopoeia (ludic),
• Parlando (imperative),
• Motherese/recitative (endearing/motivating).

Genesis of SFTO follows the path of human-to-dog whistling communication. Noteworthy, kulning’s exclamations and onomatopoeic calls engage stop- and fetch-whistle features (see Figures 5E,F).

The proof for SFTO’s pragmatic efficacy is in the herd’s fulfilling of the shepherd’s commands (Wallin, 1991, 410).

Yet another source of semiosis for kulning was phonemic symbolism. Complete absence of words in kula and minimal wording of motherese suggest the *prelingual* existence of kulning. Wallin (1991, 410–413) rightfully emphasizes that there is no reason to label kula’s sounds as “*phonemes*”—they are mere *homologues* to vowels and consonants, shaped by the anatomic-physiological conditions of breathing and acting while uttering. The same applies to traditional Alpine yodel (Fenk-Oczlon and Fenk, 2009a). Yodel and kulning vocables are formed not by phonological oppositions of local languages but by the communication distance and the extent of the desired stimulation/inhibition for a given call. Thus, the highest larynx and intensity at the onset of long-distance inhibitive kula-phrases generate a semantically “negative” [i], whereas a relaxed post-climactic position in the mid-distance tropotrophic kula generates a “positive” [å]. Similarly, glottal stops at phrasal beginnings and endings range from a gentle [h] to a harsh [tj], depending on the needed attack and tenuto decay (Rosenberg, 2014). The choice of the most common kulning syllables (Ahlbäck, 2007) can be explained by human/animal’s natural selection for effective distant communication (Wallin, 1991, 390).

**Monodization** of kulning was imperative in genesis of SFTO.

Animal communication usually employs male “chorus,” male-female “duetting,” or “antiphonal” formats (Yoshida and Okanoya, 2005). Musicologically, this corresponds to a special type of texture—“isophony”: the ongoing out-of-sync multi-part reproduction of the same thematic material (Nikolsky, 2018). Isophonic jumble precludes SFTO. For multifactorial patterning to emerge, each vocalizer must clearly hear his/her voice in order to track spectral changes without any contamination by a partner. This is how infants learn to make their own songs and how children acquire “musical ear” (Nikolsky, 2020). Even in non-European traditions that are exclusively *polyphonic*, such as Aka Pygmy, motherese and children-made music remain *monophonic* (Rouget, 2011). This is because an auditory stimulus must be objectified to become accessible for reproduction: a relation of 2 tones in certain aspect must be realized as an auditory constant to lay the foundation for construction of a musical mode (Nazaikinsky, 1973). For perception, the listener must discover permanence of the foreground “sound-object” against the background of a sound-stage, and memorize it in order to relate to it all of the subsequent changes in the thematic material.

Just as one cannot learn prosody of a language by listening to the crowd, one cannot learn SFTO by listening to isophony. And herding music promotes monodic application: herding demands hours of solitary interaction with animals, ideal for testing their response to music-making.

## Conclusion

Homo heidelbergensis was already anatomically capable of practicing proto-music which was most probably isophonic, lacking the combined coding of pitch/rhythm—without which conventionalization of the semiotically functional melody-making was hardly possible. Isophony supports only group communication of zero- and first-order intentionality, limited and conditioned by the genetically embedded instinctive responses to isophonic formula. Learning of multi-factorial particulate expression and second-order intentionality requires monophonic production. AE’s pattern becomes fully semiotic only when many senders/receivers remember it as the bearer of the same semantic value that connotes a certain affective state—“binding hearer to speaker” through “tying of some social sentiment” (Wallin, 1991, 420).

Emotional contagion is possible in isophonic signals, but it is primed to a single most salient AE—provided all communicators share the necessary neuro-anatomical substrates (Snowdon et al., 2015). Harmony, meter, texture, and form are not supported by non-human brains; neither is a premediated “construction” of an intended message. Animal interpretation of auditory signals is inherently circumstantial—determined by the signaling context (Zuberbühler, 2017). Therefore, human music is often “misunderstood” by animals, requiring music’s “translation” into animal’s “sonic templates of recognition” (Snowdon and Teie, 2013).

For ACs to evolve into music, a repertory of patterns of AEs had to be extracted from proto-musicking practice and abstracted into elemental signs to continuously inform someone(s) of the communicator’s affective state, intentions, and needs. Such use emerged in communication with domesticated dogs, thereafter, adapted for herding. Hunting/gathering does not demand such communication. Instead, it prioritizes *collective* collaboration: bringing participants emotionally “in-tune,” binding them into a group to increase one’s powers. Such use makes sense in situations of using loud complex sounds while hunting large prey and repelling human predators in open savannah space (Jordania, 2011). Large groups of big-game foragers tend to prioritize *collective* music-making over *personal*, confining the latter to pre-pubertal age, like Aka Pygmies (Rouget, 2011). Homo probably exported isophonic proto-music from Africa to Europe.

The last Glacial Maximum greatly reduced the European population by the Gravettian—until the Magdalenian repopulation (Maier, 2017) enabled the rise of symbolic cultures (Kozłowski, 2015) and ethnolinguistic genesis (Zilhão, 2014). Low-density foraging groups usually form alliances, cemented by linguistic commonalities and intermarriage (Marlowe, 2005). Music surpasses language in its bonding capacities (Nakata and Trehub, 2004). Gravettian proto-music must have adjusted isophony for new cultural applications, especially religious. Smaller groups generate a smaller sonic “jumble,” facilitating the recognition of specific musical elements. Smaller groups also promote honesty in communication (Richerson and Boyd, 2005). Honest musical expression enables and validates the person-to-person musical communication. This opens doors to the cultural development of a motherese communicative model. Small groups are likely to promote motherese-like duetic and babbling-like solitary music-making. Thus, collective music-making is exceedingly rare in Northern Siberia (Alekseyev, 1967) which has always remained underpopulated (Sikora et al., 2019)—closely resembling life in glaciated Europe.

Motherese talk, lullabies, onomatopoeia, and instinctive utterances supplied the initial material for the formation of bi-specific SFTO. Changes in distance while continuously communicating with the herd put in place the musical modes. The closest distance promotes low-register monotony, middle distance—high-register monotony, long distance—tetrachord-based tonicity, and maximal distance—conjunct pentachord/tetrachord octave-equivalent modes with dominant-tonic functionality. Monotony increases the tuning accuracy of anchor-tones, firstly defining principal degrees (tonic, supertonic, dominant), and then additional unstable degrees (Alekseyev, 1976). Characteristic modal intonations of different phrasal styles and varying position within a breathing cycle charge modal degrees with specific functionality, which directs the formation of semantic values for each of the common modal intonations. This triggers the process of modal evolution as outlined by Beliaev (1963) and elaborated by Nikolsky (2015a, 2016).

Nordic kulning is probably a vestige of an archaic cattle-oriented “domestication language” which descended from yodel—accompanying the northerly spread of Indo-European languages throughout Europe. Other Eurasian domestication languages accompanied the spread of the Uralic and Turkic language families, and were optimized, respectively, for reindeer and horse. Rémy Dor cross-analyzed vocalizations/whistles of herders speaking 20 Turkic languages, from Anatolia to Yakutia, and inferred their syntactic organization (Dor, 2005), identifying their common utterances (Dor, 1993). Like Wallin and Alekseyev, Dor too found continuity between vocalizations of hunters and herders: “somatotropic” vocalizations, designed to make the prey come closer, evolved into “fetch” or “home-return” calls, while “somatofugal” vocalizations evolved into “stop” calls to repel predators. The new class of “somatoneutral” vocalizations emerged in order to keep an animal at a constant distance (like safety-call kula). Strong biological foundation of this distance-governed communication made it well-conserved—practically indestructible—unlike languages or music systems (Dor, 2008).

Domestication languages could underlie modern languages and musics, as traditional beliefs suggest. Swedish rural informants considered kulning an ancient “language” (Moberg, 1971, 145). And on the opposite end of Eurasia, Mongolian herders believe that their music-making is derivative of the “large language,” superior to human language and designed to communicate with animals, nature, and spirits (Pegg, 2001, 235). Altaic *xöömii* most likely constitutes yet another “domestication language.”

Capacity to simultaneously control numerous AEs and second-order intentionality enabled humans to create a heterospecific semiotic system of communicating desirable affective states, which gave humans control over domestic animals, resolved human sustenance needs, and put in place music as we know it. The semiotically functional tonal organization that distinguishes music from speech might have emerged no earlier than during the Neolithic “revolution” as a result of forging new conventions of human-to-animal vocal communication.

## Directions for Future Research

Comparative examination of human-to-animal signaling for different domesticate animals across different geographic regions can confirm whether the paradigm of “musical domestication language,” divisible in “dialects” and integrable into “language families,” is applicable here.

Collecting a database of patterns of human-to-animal communication would be analogous to building a lexicon of a newly discovered natural human language or to establishing a stock of typical idioms in the musical communication within a novel musical culture. Once established, such database can be statistically analyzed and cross-examined in relation to other databases, e.g., of emotional expressions in music. This could substantiate or invalidate my conclusions.

The perception of specific elements and patterns of human-to-animal communication by humans and animals can be experimentally tested. This could identify syntactic and pragmatic rules that cannot be assessed by acoustic analysis alone. Together, both approaches can evaluate semiotic efficacy of TO in pastoral signaling. This, in turn, can establish whether introduction of herding communication during the Neolithic Revolution was capable of generating SFTO in music to make it break away from the basics of animal communication.

Experimental archaeo-ethnomusicology could provide yet another way of verifying this hypothesis. Members of isolated tribes that maintain a hunter/gatherer lifestyle and use no domestic animals can be introduced to domestic animals and “taught” to use music-like signals to command them. Their progress can be analyzed and compared to patterns of conspecific acquisition of music skills by human infants, as well as to the available archaeological, genetic, and paleo-physiological data.

## Author Contributions

The author confirms being the sole contributor of this work and has approved it for publication.

## Conflict of Interest

The authors declare that the research was conducted in the absence of any commercial or financial relationships that could be construed as a potential conflict of interest.

## Footnotes

1 Metric organization of rhythm accompanies and supports pitch organization in music (Jones and Large, 1999), jointly supporting the “musical” manner of interpretation of sounds (Huron, 2006). Tonally important pitch-classes are usually stressed by longer durations and/or dynamic accents. However, comparing to rhythm, pitch organization is much more common in known world’s music cultures (found even in music for only percussive instruments, e.g., African talking drums)—there are many forms of music that are characterized by ametric and arrhythmic free timing, but there are very few non-pitch forms. Therefore, pitch organization is a more reliable marker that distinguishes music from language than rhythmo-metric organization.

2 I will use the abbreviation “AE” when speaking of a single aspect of expression, and “AEs” when speaking of multiple aspects of expression.

3 The matter of choosing different timbres for different musical expressions has traditionally been handled by the discipline of instrumentation in Western classical music (Banshchikov, 1997). The term “instrumentation” here is somewhat misleading, because it covers not only the qualia of the timbres of musical instruments and their ensembles (trio, quartet, orchestra, orchestral group) but also various types of voices (soprano, tenor, bass), vocal ensembles (duet, trio, choir) and the rules of combining vocals with instruments (Kreitner et al., 2001). Arabic maqam, Persian dastgah, and Indian raga also observe similar rules in their respective practices.

4 Technically speaking, monophonic music can still engage some idioms that relate to harmony and texture. A melody solo often features a pronounced “harmonic rhythm” (Swain, 2002)—i.e., periodic changes of implied chords (e.g., the “Blue Danube Waltz” theme by J. Strauss Jr.) that can stay regular (as in a metric pulse), be patterned (as in rhythm), or elaborated by expansion or contraction of a pulse period. Monophonic music can also implement changes in texture by patterning a stream of sounds into familiar textural idioms (e.g., the “Alberti figuration” or tremolo on a single tone) which then carry their specific semantic expression, different from other textural components, such as a melodic theme (Skrebkova-Filatova, 1985). However, overall, harmony and texture play a secondary role in monophonic compositional practices, limited to Western classical music alone.

5 The “integralist school” of structural analysis of music was founded by the father of systematic musicology in Russia, Viktor Beliayev, in the 1920s, during his tenure in the Moscow Tchaikovsky Conservatory (Beliayev, 1990b). Beliayev’s approach was further developed by two leading Moscow theorists, Leo Mazel and Viktor Tzukkerman (Mazel and Tzukkerman, 1967). They sought to integrate thorough structural analysis of a musical work with the psychological and sociological analyses of the expressive means employed in the analyzed musical work (Khannanov, 2005). It was especially Mazel who was concerned with broadening the framework of analysis to encompass not only the domains of melody and harmony, traditional for Western musicology, but also aspects of rhythm, meter, texture, articulation, dynamics, and timbre. After Yevgeny Nazaikinsky’s death in 2006, the leading Russian “integralists” are Valentina Kholopova and Vyacheslav Medushevsky.

6 The word “movement” here refers to a principal division of a longer music work into sizeable sections, each distinguished by its own metric organization and tempo: e.g., a 4-movement symphony or a 3-movement sonata. The concept of movement emerged in 16th-century Western classical music to reflect on the old practice of switching from one tempo to another within the same piece of music (Sadie, 2001). However, by no means the use of multiple movements within the same work is exclusive to Western civilization. Well known are non-Western genres of music that employ cyclic arrangement, such as Arabo-Andalusian nubah (Touma, 1996) or Javan court Gamelan music (Sutton, 1991).

7 Ontologically, it is necessary to distinguish between “meaning” in a natural language and “meaning” in a cultural system of symbols (such as music)—especially in light of the difference in their acquisition: thus, under experimental conditions non-human primates can acquire some symbolic systems but not a full-fledged human language (Balari et al., 2011). It seems that the verbal combinatorial semiosis of referential meaning is fundamentally different from retrieving imagery, be it emotional or motivational information assigned to cultural symbols. This distinction is crucial for the investigation of origins of human language and music. Here, music, despite its combinatorial nature, occupies a place closer to signal-like semiotic systems, which *makes music more accessible to hominins than language*.

8 Entrainment (from French “*en*-” + “*traîner*”—to drag something along) is the term used in physics to address a wide range of phenomena where two oscillators are coupled, and one of them gradually comes into synchrony with the other, becoming locked in a phase. Entrainment of two pendulum clocks was discovered by Christiaan Huygens in 1666 but was explained only few centuries later. In early 20th century, other manifestations of entrainment were unveiled in acoustics (coupling of the organ pipes) and biology (glimmering fire-flies)—until it was generalized as a universal physical phenomenon (Pikovsky et al., 2001). Its biomusicological manifestations were identified in the 1990s, at first in relation to music therapy, and thereafter as an integral part of perception of rhythm and meter (Large and Kolen, 1994), of great importance to the evolution of music (Fitch, 2012).

9 Thus, Titon (2015), one of the leading Western ethnomusicologists of today, goes as far as defining the discipline of ethnomusicology as “*the study of people making music*”—rather than “the study of music” as the term “musicology” indicates (the study of human societies is conducted by another discipline—“anthropology,” reflected in the etymology of its name). Paradoxically, modern Western “people’s ethnomusicology” still shuns the Soviet ethnomusicology which shared the same approach, holding music as “belonging” to people and “reflecting” people’s mentality, while remaining totally free of the anti-textual bias (Panteleeva, 2019).

10 Gourlay argues that *no musicological study* of African music *by outsiders is justified*, because “in no African language about which we have information, and in many used by other peoples who have oral rather than written traditions, is there a word corresponding to the English term ‘music’.” So, according to Gourlay, “where the term ‘music’ is unknown to the people in question, one can conclude only that what we are presented with is *the investigating scholar’s* concept of *his/her* ‘music’.”

11 Parncutt and Hair subscribe to Gourlay’s defiance of a scientific investigation for those phenomena that do not find a corresponding term in a native language. They categorically insist that the *research of consonance and dissonance be constrained only to music of such cultures that define the concepts of consonance and dissonance*: “if musicians in that culture do not talk directly or indirectly about C/D [consonance/dissonance], it is considered irrelevant.” By this logic, there is no gravity in those countries whose native people do not have a word translatable in English as “gravity.” Parncutt and Hair see the goal of studying music in “documenting the musical and music-theoretical discourses of the insiders about which tones and rhythms should be played together and why, and considering the political and psychological mechanisms that are allowing Western music to dominate world music”—undoubtedly, a controversial and a politically biased agenda.

12 To substantiate this criticism that is rarely voiced in modern Western literature, I shall quote one of the biggest authorities in ethnomusicology (the emphasis is added by me): “*Functional analyses of musical structure* cannot be detached from *structural analyses of its social function*: the function of *tones in relation to each other cannot be explained adequately as part of a closed system* without reference to the structures of the *sociocultural system* of which the musical system is a part, and to the biological system to which all music makers belong” (Blacking, 1974, 30–31).

13 One of the main reasons for the drop in standards of musicological and ethnomusicological analyses is that in the US and UK academic curricula, music theory in general, and music analysis in particular, have been offered as rudimentary undergraduate courses (Agawu, 2004). In contrast, in countries of the former Soviet Union, music analysis has been taught at the highest level of scholarship that requires at least 10 years of study before attaining a level of training where an analyst is expected to capture and interpret the totality of expressive means employed in a music work (Khannanov, 2005).

14 In some songbirds, the innate encoding consists of smaller elements, resembling syllables, and following simple rules for how to order them, so that a bird actually learns to “assemble” its song. However, the assortment of such elements is very limited, making songs signal-like, restrained to a single species. Playback of isolated syllables of such songs either does not elicit response or produces a weak reaction in other conspecific birds (Searcy, 1992). Perhaps the rearrangement of elements constitutes not a pragmatic, but a “syntactic” production unit—thus, zebra finches were found to stop at syllabic breaks in a song, when detracted (Cynx, 1990). Rearrangement of “syllables” is also used by a few primate species (gibbon) to disclose the identity of a caller for conspecific animals (Marler and Mitani, 2008).

15 Although it is not uncommon for ACs to form a sequence according to a rule-based structure, noticeable by conspecific animals (Fitch, 2010, 182), changes in such structures apparently do not result in the changes of meaning of the entire song (Hauser, 2000). The most syntactically elaborated bird and whale songs use combinatorial features, albeit minimal. However, despite having a componential structure, such animal song in its entirety presents a *single* piece of information learned from the animal’s parent holistically rather than incrementally, element by element (in contrast to how humans learn), and is therefore highly stereotypical in form (Hurford, 2012, 3–99).

16 The concept of phonocoding (i.e., “phonological coding”) was introduced to oppose “lexicoding” of human speech (Marler, 2001). Phonocoding refers to the capacity to generate new sound patterns by recombining the constituent elements and components of known conventional signals. This capacity is minimal in non-human primates, but common in learned vocalizations of songbirds and whales, which, however, remain primarily non-symbolic and affective.

17 The term “semiosis” here refers to the Peircean concept of conveying information by encoding it into signs by one party and decoding it by another party—a “two-ended” system. A “one-ended” call can be somehow interpreted in relation to the situational context by the listening animal, but this interpretation can radically differ from the actual state of the sender: e.g., bird’s mating call might be interpreted by a nearby cat not as a signal of readiness for mating but as a signal for hunting. Then, the integrity of the information passed from sender to receiver is not preserved. Within this context, the use of the term “meaning” in regard to an AC, adopted in biosemiotics (Sebeok, 1994, 111), is confusing, since “meaning” implies that someone “means” something by displaying a specific sign. More accurate here would be to employ the term “*significance*” (as in “to signify”) instead of “meaning.”

18 By “semiotically functional,” I mean that a music-maker selects the elements and components of tonal organization for each of the aspects of expression in music based on their efficacy in conveying specific affective information (“musical emotion”) to his/her listeners and/or partners in performance. In this sense, the AC can be considered “semiotically dysfunctional”—not supporting a successful two-ended communication (delivery of the intended message) between the sender and the receiver.

19 The word “flute” here is used informally: there is not enough archeological evidence to conclude if the earliest instruments were flutes or clarinets. The oldest artifact is a bone fragment from Haua Fteah, Libya, with a single hole, dated 90–110,000 years ago (Blench, 2013). Most archeologists do not recognize it as man-made. Next in line is the 47,000 years old 3-hole artifact from Divje Babe, Slovenia, uncovered in 1995. It was interpreted as a bone bitten by a carnivore (D’Errico et al., 1998). However, experimental testing has demonstrated that none of the cave bear, wolf or hyena dentition could punch two holes without cracking and splitting the bone (Turk et al., 2001). Nevertheless this argument was not accepted by the supporters of non-human origin of the Divje Babe artifact (Morley, 2006). Subsequent tomographic analysis has concluded that the Divje Babe artifact was man-made (Tuniz et al., 2012). Slovenian researchers have presented additional reasons for its man-made origin (Turk, 2014). In spite of this, another recent British study has restated the bite origin hypothesis (Diedrich, 2015)—though, without addressing the 2012 and 2014 studies’ arguments. The third in timeline and unequivocal in its provenance, is the 5-hole Hohle Fels-1 flute, 35,000 years old (Conard et al., 2009).

20 Maynard-Smith and Harper give an example of such ritualized physiological cues as thermoregulation that causes animals to raise their feathers/hair to reduce body temperature, heightened in social interaction—which makes an animal appear larger and promotes dishonest signaling of increased body size in instances of confrontation (p. 68). Other physiological cues are respiration, urination/defecation, pupil dilation, and yawning (p. 69). The ritualized behavioral cues include “intention to move” which signals the beginning of a significant action (a bird taking a few false starts before flying), “protective movement,” and “displacement behavior” (p. 70).

21 For thorough explanation of the visual representation of the multifactorial organization of music, a way of its quantification, and its difference from the prosogram approach by Mertens, see Appendix 1 “A New Method of Modal Multifactorial Analysis of Tonal Organization in Music” in Supplementary Material.

22 Musicological literature identifies many more structural patterns of different AEs than the patterns listed in Table 3—and their semantic references include many more affective states than merely five basic emotions. Much of this information is dispersed in the treatises on music theory, some of which are cited in the beginning of this paper. There are very few books that list such structural patterns in a manner of the 18th century treatises of “musical lexicon” (Cooke, 1959; Mattheson and Harriss, 1981; Bartel, 1997; McCreless, 2002; Vashkevich, 2006). However, only isolated patches of such literature have attracted attention of psychoacousticians and received experimental trial (Kaminska and Woolf, 2000). For this reason, the metareviews on research in “musical emotions” tend to focus exclusively on 5 basic emotions.

23 Although tempo, rhythm, prosodic contours, and registers contribute meaningful motivational and attitudinal information to verbal communication, by no means can they be regarded as its primary semiotic aspects. Without knowing the lexic meaning of words of a particulate language, inferred from phonetic structures of auditioned speech, no adequate understanding of that speech is possible. This is in polar opposition to musical semiosis, where tempo, rhythm, melodic contour, and register directly convey the most important information, whereas keeping the referential meaning optional.

24 The opposition of conspecific and heterospecific distribution of acoustic features that characterize the vocal expression of a particular affective state in AC allows a researcher to identify those patterns of AEs that match cross-cultural features of corresponding affective states in “musical emotions” of human music. The patterns of expression that are present across multiple animal species are more likely to form the equivalents of “universal” traits of human “musical emotions” than those patterns that are found only within the very same animal species.

25 However, the idea of rhyming seems to have a precursor in ACs. Thus, humpback whales match the constituent syllables in some of their songs (Payne, 2001). A similar organization was noticed in mockingbird songs (Thompson et al., 2000). Its underlying cause is perhaps simplification of memorizing a complex song. Yet another cause could be the employment of repetition of a particular syllable in a song for a certain number of times as a conspecific marker for certain bird species (Fitch, 2010, 183). Hearing such birdsongs might have prompted humans to invent rhyming.

26 Thus, newer paintings often covered the older ones: hiding the underlying image did not matter—once painted, an image was “brought to life,” and stayed “alive,” even if masked—just as a person who disappears from our sight does not die (Uspensky, 1995, 173–181).

27 The earliest age when infants show the ability to recognize changes in pitch contour is 5 months (Chang and Trehub, 1977). Majority of studies demonstrate such capacities in older children, 6 months and up (Trainor and Hannon, 2013). The ability to recognize changes in rhythmic values of a familiar music seems to emerge quite earlier—at 2 months of age (Demany et al., 1977).

28 Metricality, along with tonality, influence primarily the Western musicians: non-musicians process melodic contours mostly according to the distribution of longer rhythmic values (Monahan et al., 1987). Non-trained listeners simply cannot ignore rhythm—as it governs their melodic recognition (Jones and Ralston, 1991). Majority of young and inexperienced listeners at first parse melody by rhythm and only then by pitch contour and mode (Halpern et al., 1998). Tempo/rhythm descriptors are much more prevalent in listeners’ judgments of thematic similarity than of pitch contour (Addessi and Caterina, 2000; McAdams, 2004).

29 Of course, the influence of rhythmic features on the judgment of melodic similarity is far from being simple and direct. Other factors, such as tempo and harmonization, can affect the extent of autonomy of temporal and frequency-related aspects of music (Prince, 2014).

30 There are accounts of “tone-painting” where the contour of the hills is represented through the melodic contour in songs of indigenous hunter/gatherers of Northern hemisphere (Krushanov, 1987, 234) whose life style is comparable to that of Aurignacians. However, the idea of such representation most probably was inspired by the need in mnemonic aid in long-distance navigation during migrations with reindeer herds, which doubtfully existed earlier than a few thousand years ago (see the last chapter). Such tradition had chances to survive the ongoing extinctions in harsh climate only as a part of a reliable subsistence strategy for a fairly large population.

31 Broad-scale technological clustering originated in the earlier Aurignacian tradition—attributed to the long-term influence of the ethnolinguistic variation. Forming of a continental culture during the Gravettian indicates the increased language contacts between different “clusters,” establishing pan-European networks of informational exchange (Zilhão, 2014).

32 The term “ethos” was coined in Archaic Greece, where it originally meant “custom,” but by Classic times it obtained the meaning of a certain affective “character,” associated with a particular musical melodic mode. “Ethos” embodied the consensus within a community as to which affective states would be generally “good” or “bad” for that community. The doctrine of “ethos” is closely related to the concept of “harmony of spheres,” attributed by Hellenic sources to Pythagoras, who presumably learned it from Babylonians. The discussion of ethical value of this or that musical emotion and its suitability for astrological dispositions constituted an important part of public discourse in Ancient civilizations of Near and Far East, as well as Central Asia.

33 Thus, Peter Bogucki counts as few as 14 Mesolithic “social territories”—i.e., regions differentiated by the material culture as manifested by archaeological evidence—spread out over the entirety of Western Europe during its transition from the Boreal to the Atlantic periods, c. 7500 kya (Bogucki, 1988, 41–46).

34 It could be said that an animal “centers” (i.e., focuses) on a single aspect of vocal expression, conserving the extent of increase or decrease in intensity of the psycho-physiological state that is associated with that vocal expression. This is yet another parallel between AC and the vocalization of a sensorimotor human infant. This is in contrast to the ability of an adult human to simultaneously conserve multiple dimensions of changes in multiple AEs in music.

35 For a comprehensive analysis of those musical examples that were selected for musograms in Figures 2, 3, 4, and 7, see Appendix 2 “A Comparative Structural Analysis of Musograms” in Supplementary Material.

36 Similarity of the anatomy of the supralaryngeal vocal tract of the human baby and the ancestors of Homo sapiens provides yet another justification for seeking the TO model of hypothetical Paleolithic music in the musical babbling of 1–2-year-old infants.

37 Demonstration of musical “method-acting” can be found in the video clip of Andrei Gavrilov performing Rachmaninov’s Prelude in g minor, op.23, No.5 https://www.youtube.com/watch?v=T3AEfMMyH6A. Especially telling is the pianist’s facial expressions, as he is getting up from the piano bench after completing his performance—he continues to remain in his “role.”

38 Some indigenous traditions have developed professional forms of musical art which require aesthetic evaluation (e.g., Tatar, Kazakh, Mongolian). However, they still fundamentally differ from Western classical music by not taking a musical work as a “script’ created by the composer for the performer to adhere to (Zemtsovsky and Kunanbayeva, 2011). Only the Western musician is trained as part of his occupation to accurately “execute” the composer’s script while being aware of the fictiveness of its emotional content. However, application of such treatment to a folk cover song is most likely to come across as fundamentally “inauthentic” and detrimental to the song (Moore, 2011).

39 Berlin’s rules Nos. 3, 6, and 9 call for the composer to please the consumer at the cost of insincerity: “the ideas and lyrics must suit *either a male or a female*, so both sexes want to *buy a song*,” “music and lyrics *must* have to do with *things common to everyone*,” and, most explicitly—“songwriter must look upon the song as a mere business, *not take music to heart*.” Berlin’s rules break away from the Western composer’s “canon,” established since the introduction of “musica reservata” in the 16th century (Meier and Dittmer, 1956). For this reason, Berlin’s approach provoked criticism of the American popular music in toto, seen by connoisseurs of art music as a “sweet lie” sold (for profit) to the mass audience to replace music that is “truthful” yet unpleasant in revealing “social truth” (Adorno, 1942).

40 As far as I know, Trehub (2008) remains the only scholar who believes that music, in general, operates by having the performer emotionally deceive the audience. Other scholars who point out that a professional performer can evoke emotions that he/she does not actually feel, realize that this discrepancy is possible only in music that segregates the listener, the performer, and the composer. *This solely happens in Western classical music*. And even within this tradition “deceiving” the audience is still regarded as a fault to be avoided. Noteworthy, Trehub did not respond to Juslin and Västfjäll’s (2008) objection to her criticism.

41 Prevalence of ascending contour characterizes happiness, anger, and fear. Happiness differs from anger and fear by employing variety of melodic contours called to diversify an ascending contour. Anger differs from fear by using sharp rather than wave-like contours and by dominance of staccato articulation in pitch changes (fear mixes staccato and legato articulations). Prevalence of descending contour characterizes both, sadness and love. They can be distinguished solely by intonation: flattened with stepwise falling contours for sadness, and sharpened with occasional ascending leaps for love.

42 There is a wealth of terms used in Scandinavian countries to refer to herding vocalizations (Rosenberg, 2003, 8). Although the term “kulning” (kolning) is most commonly used in English in relation to the special technique of the long-distance vocal calling, I follow Wallin (1991, 387) in reserving the term “kula” (he uses the alternative spelling “kola”) which in Swedish means “to make a distant call” exclusively for long-distance communication. This is necessary, because long-distance “kula” calls are routinely inserted in mid-distance and close-distance vocalizations, while it is the long-distance “kula” style that distinguishes shieling vocalizations from other forms of traditional Scandinavian music.

43 It should be noted that the peculiar status of pastoral music as a form of *heterospecific* communication is responsible for the emic views on kulning as **non-music**. This is yet another confirmation of the need in the etic approach. Across Eurasia, herder-made music is distinguished from “normal” music as a form of “magic.” The profession of the herder is traditionally associated with sorcery: herders are believed to sign a contract with the evil forest spirits, receiving magic power for vocal and instrumental music-making in exchange for not using their gifts publicly, under the threat of death (Plotnikova, 1999b). At the eastern end of Eurasia, in Altai, supernatural beliefs are even stronger, reserved not only for professional herders (*chabans*) but for all livestock-owners who use pastoral spells (Kondratyeva, 1996). All vocalizations of this type are considered non-music—to the extent that informants perceive any request to “sing” a spell as being ridiculous.

44 Noteworthy, despite a 16-hour-long workday and insecurity of living alone without any weapons, shieling jobs were always highly sought after, since women remained in charge of their summer life and enjoyed freedom unavailable to them at the farmstead (Rosenberg, 2014).

45 The scope and the method of geomusicology were introduced by George Carney (Nash and Carney, 1996). Izaly Zemtsovsky formulated an analogous approach in his proposal to establish a new discipline of ethnogeomusicology (Zemtsovsky, 2005).

46 Thus, Finnish “ringing calls” present a form of vocalization that acoustically and culturally resembles Swedish kulning while featuring a few unique traits (Uttman, 2002). Occasionally, ringing calls are performed by men (falsetto), utilize a peculiar lip technique (generating the “phui”-like tonal quality), and engage “darker” vowels.

47 Cattle definitely carried special symbolic significance in Neolithic England (Ray and Thomas, 2003). Cattle received the same funeral treatment as humans in Danube winter burials as part of the Sun cult which thrived throughout the 4th millennium BC, probably because of drastic swings in solar activity (Horvaìth, 2012). The second millennium BC Linear-B tablets from Knossos testify that, unlike sheep/goats, cattle was given names, bestowed with individuality—and was associated with royalty and sacrificial rites (McInerney, 2010, 50–53).

48 Lactase persistence was completely absent in early Neolithic population 5500 BC (Burger et al., 2007), making its first appearance in Scandinavia in 3400 BC (Malmström et al., 2010), by 3000 BC in Iberia (Plantinga et al., 2012) and taking over Europe thereafter (Marciniak and Perry, 2017). This timeframe agrees with the scenario represented in Figure 6.

49 The Indo-European family contains 144 languages divided amongst 11 distinct branches—with even more languages most certainly having existed in the past but gone extinct (Diamond and Bellwood, 2003). In Europe, non-Indo-European languages are limited to merely 11 documented languages (only 8% of the total number of languages): Etruscan, Basque, Iberian, Tartessian, Estonian, Finnish, Urartian, Sumerian, Hurrian, Hattic, and Mitannian—plus 3 undocumented languages: Pictish, Lepontic, and Ligurian (Robb, 1993).

50 Herders routinely let their reindeers graze unsupervised for a rather extensive length of time. Inevitably, many animals become lost, turn wild, and can then be hunted (Stépanoff et al., 2017). Also, the herder’s strategy of searching for his lost animals strikingly resembles that of hunting.

51 This caused the import of non-native reindeers via the emerging Russo-Finno-Scandinavian markets and transition to pastoralism (Røed et al., 2018). Genetic evidence points to 3 epicenters of reindeer domestication: Fennoscandia, Western and Eastern Russia (Røed et al., 2008). Reindeer domestication took about 6000 years. Its earliest evidence comes from the 4000 BC petroglyphs (Helskog, 2012), a 1510–1130 BC burial (Murashkin et al., 2016), and the paleolinguistic tracking of words for reindeer that date back to 1500–1000 BC (Aikio, 2006).

52 Ivarsdotter describes how goat-calling follows the model of cow-calling, adapting it to the livelier nature of goats, notorious for their proneness to naughtiness (Ivarsdotter, 2004). Similarity of cow-calling (*Kolock*), goat-calling (*Getlock*), and sheep-calling (*Fårlock*) is obvious from listening to their archive recordings published by Swedish radio (Ivarsdotter, 1995). The same similarity is retained in pastoral incantations and spells that survive in Altai region—all three types of calling differ primarily in the prevalence of different phonemes for each of these three animals (Tiukhteneva, 2017). The musical characteristics of all three types of calling closely resemble one another (Kondratyeva and Mazepus, 1999). This suggests that similarity between cow, goat, and sheep pastoral communication is a wide-spread Eurasian phenomenon.

53 The highest SPL level is reached at a 30 cm distance from the sound source (125 dB) which exceeds the ear’s pain threshold at 120 dB (Rosenberg, 2014). The average SPL of kula at 1000 Hz is 113 dB. This is dynamically comparable to an operatic soprano singing fortissimo, except that the soprano’s technique requires maintaining a fixed larynx configuration at a *low* position. However, the maximal SPL of the soprano does not exceed 90 dB near the lips and does not change much from modulating the pitch (Johnson, 1984).

54 The term “parlando” was adopted by Anna Johnson in her report (Johnson, 1979) despite the traditional use of this term to refer to Western operatic singing that imitates speech and engages speaking “voice registers” (Sicoli, 2015) despite the absence of such intention for *kulerska*. Sung out words of close-distance kulning surprisingly resemble the operatic “parlando” sound. The kulning parlando contrasts the recitative kulning that minimizes voicing and remains much closer to talking than to singing, especially in its dynamics. The opposition of kulning parlando and kulning recitative resembles the opposition of operatic parlando and secco recitative, on the one hand, and the genre of melodrama that became popular in Western classical music in the 19th century, on the other hand.

## Supplementary Material

DATA SHEET S1 Appendix 1 – A new method of modal multifactorial analysis of tonal organization in music and music-like sounds. This technical paper contains instructions for identifying the tonal organization in a music work, a music-like vocalization (e.g., infant’s babbling) or music-like animal signals (e.g., bird’s song) – including sounds that are indefinite or modulating in pitch.

DATA SHEET S2 Appendix 2 – A comparative structural analysis of musograms used in Figures 3, 4, 7 of this article. This document contains a comprehensive analysis of the characteristic traits of tonal organization in the examples of human musical communication, animal vocal communication, and bi-specific communication between domestic animals and their human guardians.

## Abbreviations

AC: animal call (plural ACs)
AE: aspect of expression (plural AEs)
FF: fundamental frequency (plural FFs)
ky: thousand years
kya: thousand years ago
SFTO: semiotically functional tonal organization
TO: tonal organization.
