# Supplementary material for: The Pastoral Origin of Semiotically Functional Tonal Organization of Music
Source: Front Psychol. 2020 Jul 23;11:1358. doi: 10.3389/fpsyg.2020.01358 (PMC7396614; doi:10.3389/fpsyg.2020.01358)
Supplement: DATA SHEET S1 — Appendix 1 – A new method of modal multifactorial analysis of tonal organization in music and music-like sounds. This technical paper contains instructions for identifying the tonal organization in a music work, a music-like vocalization (e.g., infant’s babbling) or music-like animal signals (e.g., bird’s song) – including sounds that are indefinite or modulating in pitch. [file Data_Sheet_1.docx]

# **Method of modal multifactorial analysis of tonal organization in music.**

## Abstract

In this technical paper, I will describe a procedure that can be used to analyze tonal organization (TO) in a monophonic^[[1]](#footnote-1)^ musical composition, such as a solo song, solo instrumental improvisation, or a discrete melody in a monodic texture (e.g., a vocal part in a song with instrumental accompaniment), which have been created within a music system where frequency acts as the primary means of TO. The proposed procedure would handle majority of the world’s known musics, ranging from Western tonality to “ekmelic” (indefinite-pitch based) modes of indigenous Siberian ethnicities. The TO of musical vocalizations by human infants and of animal calls can be approached in the same way as well. This should allow a researcher to identify those methods of integration of musical tones that are most common in practice of musicking across the world and to distinguish human music from speech and from animal communication. Potentially, the analysis of TO can deliver an additional novel source of information on the syntax, semantics, and pragmatics of musicking—extracted from musical structures alone (rather than established from accounts of musical behavior and interviews of music experts and users) in a way similar to the way in which a discourse analysis of recordings of speech can provide an insight into the semiotic organization of verbal languages.

Table of Contents:

[1. Intervallic scaling 2](#_Toc36853985)

[2. Articulation grading 3](#_Toc36853986)

[3. Breathing gradations 7](#_Toc36853987)

[4. Melodic directionality and modal inclinations 8](#_Toc36853988)

[5. Ten rules of melodic articulation. 13](#_Toc36853989)

[6. Graphic melodic chart 16](#_Toc36853990)

[7. Six rules of melodic voice-leading 21](#_Toc36853991)

[8. Motivic analysis and inference of hierarchy in TO 23](#_Toc36853992)

[9. Six rules of distribution of tonal gravity 30](#_Toc36853993)

[10. The matrix technique of identifying characteristic modal intonations 32](#_Toc36853994)

[11. The interval strength in a mode and 6 intonational rules 37](#_Toc36853995)

Theories of mode and musical intonation, developed by Russian musicologists (Alekseyev 1976, 1986, 1988; Asafyev 1952; Beliayev 1990b; Mazel 1952, 1982; Protopopov 1930; Skrebkov 1967, 1973; Tzukkerman 1965, 1975; Yavorskii 1908) provide a methodological framework for the interpretation of the acoustic features that the modern generation software can extract from the audio recordings of music. Technically speaking, the principal challenge here is to identify the characteristic traits of tonal organization (TO) of a particular music piece or music-like vocalization by means of an automated time/frequency/amplitude analysis of an audio, and to interpret the results according to the appropriate valid musicological modal theory.

The description of the principles of such analysis and its interpretation was omitted in the main article that was focused on the contribution of each of the multiple aspects of expression (AEs) in human music and animal calls to communicate basic emotions. The current presentation demonstrates the entire analytic procedure on the example of a Yakut lyrical song “Sae Dyige”—the same example that was used in the Fig.1 of the main article (“The pastoral origin of semiotically functional tonal organization of music”) but was limited to only one version (the first). Here, I compare two different renditions of this song by the same singer, performed right after one another, since together they reveal the special intervallic typology^[[2]](#footnote-2)^—what is called “**ekmelic**” in Russian musicological literature.^[[3]](#footnote-3)^ Ekmelic typology, very common in some indigenous musical cultures (e.g., Siberia, Northern America, Australia), earliest forms of infant vocalizations (cross-cultural), and in some forms of Western music (e.g., rap-songs, operatic recitatives “secco” and liturgical recitatives in plainchant), is not analyzable by any existing analytic method other than the one offered below. I identify the most important parameters of TO, formulate the principal rules of their interpretation, and show how they apply to “Sae Dyige.”

#### Intervallic scaling

At the core of modal analysis stands the procedure of **intervallic scaling** (Starostina 1973)—called as such because it “scales” the musical representation of distances in a manner of the geographic map. A musical “scale,” in this case, can be seen as *a graduated range of values for measuring and grading the systemic frequency relations between the tones that are used to make a given musical work*. This meaning incorporates the traditional meaning of “musical scale” as a set of pitch classes (i.e., degrees of a musical mode), arranged in a progressive order (bottom-up or top-down). Such “musical scale” serves as a tool to generalize the musical mode(s) utilized in a musical composition. Intervallic scaling infers the pitch classes and the interval classes that characterize a particular musical mode known to a music-maker and pre-selected by him/her for expression of a musical idea. The reverse operation is executed by a listener of music who matches the auditioned sounds to the familiar musical modes and finds the closest match based on the structural resemblance and the semantic conventions.

Since the choice of intervallic scaling is determined by the musical mode, before discussing the steps needed for identifying the interval classes in the analyzed piece of music, it is necessary to define a musical mode. Here is the definition that sums the conventions of musicological literature in English (Powers & Wiering 2001) and Russian languages (Kholopov 1976):

- A musical mode is a system of few *concordant* tones selected for music-making based on their melodic interaction in a *specific type of melody that is conventional for a chosen expression*—a set that defines the *normative frequency values*, the *rules* for their succession and subordination, and the *semantic range* of possible applications for this mode in the construction and in the perception of melody and harmony.

In a nutshell, in every music culture, a group of music users adopt a set of conventions, according to which they judge which tones are suitable for joining together, and which tones are poorly compatible and not suitable for expression of a particular idea. Musical mode can be viewed as a model of configuring all the pitch-related AEs (melody, harmony, texture, register, music form) in order to effectively encode and decode information that is important to music users.

On part of the researcher, modal scaling involves taking notice of the pitch level of each tone used in music, in its respective register within the entire ambitus of pitches available to a performer, and identifying the intervallic typology followed by that performer. In practice, this means deciding which tones in a musical composition constitute variants of the same pitch class. This requires answering the following 3 questions:

1. which tones present *accidental deviations* in tuning of the same modal “degree” that is called forth to stress an important modal intonation (e.g., slight sharpening of “C” to facilitate its melodic motion up to “D” in a mode C↑-D-E-G), thereby supporting the irregular use of that degree (it can spontaneously go sharper or flatter at any point);
2. which tones present *regular alterations* (sharp/flat) of the same modal degree (e.g., a special pitch-class of “C#” can constitute a temporary modification of the pitch class “C” to generate an alternative modal “leading tone” to D in the mode C(C#)-D-E-G), thereby supporting the use of multiple versions of such a degree, usually bound by two strict rules—one for an ascending and another for a descending melodic motion;
3. which tones constitute an *autonomous chromatic degree*—this can be tricky in a chromatic or micro-chromatic mode (i.e., “C#” can be the II degree in a chromatic mode C-C#-D-E-G)—thereby supporting the strict use of a single degree bound by a single rule for each instance of ascending and/or descending motion.

A researcher has to evaluate the melodic patterns within a piece of music in order to establish those morphological features that are principal for its TO. The computer software significantly facilitates this task, but still requires manual input. Mean values of the acoustic measurements should be related to the repeated pitch and rhythmic patterns and correlated with the intervallic distances between the neighboring tones. Modal scaling differs from the orthodox idea of creating a diatonic “gamut” scale by constructing the circle of 5^ths^ and inverting its every other 5^th^ an octave down. Instead, modal scaling establishes a pitch class in reference to a particular registral position, *not limited by octave equivalence*—to be able to identify non-octave-equivalent modes (e.g., B-C-D-E-F-G-A-Bb), which are quite common in music practice, especially in orally transmitted music (see Appendix-II “Non-octave hypermode” in Nikolsky 2016). Its degrees are register specific: whenever the ambitus exceeds the octave in such music, the upper intervals differ from the lower ones (Fernando 2007).

In conjunction with the intervallic typology, modal scaling defines the interval set of a music work. Thus, for the music conceived within the micro-chromatic typology, such as Pamir falak, the chromatic tones constitute different degrees, whereas for diatonic modes the very same chromatic tones would make alterations of diatonic degrees (see Demonstration-1 “Chromaticism” in Nikolsky 2016). In order to be able to identify intervallic typology, it is imperative to define the **musical objects** in a music work—i.e., to find out which exact acoustic constants constitute elements in TO (Nazaikinsky 1973). The starting point in this investigation belongs to the expressive aspect of articulation: the analyst has to establish which tones are interconnected, and which are disconnected—and to grade the extent of their connectedness and disconnectedness.

#### Articulation grading

**Articulation** styles, implemented in music (Chew 2001), can be measured by identifying the distribution of syllables in vocal musicking (which syllables are interconnected into words vs. which are separated by words and phrases) and the distribution of legato (connected tones) versus staccato (as well as other styles of disconnected articulation, such as non-legato, marcato or tenuto) in instrumental music. As a general prosodic rule, singing multi-syllabic words engages legato articulation. Exceptions are rather few and limited, such as not finishing a long sung-out word at the end of a stanza that is to be continued, or interrupting a word in half-stanza with a caesura in order to repeat the interrupted syllable and, this time, to finalize it in the beginning of the following stanza (Alekseyev 1976, 132). Such devices are usually reserved for special expressive purposes to mark specific melodic elements (e.g., caesura) or to maintain a selected rhyming scheme in a particular spot of the lyrics, and do not contribute statistically to the definition of the pitch classes within a song.

Staccato articulation is rarely used for the entirety of a vocalization, unless it delivers a special effect (e.g., illustration of shivering in Purcell’s “Aria of the Cold Genius” from Act III of the opera “King Arthur”). However, staccato is commonly used to contrast legato within melodic motifs, where smoothness is undesired (this is a commonplace in dances where music has to support physical motion even in situations where this motion is not smooth but abrupt). Intermittent staccato is often associated with the expression of gaiety, energy, activity, fear, or anger. Legato-staccato opposition often supports the distribution of steps (legato) versus leaps (staccato) to energize the latter. Such distribution might assign specific degrees in specific registers exclusively to the staccato function. Also, it is worth noting that staccato articulation directly affects musical intonation by excluding the portamento sliding in and out of a target pitch, thereby confining the music to a concise discrete (pointillistic) intervallic typology. That is why, staccato is seldom in ekmelic music.

A few other alternative styles of articulation should be discussed here. *Non-legato* style should be distinguished from staccato by a certain “naturalness” (casualness) in separation of tones—not displaying a deliberate effort to cut a tone shorter. The decay phase of a vocal non-legato tone appears light, easy, relaxed, without the strain associated with the bouncing impression from the salient staccato. The extent of the distinction between staccato and non-legato depends on the perception of beat: staccato tones are shorter than the beat or a binary fraction of the beat (½ or ¼), whereas non-legato tones are about equal to the beat. Non-legato melodic motion usually comfortably “walks” together with the beat, without appearing to “bounce,” “hop” or “startle,” all of which characterize staccato melodies.

For TO, non-legato is useful in revealing the gravitational relations between tones of the melody: pairs of unstable and stable tones usually require legato connection, making non-legato indicative of the absence of subordination between the adjacent non-legato tones. In a melody that is not committed exclusively to just one articulation style, *legato* is likely to reflect *subordination* of an unstable tone to a neighboring anchor tone, which often forms the nucleus of a melodic motif, extrapolating the legato articulation to the entire motif. *Non-legato*, on the other hand, more often than not, reflects the *coordination* between the equally important adjacent tones—when they belong to the same class within the tonal hierarchy (e.g., the function of a tonic triad for I, III and V degrees) or at the complete absence of *hierarchic* organization, when all tones are united by *heterarchic* relations (e.g., the whole-tone mode).

*Marcato* style can also be important for TO. Marcato (from Italian "marcare"—"to mark") implies a forceful attack at the onset of a tone, which usually emphasizes that tone’s frequency value. The attack phase of a marcato tone should appear amplified as compared to its sustained phase. Marcato is a *dynamic* articulation: it articulates the flow of music not only by shortening the duration of a tone (for a little less than non-legato), but also by increasing its amplitude. Marcato melodic motion resembles a stamping gait, where each foot is hit against the ground with excessive force. This should explain the association of marcato with the expression of intensity and strength. For TO, marcato is important because it is likely to fall on the anchor tones (e.g., the opening of “This Old Man”). Or, if it falls on the unstable degrees of a mode, it changes the melodic function of that degree (more on that below) by charging it with a greater stability so that it can stand up against the stable degrees in a melodic opposition (e.g., the opening of “Les Moissonneurs” by Francois Couperin).

Accounting for articulation styles allows the analyst to establish the way in which the creator of a melody intended its tones to be connected or disconnected. Psychoacoustic research has established that in order for a person to hear two consecutive tones as connected, they have to be separated by no more than one second. Actually, the breaking point seems to be around 800 msec, but our ear compensates for the break by extending the "echo" of the tone last heard (Huron 2001). This is how our ear deals with the melodic phrases that are performed with staccato articulation. The longer is the gap between two sounds, the less helpful is the echoic memory—and the more separated both tones appear to be.

However, temporal continuity (articulations of legato and legatissimo) is by far the default method for melody making. David Huron reports that statistic analysis of vocal melodies reveals 93% of all melodic tones to immediately follow one another. For instrumental melodies this percentage is even higher—98% (Kubovy & Howard 1976). So, not *any* pause in a spectrogram is to be taken as a sign of a break between intonations. In case of staccato style, the relative size of a gap should be estimated along with other factors: patterns of syllabling (e.g., rhyme), of pitch change (e.g., repeat), register (lower registers require longer breaks), and timbral similarity of tones within the same melodic intonation.

Each syllable of speech usually serves as a unit of tonal value, commonly resolved into hearing a particular pitch even in verbal patterns of non-tonal languages (Patel 2006). The same power that binds syllables into a word usually binds pitches of each of the syllables into a musical motif (even in strict staccato singing). Such grouping effect constitutes a rule, breaking of which usually has to do with a special effect of deceiving the listener’s expectations (e.g., appropriate as a climax in a melodic formula, sentence, or phrase). The importance of this rule is that the pitch levels used for different syllables are going to be more tonally autonomous than the pitch changes while singing out a single syllable. In the latter case, the change in frequency could constitute:

1. alternation of degrees (e.g., I-II-I);
2. melodic embellishment (e.g., a shake, a trill or a grace-note) of a single degree—differing from 1) by assigning a shorter time value and softer dynamics to the changed pitch;
3. timbral embellishment (e.g., vibrato or tremolo)—differing from 2) by retaining the same timbral coloration over the principal and the embellishing pitches.

Pitch changes *within* a syllable present the greatest measure of tonal integration into a group, and therefore produce the weakest articulation grading—each of the frequency values here are the lowest in their discreteness, except the frequency that marks the onset of the syllable. This opening frequency becomes perceptually salient and receives a higher grading in articulation as compared to the frequencies that follow the onset of that syllable.

Similarly, the pitch value of a musical tone that initiates a word should be taken as more important than that of other syllables. The beginning of a word attracts attention to the corresponding pitch value. Thereby, the distinction between different tones within the same syllable presents the first level of hierarchy. The second level is comprised of syllables within the same word. The third level relates different words within a phrase of the lyrics. The pitch value of a musical tone that initiates a verbal phrase usually bears modal importance by marking an anchor degree. Of similar importance is the pitch value of the first syllable in a word that terminates the phrase. It usually is either most stable or most unstable in a musical mode.

Altogether, articulation supports up to 5 levels of hierarchy (Table-1):

**Table-1**. Tonal hierarchy based on syllabic distribution of pitch changes, graded for each of the hierarchic levels for the modal analysis of music. The hierarchic levels are defined in relation to their musical and verbal structural functions—each receiving a corresponding number of Articulation Rating grades (AR), from the lowest 1 grade for 1^st^ hierarchic level, to the highest 5 grades for 5^th^ level. The column “Mark” shows the symbols used for displaying AR in the graphic representation of melody.

| **Level** | **Pitch change type** | **Mark** | **Music’s structural function** | **Speech’s structural function** | **AR** |
| --- | --- | --- | --- | --- | --- |
| 1 | **Melismatic**:  Frequency change within a single sung syllable. | **(…)** | Embellishing a syllabic musical intonation by inserting a fluctuation in it or adding a brand-new intonation in legato style to a syllabic intonation. | Breaking a syllable into constituent phonemes, based on vowel-consonant contrast, & stressing a vowel & its phonetic symbolism. | 1 |
| 2 | **Mono-syllabic**: Frequency change between 2 syllables of the same sung word. | **-** | Emphasizing a musical intonation by stressing each of its constituent tones by means of phonetic contrasts. | Breaking a word into constituent syllables, as in hyphenation, emphasizing word’s articulation. | 2 |
| 3 | **Lexic**: Frequency change between 2 adjacent words of the same sung phrase. ^[[4]](#footnote-4)^ | **,** | Marking a motif by stressing the pitch change and the musical intonation at the head of a word. | Breaking a phrase into constituent words, and setting one as principal in relation to the other(s). | 3 |
| 4 | **Phrasal cadential**:  Frequency value of the last syllable in a phrase. | **.** | Marking the cadential modal tone at the end of a musical phrase to show its modal stability or instability. | Segmenting a streak of words into phrases by introducing some contrast at the phrasal end. | 4 |
| 5 | **Phrasal initiating**:  Frequency value of the initial syllable in a phrase. | **!** | Marking the initial modal tone of a musical phrase to show its stability as a reference for the entire phrase. | Segmenting a streak of words into phrases by introducing some contrast at the head of a phrase. | 5 |

The frequency change within a syllable (i.e., a “melisma”—a group of tones sung out to one syllable of lyrics) constitutes a *sub-elementary* level in the tonal hierarchy. This change of frequency is the least powerful in defining a pitch class: its ornamental functionality makes the frequency change momentary in auxiliary (A-B-A) or passing (A-B-C) melodic motion of little importance for parsing the melodic contour into specific pitch values. Such ornamental melodic motion is common for production of embellishments (especially, shakes), swift passages (tirata or cascata) and slight thematic variation in repetitive use of the same thematic material. Consistent melismatic singing produces the impression of emphasizing the *legato* articulation. An embellishing tone is often under-stressed, presenting an approximate (indiscrete) inflection of a principal tone (an embellished tone) that opens the embellished intonation and therefore becomes salient. A very long melisma can contain a number of thematically important musical intonations, merged within a single syllable. This generates yet another implementation of the legato style, where multiple intonations are fused into a single “vocal run.”

Mono-syllabic musical intonation discloses the *elementary* level of tonal hierarchy. Mono-syllabic tones stand out as clear-cut, most likely representing pitch classes that are modally significant (e.g., principal degrees of a musical mode). Syllabic singing generally produces the impression of *marcato* (dynamically accentuated) or *sostenuto* (sustained to the full rhythmic value to perfectly fit the metric grid) articulation styles. In both cases, pitch changes become rhythmically emphasized. The pitch values of each of the syllables usually present discrete modal degrees, which can be “natural” or “altered” (sharpened or flattened).

The frequency change between words is usually responsible for forming the surface level for musical phrasing by generating motifs and selectively stressing one against the others. This can be regarded as a *compound* level of TO. The more syllables in a word, the stronger its motivic power, especially if that word is semantically significant in a phrase. Stressing specific motifs usually makes certain degrees in a mode more important than others. Motivic segmentation of a melody often places the *staccato* articulation (shortening of the actual duration of a tone while retaining its metric value, so that the succeeding tone becomes separated by a tiny gap) on the last tone in a motif that corresponds to the last phoneme of a word. Such staccato lightening of the last tone makes the latter less tonally important compared to the tone that opens a word.

The frequency value of the last word in a phrase usually marks a melodic cadence, acquiring the highest order tonal status. Perfect and imperfect cadences sum up a phrase or a sentence by indicating its tonal function (e.g., “affirmative” function of the consequent versus “interrogatory” function of the antecedent sentence). Cadential pitches often engage “characteristic modal degree(s)”—those tones whose relations to other degrees are unique to a given mode and therefore can prompt recognition of that mode upon hearing it (Grigoryev 1981)^[[5]](#footnote-5)^. Hence, cadences usually reveal stability or instability of a phrase by terminating it with a stable or unstable modal degree. Cadences generate *higher order compound* structures between musical phrases. Unlike the optional staccato endings of a word-motif, cadential tone nearly always receives a stress of the *sostenuto* articulation, followed by a well-marked pause.

Emphasizing the frequency value of the first word in a phrase often defines the stability axis for the entire mode, initiating the melodic motion from its most stable or second most stable modal degree. Especially for the opening phrase in a song, its initial tone establishes the frame of tonal reference, and therefore somehow marks the “tonic.” Certainly, there are other possibilities, but it is important to keep this one in mind. This capacity gives the initial tone the upper hand over the cadential tone in TO—a rule inferred by Alekseyev in relation to music composed in indefinite intervallic typologies (Alekseyev 1976, 138). Perhaps, it has to do with the increased malleability of morphological units (motifs, phrases) in such music—similar to languages with fusional morphology, like Greek and Russian, where dependence on morphological headedness is a central component in the accentuation of the prosodic system: “a morphological head becomes a prosodic head, provided that it is marked” (Revithiadou 1999 p. 20).

Initiation and cadential tones often mark the “tonic” (the most stable modal degree) and “dominant” (an anchor that is alternative to tonic and generally less stable). “Anchor” should be distinguished from “tonic” in modal theory (Asafyev 1971 p. 201). “Tonicity” is a particular form of “anchoring” a frequency value—characterized by hierarchic subordination of many tones to one tone and manifested in the sense of completion of a motif or phrase that end on tonic. As such, tonicity is atypical for pretonal forms of TO, where tones of the melody usually have coordinative, rather than subordinative, relations.

Alekseyev (1976, 37) distinguishes between 3 levels of anchoring,^[[6]](#footnote-6)^ commonly employed in modal music.

1) “*Leaning* tone” (“opornyi ton”) relies on the anchoring function of an entire registral zone—any tone that falls within its range turns into a melodic anchor—according to Alekseyev, this is the earliest ontogenetic and phylogenetic form of anchoring;

2) *“Stable* tone” (“ustoi”) assigns an anchoring function to a narrower pitch zone defined in contradistinction to the adjacent registral pitch zones that are recognized as *unstable*—this form of anchoring evolutionarily succeeds that of “leaning”;

3) *“Tonic* tone” (“tonika”) assigns an anchoring function to a specific pitch value (usually single, but sometimes 2 alternating anchors) in a mode.

It should be underlined that melismas, in general, weaken articulation. Frequent melismas make music sound inarticulate and excessively gradual. This fact has been known for centuries in such genres of Western music where melismatic singing has become a trademark (e.g., Halelluja). The longer the melisma, the more fluent the flow. For this reason, the arithmetic of articulatory grading cannot be all positive. If melisma falls on the syllable that terminates a word or a phrase of the lyrics, then, the grading for that tone has to be reduced by 1 grade (to receive 2 grades for the melismatic end of a word, and 3 grades for the end of a phrase). This reduction only affects the endings—and not the beginnings of phrases—since at the onset of a syllable it is impossible to predict whether it will be treated melismatically or syllabically, whereas the onset of a phrase receives its emphasis regardless of whether or not it is followed by melisma.

It does not matter if the song that has lyrics is built on the repetition of a single word or a personal name, or whether it uses vocables—a set of meaningless syllables applied as a pattern on musical pitches. Vocables still provide basic articulation by marking beginnings of the syllables and their groups—in case of contrasting syllables and repetitions of the same syllable. The only thing missing in a vocable-based song is a higher order syllabic correspondence. For a song in a foreign language it is crucial to obtain emic information from a native speaker as to what the margins of words, phrases, and sentences in the lyrics are. Instrumental melodies should be treated like emulations of vocable-based vocal melodies (i.e., akin singing an instrumental melody on arbitrary solfa syllables, like “la-la-la”).

#### Breathing gradations

Breathing is rarely discussed in relation to the analysis of music form, yet it constitutes a powerful articulation tool—even more so in musical than in verbal vocalizations, since the former relies much heavier on the fine control of air pressure in sustaining the pitch levels. Alekseyev underlines that the modal analysis of non-diatonic music should start from the definition of the breath cycle in music (Alekseyev 1976, 133). Taking a breath interrupts the flow of music and marks the phrasal boundaries. The deeper the breath (usually expressed by the increase in duration of a pause), the stronger the phrasal emphasis, quite indicative of the prosodic complexity in speech production (Krivokapic 2012). For music, too, the longer the pause, the greater importance is assigned to the starting tone of a new phrase.

The breath cycle imposes the “clausal structure” that governs parsing of both, music and speech flows, marking meaningful verbal and musical intonations (Fenk-Oczlon & Fenk 2009). The “breath groups” of sounds are physiologically marked by the breath cycle, creating phraseological units—and this might be regarded as a linguistic universal (Lieberman 1966). The formative influence of breathing on phrasal grouping and intonation is pronounced in language acquisition during early childhood (Snow 2006), as well as in morphological organization of indigenous music that uses pre-tonal forms of TO (Alekseyev 1976, 130). Asafyev regarded breathing as a determinant factor in generating phrases in, both, speech and music—including instrumental and even dance music (Asafyev 1965 p. 20)—due to *the power of respiration to bind physical motions that occur during the breath cycle into motoric compounds equivalent to phraseological units*.

- Most commonly, breath is taken between two adjacent verbal phrases in lyrics. In our articulation grading system, this position will amount to 6 points assigned to the pitch value of a word that initiates a new phrase.

It is quite common to take a breath between two words, in which case the tone after the breath will receive 4 points. Occasionally, a breath can be inserted between the syllables of the same word (producing 3 points), or occasionally even inside a melisma (2 points). The cadential tone in a musical phrase is rarely, if ever, separated by a breath, but should it happen, it should receive 5 points. Just as a reminder, the breath not always accommodates the cadence. Alekseyev reports cases where the unstable tone that terminates the cadence is immediately followed by some new material, without any break. This usually occurs in situations where the functions of stability and instability of particular degrees are very clear to the performer, so that she/he perceives the idea of stressing them by a full stop as unnecessary redundancy, preferring instead to pursue greater animation and coherence of the music (Alekseyev 1976, 172). In a situation of such “swallowed” cadential ending, the rating value assigned by syllabification should be decreased by 1 (5-1=4)—to reflect the fact that the normative emphasis on the beginning of a new musical thought is removed in sake of a greater dramatic effect of a “run-in” sentence.

Moreover, *the duration of “breath groups” has a formative influence on the metric organization of music, even at the absence of a distinct beat*. The frequency of occurrences of the breathing pauses generates its own expressive aspect of music—a **breathing pulsation**. This pulsation usually becomes synchronized with the metric pulsation through the entrainment mechanism, which most probably also involves emotional contagion (Etzel *et al.* 2006). After a while, a music performer starts breathing in a cycle that coincides or stands in multiple ratio to the downbeat of the music. If the music does not provide a clear regular stress, breathing pulsation becomes the primary means of breaking the music flow into “iso-metric” units. Nils Wallin explains that a spontaneous respiratory cycle triggers impulses in the muscles that are superimposed on their tonic activity, and when coupled with the oscillatory curve of respiration, they cause regular shifting between phases. The rhythmic coordination between breathing and muscle contraction corresponds to “the economical principle of the greatest cooperation possible through the greatest possible simplicity” (Wallin 1983).

Therefore, it is imperative for any modal analysis to take note of any breathing places—caesuras—and estimate their duration in relation to the duration of the breathing cycles. For this reason I will mark the breathing with a checkmark at the onset of caesura. In a longer piece of music, the size of a checkmark will indicate the relative duration of a pause for taking a breath. There is experimental evidence that the pause duration is determined by the phrase length and prosodic complexity of the conceived phrase as a measure of its prosodic planning (Krivokapic 2012).

Making a distinction between short and long caesuras helps to reveal the underlying hierarchic organization in the breathing cycle. Just as metric grid can have a few pulses running in parallel at different hierarchic levels such as beat pulse at the basic level combined with the downbeat pulse at the higher order level (Rothstein 1989), the breathing pulsation can too be complex. It can be organized in reference to two levels of basic (every cycle) as well as higher order hyper-pulse (every other cycle). This is analogous to the relation between the *metric* and *hyper-metric* pulsations in music (Temperley 2008). The longer is the music, the more likely it is for the breathing and the metric pulses to be complex, since dense information benefits from chunking, and higher order cycles present convenient markers for chunking up data.

The breathing cycle can also feature a particular style. Regularity of the breathing cycle is likely to correspond to the expression of a musical character that features little tension. Irregularity of the breathing cycle is an indicator of increase in tension, which can be temporary (at a climax point) or on-going within an entire work of dramatic or tragic genre (e.g., Pamir falak).

Luckily, taking a breath is quite visible in the spectrogram (intense breathing introduces stochastic “smears” in the mid frequency range)—which comes handy in case, for some reason, breathing is not audible. For TO, regularity of breathing increases the likelihood for the initial and/or the cadential tones in a musical phrase to mark anchoring degrees. Regular breathing also characterizes formulaic melodic structure. Irregularity of breathing, in contrary, often accompanies a greater functional diversity of the melody, promoting melodic elaboration.

#### Melodic directionality and modal inclinations

Closely related to breathing is yet another expressive aspect of melody—its **directionality**. Musical phrases are tonally shaped by their melodic direction. Alekseyev considers ascending and descending directions to constitute the primordial modal “*inclinations”* [Rus., “nakloneniye”]*—*the prototypes of “major” and “minor” of diatonic music systems—which in pre-diatonic music acquire tonal functions of respectively contraction/activation versus relaxation/passivity (Alekseyev 1976, 143).

The notion of *modal inclination*, elaborated by Russian modal music theory, is cardinal for TO and deserves explanation due to scarcity of information about it. Modal inclination takes its origin from the Ancient Greek concept of melodic “genus” that distinguished between 3 basic genera of disposition of tones: diatonic, chromatic, and enharmonic—each implementing its own method of distribution of intervallic distances, and each associated with expression of a dedicated range of semantic values (West 1992). From the modern musicological perspective, Ancient Greek genera presented 3 different intervallic typologies, each allowing for the construction of the repertory of modes, where every mode could be implemented in 3 ways (e.g., diatonic Dorian, chromatic Dorian, and enharmonic Dorian) following the same modal scheme (for Ancient Greek Dorian, this was the smallest interval placed at the bottom of a tetrachord, and the larger interval—at the top), but scaled differently depending on the choice of intervallic typology (semitone/tone in the diatonic, semitone/tone-and-a-half in the chromatic, and quarter-tone/tone-and-three-quarter-tones in the enharmonic genera). Here, the relation between intervallic typology and mode can be thought of in terms of electronic signal generation, where a signal generator produces a continuous wave signal that can be modulated by amplitude, frequency, phase, or pulse. Just as a wave can be sine or square and can be modulated by amplitude or frequency, the genus can be diatonic or chromatic and can be used to generate, say, Dorian or Phrygian mode.

In the process of historic development towards the 2^nd^ century AD, Ancient Greek genera became reduced to a single diatonic variety which received a new implementation in the Christian Medieval 8-mode system. As a result of genesis of the triadic harmony during the 15–16^th^ century (Nikolsky 2016), the concept of major and minor triad was formed: “major” was associated with greater stability and happiness, and “minor”—with lesser stability and sadness (Wienpahl 1959). Under the influence of major/minor semantic dichotomy, by the 17^th^ century Western modal theory elaborated a new meaning for the old concept of genus: the modes inherited from the Medieval music theory came to be regarded as “genera,” where major and minor triads that were formed at the most stable tone of a mode (tonic) were treated as “generic categories” (Barsky 2014 p. 40).

Contrasts, generated by modulating from major to minor tonic between a pair of the related keys (e.g., parallel or relative), prompted German theorists of the early 17^th^ century to conceptualize major and minor modes (Lester 1977). This led to the introduction of the term Tongeschlecht (literally, "Tone gender")—translated into English from the original German in Riemann’s influential music dictionary as “mode,” or “tonal genus,” or “Clang genus” (Riemann 1896). Surprisingly, English musicology has not established its own term to refer to the distinction between the intervallic classes of major and minor that are used to generate keys. The term “tonal genus” did not earn recognition, and the term “mode” has become extremely confusing in this context, since it has been already employed in reference to the subclass of a subclass of Tongeschlecht  (e.g., the harmonic mode of C Minor key, C-D-Eb-F-G-Ab-B, as opposed to the harmonic mode of C Major key, C-D-E-F-G-Ab-B).

German theorists have generalized the Tongeschlecht distinction into a dualistic theory, with a pronounced Hegelian flavor, as evident in the work of Moritz Hauptman “The Nature of Harmony and Metre” (1853) that opposed the active “das Haben” of Major to the passive “das Sein” of Minor to draw the axis of interaction between the ascending force of major and the descending gravity of minor (Hauptmann 1888). German theories kept capitalizing on the “tone gender” interpretation of the distinction between major and minor, stressing its biological connotations: “major” as an embodiment of masculine, active, and positive qualities; and “minor” as an embodiment of feminine, passive, and negative qualities (Rothfarb, 1979). Ernst Kurth further advanced the notion of Tongeschlecht to fit into his “dynamic model” of harmony and melody. Kurth regarded not only the chordal structure, but the *scale* of a major key to embed the trajectory toward the apex with its bi-tetrachordal structure—embodying the inherently active idea of ascension toward the upper tonic (Kurth 1931 pp. 205–6). Likewise, he considered the minor scale to embed conflicting dynamic impetuses, where the upper tetrachord was pushing up when ascending, and collapsing when descending (melodic minor), while the lower tetrachord led down, which indicated its melodic passivity (p.208–9).

Like other ideas of Kurth that inspired Russian modal theory, melodic Tongeschlecht also found reflection in Asafyev’s theory. It was Asafyev who suggested to use the term “inclination” (“nakloneniye”) in reference to Tongeschlecht (Asafyev 1971 p. 230). In Russian grammar, this word is reserved to distinguish an *active voice* sentence from a *passive* *voice*, so Asafyev’s proposition very closely followed Hauptman’s direction. What was new was that the term “inclination” implied the *tendency of leaning toward something* (“naklon” means “incline”). Asafyev considered a musical mode to constitute a unity that integrates all tones in a melody, and due to the processual nature of melodic motion, it is something that is to be experienced as some melodic model that is coming-into-being. Hence, “major” and “minor” present only “episodes” in the melodic motion that normally keeps oscillating between the two, so that upon hearing the entirety of a musical work we estimate the overall “majorness” or “minorness” of certain transitions in it. The terms “majorness” and “minorness” (Rus., “mazhornost” and “minornost”) as well as the idea of their opposition were introduced by Aleksei Ogolevets, a distinguished Russian acoustician and theorist from Moscow Conservatory, who developed a methodology to estimate the index of majorness and minorness of a particular mode based on the distribution of its tones by the circle of 5th in relation to its tonic (Ogolevets 1941).

Another development of the inclination theory proceeded towards investigating whether the major/minor distinction was applicable to traditional folk music. Alexander Nikolsky (1926) pioneered this direction of research. He overviewed and analyzed a bulk of Russian folk songs that used unconventional tonal organization, especially “mesotonal” modes—modes with 5-6 degrees (Starostina 1973). Nikolsky concluded that the modes of folk music used their own proprietary inclinations, different from both, major/minor of Western tonality, and from the medieval Church modes. Nikolsky qualified folk inclinations as “quartal” (based on the interval of 4^th^) or “quintal” (5^th^). Feodosii Rubtsov followed Nikolsky’s lead and established the line of historic development in Russian folk song: starting from what he called “neutral” inclination (instead of quartal and quintal inclinations that Rubtsov had rejected) and eventually forming the proprietary folk “major” and “minor” inclinations under the influence of Western classical music (Rubtsov 1964).

The wide range of semantic applications of major and minor modes in practice of classical music, when a sad funeral march could be written in a major key (e.g., Handel – famous march from “Samson and Dalila”), whereas a proud national anthem in a minor key (e.g., Israeli “Hatikvah”), prompted the Russian modal theorists to conceptualize the modal inclinations as musical equivalents of grammatical gender (Mazel 1972 pp. 109–10). In Russian, a noun could be feminine, yet not necessarily denoting submissiveness, and masculine, not denoting aggressiveness, while there is a neuter gender. Regarded as a musical grammatical form, modal inclination has greater leeway in reflecting the existence of general semantic tendencies while allowing for semantic exceptions. This capacity of modal inclination makes it invaluable for analysis of music structures.

The truth of the matter is that, both, major and minor, can be found across different intervallic typologies. Major can be chromatic (C-C#-D-D#-E-F-F#-G-Ab-A-Bb-B-C), diatonic heptatonic (C-D-E-F-G-A-B-C), anhemitonic pentatonic (C-D-E-G-A), hemiolic (C-Db-E-F-G-Ab-B-C), non-octave hypermodal (C-D-E-F-G-A-Bb-C-D-Eb-F-G-Ab), mesotonal (C-E-F#-G-A), oligotonal (C-D-E-G), or it can even color the ekmelic modes, evident in some examples of cultural borrowing reported by Alekseyev (Alekseyev 1986, 148). In all such cases, major inclination usually makes the music appear more optimistic than minor inclination. The term “inclination” is very valuable in pointing out such cross-typological similarities.

Of course, modern psychology has the term “valence” which is employed in reference to semantic distinctions between major and minor. However, this term is too general and has clear individualistic application (two listeners might report different valence in their evaluation of the same auditioned sample of music). Musicological analysis cannot rely on subjective estimations—it has to reveal the objective features of musical arrangement. It is exactly the term “inclination” that captures the structural features of music: prominence of tonic major 3^rd^ for major inclination, and minor 3^rd^ for minor. Another limitation of the conventional major/minor valence approach, even in relation to diatonic music, is its failure to account for the neutral inclination in those cases where music displays neither salient tonic major 3^rd^ nor tonic minor 3^rd^ (e.g., in the pentatonic mode of C-D-F-G-A, where C is tonic).

Yet another inclination often found in diatonic music is the ***diminished*** inclination, produced by melodies that lean on the tones of the diminished triad (e.g., B-D-F). There is experimental evidence that non-musicians distinguish between minor, major, and diminished diatonic modes upon hearing an unfamiliar melody in one of these modes—with quite pronounced bundling of 3 major and 3 minor modes versus one diminished (Locrian) mode (Ramos *et al.* 2011). The tritone that is most prominent in diminished triad has been experimentally confirmed to be perceived in a way distinct from other intervals—associated with danger and violence (Smith & Williams 1999). Music in Locrian mode differs from minor by inducing the darkest sensations in the listeners, and to suggest associations with anger rather than sadness, as evident in the listeners’ interviews combined with the measures of EEG frontal activity (Trochidis & Bigand 2013). Another study found that Locrian mode was more strongly associated with loathing, boredom, and disgust than terror, fear, and apprehension, attributing this to the lack of melodic resolution, imposed by the “tonic” diminished triad (Straehley & Loebach 2014).

Like major and minor, diminished inclination can be found across different intervallic typologies: diatonic (Locrian mode B-C-D-E-F-G-A-B, popular in South Balkans), chromatic (two octatonic modes, tone-semitone B-C#-D-E-F-G-Ab-Bb and semitone-tone B-C-D-Eb-F-Gb-Ab-A), mesotonal (Istrian mode B-C-D-Eb-F-Gb), non-octave hypermodal (Russian “ukosnyonnyi obikhod” B-C-D-E-F-G-A-Bb-C). Of them all, the latter received the strongest theoretic foundation, being accepted by the Russian Orthodox Church as part of the obikhod system in 1772 (Kholopov 2004). Obikhod also has made a deep imprint on Russian folk music from the 15–16^th^ centuries onward (Rudneva 1994 p. 121). The underlying reason for this could be that common gender divisions in Russian language include masculine, feminine, and neuter, that find close correspondence to major, minor, and diminished inclinations of Russian folk music (Nikolsky, 2016, Appendix-2). The music written in diminished modes is usually perceived as bearing more tension than those written in minor. Diminished modes are often employed by composers to express anxiety, uncertainty, or some conflicting emotional state.

A case for the ***augmented*** inclination could be made. Thus, in traditional folk music, there is an archaic Setu mode, found in Estonia (Ambrazevičius and Pärtlas 2011) and Siberia (Kalkun & Oras 2014), which features the mode with prominent two augmented triads (e.g., C-E-G# and Db-F-A in the scale of C-Db-E-F-G#-A) for arrangement of 2-part singing. Another source for tonic augmented triad is the whole-tone scale that has been explored by Rossini, Schubert, Berlioz, Glinka, Borodin, and Dargomyzhsky in fragments of their large compositions within the framework of classical harmony. Toward the end of the 19^th^ century, such experiments resulted in the emergence of a new modal style alternative to traditional functional harmony where the entirety of a composition could be sustained strictly in a whole-tone mode (Rebikov, Debussy, Bartok, Messiaen). Despite the relative rarity of such music, it is easily recognizable and is associated with expression of magic and strangeness—thereby clearly differing from major, minor, and diminished-based music.

Alekseyev should be credited with the expansion of the concept of inclination onto music systems that use indefinite intervallic typology. Absence of the notion of “chord” and “thirdness” prevents such systems from adhering to major/minor typologies in a strict sense, so that their presence is either an illusion to a foreign listener whose ear is accustomed to diatonic music, or, an accidental byproduct, unintended by the indigenous performer (Alekseyev 1976:144). Shifts in directionality in reference to the breathing cycle execute the primary formative role in music based on indefinite intervals. And here the opposition of ascending and descending melodic contours effectively replaces the opposition of major and minor of definite pitch music (i.e., “emmelic” intervallic typology). In the same way that leaning on the tones of a major triad makes the melody sound active, whether it is created in chromatic, diatonic, or pentatonic system, leaning on the anchored initial tone of the ascending contour and on the climactic tone (the most unstable) produces an active impression, no matter which exact intervallic distances separate the tones. Therefore, Alekseyev’s inclinations are essentially *directional*—as opposed to *chordal* inclinations of major and minor keys of Western tonality.

What Alekseyev has generalized from the comparative analysis of thousands of songs of Yakuts and neighboring indigenous ethnicities, is confirmed by the researchers of the acquisition of verbal prosody. Thus, David Snow’s analysis supports Philip Lieberman’s claim (Lieberman 1966) in that rising and falling intonations differ in frequency and magnitude because they have different physiological bases. Declining subglottal pressure, induced by relaxation of the thoracic and intercostal muscles during expiration, characterizes the falling intonation. Rising intonation is more effortful to produce and therefore is associated with activation and excitement (Snow 2006). This distinction is very important for production of vocal music. Speech does not require such fine voluntary breathing control as music needs: normally, speakers breath twice more frequently than singers, engaging only a fraction of their lungs’ capacity, whereas singers spend nearly all of their lungs’ capacity and engage all their major respiratory muscles (Fitch 2006). Greater effort, demanded by the ascending inclination, contrasts the comfort of the descending inclination much more so than the corresponding prosodic typology of spoken verbal phrases.

Physical strain or its absence imposes structural differences on musical phrases. Ascending inclination is likely to lead to an unstable tone—in contrary to descending intonation that is more suited to lead to a stable tone. This opposition becomes most important for intonations that feature a change in direction, where the tone around which the melody turns becomes well defined. This is what Narmour calls “closure” (Narmour 1992). Even ekmelic singers, who do not observe the octave equivalence, intervallic incrementality, and conservation of intervals, do mark the pitch values of the transitional points in the indiscrete pitch contour (Alekseyev 1976, 134). Hence, switches in direction are crucial for identifying degrees in music that is indefinite in pitch.

*Descending inclination should be regarded as the basic “default” melodic contour*. Just as it is evident in ontogeny, where falling intonations are known to be the first to develop during the first year of life (Snow 2006), cultivation of ascending inclination in phylogeny usually follows mastering of descending inclination (Alekseyev 1976, 133). Correspondence of the expiration cycle to instinctive relaxation of the vocal apparatus during vocalization of the falling contour, makes the latter feel “natural”—in contrast to the challenges of coordinating the increase in the work load and the air expenditure that are needed for production of the ascending inclination at the end of the expiration cycle. It is this contradiction between the reducing oxygen and the needed effort that should be held responsible for the association of the ascending inclination with will power, forward directedness, and enthusiasm—as well as for its greater sophistication in comparison with the descending inclination. Already in Antiquity, Pseudo-Aristotle (Aristotle & Mayhew 2011) noticed that music sounded especially "good" in a descending as opposed to an ascending direction (Problem No.33 from Book 19). And one of the chief specialists on Ancient Greek music, Martin West, remarked that the melodic functionality of Greek music must have been decidedly descending, explaining this by its inheritance from the Archaic tradition (West 1981).

Ascension and declination are not the only inclinations of the indefinite intervallic typologies. The idea of activation and relaxation, represented in these inclinations, comfortably forms a compound, constituting an inclination in its own right—a *wave* pitch contour. The most common form of a wave is an arch. Just as every ascending phrase is likely to charge the melodic material with energy, and every descending phrase—to make it relaxed, in the same way every arch is likely to project a balanced impression, associated with confidence, control, and moderation. Subsequently, Alekseyev believes that the third in line of the evolution of the directional typologies should be the hybrid of ascending and descending inclinations—the **convex wave**: rise of the intonation immediately after taking a breath, when the resources are plentiful, followed by a comfortably falling intonation towards the end of a phrase (Alekseyev 1976, 133).^[[7]](#footnote-7)^

In simple vocalizations by non-professional singers, the prevalent directionality of a melodic phrase is likely to be descending, arch, being the second most common, and ascending—the third. This is in line with the prevalence of arched and descending pitch contours in majority of verbal utterances, which are also the first to be acquired during infancy as respectively a declarative and an imperative speech acts (Tomasello 2003 p. 36). The intonational profiles learned during the second year of life must provide the basis for strategies of planning musical phrases employed by young children, and therefore they retain their declarative and imperative functionality in their initial attempts of spontaneous vocal music making.

For pre-diatonic music systems, modal inclinations are determined by the succession of the first tones that open the melody: they set the direction, and limit available options for placement of an anchor tone (Alekseyev 1976, 137). The closer is the first rising or falling to the opening of a new phrase, the greater is its formative impact on a musical mode (p.138). The direction of the initiating intonation defines the position of the modal anchor due to the biological universality of breathing cycle (p.139). The ascending inclination usually starts from the principal anchor and proceeds toward the challenging anchor (“tonic”-“dominant” functional relation). The descending inclination usually ends on the anchor, or slightly passes it by an extra tone (usually complementary), and starts on an unstable tone. The tonal characteristics of the arch of a convex wave depend on the distribution of pitches along the metric grid, determining rhythmically “male” or “female” termination of a phrase, where metric stress would mark the most stable anchor. Certainly, these three scenarios are not the only ones possible, but they are expected to be statistically prevalent.

The most important contribution of melodic directionality to the modal analysis is that the “closure tones” (shifts in melodic direction) in the opening intonation of a melodic phrase (especially in formulaic melodies) provide the clearest definition for a degree in pre-diatonic indefinite-pitch modes (ekmelic and khasmatonal) that are the hardest to detect. The upper closure is likely to form the “dominant” functionality towards the lower anchor, marked by the lower “closure” or the tone right above it—setting a competing relation between both anchors. The lowest of them is likely to present a gravitational center for a mode. In emmelic modes (definite pitch), the closure tones are likely to indicate a stable or a relatively stable degree in a mode.

#### Ten rules of melodic articulation.

First, let us sum up 6 rules of melodic articulation that we have so far identified.

1. ***Temporal Continuity*** rule—the tones of the melody should follow one another with minimal breaks of time, typically less than 0.8 second. A break longer than that should be taken as a marker of a new melodic phrase.
2. ***Syllabic Distribution*** rule—the frequency changes in vocal music are related to the patterns of distribution of syllables in lyrics or vocables, which adopt the function of elementary units of melodic organization, so that the onset of a syllable marks a particular pitch value.
3. ***Syllabic Hierarchy*** rule—the organization of syllables into words, and words into phrases, usually corresponds to the analogous organization of pitches into melodic intonations, and melodic intonations into motifs, where the pitch that falls on the head syllable in a word, and the head word in a phrase, receives tonal prevalence over other pitches. The last syllable in a phrase also obtains tonal emphasis—greater than the lexic, but smaller than the phrasal head.
4. ***Caesura Proximity*** rule—the gap in the succession of sounds, introduced by breathing, makes the frequency value of the tones that immediately precede and follow the breath more important (including in instrumental performance). This relates not only to the actual taking of a breath, but also to any articulation that shortens the actual duration of a tone while keeping its metric value (provided that the entire phrase is not articulated staccato) or the inserted silence that was generated by expanding the metric value of the corresponding beat.
5. ***Caesura Pulsation*** rule—groups of tones, enclosed by caesuras, delimit melodic phrases, the regularity of whose span contributes to the establishment of a metric feel in the succession of frequency changes in the melody. Regular caesuras produce relaxation and charge those frequency values after which they fall, with tonal stability. Irregular caesuras increase melodic tension and tonal instability. Caesural “map” usually retains the same average pulse as long as the same thematic material is elaborated (e.g., repetition or variation of a melodic pattern).
6. ***Directional Inclination*** rule—the direction of the melody at the beginning of a musical phrase (immediately after caesura) sets the breathing algorithm and the distribution pattern of the tonal stress over the melodic contour. This directionality can be simple (elementary) or complex (compound). There are 3 inclinations that are most common:
7. ***Descending Inclination*** (elementary) sets an expiration-based model, based on the gradual release of energy that marks the last tone or the tone before last, of a descending phrase, as the “leaning tone.”
8. ***Ascending Inclination*** (elementary) sets the inspiration-based model, based on the accumulation of energy that reaches its climax, marking the initial tone as “leaning,” and the climactic tone, as the most unstable.
9. ***Wavelike Inclination*** (compound) sets a balanced inspiration-expiration cycle, marking the tones depending on whether it is a “male” (trochaic, prevalent in pre-diatonic folk music) or a “female” (iambic, common in diatonic music) metric stress in the phrasal ending.

These 3 inclinations are not the only ones that might be engaged in the production of melody (e.g., there is also the *zigzag* type). However, the 3 above-mentioned inclinations are the most important. Due to their engagement in prosodic acquisition of verbal speech, they likely constitute a prosodic universal that spreads over the domain of music as well. And if that is the case, then every indigenous music culture should contain these basic inclinations.

Modal inclinations exercise formative power over the morphological units of melody: its intonations, motifs, and phrases. Melodic articulation controls the elementary level of structuring the melodic motion by assigning specific melodic functions to the intonations depending on their position in the stereotypical melodic contour and on their registral position in the vocal ambitus. This dependency generates 4 more rules.

1. ***Syntactic Function*** rule—the portions of a phrase, rendered in accordance with a particular modal inclination, contain melodic intonations that necessarily support a specific syntactic function within that phrase (its initiation, climax, or termination). Switching between such functions shapes the melodic motifs. Since each inclination limits the range of possible changes applicable to the melodic line, at any given moment there are only a few options of phrasal intonations suitable for continuing the melody.
2. ***Binary Typology of Intonations*** rule—each phrasal intonation is tonally defined in 2 aspects: *melodic direction*, set by the modal inclination, and *intervallic value*, set by the “intonational function” within a particular portion of a melodic contour (e.g., the ascending initiation or the descending cadence). Each tone of the melodic line necessarily adopts a specific directional and intervallic relation with the preceding connected tone.

Compositionally, “intonational function” is a type of melodic motion that a tone can perform to produce variations in directionality: e.g., the initiation of an arch phrase can occur through one of two functions—by a leap or by a step. Each of the two possesses a certain intervallic range (leaps and steps can be of different sizes). Each range, in turn, is defined by the perceptual mechanism of “segregation of audio stream” (Bregman and Mcadams 1979) according to the “temporal coherence boundary” (Noorden 1975) that is responsible for splitting a single melodic line into two. The interplay of a critical intervallic size threshold (300–400 cents) and tempo (the faster the tempo the greater the threshold), distinguishes the *melodically* *dissonant* leap from the *melodically consonant* step: leap has the power to break the melody, whereas step does not have this power (Huron 2001).

Even if a leap does not actually fissure a melodic line in a particular piece of music (i.e., a melody is not broken into 2 melodies by a leap), leaping is still perceived as a “dissonant” melodic event that requires extra processing on the part of the brain, making the perception of leaps inherently more costly than that of steps. This opposition between the easy processing of steps and the difficult processing of leaps must be responsible for their association with consonance/dissonance, which seems to have biological roots—very much like the perception of acoustic harmonic consonance and dissonance. Thus, the segregation of auditory streams is found to be already active at birth (Stefanics *et al.* 2009), and require no attention from the listener (Sussman *et al.* 2007).

Ascending/descending step/leap dialectics determines the melodic motion (Larson 1997). Any tone either steps up or down (consonant tension/relaxation), leaps up or down (dissonant tension/relaxation), or is repeated (neutral tension/relaxation—i.e., repetition of the same pitch value either indicates insistence or hesitation, both of which neither disrupt nor promote the melodic motion).

Therefore, as related to their status, any pair of connected tones has only 4 options. A pair either emphasizes a single pitch value by reproducing it (repetition), complements the principal pitch value with a subordinated auxiliary one (subordination), opposes one pitch value to another alternative value of equal status (coordination), or overwhelms one pitch value with another value of greater status (polarization). This produces 4 intonational functions.

1. ***4 Intonational Options*** rule—there are only 4 intonational functions that are possible between any two connected tones within a melodic line:
2. ***Anchoring Function*** (ƒ-anc)—the melodic motion marks the pitch level of a tone as an attractor for some other unstressed (unstable) tones by means of its immediate repetition (reinforcement). In terms of diatonic music theory this function is expressed by the melodic interval of unison. As a form of melodic motion prototyped after physical motion, ƒ-anc represents stepping “in the same place.” This can be “positive” in a sense of “insisting” on a pitch value (as in “restating” something), or “negative,” as a compensatory affirmation in the situation of uncertainty (as in “stuttering”). So, ƒ-anc might sound pushy (e.g., in rap-songs), restrictive (e.g., in enchantments or spells) or undecided (e.g., in monotonous recitatives).
3. ***Complementing Function*** (ƒ-com)—the melodic motion marks a tone as subordinate to a neighboring stressed tone that acts as an anchor attracting this complementary tone. Diatonic music theory qualifies this function as the melodic interval of 2^nd^ (minor or major). As a form of melodic motion, ƒ-com represents stepping “from one place to another.” It can also be “positive,” in case a complementary tone leads to a new successive anchor (as in a “passing” melodic motion—“positive” in a sense of heading somewhere, therefore being constructive), or “negative,” if a complementary tone reinforces the previous anchor (as in “auxiliary” melodic motion—“negative” in a sense of “shifting weight from foot to foot” without heading toward a specific target). Accordingly, ƒ-com can project positive tension (as in being determined to attain a goal, e.g., in marches) or negative relaxation (as in idleness, hesitation, or meditation, e.g., in plainchant).
4. ***Opposing Function*** (ƒ-opp)—the melodic motion marks a tone as the coordinator of the adjacent tone by presenting an alternative anchor that is potentially capable of breaking the melodic line into two parts or causing a so-called “implied polyphony” (Bukofzer 2008 p. 533). Unlike the two options above, this one presents a melodic dissonance by obstructing or resisting melodic fluidity. In terms of diatonic music theory this function is expressed by the melodic intervals of 3^rd^ and 4^th^ (in very fast tempo it could be a 5^th^). As a form of melodic motion, ƒ-opp represents “medium size leaping” (usually over a degree, as if stepping over a stair in a staircase) between two modal degrees that are approximately *equal* in their gravitational values (have the same level of gravity). Unlike the previous two functions, ƒ-opp adopts a negative connotation, if it is ascending (abrupt increase in tension, e.g., in play-songs), and a positive connotation, if it is descending (sudden relaxation, e.g., in lullabies).
5. ***Extreme Function*** (ƒ-ext), or, *polarizing* function—the melodic motion marks a tone as one that is pushing the registral unity to the limit by skipping to the opposite end of the register and landing on a tone whose gravitational value contrasts the preceding adjacent tone. Unlike ƒ-opp, this function does not involve competition for dominance between two tones. Here, one tone is stressed, whereas the other is unstressed, while both are intervallically divorced—what can be characterized as their polarization. In terms of diatonic music theory this function is usually expressed by the melodic intervals of 5^th^ and wider (but can engage 4^th^ in slow tempo). As a form of melodic motion, ƒ-ext represents “long leaping” (over two or more degrees, as if jumping over a few hurdles) between two modal degrees that are contrasting in their gravitational values (two different levels of gravity). Like the opposing function, ƒ-ext sounds negative, when it is ascending (sudden overwhelming of the low anchor with more salient upper end of a register, e.g., in yodel), and positive, when it is descending (sudden drop of energy while moving to a less salient end of the register, e.g., in motherese).
6. ***Singularity of Intonational Function*** rule—each melodic intonation carries a single intonational function that is related to a single syntactic function through its modal inclination. Any given tone belongs to some melodic intonation of either ƒ-anc, ƒ-com, ƒ-opp or ƒ-ext type, which injects an amount of tension or relaxation necessary to support initiation of a phrase, its climax, decay, or cadence (perfect or imperfect).

Although the idea for such categorization of intonational functions was originally inferred through the comparative analysis of the repertory of ekmelic melodies (Alekseyev 1976; 1986), which I have adapted and generalized for any music that uses indefinite intervallic typology (Nikolsky 2015), *the framework for the four intonational functions should be applicable to any kind of frequency-based music, including tonality*. Locomotive foundation of the opposition of leap and step makes it very likely that their dialectics constitutes a musical universal. Any dyadic intonation can either leap or step, which can be either subordinated (one tone is stressed more than the other) or coordinated (both tones are equally stressed). Any of the two connected pitch levels in an auditory stream belong to one of 4 options (Table-2):

- Melodically consonant, when both tones (two instances of the same degree) stressed (ƒ-anc);
- Melodically consonant, when one tone stressed, whereas another unstressed (ƒ-com);
- Melodically dissonant, when both tones stressed, or consonant, when both tones unstressed (ƒ-opp);
- Melodically dissonant, when one tone stressed, whereas the other unstressed (ƒ-ext).

**Table-2**. Approximation of Ekmelic Functions by means of Diatonic Interval Ranges. “Function’s pitch range” represents the minimal and maximal intervallic values for each “ekmelic interval” that is generated by the ekmelic function in terms of harmonic intervals of the diatonic music system. “Equivalent Diatonic Intervallic Class” indicates the median melodic interval class of the diatonic music system that corresponds to an ekmelic “functional interval” through a particular relation of tones in their frequency levels.

| Function’s symbol | Function’s name & brief definition of its ekmelic intervallic class | Function’s pitch range (in diatonic intervals) | Equivalent diatonic intervallic class |
| --- | --- | --- | --- |
| ƒ-anc | **Anchoring** (reaffirming stability of a modal degree by its repetition and prolongation) | Unison, plus/minus about a quartertone | Unison: consonant (perfectly smooth transition) |
| ƒ-com | **Complementing** (supporting a modal degree by stepping up/down and returning to it) | Minor 2^nd^ up to minor 3^rd^ (augmented 2^nd^) | 2^nd^: consonant (slightly uneven but relatively smooth transition) |
| ƒ-opp | **Opposing** (two remote yet timbrally similar degrees that compete for the anchoring function) | Minor 3^rd^ up to Perfect 5th | 3^rd^: consonant or dissonant (coarse transition that generates tension) |
| ƒ-ext | **Extreme** (two degrees polarized in their registral position & timbrally contrasting) | Range of ƒ-opp + range of ƒ-com | 4^th^: dissonant (jagged transition that requires resolution) |

The tendency of listeners to group tones based on stress is so strong that it is responsible for what has been known as “subjective rhythmization”—subconscious imposition of two or three, rarely four, group membership upon the heard tones even if they are actually equal in their acoustic attributes (Fraisse 1982). Any form of salience of a particular tone in terms of loudness, frequency, duration, or timbre results in that tone being perceived as stressed and marking the first or the last tone in a group. For a succession of two unstressed tones to be noticed, that means that an intonation would have to be expanded from 2 to 3 (or more) tones to include a stressed tone. Therefore, the hypothetical consonant and dissonant dyadic intonation that would consist of unstressed tones alone is not practically feasible.

*Color-coding* will represent the intonational function of each frequency change in the melody.

- The ƒ-ext will be indicated by a red thin line that connects two rectangular bars representing two tones,
- the ƒ-opp will be shown as color yellow,
- the ƒ-com—green, and
- the ƒ-anc—blue (Fig.2).

Other than marking the melodic consonance by darker colors, while melodic dissonance—by brighter, this color-coding also reflects the contradistinctive relations between the pairs of functions. Thus, the ƒ-ext counters the ƒ-com and the ƒ-anc—just as red color counters green color. The ƒ-opp counters the ƒ-anc and the ƒ-com—just as yellow counters blue and green. On the other hand, the ƒ-opp complements the ƒ-ext—just as yellow complements red, whereas the ƒ-com complements the ƒ-anc just as green complements blue.

#### Graphic melodic chart

Graphic representation of the TO data outlined above cannot be based on waveform and spectrogram—the most common means of visualization of acoustic parameters of music and speech—since they are capable of indicating neither the functionality of gradations in articulatory styles, nor caesura patterns, or typology of inclinations and intonations, and they certainly do not disclose patterns of the 5 important AEs of music: tempo, meter, harmony, texture, and music form (thematicity). Conventional musical notation is as useless in simultaneous display of all AEs in music. It can only very roughly indicate the patterns of 7 (out of 10) AEs: pitch, harmony, rhythm, meter, tempo, dynamics, and form—which is deficient for thorough comparative analysis of flections in tuning and timing, and is totally unsuitable for visualization of functionality of articulations, inclinations and intonations. The most detailed frequency/time/amplitude information is provided by spectrograms generated by stereo editing software capable of frequency and/or spectral analyses of an audio selection (e.g., iZotope RX Pro). However, the spectrogram view has to be simplified to simultaneously reflect the characteristics of multiple pitch- and time-related AEs.

Of all the existing visualization methods, the fullest and most refined multi-factorial representation comes from the stylization of pitch variations introduced by d’Alessandro and Mertens (1995) for the analysis and transcription of pitch variations in speech, based on the research by Hart (Hart 1976). This stylization method simulates tonal perception by approximating the image of the sound in the listener’s auditory memory, according to the principles validated in the listening experiments (Mertens *et al.* 1997). Following this model, Mertens developed a new system of visual representation of prosodic organization of speech—**prosogram**—and built a computer application capable of automatic parsing of speech and measurement of prosodic features of individual syllables: their duration, pitch value, melodic direction, intervallic size, speech rate, share of pauses, pitch ambitus, pitch trajectory, and the extent of portamento (Mertens 2004). In its latest implementation, prosogram enables cross-linguistic speaker-independent comparison of the transcribed speech samples (Mertens 2013).

Although Mertens’ Prosogram Tool might be applied to music, it is optimized for automatic recognition of pitch changes between syllables of speech, and its efficacy in parsing vocal and instrumental music remains to be tested. However, many of its limitations are already obvious. Even if Prosogram is used for measuring fine frequency and temporal changes in vocal monophonic music, 5 AEs that are exceedingly important in music communication (harmony, texture, tempo, meter and music form) fall entirely out of Prosogram’s scope. And Prosogram’s representation of harmonicity is way too rough to support the comparison of different timbres in regard to the relative intensity and tuning of their harmonics—which is indispensable for identifying TO in timbre-based music (Nikolsky *et al.* 2020).

The principal advantage of prosogram—its reduction of the graphic representation to the bare minimum of features that are audible and pertinent to TO of music—should be adapted to multi-factorial nature of musical composition. In addition to this, the necessity of pitch scaling for establishing TO in music demands high concision in comparing the exact pitch levels and rhythmic values for each tone of a music work, which they all have to be visible at a single glance—something that is unnecessary for prosodic analysis. That is why the prosogramic approach has to be implemented in a new format, friendly to music. I propose to call it a **musogram**—*a graphic stylization of a melodic line (or a melodic voice/part within a multi-part texture) called forth to display all patterns in frequency-, time-, amplitude- and timbre-related AEs of TO*.

- The spectrogram of an analyzed melodic line should be reduced to a pitch contour based on the criterion of importance or unimportance of a frequency change: *switches between different pitch classes and alteration of a specific modal degree* are important for TO, whereas melodic or timbral ornamentation (shakes, tremolos, trills, portamento slides, and expressive noises, such as breathiness) is not.

This requires a manual input on part of the researcher (until the automatic recognition of ornamental elements becomes possible in software implementation of musogram analysis). Each meaningful frequency change is represented by a straight line on two axes (Fig.1): vertical (frequency in Hertz) and horizontal (time in milliseconds). Vertical line reflects discrete pitch changes, whereas diagonal line—a gradual inflection (i.e., portamento) that carries a semantic function (e.g., the timbral equalization of two contrasting registers in a wide leap usually expresses mellowness). Duration of the sustained pitch level determines whether a tone under question should be regarded as meaningful (pitch class or alteration) or meaningless (ornamentation or unintended artifact, resulting from technical imperfection).

The boundary between singing and speaking usually lies ≈250 ms of a more or less sustained pitch level. Like music, speech uses changes in pitch levels that might be discrete—in which case, they can be qualified as “level tones” (Patel 2010 pp. 39–44). In the reciting style of speech (e.g., poetic verses) level tones are usually longer than in prosaic speech—lasting for about 100–250 ms, whereas musical pitches in folk songs typically last for 230–3,400ms (Nikolsky, 2015, Demonstration-1). If the thematic material contains fluctuations in pitch, let the software resolve the frequency in an audio selection from the audio clip. Here, it is important to optimize the exact selection in a software frequency analyzer prior to taking measurements. The exact beginning and end point of the audio selection should be verified by audition: to establish at which exact moment during the playback the change in frequency becomes audible.^[[8]](#footnote-8)^

In the absence of a clear-cut glissando threshold and strong perceptual asymmetry of ascending and descending glissando (d’Alessandro *et al.* 1998), it is imperative to rely not only on the graphic display in the spectrogram in deciding which frequency changes should be taken into consideration for modal analysis. Careful audition of a clip is as important as its visual examination. Listeners usually discard short-duration frequency fluctuations in order to average the intra-syllabic F0 movement of the pitch contour, reducing these smooth fluctuations to discrete pitches—unless the gliding exceeds the “glissando threshold” (Patel 2006). Engaging this “natural” mechanism of auditory pitch reduction might provide a clue for correct interpretation of pitch changes in situations where a software cannot deliver a clear result. It might be useful to omit consonants from the selection. Consonants usually take 10–75 ms of the voice onset time before the vowel initiates periodic vibrations that are noticeable in the spectrogram (Zsiga 2013).

The exact frequency and time value for each tone will be marked underneath its beginning point (Fig.1). It is inevitable that the choice of different software, slight inconsistencies in selecting the audio fragment for analysis by different researchers, and discrepancies in their interpretation of controversial intonations will introduce discrepancies in frequency analysis of the same audio material conducted by different people. However, experimental trials reveal that such discrepancies are rather negligible—10 cents maximum for frequency analysis of an isolated tone, and much smaller, in the order of 1.6–3.4 cents, for defining degrees in a musical mode (Ambrazevičius & Budrys 2012). This is far below the threshold for the identification of short melodic patterns: 10–20 cents (Parncutt and Cohen 1995).

In the figure below, a bunch of rectangular horizontal bars represent distinct pitch classes, interconnected by straight thin line from the point of decay to the onset point of the subsequent tone of a different pitch value. A system of horizontal dashed grid lines is to mark those frequencies that are important for the definition of modal degrees. Horizontal grid needs to be optimized for a given music work to capture the incremental changes that are most significant for its TO. Above each tone in the graph, a numeral reflects its order in the sound clip, followed by the punctuation mark that indicates the syllabification of that pitch. The pitch that opens a phrase has an exclamation sign in front of it. The checkmark precedes the numeral where the gap articulates the melodic motion. The size of the checkmark reflects the relative value of the break. In the example below, a little checkmark reflects gaps that are very fine and are barely audible (however such gaps are easily visible on the spectrogram) that are nevertheless tonally important because of the overwhelming legato articulation.


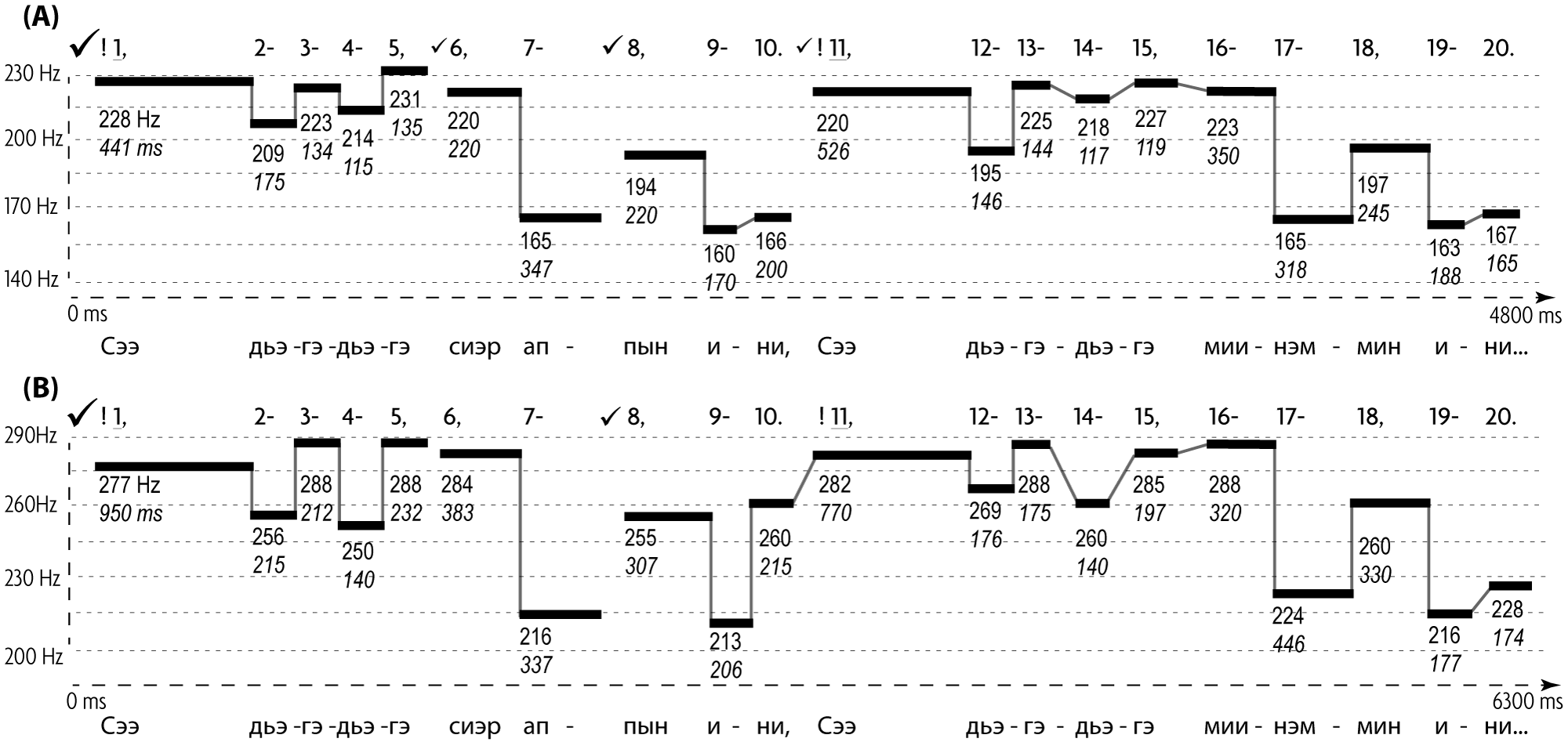


**Figure-1**. The graphic representation of syllabic, temporal, and frequency organization in two versions of the Yakut traditional song’s “Sae Dyige-dyige,” first two verses. **(A)** Lower version, with wider frequency range. **(B)** Higher version, with a narrower range. Each pitch is numbered and assigned a frequency and time value. The underlined number indicates the anchor-tone. The punctuation signs indicate syllabification of the lyrics and their division in words and phrases. The lyrics are written under the graph. Listen to the audio of both versions, following each other, at: <http://chirb.it/g36sC2>

Most of the pitches are connected, and no pronounced caesuras are present after the start of the melody. The audio clip goes further than the analyzed excerpt in Fig.1, yet, it still shows no caesuras until the full stop of the performer. This testifies that the singer conceives this melody as an on-going running streak of the repetitions of the same melodic formula. The syllabification pattern generally supports this conclusion. The lyrics follow the 10-syllable pattern, without melismas, which is typical for Yakut songs (Alekseyev 2013).

The simplified musogram of Fig.1 illustrates how syllabic, respiratory, frequency, and duration information can be used to identify the modal degrees in an ekmelic mode. There are 3 breaks in legato in (A): between tones 5 and 6, 7 and 8, and 10 and 11. All breaks are very small, and had this melody been non-legato, I would have not marked them in the analysis. However, in this legato-driven music, the break between Nos. 7 and 8 violates the interconnection of 2 syllables in the word “аппын” [ap-pyn]. Its separation from the next word, in contrary, is melodically ignored, both words are stitched together without a break in the legato. This contradiction leads one to believe that breaks between words of the lyrics and breaks between the tones of the melody are purposefully intertwined, as in coils in a basket, to provide a greater sense of togetherness. That is why every little break in legato in the style of this music should be counted, and I mark it with a smallest size checkmark.

The only break between words in lyrics that coincides with actual break in legato occurs between tones 5 and 6. This break is accompanied with a functional change: if tones 2–5 were all engaged in ƒ-com complementary relations, tones 6–7 contrast them with their ƒ-opp function (Fig.2). Their relation should be regarded as ƒ-opp rather than ƒ-ext, despite both tones lying at the margins of the song’s ambitus, because both tones are emphasized as anchors—thereby challenging each other. Both tones are supported by an adjacent complementary tone. Both tones receive longer durations. Finally, each of these tones dominates in its dedicated phrasal segment: the upper tone prevails in the opening segment, and the lowest tone—in the second segment. Both segments are equal in their syllabic quantity (5 syllables of the 1^st^ segment and 5 syllables of the 2^nd^ segment). Both half-lines form a clear symmetric relation in a manner of a seesaw, indicating their competition for gravity. Competition for anchoring characterizes ƒ-opp—and not ƒ-ext.

The 2^nd^ line of the lyrics (tones 11–20) corresponds to the repetition of the same melodic formula with slight variations. Tone 14 does not step down like its analog, tone 4, in the first rendition of that formula. Tone 15 is connected to tone 16, unlike the disconnected tones 5 and 6. Tones 17 and 18 do not contain a break between them, unlike tones 7 and 8. Altogether, the second entry of the formula features 100% legato articulation. The fact that the division of the formula in half was present in the first entry and absent in the second, indicates that the performer conceived the formula as a combination of two motifs, “a” and “b,” and thereafter decided that merging them sounded more appropriate for the chosen melodic material—probably, to emphasize the importance of legato. Subsequently, the entire formula should be regarded as a single melodic phrase, comprised of two contrasting motifs.

The first motif establishes the stable anchor at the upper degree. The second motif establishes the opposing anchor at the lowest degree which competes with the upper degree. Both anchors receive support from a complementing degree in the middle of the ambitus. The only melodic inclination that is pronounced in this formula is the descending inclination of the second motif. However, since this motif is positioned far from the opening of the formula, it does not carry a formative modal influence on the entire mode: its ending does not overwhelm the opening anchor established in the first motif. Tones 1, 3, 5 and 6, should all be regarded as the same degree, sharing the same function of stability—generally outweighing tones 7, 9 and 10. This conclusion is confirmed by the upper degree’s lead in consistency of tuning, frequency of its repetition, and its average duration.

At this point we can update the melodic graph (Fig.2) by marking the melodic functionality of each of the intonations and indicating the motifs and the phrases. If the tones at a particular registral position receive one prevailing function while varying much in their exact tuning, then the mode will be considered ekmelic (indefinite in pitch). If no particular function prevails and the frequency values of each occurrence of that pitch level remain close, then the mode is diatonic—i.e., it constitutes a key of Western tonality (well-defined in pitch). Weak prevalence can indicate pre-diatonic emmelic forms of TO (e.g., oligotonal mode—where only modal anchors are well-defined in pitch).

In (A), the upper degree has the mean value of 224 Hz (A3+30 cents), the middle degree—201 Hz (G3+43), and the lower degree—164 Hz (E3-8). The range of fluctuations in frequency values for these degrees are, respectively: 220–231 Hz (which makes 84 cents), 194–214 Hz (169 cents) and 160–167 Hz (74 cents). Evidently, the marginal degrees are much better tuned than the middle degree. The lowest degree is the most concisely defined, but this could just be a consequence of its smaller frequency of repetition: 6—vs. 9 repetitions of the upper degree (this is out of 20 tones).

Timewise, the upper degree has an average duration of 243 ms versus 231 ms for the lowest degree. The middle degree is the shortest in mean time value, at 180 ms. Of the individual tones, the longest in duration are the initial tones that open the formula (the upper degree). The first motif (“a”) is terminated by the same upper degree that started it. The very same degree initiates the second motif. The cadential point of the whole formula is the only prominent position given to the lowest degree—but it receives a very little duration value, by far inferior to the upper degree. The upper degree also receives an edge over the lowest degree in its modal versatility: it has one more ƒ-anc intonation and one more ƒ-com intonation. So, overall, the upper degree seems the most important.


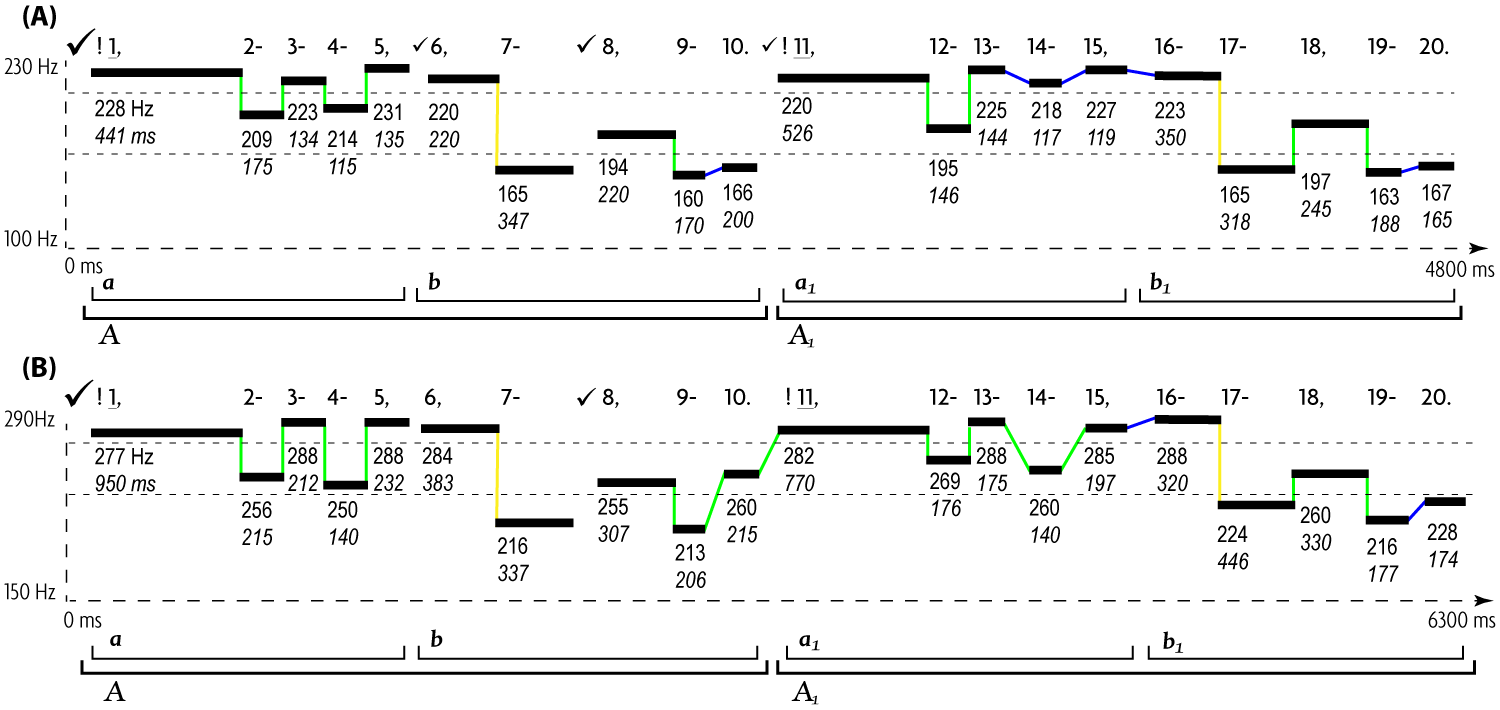


**Figure 2**. The graphic representation of modal organization in two versions of the Yakut song “Sae Dyige-dyige.” **(A)** Lower version. **(B)** Higher version. The mode uses 3 degrees, demarcated by thin dashed lines. The lowercase letters under the graph identify the thematic material of musical motifs. The uppercase letters mark musical phrases. Green color represents ƒ-com, blue – ƒ-anc, and yellow – ƒ-opp intonations. See the video of the playback of this song’s spectrogram: https://youtu.be/FJ11Y2NrFq0

Does the “Sae Dyige” mode consist of 4 degrees or of 3 degrees? Should the complementary degree for the upper anchor be considered different from the complementary degree for the lower, competing, anchor? We do not see a single occurrence of the ascending ƒ-com for the upper and the descending ƒ-com for the lower degrees to follow one another. The middle degree is never repeated and always leads *either* up *or* down. Tones Nos. 8, 12, and 18 registrally overlap in their frequency values: respectively, 194–195–197 Hz for (A) and 255–269–260 Hz for (B). The deciding argument comes from tone No. 12: its short time value and its position in the formula is analogous to tone 2 (that is clearly auxiliary), which indicates that tones Nos. 11–12 must *not* be regarded as ƒ-opp. This limits the use of the middle degree to auxiliary melodic motion, except the single occurrence of the passing melodic motion between tones 9–11 in (Fig.2-B)—justifying the modal scaling of 3 degrees.

#### Six rules of melodic voice-leading

The fundamental law of melodic motion holds that the melodic dissonance (leap) generally requires longer time values than the melodic consonance (step). This is consistent with the general principle of locomotion, according to which, leaping takes greater energy expenditure than stepping, and therefore it takes longer to prepare for it, as well as to recover from it.

There are very few exceptions to this paradigm of melodic and physical motion.^[[9]](#footnote-9)^ It underlies 6 more voicing rules that all have to do with the idea of optimizing a motion for a particular *relative weight* based on the projected mass of the moving object. These 6 rules will continue the previous 10 rules of melodic articulation and apply to diatonic as well as to pre-diatonic forms of music.

1. ***Registral integrity rule***—the tones of a melodic motif and a melodic intonation are supposed to stay within the same registral position to preserve their tonal homogeneity. Timbral break-offs at the end of a phrase, such as harsh gliding “*otglas*” in Slavic songs (Kholopov 2005), mark the termination of the melody by virtue of their tonal contrast to the tonal quality of singing. Similar break-offs amidst vocalization, common in the exaggerated glissando leaps of motherese (Boysson-Bardies 2001), most likely are designed to attract the attention of an infant and therefore break away from the musically oriented rule of registral integrity.
2. ***Prevalence of proximity*** rule—overall, in an average piece of music, the number of melodically consonant intervals (steps and unisons) that readily form a melodic line should exceed the melodically dissonant intervals (leaps) that disrupt the melodic motion. The impression of many leaps in unknown forms of non-Western music might be false, produced by rasterization of indigenous intervals in interval classes of Western tonality—which is prone to mistake a “large step” within an oligotonal mode of wide ambitus for a “leap” within the 12-tone framework.
3. ***Preparation of dissonance*** rule—normally, harmonic theory requires that the dissonant combination of tones should be prepared by the consonant combination. In relation to melodic intervals, this would transpire into such requirement that any leap should be prepared by a step, so that a gradual melodic motion would build the momentum for any abrupt changes. Alternatively, a longer caesura can prepare a wide leap by supplying a greater amount of air to support the excessive expenditure of energy that is required by the coming leap.
4. ***Resolution of dissonance*** rule—ideally, melodic leap (ƒ-ext or ƒ-opp) should be terminated by melodic step (ƒ-com) or repetition of the same pitch value (ƒ-anc). This is equivalent to the harmonic rule of terminating tension of a dissonant chord/interval with relaxation of a consonant chord/interval. Ending a melodic phrase by a leap (melodic dissonance) constitutes an exceptionally strong cadence that is suitable only for special cases such as ending the extensive section in emotionally intense music.
5. ***Relative weight*** rule—shorter rhythmic values require more melodically consonant motion, with closer intervallic proximity of tones. Longer leaps require longer duration values—especially for the pitch that is reached by means of a leap. Leaps in swift passages usually sound too busy, unless they are marked by longer rhythmic values.
6. ***Counterbalance of a leap by steps*** rule—the greater the leaps, the more of balancing the melodic line demands, so that the target pitch of the leap would receive a greater time value to lean on and/or present an opportunity for some kind of melodic relaxation: either by repeating a target pitch (ƒ-anc) or reversing the direction of the leap by throwing in some proximal steps in the opposite direction (ƒ-com). At any rate, the energy of the leap should be matched to the combined weight of the steps and/or repeats in the melody right after that leap.

These weight rules collectively determine why it would be wrong to consider tone No. 12 in “Sae Dyige” as ƒ-opp in relation to tone No. 11. If tone 12 in (A) presents a dissonant ƒ-opp, then it receives neither preparation nor resolution, violating the proximity of dissonance rule by leaping and immediately coming back, and breaking the relative weight rule by an abrupt wiggling on one of the shortest duration values. Moreover, the greater smoothening of tone 14 in comparison to its formulaic analog, tone 4, would make little sense next to the dissonant ƒ-opp. Dissonance requires tension and therefore benefits from increase in contrast rather than the smoothening of it. That is why we have no other choice but to conceptualize a single middle degree to support both marginal degrees with the consonant ƒ-com. But *the upper and lower ƒ-com functions are not equal*. The ƒ-com between the mean frequency values of the upper and middle degrees equals 188 cents as opposed to 352 cents of the lower ƒ-com. The latter is more dissonant probably because of the straining influence of the ƒ-opp function between tones 6 and 7. This particular ƒ-opp is huge at 540 cents.

The comparison of the intervallic distances between the degrees of the lower version of “Sae Dyige” (A) with those in the higher version of the same song, in (B), illustrates the scalable nature of ekmelic intervals. Transposing a song to a higher register causes the exponential scaling of intervallic distances between the tones of its melody. In (B), the upper degree averages at 285 Hz (C#4+48), the middle degree—at 259 Hz (C4-17), and the lower degree—at 219 Hz (A3-8). Their ranges of fluctuation are respectively: 277–288 Hz (67 cents), 250–269 Hz (127 cents) and 213–228 Hz (118 cents). The ƒ-com between the mean frequency values of the upper and middle degrees is 166 cents. The ƒ-opp between the mean frequency values of the upper and lower degrees is 456 cents. And the ƒ-com between the mean frequency values of the lower and middle degrees is 290 cents. Evidently, all 3 ekmelic functional intervals are scaled down during the transposition of the song by about a 3^rd^ up the register: 13% for III-II degrees, 18% for III-I and 21% for II-I.

Interestingly, an increase in pitch became supported by a considerable increase in duration, so that the higher version of the same song turned out to be, overall, 31% longer. Nevertheless, the musical mode of (B) remains essentially the same as (A). The average durations of each of its degrees remain 405 ms for the upper degree, 179 ms for the lower degree, and 337 ms for the middle degree. As we see, in this higher version of the song, the upper anchor receives even greater prominence than its lower “antipode.” Presumably, slower speed has caused the performer to lean heavier on the stable anchor. Modal salience of such leaning is indicated in the graphic representation by underlining the corresponding tone number. Its salience is determined by the initiation function in conjunction with the longest duration in an entire melodic formula.

All in all, in this song, the cadential tones are so short in comparison to the initiation tones, that their evaluation with regard to whether the phrasal endings are female or male is hardly possible at all. In the overwhelming legato articulation, such phrasal endings make more of an impression of anacrusis.

#### Motivic analysis and inference of hierarchy in TO

Now we can proceed to quantification of the key parameters that contribute to TO, which will provide the ultimate answer to the questions of how many degrees are used in the melody and which functions they carry.

Underneath the melodic graph, the square brackets reflect the boundaries of the basic structural units in the changes of thematic material. There are three hierarchic levels, established by syllabification, dynamic contour and melodic patterning (changes in directionality, distribution of duration values, and intonational functionality):

1. **Melodic intonation**—is a succession of two pitched tones that comprises a pitch change. As simple as it sounds, recognition of an intonation can pose a problem in a melody that engages staccato articulation and contains deliberate or inadvertent breaks in the melody due to the performer taking a breath within a word. Such cases require cross-examination of the intonation in question in regard to all AEs. Melodic intonations are used systemically and retain their melodic functionality in most incidents of their use within the same music work—which helps to identify the most important modal intonations. They can initiate a motif or phrase, mark their climax point, terminate a cadence, transform a motif in thematic development, contrast another intonation, or reproduce the same intonation once again.
2. **Melodic motif** —is a succession of two or more intonations, marked at its initiation and termination points by either a pause, longer duration value, a repetition of the same pattern of pitches, or the onset of a contrasting pattern. A single intonation can constitute a motif in case it is distinctly marked by a caesura, long duration value(s), and/or salient leap(s) (e.g., the famous opening of Symphony No.5 by Beethoven). Motifs are usually more specific in their expression than intonations (e.g., they can express such intricate states as “hesitation” or “surprise”). Motifs can be varied by slight modification of constituent intonations through changing a duration value or the exact size of a leap or a step. Motifs can also be extended by adding a tone at the end of a motif, thereby changing its gender from female to male, or at the beginning of a motif by adding an anacrusis. By the same token, a motif can be contracted by taking away its anacrusis or cutting off its final tone.
3. **Melodic phrase**—is a succession of two or more motifs that form a compound structure to express some general idea (usually, the character of an object or an action, or an affective state). The melodic phrase can be recognized by the agreement of multiple AEs in conveying a single idea and by a sense of completion of the melodic motion at its end point, caused by a pause, a long duration value or a dynamic stress placed on the last or second-to-last tone, and the termination of a familiar melodic and/or dynamic contour. In very animated melodies, phrases can “run in” to produce a rushing impression, in which case, phrasal boundaries can be recognized by the reuse of the same intonations. Phrases can be extended and contracted through extending or contracting their constituent motifs. Especially common is fragmentation of phrases, when a previously used phrase is halved (usually, the first half is left over) and reproduced from a different pitch (the so-called melodic “sequence”). Phrases are even more diverse and specific in their expression than motifs (e.g., a phrase can “beg” or “threaten”).

We shall employ letters to mark motifs and phrases. These letters will help identify the patterns in the distribution of melodic intonations according to their similarity and contrast. There are 4 options in total:

1. Repetition of the same melodic pattern is reflected by the repetition of the same letter (“aa”).
2. Variation of a melodic pattern is reflected by adding a numerical suffix after the repeated letter (“aa_1_”).
3. The beginning of some contrasting material is reflected by the use of a new letter (“ab”).
4. The recapitulation of some previous material is reflected by reuse of the previous letter (“ba”).

This abbreviation establishes the makeup of melodic material, which becomes especially valuable, when pauses are scarce. The moment of switching from one letter to another, or to its repetition, as a rule, marks the end of a previous melodic intonation. This is what we already observed in the articulation analysis earlier, between tones 15 and 16 in (A).

The **lowercase** letters indicate the melodic material of motifs: e.g., letter “a” marks a specific configuration of a few intonations usually united by a particular salient melodic function. Every subsequent reuse of this configuration will receive the nomenclature of “a,” if it exactly repeats the original configuration, or “a_1_,” if some modification in frequency or duration is present. Every entrance of a new configuration of intonations will be marked as “b,” “c,” etc.. Square brackets indicate the exact starting and ending points of a motif according to the following factors:

1. the break of silence;
2. a longer sound at the end of a pattern, or of one tone (sometimes two) before the end;
3. an immediate reproduction of the same pattern (from the same or a different pitch);
4. recapitulation (reuse) of some pattern that was used at some point earlier on;
5. the end of the phrase/sentence in lyrics;
6. an abrupt change in the register and/or timbral quality.

The **uppercase** letters will indicate the motivic makeup of each phrase. If the first phrase is made of motifs “a” and “b”, it receives the letter “A”. If the second phrase is made of new motifs “c” and “d”, it receives the letter “B”. If only one of the motifs is new, e.g., “c” combined with “b” that was used in a previous phrase, the phrase still qualifies as “B”, since the start of the second phrase differs from the start of the first phrase in addition to the presence of a new motif. If a new motif is combined with “a” such that the start of the second phrase is the same as the first phrase, and only the end of the phrase is different, then the second phrase qualifies as “A_1_”.

The uppercase letters receive their own brackets, placed under the motivic brackets, to reflect the grouping of the motifs inside of the phrase. For each phrasal bracket, the initial and cadential tones should be noted and examined in the context of the entirety of the music. Apart from their importance for the estimation of modal degrees, their association with specific intonations and motifs might help to identify and define unclear intonations and motifs throughout a musical work.

Once all melodic intonations are thematically identified, the following progression of steps will allow an analyst to infer the degrees of the mode.

1. Use proportionally scaled horizontal grid lines to identify which of the pitches of the melody maintain closely proximal registral position.
2. Cross-examine the melodic motifs, intonational functions, distribution of the shortest and longest duration values, placement of cadences and initiations—to establish which pitch values constitute the “same” degree. Start with those pitches that present the clearest case of a single pitch class. Calculate the extent of pitch variability (S.D.) within that class. This should give an idea of what deviation range is normative for definition of degrees in a given piece of music, and which approximate increments are likely to separate the degrees—these parameters are usually systemic, determined by the choice of intervallic typology (e.g., chromatic, microchromatic, etc.—see Appendix-1 “Taxonomy of modal music” (Nikolsky 2015)).
3. The degrees are usually distributed more or less evenly across the ambitus of a music work—the notable exception is the lowest degree, which can be separated from the II degree by a gap up to about 500 cents (so-called “infrafix”). Sometimes, an uppermost degree can be gapped (“suprafix”). If you see gaps larger than an average increment plus/minus 50% (e.g., 200, 300 and 600 cents between what appears to be 4 degrees, in the ascending order), then the chances are that there are more degrees obscured in a larger span. In which case, a more thorough examination of intonations and patterns is needed, preferably, on a larger sample.
4. After defining the most obvious degrees, isolate the degrees under question. Examine their membership in intonations. Take into consideration the successive order of each intonation and the melodic direction in every instance of its occurrence. The pitch that consistently connects to a higher pitch in similar motifs is likely to be sharpened, and therefore normally features a greater variability in its tuning than a pitch that functions as an anchor point due to its greater duration, loudness, and prominence in a melodic contour. Similarly, the pitch that consistently connects to a lower pitch is likely to be flattened.
5. Examine all instances of a degree under question in regard to registral overlapping and decide whether the overlapped frequency values can be interpreted as two discrete degrees that retain their functionality throughout the melody, or if their overlapping is accidental and should be regarded as a temporary alteration of a single degree in the mode.
6. Take note of which degrees are well-defined and which pitches are vague. It is common to have one or two vaguely defined degrees enclosed between well-defined degrees throughout the span of a musical mode. This is to fulfill Roman Jakobson’s suggestion that the phonological rule that “the difference between two correlated values always emerges as the opposition between a marked and an unmarked value” must have a musicological equivalent (Jakobson 1987 p. 467). Neighboring degrees in musical modes are often related in this way. Thus, in tonality, it is exceedingly common for I, III and V degrees to have low variability, whereas for II, IV, VI, and especially VII degree to be highly variable in tuning.
7. Place an Arabic numeral in bold with a degree symbol (e.g., 1°…) next to each tone in the melody to indicate its position within an entire ambitus of a musical work, from bottom to top. Write out the total number of the degrees found in the entire melody, arrange them in a manner of a scale, and mark their frequency ranges. Do not assume that this enumeration is octave equivalent unless octave equivalence is self-evident from the musical setting (e.g., the music has been composed for piano solo in the 19^th^ century). Otherwise, all degrees should be numbered consecutively even if there are 20 or 30 of them. This is important, because certain degrees in a mode might be non-octave equivalent, some alterations might be specific to certain registers, characteristic modal intonations might be register-specific, causing tuning discrepancies across different registers. Such traits can be characteristic for TO in a musical mode.

Another point to remember is that “number one” here does not necessarily mark the most stable degree. The I degree always remains “tonic” only for Western tonality. Modality as a method of TO^[[10]](#footnote-10)^ is characterized by weak tonicity and by ongoing gravitational shifts between different tetrachords and trichords. Therefore, the numbering scheme where the I degree indicates the greatest stability in a mode should be applied only *after* defining all the degrees in the ambitus, which requires two systems of denomination for the degrees: one *registral* (1°, 2°, etc.), and another *gravitational* (I, II, etc.). The conversion from registral nomination to gravitational is quite straightforward for the modes and keys whose ambitus does not extend an octave: for instance, the mode of the tune “Hush, Little Baby” would have to be converted from 1°, 2°, 3°, 4°, 5° to V-I-II-III-IV, respectively.

However, if we had a melody that consisted of 20 pitches and spread over the ambitus of a few octaves, we would have had to resolve the degrees in their proprietary chroma relations (over an octave)—as whether they represent the same octave-equivalent modal pitch class or different non-octave pitch classes. If the mean frequency values of any two degrees constitute a number close to double (e.g., 440 Hz and 884 Hz), then these two degrees should be regarded as a single pitch class, and their duration value as well as the number of occurrences per musical work should be summed up (e.g., 1°, 8° and 15° all become I). Therefore, octave equivalence can significantly skew the numbers to strengthen “tonicity” of a particular degree. Degrees that do not form clear multiples in their mean frequency values have to be examined more thoroughly.

In a longer piece of music, or in melodies with ambiguous TO, the graphic representation of music might not be sufficient to grasp all the details in TO. In that case, create a table or a spreadsheet that would list the modal degrees in rows, and display the following important parameters of TO in the columns (Table-3):

**Table-3**. The tonal summary of all the pitches in the melody of the first (high) version of “Sae Dyige” (Fig.1a).

| Degree | Tone # | Occur. # | F0 (Hz) | Dur. (ms) | AR (0-6) | Phr. ƒ° | Clos. | Int. ƒ° | Max. Int. | F.Dif. (c) | Dir. |
| --- | --- | --- | --- | --- | --- | --- | --- | --- | --- | --- | --- |
| 1° | 7 | 1 | 165 | 347 | 3 |  |  | opp |  | +11 |  |
|  | 9 | 2 | 160 | 170 | 3 |  |  | com |  | -43 | 🡺 |
|  | 10 | 3 | 166 | 200 | 2 | c |  | anc |  | +21 |  |
|  | 17 | 4 | 165 | 318 | 2 |  | x | opp | 665 ms | +11 | 🡹 |
|  | 19 | 5 | 163 | 188 | 3 |  |  | com | 358 ms | -11 | 🡺 |
|  | 20 | 6 | 167 | 165 | 2 | c |  | anc | 365 ms | +31 |  |
| Total: |  | 6 |  | 1388 | 15 | 2c | 1 | 3 | opp | ARV: 74 |  |
| Mean: |  |  | 164.3 | 231 | 2.5 |  |  | opp/com/anc |  |  | 🡺 |
| S.Dev. |  |  | 2.3 | 73 | 0.5 |  |  |  |  | SDV: 48 |  |
| Share: |  | 30% |  | 31% | 26% | c: 33% | 17% |  | 48% | 17% |  |
| 2° | 2 | 1 | 209 | 175 | 3 |  | x | com |  | +68 | 🡹 |
|  | 4 | 2 | 214 | 115 | 2 |  | x | com |  | +108 | 🡹 |
|  | 8 | 3 | 194 | 220 | 3 |  |  |  |  | -61 | 🡻 |
|  | 12 | 4 | 195 | 146 | 3 |  | x | com |  | -52 | 🡹 |
|  | 18 | 5 | 197 | 245 | 2 |  | x | com | 681 ms | -35 | 🡻 |
| Total: |  | 5 |  | 901 | 13 |  | 4 | 1 | com | ARV: 169 |  |
| Mean: |  |  | 201.8 | 180 | 2.6 |  |  | com |  |  | 🡹 |
| S.Dev. |  |  | 8.1 | 47 | 0.48 |  |  |  |  | SDV: 139 |  |
| Share: |  | 25% |  | 20% | 23% | 0 | 80% |  | 76% | 27% |  |
| 3° | 1 | 1 | 228 | 441 | 6 | i |  |  |  | +31 | 🡻 |
|  | 3 | 2 | 223 | 134 | 2 |  | x | com |  | -8 | 🡻 |
|  | 5 | 3 | 231 | 135 | 2 |  |  | com |  | +53 |  |
|  | 6 | 4 | 220 | 220 | 4 |  |  |  |  | -31 | 🡻 |
|  | 11 | 5 | 220 | 526 | 6 | i |  |  |  | -31 | 🡻 |
|  | 13 | 6 | 225 | 144 | 2 |  |  | anc |  | +8 | 🡺 |
|  | 14 | 7 | 218 | 117 | 2 |  |  | anc |  | -47 | 🡺 |
|  | 15 | 8 | 227 | 119 | 2 |  |  | anc | 670 ms | +23 | 🡺 |
|  | 16 | 9 | 223 | 350 | 3 |  |  | com | 619 ms | -8 | 🡻 |
| Total: |  | 9 |  | 2186 | 29 | 2i | 1 | 2 | ƒ-anc | ARV: 100 |  |
| Mean: |  |  | 223.9 | 242 | 3.2 |  |  | anc/com |  |  | 🡻 |
| S.Dev. |  |  | 4 | 148 | 1.61 |  |  |  |  | SDV: 62 |  |
| Share: |  | 45% |  | 49% | 51% | i: 22% | 11% |  | 31% | 16% |  |
| Grand: |  | 20 | 636 c | 4475 ms | 57 | 2c/2i | 6 |  |  |  |  |

Such summary identifies the principal features of TO: which degree is used more frequently, has higher articulation rating, features the longest time values, and specializes in cadential or initialization functions. This information allows one to see the distribution of gravity (i.e., relative stability and instability of modal degrees) and define the functionality of each of the degrees.

The first column, “**Degree**,” indicates the *registral* degree number as established by the position of the analyzed tone within the entire ambitus of the music excerpt.

The second column, “**Tone #**,” indicates the successive number of every tone that engages the registral degree specified in the first column within the analyzed melody—starting from its opening.

The third column, “**Occur. #**,” lists the number of occurrence (or re-occurrence) of the registral degree specified in the first column within the analyzed melody in the consecutive order of its appearance.

At the bottom row “**Total**” in the first panel for the first degree (1°), the total number of occurrences per analyzed piece is calculated. The more frequently a degree is used, the more likely it performs the stable function within the mode. The lower row “**Share**” displays the percentage of the occurrences of that degree in relation to the total number of tones in the entire melody. This number is useful for cross-comparisons of a specific degree across different modes for estimating its overall comparative modal stability/instability.

The fourth column, “**F0**,” (in Hz) indicates the fundamental frequency for each of the occurrences of a given degree. Its intersection with the row “**Mean**” displays the mean frequency value for that degree. Below that cell, calculate the **standard deviation** from the mean value. It will reflect how consistent that degree stays in its tuning throughout the music. Smaller standard deviation characterizes more stable degrees.

The fifth column, “**Dur.**” (in ms) specifies the exact duration value for each of the occurrences of a given degree. After all the duration values are listed, place the total duration of all occurrences in the row “**Total**”. Underneath that cell, specify the mean duration for a single occurrence of that degree, and still below—the **standard deviation** from that mean value. The longer mean duration value indicates stability, whereas the smaller standard deviation with short mean duration value indicates instability of a degree and/or its complementary melodic function to other degrees. The row “**Share**” displays the share of the duration value of that specific degree in the total duration of all tones in the analyzed melody. This is useful for cross-comparison of a specific degree across different modes.

The sixth column, “**AR**” (in points, from 0 to 6), reflects the articulation rating—the extent of stress imposed by syllabification and breathing cycle in vocal music (in instrumental music it could be simplified to a 3-point rating of legato/non-legato plus breathing between succeeding pitches). The higher the rating of a tone, the more certain is its modal significance. The row “**Total**” displays the sum of all points earned by all occurrences of a given degree. Under this cell, the row “**Mean**” shows the average articulation value per instance of that degree. This is handy when comparing those degrees that do not have high articulation rating totals. “**Standard deviation**” in articulation rating might help to identify cases of chromatic alteration, since greater deviation reflects greater contrast between stable (e.g., anchor) and unstable (e.g., neighboring or passing tones) usages of the same degree. “**Share**” of the AR specifies the percentage of the articulation grading of that particular degree in relation to the Grand Total of the articulation rating of all the degrees for the entire mode.

The seventh column, “**Phr. ƒ°**,” displays the phrasal function of a degree for each of its occurrences in music. Put “i” in case a tone initiates a phrase or “c” in case it is cadential (otherwise leave the cell blank). Available functions depend on the modal inclination. For major/minor tonality, it is “c.” For ekmelic intonations it could be “i”, “c”, and “p” (peak). A degree can specialize in a particular phrasal function, carry few different functions, or be non-functional (neutral). The intersection of this column with the row “**Total**” indicates the total number of phrasal functions for that degree. The row “**Share**” shows the prevailing function for that degree and the share of this function in the total number of occurrences of that degree. This value helps to understand the contribution of a specific degree to a musical mode.

The eighth column, “**Clos**.,” shows whether a degree marks the closure in the melodic contour (i.e., if a given degree is used to change the direction in a melodic line). Put a cross in the corresponding cell in case that particular occurrence of a degree constitutes a directional shift within a musical phrase (tones that are separated by a caesura are not counted). Closures are valuable in providing clues to the reliability of a frequency value in cases of possible chromatic alterations or of ekmelic drift in a stretchable ekmelic interval—the point of reversal in melodic direction becomes salient and acquires a discrete value. The intersection with the row “**Share**” shows the percentage of closures in relation to the total number of all occurrences of that given degree. The smaller percentage of closures is indicative of the passing functionality of a degree—to support the connectivity between two stable neighboring degrees.

The ninth column, “**Int. ƒ°**,” specifies the intonational functionality of a degree: ƒ-anc, ƒ-com, ƒ-opp or ƒ-ext. Note, this parameter is defined by the intonation that *leads to the occurrence* of that given degree, charging it with the corresponding melodic function. This cell should stay blank for the tone that is preceded by a pause. “**Int. ƒ°**” comes especially handy in identifying ekmelic intervallic typology. Ekmelic mode is characterized by strict assignment of a single function for each of the degrees throughout the entirety of a music piece. This parameter can also confirm the use of tonality, in which case different intonational functions would be constantly changing for the same degree. In the intersection of this column with the row “**Total**,” place the total number of different ekmelic functions carried by the respective degree. Under this cell, in the row “**Mean**,” indicate the average number of occurrences for the prevalent function of that degree (note that there could be a tie between two functions).

The tenth column, “**Max. Int.**,” shows the maximal durational value for each of the intonational functions *terminated* by that degree. This parameter is related to the previous column. Add up all the duration values (**Dur.**) for each of the intonational functions listed in the previous column (**Int. ƒ°**). Name the ekmelic function that receives the longest maximal duration in the intersection with the row “**Total**.” This parameter is of particular value for ekmelic and khasmatonal music, where a degree often receives a permanent function. The intersection of this column with the row “**Share**” will show the share of the longest maximal duration of a particular intonational function in relation to the total duration of all appearances of the given degree. This figure is mostly useful for comparison of those degrees of the same mode that share the same functionality (most commonly, ƒ-com). This is important in cases of a few instances where a particular function receive very long durations.

The eleventh column, “**F.Dif.**” (in cents), specifies by how much a particular occurrence of a degree is higher or lower in its frequency in relation to the mean frequency value of that degree. This allows one to identify which particular instance of a degree presents an abnormal fluctuation. Then, such abnormality can be related to a particular spot in the melodic line and explained accordingly (e.g., the fluctuation at the climax point would indicate the need to stress that climax). “**F.Dif.**” also identifies which values are normative for the frequency range of a particular degree, and by how much they deviate from the typical threshold of frequency resolution.

The cell, “**SDV**” in the “F.Dif.” column (in cents), indicates the standard deviation in frequency values for a given degree throughout the analyzed music. Since the value expressed in Hertz does not allow to compare the intervallic ranges across different registers for different degrees (difference in Hertz is not linear: e.g., 10 Hz in low register wider than 10 Hz in high register), we need a conversion into cents. SDV is calculated as the sum of the intervallic values of the positive and negative standard deviations (**S.Dev**., in the F0 column) from the mean frequency value (**Mean**, in the F0 column) of a given degree.

The “**ARV**” cell in the **F.Dif.** column (in cents) shows the absolute registral range of all frequency values for a given degree. It is inferred from F.Dif. as the difference between the absolute maximal positive and maximal negative frequency values for the frequency variations between all occurrences of the same degree (defined by comparing all the values in the F.Dif. column). The distinction between ARV and SDV is useful in distinguishing between incidental and normative fluctuations in frequency values. ARV value displays the *exact registral zone* reserved for the frequency range of a specific degree, including all of its fluctuations in tuning. This is important for comparing degrees in relation to the extent of their registral separation within a mode. In controversial cases this can help in deciding which of the neighboring degrees is altered. SDV value, on the other hand, shows the *intervallic value that is normative* for a particular degree, which also helps to identify chromatic alterations, but is most instrumental in finding out which degree is more stable. The intersection of the column “F.Diff.” with the row “**Share**” indicates the ratio of the ARV value in relation to the grand total of the melody’s ambitus. The higher the number, the more unstable the degree.

The twelfth column, “**Dir.**,” identifies the direction of melodic motion after each occurrence of a given degree. This parameter relates to the previous column (**F.Dif.**). If the positive number in F.Dif. is combined with the ascending direction of the succeeding melodic tones, the deviation in tuning is likely to be caused by expressive tuning—aimed to bring a “departure tone” closer to the “destination tone” for their smoother progression. This is especially helpful in identifying chromatic alterations, where the destination is a step above. Similarly, the combination of a negative number in F.Dif. and the descending direction is suggestive of flattening a pitch in order to come closer to the destination tone that is a step lower. Leave this cell blank for those tones that terminate a phrase and therefore are followed by a pause. The *number of blank cells* is also important. It provides an easy way to estimate the capacity of a degree to resolve a melodic motion. Ascending motion is indicated by the “arrow up,” whereas the descending motion by “arrow down.” The repetition of the same degree is indicated by the “right” arrow. The intersection of this column with the row “**Mean**” identifies the melodic direction that is most commonly triggered by a given degree.

At the very bottom of the table, the row “**Grand**” summarizes the most important characteristics of the entire mode. Its intersection with the column “**Occur.#**” displays the total number of tones in the melody. Its intersection with the column “**F0**” displays the intervallic size of the entire ambitus—from the lowest F0 of the lowest degree to the highest F0 of the highest degree (in cents). Its intersection with the column “**Dur.**” displays the grand total duration of all the tones in the melody (in ms). The intersection with the column “**AR**” displays the total number of the articulation grading points for the entire melody. The intersection with the column “**Clos.**” displays the total number of closures (intonational changes in direction) for the entire melody.

In the example of “Sae Dyige,” the degrees are well marked and clearly separated from each other, testifying that its mode belongs to the definite pitch typology—its 1° and 3° do not show any sign of shifting apart from each other as the melody progresses. However, the consistency of scaling of intervals between the transposed (B) and the original (A) versions does point to the direction of the ekmelic typology. Therefore, the mode should be viewed as a transition from ekmelic to emmelic oligotonal TO. There is no overlapping in the registral positions for any of the instances of each of the degrees. Moreover, degrees are separated by relatively wide registral gaps. This is evident if we sum the “Share” values for each of the “ARV” values (16% + 27% + 17% = 60%). We then see that the inter-registral gaps constitute 40% of the entire ambitus. Obviously, the singer has a very good idea as to what the right registral position is for each of the modal degrees.

The gap between 1° and 2° is 259 cents. This is much wider than the tuning range for 2° (which is the widest of all 3 degrees—at 169 c). And the gap between 2° and 3° is only 32 cents. This discrepancy suggests that for this mode the comfortable melodic motion between 2° and 3° is more important than that between 1° and 2°. Greater distance and stronger stress (durational and articulatory) placed on 2° in the progression 2°–1° suggests not complimentary but *opposing* melodic functionality.

The middle degree features the greatest fluctuations in tuning in the entire sample (up to +108 c for tone No.4). This is the only degree whose SDV exceeds a semitone. Most of its fluctuations are accommodating to other degrees: No.2 comes closer to the upper degree at No.3, and No.4—to No.5, whereas No.8 comes closer to the lower degree at No.9, so does No.18 in relation to No.19. Four out of 5 occurrences of the middle degree (80%) shift in their tuning closer towards the succeeding degree. Such tendency is typical for an unstable “tendency tone” (Huron 2006, 161) which is bound to resolve into a more stable tone. To compare, the upper degree has only 2 out of its 9 occurrences (22%) that feature a shift for a comparable value (-31 c of tones Nos.6 and 11). Its other shifts (-8 c) are too insignificant to count. The lower degree in our example has 2 out of 6 instances (33%) of shift in tuning towards the higher degree (21 c for No. 10, and 31 c for No. 20)—in both cases towards the upper rather than the middle degree. This indicates a greater stability of the upper degree, followed by the lower degree, while the middle degree is unstable and complementary to the other two.

The distribution of intonational functions confirms this conclusion. The marginal degrees oppose each other (prevalent ƒ-opp of the lowest degree), while affirming themselves with the help of anchoring (ƒ-anc) that is more pronounced for the upper degree. The middle degree contrasts both marginal degrees by featuring only complementary intonations (ƒ-com) to both marginal degrees. In fact, this complementary specialization of the middle degree is the strongest as compared to the specialization of other degrees. For comparison, 76% of all occurrences of the middle degree bear complementary function—against just 48% of the opposite function that is prevalent for the lower degree, and 31% of the anchoring function for the upper degree.

The upper degree features the highest frequency of occurrence, the highest articulation rating, two longest duration values and the highest mean durational value. Although 3° is less concise in its tuning than 1°, this is not by much, as it still significantly exceeds 2°. The articulation rating of the upper degree is not only greater but also features higher standard deviation than the two other degrees (1.61 versus 0.48 and 0.5). Evidently, 3° features much greater diversity in syllabification and contrasts between phrasal, lexic and syllabic articulations. All of these makes 3° more salient than 1°.

The middle degree is clearly unstable, since it never terminates the melodic motion in intonations, and features the smallest standard deviation in duration as compared to the other degrees. The middle degree swiftly passes the melodic motion to the marginal degrees. They at times house long durations (aimed to anchor the melodic motion), whereas at other times—brief durations (to complement the other degrees). The middle degree not only features the longest total duration of ƒ-com, but this ƒ-com is the only function that 2° carries—in contrast to 3° that carries 2 functions and to 1° that carries 3 functions.

- The middle degree specializes in supporting other degrees,
- the lowest degree specializes in melodic diversity, thereby fueling the melodic motion by contrasting and challenging the upper degree,
- the ƒ-anc prevalence of the upper degree manifests its overall stability.

The middle degree carries 4 times greater number of melodic closures as compared to the two other degrees, which reflects that this degree is used to “bounce off” the melodic motion rather than to “rest on.” The distribution of closures for the middle degree (80%) by far exceeds that of the lowest (17%) and the highest (11%) degrees. This confirms that the “bouncing” of 3° is a characteristic feature of this mode—supposedly because of the lower degree’s smaller “leaning capacity” in comparison to the gravity of the upper degree. All of these factors combined, along with the overwhelming dominance of anchoring function, confirm the “tonicity” of 3°.

The most stressed degree (3°) is likely to execute a “tonic” function (I), while the second most stressed degree (1°)—the function of a “dominant” (III), establishing the axis of the “opposing” relation across both margins of the register. Similarly, the relation between the “tonic” I degree of a standard key in Western tonality and its “dominant” V degree subscribes to the model of assigning the ƒ-opp functionality to the connection of V–I. In essence, this is exactly what happens in the “Sae Dyige” mode between its 1° (i.e., “dominant” degree = III) and its 3° (i.e., “tonic” = I), whenever they are connected (as in tones Nos.6–7 and 16–17).

#### Six rules of distribution of tonal gravity

At this point 6 more rules can be added to the 10 articulation and 6 voicing rules formulated earlier. These 6 rules deal with the distribution of **tonal gravity**—tonal stability or instability that stays more or less permanent in its relative level of intensity throughout the analyzed music for each of the degrees in a musical mode.

1. ***Incidence of the degree use*** rule—the *degrees that are used more often tend to be more stable* than the less common degrees (Krumhansl 1990, 67). Although this rule has been formulated in relation to Western tonality, there is evidence that it also applies to modality, including non-Western modes (Bharucha 2002). The more often a pitch value is used, the easier it is to remember that value and use it as a reference for other pitches. The rare pitch values are likely to be forgotten throughout the course of listening to the same piece of music (Krumhansl 1979).
2. ***Longer duration value*** rule—the *tones that feature longer duration values are perceived as more stable*, provided their frequency values correspond to the *hierarchic* organization, such as in a key of Western tonality (Smith and Schmuckler 2004). The same is likely to apply to modality. Validity of this rule for folk music was demonstrated by Aarden (2003). Even if tonal hierarchy is weak, longer duration rule still retains its formative power in marking stability through the cross-modal association of “stopping” on a particular pitch with the state of relative equilibrium and repose as opposed to the melodic motion associated with the state of dynamic imbalance and a melodic and harmonic momentum gathered (until reaching the perfect resolution of an unstable degree into a stable degree).

Of these two factors, the longer duration value seems to be more important: fewer occurrences of a degree, yet of much longer duration, tend to generate greater stability than many occurrences of short duration—provided the rest of the factors are all equal.

1. **Primacy of duration over incidence** rule—if the degree(s) that have the longest duration are not the same as the degree(s) that occur most frequently, then the duration criterion receives greater importance than the frequency of occurrence.

This rule remains to be experimentally proven. However, already, there are reasons to believe that it is valid at least for emmelic TO. Stopping the melodic motion on a particular discrete pitch necessarily makes that pitch momentarily stable—the more prolonged is the stop, the less unstable is the corresponding degree in a mode. The more often such stopping occurs, the more stable that pitch level becomes. When melodic halts occur at a specific pitch value most of the time, that pitch value is conceptualized and remembered as a stable modal degree, used to reference unstable pitches in a melodic motion. It comes as no surprise that the music theory of Western classical music holds that the number of non-chordal (unstable) tones does not affect the harmonic status of the underlying harmony *as long as the non-chordal tones are shorter than the beat*, while chordal (stable) tones are longer than the beat (Dubovsky *et al.* 1965)—the longer the leaning, the more stable the degree. Perhaps, the best demonstration of the validity of this rule is the optionality of adding a trill on the long unstable tone in a perfect cadence in classical music (Vakhromeyev 1981). This correspondence is most obvious in metrically regular music, but is not limited to it. Audition the following two examples below and judge for yourself if “C” in them indeed sounds like a tonic (Fig.3):

**Figure-3**. Pentachordal melody with the stable “C” that features the longest duration of all the degrees despite having fewer occurrences than E, D and B. **(A)** Regular metric pulse. **(B)** Irregular metric pulse. The audio recording of these examples can be heard here: <http://chirb.it/83rhtK>

The “halt effect” of longer duration is likely to be amplified by listener’s recognition of the relevant familiar melodic intonations known to have established the “tonic” in a mode of a similar intervallic structure (e.g., three characteristic modal intonations of III-I, II-I, VII-I in a heptatonic major key set the model for the “stability bias” in regard to the I degree even in an excerpt where it is stressed not by frequency of repetition but by longer duration). Memory of the sound of tonic in a familiar musical mode can substitute the actual hearing of that “tonic.” And once the anchor tones are internalized, it is no longer necessary for music to follow the usual statistical regularities in order to be perceived as gravitating toward an anchor point (Bigand *et al.* 2003).

1. ***Tuning permanence*** rule—those *tones whose frequency values vary the least throughout a piece of music are likely to constitute the most stable degrees in a mode*, whereas those tones that demonstrate the highest variability in their tuning are likely to constitute the most unstable degrees. The threshold of deviation from the normative frequency value for this rule should be *about 20 c* for an experienced music user. Nikolai Garbuzov demonstrated experimentally that listeners tend to identify a frequency value that is 6–21 cent away from the reference tone as the “same” pitch (Garbuzov 1980 p. 205). Parncutt and Cohen (1995) established quite close threshold of 10–20 cent for pitch discretization.

The clearest confirmation of this rule occurs in a situation where exactly the same frequency value is generated by the melodic leap over some other degree as well as via a stepwise motion.

1. ***Gravitational contrast pairing*** rule—a stable degree is likely to form the “tendency tone” relation (Huron 2006, 160) to an unstable degree that is the most proximal to it in a musical mode, so that *modal degrees are usually paired through their tonal contrast*. Listeners judge the tones that are closer in pitch to be more similar (Krumhansl 1979). And each stable degree resolves at least one unstable degree: II-I, II-III, IV-III, IV-V, VI-V, VII-I (Bharucha 1996). It is extremely unlikely for two stable degrees in a mode to be positioned right next to each other. It is possible for two unstable degrees to be positioned next to each other (e.g., VI-VII degrees in heptatonic modes).

The importance of this rule is that permanence in tuning of modal degrees shows which degrees execute complementary melodic function most often. Such degrees will expose the greatest standard deviation in tuning. It is to be expected that these degrees should be proximal to stable degrees, even if these stable degrees do not display very low standard deviation. The gravitational power of this rule is weaker than that of the incidence and of longer duration rules.

1. ***Intra-modal transposability*** rule—if functional relation between two particular degrees in a mode can be transposed to some other degrees within the same mode, this mode is likely to possess diatonic organization. *Ekmelic functions are not transposable at all*—except ƒ-anc that can be used on any of the ekmelic degrees. Otherwise, one intonational function is bound to just one degree in an ekmelic mode. Emmelic oligotonal and mesotonal functions afford transposition of ƒ-com function from any “tonic”-like degree to any “dominant”-like degree. *Only diatonic degrees support transposition of auxiliary or complementary functionality between all the degrees* (e.g., I-II, III-IV, V-VI…; or I-III, II-IV…), enabling such transposition of a melody that does not involve re-scaling of all intervallic values [unlike what we found between versions (A) and (B) of “Sae Dyige”].

The importance of this last rule is that the *tonicity strength in a mode stands in reverse proportion to transposability of its intonational functions*. In modes with weak tonic, intonational functions are usually fixed per degree so that many degrees house at least one characteristic modal intonation. In modes with strong tonic, intonational functions can be transferred from one degree to another for most degrees. Thus, keys of Western tonality afford very few characteristic modal intonations. On the other hand, the most unstable modes—modes with indefinite intervallic typology (premelic,^[[11]](#footnote-11)^ ekmelic and khasmatonal)—simply cannot be transposed without re-scaling of all the intervallic distances between the modal degrees. Their melodic intervals are bound to contract or expand at every instance of reproduction of the same interval set from a different pitch level. Modes with mixed intervallic typology (emmelic oligotonal) can be transposed “by interval” from one register to another across the ambitus, while retaining some intervallic values. Modes with definite intervallic typology (mesotonal, pentatonic and heptatonic) can be transposed “by interval” as well as “by degree.”^[[12]](#footnote-12)^

#### The matrix technique of identifying characteristic modal intonations

Now we can finalize our inquiry into the TO of the “Sae Dyige” mode and examine its characteristic modal intonations. Instrumental in this respect is the *matrix technique* invented by Vladimir Goshovsky for the purpose of identification of the morphological variations in geographic distributions of a particular folk tune (Goshovsky, 1964a; Goshovsky, 1964b). A demonstration of such analysis can be found in Goshovsky’s book “At the origins of Slavic folk music”—applied to the Ukrainian ballade “Plive Katchur po Dunayu” (Goshovsky 1971 pp. 255–9). Goshovsky combined the matrix interface and what he called “kinematic graphs” to identify the systemic melodic relations between specific degrees within a mode (in his terminology, the *quality* aspect), as well as to estimate their relative contribution to the melody composed in that mode (the *quantity* aspect).

Goshovsky’s technique was further advanced by Mikhail Roitershtein, who adjusted it for the comparative historic musicological analysis of the melodic styles by W. Mozart and J.S. Bach (Roitershtein 1973). This technique allowed Roitershtein to identify the tendency of increasing triadicity in melodies of Classicistic versus Baroque music, combined with the growing preference for ascending intonations. Thus, he found an increase in half of the instances of melodic motion by the interval of a 3^rd^ as well as triple reduction of descending intonations in melodies by Mozart as opposed to Bach (6:1 ratio of descending to ascending intonations for Bach versus 2:1 ratio for Mozart). Roitershtein interpreted this as demonstration that *the very same framework of Western tonality could feature different schemes of modal organization*.

The very same hierarchic relations between the degrees of a major key could be realized differently in different compositions created in the same key, if one implementation of that key featured a characteristic modal intonation that was absent in another implementation of the same key. Thus, Roitershtein discovered that Bach preferred descending stepwise modal intonations, whereas Mozart favored ascending arpeggio intonations, which, in effect, generated different musical modes within the same tonal key.

This important conclusion supports another line of evidence coming from the history of tuning. In the beginning of the 19^th^ century, string players had an option of choosing between two different alternative standards: *Gamme europeenne* and *Gamme grecque* (Barbieri and Mangsen 1991). *Gamme europeenne* featured symmetric design of two tetrachords, C-D-E↓-F and G-A-B↓-C (where E and B were slightly flattened); whereas *Gamme grecque* had asymmetric tuning in both tetrachords: C-D-E↓-F and G-A-B-C. Therefore, *Gamme europeenne* emphasized *autonomy* of two tetrachords, marking out their stable tones: C and G (I and V degree of C Major), each of which supported its own network of unstable tones. *Gamme grecque,* on the other hand, subordinated the upper tetrachord to the lower one, assigning greater stability to the lower C (I degree). Therefore, both tunings reflected a difference in modal structure of the very same pitch set. *Gamme europeenne* was formed because of the abundance of melodies in the violin repertoire, where each tetrachord possessed its own characteristic intonations, and there were many intonations that terminated on the V degree. *Gamme grecque* did not have such intonations. So, both gammas presented the same key of C major, yet, via two different musical modes.

The kinematic graph summarizes all the intonations found in a melody. Each of the degrees is represented by a thick dot, connected by straight lines in case the corresponding degrees are conjoined at least in one melodic intonation within the analyzed piece of music. A curved arrow along the straight line indicates from which degree to which other degree the intonation proceeds. Conceptually, “kinematic graphing” is analogous to the “degree dyads” of David Huron (Huron 2006 p. 114), except that the former presents real connections rather than probabilities, and takes into account only those degrees that actually receive a direct melodic connection (not separated by pauses). For both version of “Sae Dyige,” the graph will look like this (Fig.4):


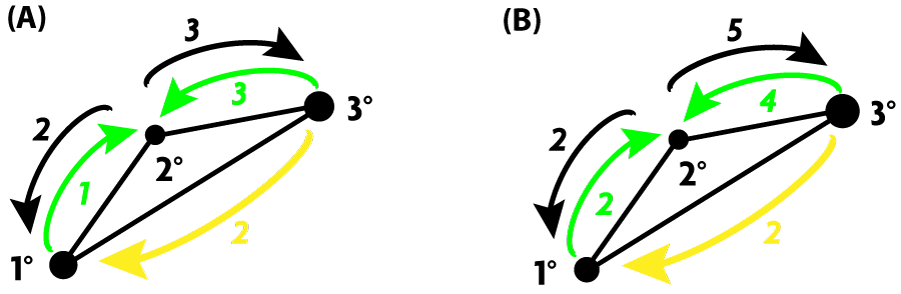


**Figure-4**. The kinematic graph of the modal organization in both versions of “Sae Dyige,”, equivalent to **(A)** and **(B)** of the previous figure. The green arrow represents ƒ-com, and yellow arrow ƒ-opp, where the arrowhead points respectively to the complementary and opposite degrees. Black arrow indicates the return of the ƒ-com motion back to the anchors. The italic number displays the number of occurrences for each intonation. The relative size of black dots represents the relative stability of each of the degrees: the greater is the “leaning” capacity of a degree, the bigger the dot.

The only difference between both versions is the number of occurrences of the same intonations. The mode remains strongly ekmelic due to the pronounced specialization of each of the degrees, while maintaining high volatility of the frequency values of the unstable middle degree.

Not every melody will give that simple a graph. Roitershtein (1973) analyzed all the preludes and fugues from Bach’s “Well Tempered Clavier” and all the themes from Mozart’s keyboard sonatas. He discovered substantial differences in the composers’ preferences for intonations. Thus, Mozart proved to be more versatile by engaging 78 intonations, whereas Bach—only 64. Some of Mozart’s favorite intonations were completely absent in Bach’s repertory (e.g., III->VIII and VIII->II, where “VIII” stands for an upper “chrome” of I, an octave higher). By the same token, some of Bach’s favorites were completely absent in Mozart’s repertory (e.g., VII->III). Each of the composers was found to maintain the integrity of his style across the gamut of works despite contrasts in tempo and keys.

The matrix representation allows to quantify each of the intonational vectors indicated by the kinematic graph. The upper row of the table represents the initial, *departure*, tones of melodic dyads, whereas the utmost left column—their *destination* tones (Tables 4-5). The columns, from left to right, reflect the ascending order of all degrees. The rows, from top to bottom, reflect the ascending order of all degrees for the “destination” pitches in every intonation (the tone that follows its initial tone). The position of the cell that receives a number in a table reflects which two degrees are engaged in an intonation, and which of those degrees comes first. The number in each cell displays how many instances of a given pair of tones take place within the analyzed music work.^[[13]](#footnote-13)^ In case the intonation includes not 2 but 3 tones, at first fill up the cells for the degrees that correspond to the tones No.1-No.2, and then the cells for the tones No.2-No.3. The relation between tones No.3-No.4 is not counted, since in “Sae Dyige” the intonation ends on tone No.3.

**Tables 4-5**. The matrix of the modal intonations in “Sae Dyige,” 1^st^ low version (Table-4) and 2^nd^ high version (Table-5). The blue color indicates the unison intonations (ƒ-com). 1°, 2° and 3° in the upper row represent the initial tones in all melodic intonations. 1°, 2° and 3° in the utmost left column represent the second tone in melodic intonations.

| ***low*** | **1° #** | **2° #** | **3° #** |
| --- | --- | --- | --- |
| **1° #** | 2 | 2 | 2 |
| **2° #** | 1 | 0 | 3 |
| **3° #** | 0 | 3 | 3 |

| ***high*** | **1° #** | **2° #** | **3° #** |
| --- | --- | --- | --- |
| **1° #** | 1 | 2 | 2 |
| **2° #** | 2 | 0 | 4 |
| **3° #** | 0 | 5 | 1 |

Once filled up, the matrix identifies and counts the *characteristic* *modal intonations*—those “combination tones” that characterize a particular mode, i.e., this mode can be recognized by ear upon hearing those modal intonations. Characteristic modal intonations are those that receive the greatest numerical value.

By the same token, this matrix also identifies the *least important intonations*, which might come handy for comparison with another mode that resembles the one under investigation. Thus, in Table-4, the intonations that originate from 3° receive the greatest modal importance. The least important intonation is 1°–3°. The very same mode receives a different intonational filling in Table-5. There we see that the intonation 2°–3° becomes most important, trailed by another intonation, 3°–2°. Evidently, higher registral position of this version prompted the same performer to place greater emphasis on the complementarity of the 2° and 2° degrees.

At the same time, the matrix facilitates the calculation of the frequency of occurrence of each of the degrees for any given melody. To do this, the numbers in all the boxes in column (m) and in row (n), which are both tagged by the same number of a degree, should all be added up.

$$\sum_{i=1}^{n} aim+\sum_{\begin{aligned} j=1 \\ j\neq m \end{aligned}}^{n} amj$$

The resultant number will show *how many times the corresponding degree was engaged in melodic intonations*, allowing to establish the most and the least melodically productive degrees.

Thus, for the lower version of “Sae Dyige” (Table 4), 1° is used in 7 intonations, 2° in 9, and 3° in 11. This information can be cross-related to the frequency of occurrence of each degree within the analyzed piece of music (Table 3). In our example, 6 occurrences of 1° degree produce 7 intonations, 5 occurrences of 2° produce 9 intonations, and 9 occurrences of 3° produce 11 intonations. This comparison of the intonational productivity of both marginal degrees reflects the greater anchoring role of 3°, pointing to it as a single source of modal gravity.

Greater number of intonations per a particular degree, in general, indicates that degree’s contribution to the melodic motion. A degree that produces the fewest intonations is more *static* than a degree that produces the most intonations. Thus, 2° is the most active in the “Sae Dyige” mode because of its complementary relation to other degrees. The most static is 1° due to the isolating influence of its opposing intonations. The ƒ-opp function in relation to a stable anchor is melodically dissonant, making the opposing tone “drop out” from the smooth flow of the melody. Many melodic dissonances falling on the same degree are likely to make that degree in some ways “anti-social” in generation of the melodic motion.

For the higher version of “Sae Dyige” (Table-5), 5 occurrences of 1° are engaged in 7 intonations, 7 occurrences of 2°—in 13 intonations, and 8 occurrences of 3°—in 12 intonations. Evidently, the upper degree here is even more productive, which agrees with its higher articulation rating. We can see how the upward “ekmelic transposition” affects the distribution of intonations by increasing gravitational dynamism and melodic animation in qualitative, as well as quantitative respects.

Yet there is another important generalization that can be made from the matrix analysis in respect to **directionality** of modal intonations. Matrix presents a very convenient way of estimating directions—which is especially valuable for melodies that spread many degrees across extensive ambitus. All it takes is to glance at the diagonal axis from the upper left corner to the lower right corner (Fig.5).

| a_11_ | a_12_ | a_13_ |
| --- | --- | --- |
| a_21_ | a_22_ | a_23_ |
| a_31_ | a_32_ | a_33_ |

**Figure-5**. The representation of melodic directionality in a kinematic graph. The diagonal shading of cells establishes the reference axis for all the melodic pairing within the mode.

All the cells on the *right* side of the shaded diagonal (a_11_-a_22_-a_33_) represent *descending* intonations, while the ones on the *left*—*ascending*. Thus, in the lower song (Table-4), we see the prevalence of descending intonations: 7 descending versus 4 ascending. In the higher song (Table-5), both directions become balanced: 8 descending versus 7 ascending. Such a change confirms a greater melodic animation of the higher version as noted earlier: more frequent alternations in melodic direction become better balanced, thereby making the melodic line more melodically consonant, facilitating its run for a performer and its recognition for a listener.

Finally, we will create the table that will reflect the exact intervallic sizes of the melodic intonations between all the degrees (Tables 6-7). The same matrix that was used in Tables 4-5 will now be filled up with the mean intervallic values of all the occurrences of melodic intonations between each pair of degrees.

**Tables 6-7**. The matrix of mean intervallic values for each of the modal intonations in “Sae Dyige,” 1st (low) and 2^nd^ (high) versions, in cents. The blue color marks the unisons.

| ***low*** | **1° (c)** | **2° (c)** | **3° (c)** |
| --- | --- | --- | --- |
| **1° (c)** | 53 | 331 | 510 |
| **2° (c)** | 307 | 0 | 144 |
| **3° (c)** | 0 | 164 | 52 |

| ***high*** | **1° (c)** | **2° (c)** | **3° (c)** |
| --- | --- | --- | --- |
| **1° (c)** | 94 | 317 | 455 |
| **2° (c)** | 302 | 0 | 160 |
| **3° (c)** | 0 | 173 | 18 |

For instance, the unison on 1° in low “Sae Dyige” occurs twice: the first time, between 160 and 166 Hz, which gives 64 cents, and the second time, between 163 and 167 Hz, producing 42 cents. This gives the mean value of 53 cents. The significance of these sizes is that *the intonations that feature the smallest intervals in a mode usually adopt the function of a modal melodic resolution*, thereby marking the most important stable and unstable degrees in a mode. A well-known example of such a characteristic modal intonation in Western tonality is the progression of the VII-VIII degrees (VIII=I) in a minor key. Ascending resolution of its most unstable degree (VII↑) sharpens it, whenever it is used in an ascending melodic progression. This characteristic modal intonation constitutes a very important pair of “tendency tones” (Huron 2006, 161). It is distinguished by the shortest distance between two adjacent degrees in a Western key, called forth to secure the closest transition to the tonic with the aid of the dedicated “leading” tone, sharpened for this purpose (Friberg 1995).^^[[14]](#footnote-14)^^ Such sharpening is normative for both, vocal (Tchesnokov, 1961, pp. 67–8) and instrumental performance practices (Rags 1999 pp. 175–6). It is this resolution that must have shaped a specialized mode of minor—the so-called “harmonic” mode (e.g., A-B-C-D-E-F-G#-A, characterized by the presence of the interval of the augmented 2^nd^ formed between the “leading” tone, G#, and the VI degree, F).

The easiest way to see how the smallest intonation rule applies to heptatonic modes is to cross-examine the distribution of the characteristic modal intonation of the “leading” 2^nd^ in 3 modes of minor key versus the averaged probe tone ratings for natural, harmonic, and melodic minor modes, as established by Vuvan, Prince and Schmickler (Vuvan *et al.* 2011). The VII#-VIII(I) intonation of the harmonic minor receives the highest rating of 5.30, followed by the fourth highest rating of 4.62 of the I degree. In the natural minor mode (A-B-C-D-E-F-G-A), VII#-VIII(I) is absent, so the smallest sized minor 2^nd^ falls between the VI and V degrees (F-E). The intonation VI–>V here takes on the modal function of a “leading tone” that characterizes the natural minor mode by making its V degree gravitationally stronger, bringing it closer to the gravity of the I degree. Therefore, its “natural” VII degree receives one of the lowest rankings in the natural minor mode, while its V degree outweighs the I degree (5.29 vs. 5.08). “Natural” VI degree receives the fourth highest ranking (4.43).

The third minor mode, the so-called “melodic,” is asymmetric: its ascending interval set (A-B-C-D-E-F#-G#-A) differs from its descending interval set (A-G-F-E-D-C-B-A) by engaging the sharpened versions of the VI and VII degrees on the way up and their natural versions on the way down. This mode hybridizes the harmonic and the natural minor modes by combining their respective leading tones: **VII#**–>VIII in ascending motion with the **VI–**>V in descending motion [the leading tones are bolded]. The highest-ranking degree of the melodic mode, according to the Vuvan, Prince and Schmickler study, is the same as in the natural mode—V degree (5.08)—followed by the VII#, being the second highest ranked (4.91). In all three minor modes, the position of the leading tone that features the smallest size 2^nd^ determines the tonal strength of the modal anchor.

The rule of the smallest size interval is not limited to minor keys of Western classical music. Ambrazevičius and Wiśniewska inferred the “rule of leading tone” from measurements of the tuning of the base 3^rd^ in the tetrachord where the stable tone is positioned at its top—this 3^rd^ was on average 38 cents sharper whenever it was connected to the upper anchor (Ambrazevičius & Wiśniewska 2008). Viktor Beliayev considered the emergence of the “leading tone” to be a universal stage in spontaneous formation of folk diatonic music systems across musical cultures (Beliayev 1990a). He held that the placement of semitone in a musical mode determines its gravitational map.

It is this semitone that is responsible for projecting the impression of directionality in a scale (Roederer 2008 p. 184). Delviniotis, Kouroupetroglou and Theodoridis (2008) experimentally confirmed that performers habitually increase the size of the first interval in a scale, and proportionally decrease the size of the last interval in ascending motion, while inversing this treatment in descending motion—thereby tweaking the semitone’s size in a tetrachord in order to emphasize its melodic direction. Narrowing of the minor 2^nd^ constitutes one of the principal rules of expressive tuning in performance practice (Friberg 1995). Music users tend to put in place conventions for expressive tuning of the degrees for each of the common musical modes. Such conventions include a set of rules for sharpening or flattening unstable modal degrees to facilitate their “resolution” to stable degrees (Bharucha 1996). Directionality of a tetrachord towards the narrowest step most probably constitutes a musical universal, applicable to all forms of TO (including anhemitonic modes). It might constitute an auditory manifestation of a deeply rooted cross-modal perceptual phenomenon known in visual domain as *Fröhlich effect—*the impression of displacement in the direction of the target motion (Hubbard and Ruppel 2013).

The matrix of mean intervallic values greatly facilitates identification of the smallest size intonations in a mode. Thus, Table-3 reveals that apart of the unison intonation (52 cents) that is supposed to reproduce the same pitch class, the smallest intervallic step in the “Sae Dyige” mode is 144 cents for the descending 3°–>2° intonation. Figure-2 provides an additional information that in all occurrences of this intonation, 3° exceeds 2° in its duration. This indicates that the transition from “tonic” 3° to complementary unstable 2° constitutes *a characteristic modal intonation* that distinguishes the mode of “Sae Dyige.” The closest modal sidekick of this intonation is the 2°–>3° intonation whose function is to bring the melody back to “tonic” to complete the auxiliary melodic motion.

Since the duration of both tones in 2°–3° is about the same (Fig.2), and neither duration nor articulation indicate the salience of 2°, the compound 3-tone intonation 3°–2°–3° should be regarded as a characteristic modal intonation of this mode, in agreement with the “proximity rule” of the generative theory of tonal music (Lerdahl & Jackendoff 1985). It is this intonation that should be credited with setting the gravitational map in this music. Its “opposite” anchor, 1°, is defined through the third smallest intonation: 1°–2° (307 cents). This intonation occurs three times less often comparing to 3°–2° (Tables 4-5), reflecting its subsidiary modal function. Analogous to intonation 3°–2°–3°, devoted to the function of marking “tonicity,” the opposing anchor forms a symmetric 3-tone intonation 1°–2°–1°. The anchor point at 1° is set to compete with 3°, but only as an underdog—evident from its lower rate of occurrence as well as the larger intervallic distance and the lower articulation value. Hence, the entire mode is defined by the opposition of just two intonations: 3°–2°–3° domineering over 1°–2°–1°. Table-6 confirms that the higher transposition of the same song does not alter its mode. Both intonations, 3°–2°–3° and 1°–2°–1°, retain their significance. The only intonational difference is that the “tonic” unison receives a much greater tuning precision than the “opposite” unison of the lower degree—indicating the increase in the anchoring power of the upper degree and decrease of such function for the lower degree.

#### The interval strength in a mode and 6 intonational rules

In conclusion of our modal analysis, we shall estimate the “interval strength” of the intonations within a musical mode (Table-8). *Interval strength* is the concept introduced by Andrzej Rakowski (Rakowski 2004) in reference to the relative permanence of tuning for a particular interval within a music work. Interval strength is instrumental in the ultimate definition of modal functionality for modal degrees. “Weak intervals” are those that surround the tones that have multiple options for their melodic progressions. “Strong intervals” correspond to the intonations that interconnect the “tendency tones” (Huron 2006)—tones with reserved melodic progression, which are therefore modally important.

**Table-8**. The interval strength of all modal intonations in “Sae Dyige-dyige,” versions 1 and 2, expressed in standard deviation from the mean intervallic value of each of the modal intonations.

| No. | **1°**-**1°** | **1°**-**2°** | **2°**-**1°** | **2°**-**3°** | **3°**-**1°** | **3°-2°** | **3°-3°** |
| --- | --- | --- | --- | --- | --- | --- | --- |
| 1 F (Hz) | 160-166 | 165-197 | 194-160 | 209-223 | 220-165 | 228-209 | 225-218 |
| 1 Int. (c) | +64 | +307 | -334 | +112 | -498 | -151 | -55 |
| 1 ID (c) | -10 | +4 | -10 | -58 | -16 | +2 | -54.5 |
| 2 F (Hz) | 163-167 | 213-260 | 197-163 | 214-231 | 223-165 | 223-214 | 218-227 |
| 2 Int. (c) | +64 | +345 | -328 | +132 | -521 | -71 | +70 |
| 2 ID (c) | -10 | +42 | -4 | -38 | -39 | +82 | +69.5 |
| 3 F (Hz) | 216-228 | 224-260 | 255-213 | 195-225 | 284-216 | 220-195 | 227-223 |
| 3 Int. (c) | +94 | +258 | -312 | +248 | -474 | -209 | -31 |
| 3 ID (c) | +20 | -45 | +12 | +78 | +8 | -56 | -30.5 |
| 4 F (Hz) |  |  | 260-216 | 256-288 | 288-224 | 277-256 | 285-288 |
| 4 Int. (c) |  |  | -321 | +204 | -435 | -136 | 18 |
| 4 ID (c) |  |  | +3 | +34 | +47 | +17 | +17.5 |
| 5 F (Hz) |  |  |  | 250-288 |  | 288-250 |  |
| 5 Int. (c) |  |  |  | +245 |  | -245 |  |
| 5 ID (c) |  |  |  | +75 |  | -92 |  |
| 6 F (Hz) |  |  |  | 260-282 |  | 282-269 |  |
| 6 Int. (c) |  |  |  | +141 |  | -82 |  |
| 6 ID (c) |  |  |  | -29 |  | +71 |  |
| 7 F (Hz) |  |  |  | 269-288 |  | 288-260 |  |
| 7 Int. (c) |  |  |  | +118 |  | -177 |  |
| 7 ID (c) |  |  |  | -52 |  | -24 |  |
| 8 F (Hz) |  |  |  | 260-285 |  |  |  |
| 8 Int. (c) |  |  |  | +159 |  |  |  |
| 8 ID (c) |  |  |  | -11 |  |  |  |
| Mean (c) | +74 | +303 | -324 | +170 | -482 | -153 | +0.5 |
| SD | 14.1 | 35.6 | 8.2 | 51.6 | 31.8 | 58.8 | 47.6 |

The idea behind this last estimation is that the smaller standard deviation of an intervallic size for each of the intonations within a mode is likely to reflect their greater tonal stability, and therefore mark the most important degrees in a mode (stable as well as unstable)—taking in account their directionality. Obviously, the reliability of this estimation highly depends on the sample size. Ideally, this calculation should involve the entire song rather than 2 verses. However, for this presentation, I only wanted to demonstrate the entire analytical procedure. Each of the occurrences of 7 modal intonations of my sample (1°-1°, 1°-2°, 2°-1°, 2°-3°, 3°-1°, 3°-2° and 3°-3°) is represented as two frequency values, respectively, of the intonation’s first and last tones in Hertz (marked in the table as 1 F for the 1^st^ occurrence, 2 F for the 2^nd^ etc.). The interval between these two frequencies is measured in cents in the cell below it (1 Int. for the 1^st^ occurrence, 2 Int. for the 2^nd^ etc.). Then the mean value for all the occurrences is calculated at the bottom of the table (Mean (c)). Based on this mean value, the *intervallic difference* (ID) is defined—*the extent by which each occurrence of a modal intonation differs from its mean intervallic value*. Finally, the standard deviation (SD) for each of the modal intonations is calculated based on the intervallic differences (ID) of each of its occurrence.

In the example above, the strongest intonation is indisputably 2°–>1°, since it exceeds even both unison intonations in its concision of tuning. The second most important intonation is a unison 1°–>1°, followed by a leap 3°–>1°. Altogether, these three intonations seem to manifest the special importance of the melodic motion towards the lowest degree: both, in descending direction as well as in reaffirming the repetitions of 1°. Taken in the light of the earlier evidence for tonicity of the upper degree, this emphasis on the intonations directed at the lower degree suggests that the entire mode here should be characterized as “*oppositional*”: based on the idea of challenging the “tonic” anchor.

Now we can finalize our list of melodic rules with the last 6 intonational rules, identified and discussed above in relation to the matrix representation of a mode.

1. ***Characteristic modal intonation*** rule—the basic (dyadic) melodic intonation that features *the highest incidence rate* amongst all pairs of degrees within a mode and retains its melodic functionality throughout all (or most) of its occurrences is likely to constitute a characteristic modal intonation that distinguishes this mode.

This rule is especially valuable for inferring a repertory of modes that are common for a particular music culture. Reuse of a musical mode in some other musical work can be recognized through identification of characteristic modal intonations, should the mode in question uses the same set of degrees and a similar gravitational map. We have already touched upon this issue earlier when we established that both versions of “Sae Dyige” share the same mode.

1. ***Intonational diversity of a degree*** rule—the *more intonations* a particular degree is engaged in, the *more modally active* that degree is—i.e., it supports a *more diverse melodic motion*. The fewer intonations a degree supports, the more modally specialized it is, which is likely to limit its contribution to the melodic motion. This correspondence is relative: there could be many instances of a particular degree in a melody, materialized in a handful of intonations—in this scenario, in spite of the melodic activity of such a degree, its melodic motion would be less versatile. This limiting effect will be even more pronounced in a situation where a degree specializes in dissonant ƒ-opp or ƒ-ext functions, that are likely to isolate a degree comparing to the complementary melodic motion.
2. ***Contribution of directionality to gravity*** rule—prevalence of *descending* intonations results in the increase of *stability and relaxation*; prevalence of *ascending* intonations results in the increase of *instability and tension*; relative *equality* of the number of descending and ascending intonations results in the state of tonal *equilibrium*. The latter also depends on the functionality of the intonations: the balancing effect is the strongest where ascent and descent are functionally tied to the dedicated intonations within the same melodic phrase.
3. ***Smallest interval*** rule—intonations that feature the *smallest* intervals within a mode usually adopt the function of *characteristic modal melodic resolutions*, thereby marking the most important stable or/and unstable degrees in a mode. These intonations also reveal the common target tones for the melodic motion, reaching of which earns the highest cadential significance. Like rule 19, this rule helps to identify a familiar mode, thereby enabling the accumulation of the repertory of modes within a musical culture.
4. ***Interval strength*** rule—the *strongest* intervals in a mode are those that connect those degrees that *host a most stable melodic intonation*—i.e., the intonation whose exact intervallic size varies the least throughout all occurrences of that intonation in the melody. The most stable intonation is not necessarily the same as the most stable degree. For instance, in a harmonic minor key, the VI–VII# intonation (augmented 2^nd^) could constitute the most stable melodic intonation, while marking the most unstable VII# degree (a leading tone). Therefore, this rule should be viewed in dialectic relation with a pack of 6 degree-related rules, formulated earlier. The interval-strength rule is most helpful in confirming or denying rule No. 22 in ambiguous portions of the melody.
5. ***Compound intonation*** rule—basic (dyadic) intonations can be combined in triadic (3-tone) and even more *complex* (4-tone and greater) intonations, *if the frequency of occurrence and intervallic strength of the constituent intonations are relatively close, and they functionally interact in a way that is constructive for the melodic motion* (e.g., generating a passing or auxiliary progression, or comprising a melodic closure by connecting an ascending leap with a descending step). The expression of a compound intonation is made of the sum of expressions of the constituent intonations.

The set of these 28 rules will provide a good starting point for formulating a universal generative theory of melodic motion (that covers not only tonality, but modality and timbre-based music) and exploring the possibility of an automated modal analysis of music. Unfortunately, the multifactorial visualization of music currently remains in its virgin state and lacks any software implementation, unlike Mertens’ Prosogram Tool, which makes the task of creating musograms rather tedious. For this reason, it might be advisable for researchers to resort to Mertens’ prosograms and use them in conjunction with conventional musical notation that is capable of fair representation of harmony, texture, meter, and musical form (thematicity). However, the ability to accurately notate an audio clip requires extensive musicological training and ear development. Therefore, for a researcher interested in modal analysis of pretonal music, it might still be easier to learn and follow the procedure outlined above to secure those measurements and their interpretations that fall out of scope of Mertens’ method. On the other hand, a researcher interested only in multi-factorial analysis of the AEs’ patterns might be satisfied by using the Prosogram Tool.

BIBLIOGRAPHY

Aarden, B. J. (2003). *Dynamic melodic expectancy*, Ohio State University.

Alekseyev, E. Y. (1976). *Problems in the genesis of musical mode (on the example of Yakut folksong): analysis [Проблемы формирования лада (на материале якутской народной песни): исследование]*, Moscow: Muzyka.

Alekseyev, E. Y. (1986). *Musical intonation in the earliest forms of folklore. The aspect of pitch [Раннефольклорное интонирование: звуковысотный аспект]*, Moscow: Soviet Composer. Retrieved from http://eduard.alekseyev.org/rfi/index.html

Alekseyev, E. Y. (1988). *Folklore in the context of modern culture: thoughts on the future of folk song [Фольклор в контексте современной культуры: рассуждения о судьбах народной песни]*, Moscow: Soviet Composer. Retrieved from http://eduard.alekseyev.org/fic/index.html

Alekseyev, E. Y. (2013). Ethnomusicological experiment: on the way of trial and error [Этномузыковедческий эксперимент: на пути проб и ошибок]. In A. Varlamova & Z. Pavlova, eds., *Music. Performance. Education [Музыка. Исполнительство. Образование]*, Vol. 4, Yakutsk: University of the Republic of Sakha, pp. 162–179.

Ambrazevičius, R., & Budrys, R. (2012). Pitch Evaluations in Traditional Solo Singing: Comparison of Methods. In E. Cambouropoulos & C. Tsougras, eds., *Proceedings of the 12th International Conference on Music Perception and Cognition (ICMPC) and 8th Triennial Conference of the European Society for the Cognitive Sciences of Music (ESCOM)*, Thessaloniki, Greece: Aristotle University of Thessaloniki, pp. 58–63.

Ambrazevičius, R., & Pärtlas, Ž. (2011). Searching for the “natural” origins of the symmetrical scales: Traditional multipart Setu songs. *Journal of Interdisciplinary Music Studies*, **5**(1), 1–17.

Ambrazevičius, R., & Wiśniewska, I. (2008). Chromaticisms or Performance Rules? Evidence from Traditional Singing Pitch transcriptions. *Journal of Interdisciplinary Music Studies*, **2**(1/2), 19–31.

Aristotle, & Mayhew, R. (2011). *Problems*, Vol. 1, Cambridge, MA: Harvard University Press.

Asafyev, B. (1952). *Selected Works [Избранные труды]*, Vol. 1, Moscow: Academy of Science of the USSR.

Asafyev, B. (1965). *Verbal intonation [Речевая интонация]*, Moscow: Muzyka.

Asafyev, B. (1971). *Musical form as a process [Музыкальная форма как процесс]*, 2nd editio, Leningrad: Muzyka.

Barbieri, P., & Mangsen, S. (1991). Violin intonation: a historical survey. *Early Music*, **19**(1), 69–88.

Barsky, V. (2014). *Chromaticism*, London: Routledge.

Beliayev, V. M. (1990a). Modal systems in the traditional music of the USSR [Ладовые системы в музыке народов СССР]. In I. Travkina, ed., *Viktor Mikhailovich Beliayev [Виктор Михайлович Беляев]*, Moscow: Soviet Composer, pp. 223–377.

Beliayev, V. M. (1990b). *Viktor Mikhailovich Beliayev [Виктор Михайлович Беляев]*, Moscow: Soviet Composer.

Bharucha, J. J. (1996). Melodic anchoring. *Music Perception*, **13**(3), 383–400.

Bharucha, J. J. (2002). Neural nets, temporal composites, and tonality. In D. Levitin, ed., *Foundations of cognitive psychology: core readings*, Cambridge MA: Bradford Books MIT Press, pp. 455–480.

Bigand, E., Poulin-Charronnat, B., Tillmann, B., Madurell, F., & D’Adamo, D. A. (2003). Sensory versus cognitive components in harmonic priming. *Journal of Experimental Psychology. Human Perception and Performance*, **29**(1), 159–171.

Boysson-Bardies, B. de. (2001). *How Language Comes to Children: From Birth to Two Years*. (M. B. DeBevoise, Trans.), Cambridge, MA: MIT Press.

Bregman, A. S., & McAdams, S. (1979). Hearing Musical Streams. *Computer Music Journal*, **3**(4), 26–43.

Bukofzer, M. F. (2008). *Music in the Baroque Era - From Monteverdi to Bach*, Read Books.

Chew, G. (2001). Articulation and phrasing. In S. Sadie & J. Tyrrell, eds., *The New Grove Dictionary of Music and Musicians*, London, UK: 10.1093/gmo/9781561592630.article.08458. doi:10.1093/gmo/9781561592630.article.40952

Clough, J. (1957). The Leading Tone in Direct Chromaticism: From Renaissance to Baroque. *Journal of Music Theory*, **1**(1), 2–21.

d’Alessandro, C., & Mertens, P. (1995). Automatic pitch contour stylization using a model of tonal perception. *Computer Speech and Language*, **9**(3), 257–288.

d’Alessandro, C., Rosset, S., & Rossi, J.-P. (1998). The pitch of short-duration fundamental frequency glissandos. *The Journal of the Acoustical Society of America*, **104**(4), 2339–2348.

Delviniotis, D. S., Kouroupetroglou, G., & Theodoridis, S. (2008). Acoustic analysis of musical intervals in modern Byzantine Chant scales. *The Journal of the Acoustical Society of America*, **124**(4), EL262-9.

Dubovsky, I. I., Yevseyev, S. V., Sokolov, V. V., & Sposobin, I. V. (1965). *The textbook of harmony [Учебник гармонии]*, 4th edn, Moscow: Muzyka.

Etzel, J. A., Johnsen, E. L., Dickerson, J., & Adolphs, R. (2006). Cardiovascular and respiratory responses during musical mood induction. *International Journal of Psychophysiology*, **61**(1), 57–69.

Fenk-Oczlon, G., & Fenk, A. (2009). Some parallels between language and music from a cognitive and evolutionary perspective. *Musicae Scientiae*, **13**(2 suppl.), 201–226.

Fernando, N. (2007). Study of African Scales: A new experimental approach for cognitive aspects. *Revista Transcultural de Música*, **11**(11), 3.

Fitch, W. T. (2006). The biology and evolution of music: a comparative perspective. *Cognition*, **100**(1), 173–215.

Fraisse, P. (1982). Rhythm and tempo. In D. Deutsch, ed., *Psychology of music*, New York: Academic Press, pp. 149–180.

Friberg, A. (1995). *A quantitative rule system for musical performance*, Royal Institute of Technology, Stockholm, Sweden. Retrieved from http://www.speech.kth.se/music/publications/thesisaf/sammfa2nd.htm

Garbuzov, N. (1980). Selected Works (1925-1955) [Избранные труды]. In Y. Rags, ed., *Garbuzov N.A. - Musician, researcher and pedagogue [Гарбузов Н.А. - Музыкант, исследователь, педагог]*, Moscow: Muzyka, pp. 49–263.

Gelbart, M. (2013). Once More to Mendelssohn’s Scotland: The Laws of Music, the Double Tonic, and the Sublimation of Modality. *19th-Century Music*, **37**(1), 3–36.

Goshovsky, V. L. (1964a). Folk music and cybernetics. Part 1 [Фольклор и кибернетика]. *Sovetskaya Muzyka*, **11**, 74–83.

Goshovsky, V. L. (1964b). Folk music and cybernetics. Part 2 [Фольклор и кибернетика]. *Sovetskaya Muzyka*, **12**, 83–89.

Goshovsky, V. L. (1971). *At the origins of Slavic folk music [У истоков народной музыки славян]*, Moscow: Soviet Composer.

Grigoryev, S. S. (1981). *The Theoretic Course of Harmony [Теоретический Курс Гармонии]*, Moscow: Muzyka.

Hart, J. (1976). Psychoacoustic backgrounds of pitch contour stylisation. *IPO Annual Progress Report*, **2**, 11–19.

Hauptmann, M. (1888). *The Nature of Harmony and Metre*. (W. E. Heathcote, Trans.), London: Swan Sonnenschein.

Helmholtz, H. von. (1877). *On the Sensations of Tone as a Physiological Basis for the Theory of Music*. (A. J. Ellis, Trans.), London: Longmans, Green and Co.

Hubbard, T. L., & Ruppel, S. E. (2013). A Fröhlich effect and representational gravity in memory for auditory pitch. *Journal of Experimental Psychology.*, **39**(4), 1153–64.

Huron, D. (2001). Tone and Voice: A Derivation of the Rules of Voice-Leading from Perceptual Principles. *Music Perception*, **19**(1), 1–64.

Huron, D. (2006). *Sweet Anticipation: Music and the Psychology of Expectation*, Cambridge, MA: MIT Press.

Jakobson, R. (1987). *Language in literature*. (S. Rudy & K. Pomorska, Eds.), Cambridge MA: Belknap Press.

Jouste, M. (2009). Traditional melodic types in the music of the Sámi in Finland. In J. Niemi, ed., *Perspectives on the song of the indigenous peoples of northern Eurasia: performance, genres, musical syntax, sound*, Tampere, Finland: Tampere University Press, pp. 240–266.

Kalkun, A., & Oras, J. (2014). Seto Singing Tradition in Siberia: Songs and ‘Non-Songs.’ *Folklore*, **58**, 149–186.

Kholopov, Y. (1976). Mode [Лад]. In Y. Keldysh, ed., *Encyclopedia of Music [Музыкальная энциклопедия]*, Vol. 3, Moscow, Russia: Soviet Encyclopedia [Советская энциклопедия], pp. 130–43.

Kholopov, Y. (1988). *Harmony: A theoretic course [Гармония: теоретический курс]*, Moscow: Muzyka.

Kholopov, Y. (2004). Obikhod modes and multi-part music [Обиходные лады и многоголосие]. In N. S. Guliantiskaya, ed., *Christian culture: past and present. 2000 anniversary of BC [Христианская культура: прошлое и настоящее. К 2000-летию Рождества Христова]*, Moscow: Russian Academy of Music named after Gnessin, pp. 39–54.

Kholopov, Y. (2005). Towards the problem of mode in Russian theoretic musicology [К проблеме лада в русском теоретическом музыкознании]. In E. Struchalina, ed., *Harmony: problems of science and methodology [Гармония: проблемы науки и методики]*, Vol. 2, Rostov-na-Donu: RGK (Rostov State Conservatory), pp. 135–157.

Krivokapic, J. (2012). Prosodic planning in speech production. In S. Fuchs, M. Weihrich, D. Pape, & P. Perrier, eds., *Speech Planning and Dynamics*, Bern Switzerland: Peter Lang Verlag, pp. 157–190.

Krumhansl, C. L. (1979). The psychological representation of musical pitch in a tonal context. *Cognitive Psychology*, **11**(3), 346–374.

Krumhansl, C. L. (1990). *Cognitive foundations of musical pitch*, New York: Oxford University Press. doi:10.1121/1.404005

Krumhansl, C. L., Toivanen, P., Eerola, T., Toiviainen, P., Järvinen, T., & Louhivuori, J. (2000). Cross-cultural music cognition: cognitive methodology applied to North Sami yoiks. *Cognition*, **76**(1), 13–58.

Kubovy, M., & Howard, F. P. (1976). Persistence of a pitch-segregating echoic memory. *Journal of Experimental Psychology: Human Perception and Performance*, **2**(4), 531–537.

Kurth, E. (1931). *Musikpsychologie*, Berlin: Max Hesses Verlag.

Larson, S. (1997). The Problem of Prolongation in “Tonal” Music: Terminology, Perception, and Expressive Meaning. *Journal of Music Theory*, **41**, 101.

Lerdahl, F., & Jackendoff, R. S. (1985). *A Generative Theory of Tonal Music*, Cambridge, MA: MIT Press.

Lester, J. (1977). Major-minor concepts and modal theory in Germany: 1592-1680. *Journal of the American Musicological Society*, **30**(2), 208–253.

Lieberman, P. (1966). *Intonation, perception, and language*. *M.I.T. Research Monograph*, Massachusetts Institute of Technology, Cambridge, MA. Retrieved from http://hdl.handle.net/1721.1/12996

Lippman, E. A. (1964). *Musical Thought in Ancient Greece*, New York: Da Capo Press.

Mazel, L. (1952). *On melody [О мелодии]*, Moscow: Gos Muz Izdat [State Musical Publishing].

Mazel, L. (1972). *Problems of Classical Harmony [Проблемы классической гармонии]*, Moscow: Muzyka.

Mazel, L. (1982). On certain aspects of Asafyev’s concept [О некоторых сторонах концепции Б.В. Асафьева]. In I. Prudnikova, ed., *Essays on theory and analysis of music [Статьи по теории и анализу музыки]*, Moscow: Soviet Composer, pp. 277–307.

Mertens, P. (2004). The Prosogram: Semi-Automatic Transcription of Prosody Based on a Tonal Perception Model. In *Proceedings of the 2nd International Conference on Speech Prosody*, pp. 549–552.

Mertens, P. (2013). Automatic labelling of pitch levels and pitch movements in speech corpora. In B. Bigi & D. Hirst, eds., *Proceedings of Tools and Resources for the Analysis of Speech Prosody, Aix-en-Provence, August 30, 2013*, Aix-en-Provence, France: TRASP, pp. 42–46.

Mertens, P., Beaugendre, F., & D’Alessandro, C. R. (1997). Comparing Approaches to Pitch Contour Stylization for Speech Synthesis. In *Progress in Speech Synthesis*, New York, NY: Springer New York, pp. 347–363.

Narmour, E. (1992). *The Analysis and Cognition of Melodic Complexity: The Implication-Realization Model*, Vol. 50, Chicago, IL: Chicago Review Press.

Nazaikinsky, Y. V. (1973). On Constants in Perception of Music [О Константности в Восприятии Музыки]. In Y. V Nazaikinsky, ed., *Musical Art and Science [Музыкальное искусство и наука]*, Vol. 2, Moscow: Muzyka, pp. 59–98.

Nikolsky, A. (2015). Evolution of tonal organization in music mirrors symbolic representation of perceptual reality. Part-1: Prehistoric. *Frontiers in Psychology*, **6**(1405). doi:http://dx.doi.org/10.3389/fpsyg.2015.01405

Nikolsky, A. (2016). Evolution of Tonal Organization in Music Optimizes Neural Mechanisms in Symbolic Encoding of Perceptual Reality. Part-2: Ancient to Seventeenth Century. *Frontiers in Psychology*. doi:10.3389/fpsyg.2016.00211

Nikolsky, A., Alekseyev, E. Y., Alekseev, I. Y., & Dyakonova, V. E. (2020). The overlooked tradition of ‘personal music’ and its place in the evolution of music. *Frontiers in Psychology*, **10**(Feb.), 3051.

Nikolsky, A. V. (1926). Scales of folk song [Звукоряды народной песни]. In A. V. Nikolsky, ed., *The collection of works of the ethnographic department of GIMN [Сборник работ этнографической секции ГИМНа]*, Vol. 1, Moscow: State Institute of Musical Science.

Noorden, L. van. (1975). *Temporal Coherence in the Perception of Tone Sequences*, Vol. 3, Eindhoven, Holland: Institute for Perceptual Research.

Ogolevets, A. (1941). *The Basics of Harmonic Language [Основы гармонического языка]*, Moscow: Muzgiz.

Parncutt, R., & Cohen, A. J. (1995). Identification of microtonal melodies: effects of scale-step size, serial order, and training. *Perception & Psychophysics*, **57**(6), 835–846.

Patel, A. D. (2006). An empirical method for comparing pitch patterns in spoken and musical melodies: a comment on Pearl’s “Eavesdropping with a master: Leos Janácek and the music of speech". *Empirical Musicology Review*, **1**(3), 166–169.

Patel, A. D. (2010). *Music, Language, and the Brain*, Oxford; New York: Oxford University Press.

Powers, H. S., & Wiering, F. (2001). Mode. The term. Medieval modal theory. Modal theories and polyphonic music. In S. Sadie & J. Tyrrell, eds., *The New Grove Dictionary of Music and Musicians*, Stanley, London, UK: Macmillan Publishers. doi:10.1093/gmo/9781561592630.article.43718

Protopopov, S. (1930). *Elements of construction of musical speech [Элементы строения музыкальной речи]*. (B. Yavorskii, Ed.), Vol. 1, Moscow: State Edition, Musical Sector [Госуд. Изд-во Музык. Сектор].

Rags, Y. N. (1999). *Aesthetics from the bottom and aesthetics from the top - quantitative ways of approaching [Эстетика снизу и эстетика сверху – квантитативные пути сближения]*, Moscow: Nauchnyi mir.

Rakowski, A. (2004). From acoustics to psychology: Pitch strength of sounds. In *The music practitioner: Research for the music performer, teacher and listener*, Burlington, VT: Aldershot, Ashgate, pp. 67–78.

Ramos, D., Bueno, J. L. O., & Bigand, E. (2011). Manipulating Greek musical modes and tempo affects perceived musical emotion in musicians and nonmusicians. *Brazilian Journal of Medical and Biological Research*, **44**(2), 165–172.

Revithiadou, A. (1999). *Headmost accent wins: Head dominance and ideal prosodic form in lexical accent systems*, Rutgers, Rutgers, NJ. Retrieved from https://rucore.libraries.rutgers.edu/rutgers-lib/38604/

Riemann, H. (1896). Mode. In J. S. Shedlock, ed., *Musik-Lexikon [Dictionary of music]*, London, England, p. 505.

Riemann, H. (1916). *Ideen zu einer “Lehre von den Tonvorstellungen,”* Leipzig: C. F. Peters.

Roederer, J. G. (2008). *The Physics and Psychophysics of Music: An Introduction*, Berlin, Heidelberg: Springer Science & Business Media.

Roitershtein, M. (1973). Graph and matrix as instruments of modal analysis [Граф и матрица как инструменты ладового анализа]. In Y. V. Nazaikinsky, ed., *Musical Art and Science [Музыкальное искусство и наука]*, Vol. 2, Moscow: Muzyka, pp. 175–189.

Rothfarb, L. A. (1979). *Ernst Kurth’s The Requirements for a Theory of Harmony: an annotated translation with an introductory essay*, University of Hartford, Hartford, CT.

Rothstein, W. N. (1989). *Phrase rhythm in tonal music*, New York, NY: Schirmer Books.

Rubtsov, F. (1964). *The foundations of modal morphology of Russian traditional songs [Основы ладового строения русских народных песен]*, Moscow: Muzyka.

Rudneva, A. (1994). *Russian traditional musical works: essays on the theory of folklore [Русское народное музыкальное творчество: очерки по теории фольклора]*, Moscow: Kompozitor.

Scherbaum, F. (2016). On the benefit of larynx-microphone field recordings for the documentation and analysis of polyphonic vocal music. *Proc. of the 6th International Workshop Folk Music Analysis,15 - 17 June, Dublin/Ireland*, 80–87.

Skrebkov, S. (1967). Intonation and mode [Интонация и лад]. *Sovetskaya Muzyka*, (1), 89–94.

Skrebkov, S. (1973). *Artistic principles of musical styles [Художественные принципы музыкальных стилей]*, Moscow: Muzyka.

Smith, L. D., & Williams, R. N. (1999). Children’s artistic responses to musical intervals. *The American Journal of Psychology*, **112**(3), 383–410.

Smith, N. A., & Schmuckler, M. A. (2004). The perception of tonal structure through the differentiation and organization of pitches. *Journal of Experimental Psychology. Human Perception and Performance*, **30**(2), 268–86.

Snow, D. (2006). Regression and Reorganization of Intonation Between 6 and 23 Months. *Child Development*, **77**(2), 281–296.

Starostina, T. (1973). Modal systematization of Russian traditional song [Ладовая систематика русской народной песни]. In *Harmony: problems of science and methodology [Гармония: Проблемы науки и методики]*, Vol. 1, Moscow: Muzyka, pp. 85–105.

Stefanics, G., Háden, G. P., Sziller, I., Balázs, L., Beke, A., & Winkler, I. (2009). Newborn infants process pitch intervals. *Clinical Neurophysiology*, **120**(2), 304–8.

Stockmann, D. (1994). Synthesis in the culture of scholarship: problems in investigating and documenting the archaic and modern styles of yoiking by the Sami in Scandinavia. In M. J. Kartomi & S. Blum, eds., *Music-Cultures in Contact: Convergences and Collisions*, Basel, Switzerland: Gordon and Breach Publishers, pp. 1–12.

Straehley, I. C., & Loebach, J. L. (2014). The influence of mode and musical experience on the attribution of emotions to melodic sequences. *Psychomusicology: Music, Mind, and Brain*, **24**(1), 21–34.

Sussman, E., Horváth, J., Winkler, I., & Orr, M. (2007). The role of attention in the formation of auditory streams. *Attention, Perception, and Psychophysics*, **69**(1), 136–152.

Tchesnokov, P. (1961). *Choir and conducting of it [Хор и управление им]*, Moscow: Gos Muz Izdat [State Musical Publishing].

Temperley, D. (2008). Hypermetrical Transitions. *Music Theory Spectrum*, **30**(2), 305–325.

Tomasello, M. (2003). *Constructing a language: a usage-based theory of language acquisition*, Cambridge, MA: Harvard University Press.

Trochidis, K., & Bigand, E. (2013). Investigation of the Effect of Mode and Tempo on Emotional Responses to Music Using EEG Power Asymmetry. *Journal of Psychophysiology*, **27**(3), 142–148.

Tzukkerman, V. (1965). The Integrative Analysis of Music Works and Its Methodology [Целостный анализ музыкальных произведений и его методика]. In B. M. Yarustovsky, ed., *Intonation and musical imagery: essays and research of the musicologists of the Soviet Union and other Socialist States*, Moscow: Muzyka, pp. 264–320.

Tzukkerman, V. (1975). *Glinka’s Kamarinskaya and its traditions in Russian music [«Камаринская» Глинки и её традиции в русской музыке]*, Moscow: Soviet Composer.

Vakhromeyev, V. A. (1981). Trill [Tрель]. In Y. V. Keldysh, ed., *Musical Encyclopedia [Музыкальная энциклопедия]*, Vol. 5, Moscow, Russia: Soviet Encyclopedia, pp. 595–6.

Vuvan, D. T., Prince, J. B., & Schmuckler, M. A. (2011). Probing the Minor Tonal Hierarchy. *Music Perception: An Interdisciplinary Journal*, **28**(5), 461–472.

Wallin, N. L. (1983). Pitch Perception as Expression for Exogene and Endogene Coordinated Oscillations. *The World of Music*, **25**(3), 46–64.

West, M. L. (1981). The Singing of Homer and the Modes of Early Greek Music. *The Journal of Hellenic Studies*, **101**, 113–129.

West, M. L. (1992). *Ancient Greek music*, New York, London: Oxford University Press.

Wienpahl, R. W. (1959). Zarlino, the Senario, and Tonality. *Journal of the American Musicological Society*, **12**(1), 27–41.

Yavorskii, B. (1908). *The construction of musical speech. Data and notes [Строение музыкальной речи. Материалы и заметки]*, Vol. 1, Moscow: Aralov, G.

Zsiga, E. C. (2013). *The sounds of language: an introduction to phonetics and phonology*, Oxford UK: Wiley-Blackwell.

1. It is possible to apply this analysis to multi-part music in the manner Frank Scherbaum (2016) used the TONY pitch tracking algorithm to establish the melodic interval set in 3-part Svan songs by recording each of individual parts with the help of a larynx microphone. The TO of each constituent musical part can be graphically represented in combination with the other constituent parts, following the XYZ projection proposed in the Fig.1 of the main article. [↑](#footnote-ref-1)
2. The concept of “intervallic typology” is thoroughly explained in the Appendix-1 “Taxonomy of tonal organization of modal music” (Nikolsky, 2015). The structural features of all known intervallic typologies are listed in the Appendix-6 “Comparison of the principal features in types of tonal organization” (Nikolsky, 2016). [↑](#footnote-ref-2)
3. This term was introduced by Yurii Kholopov (1988, 117) for classification of the existing intervallic typologies in reference to music that is constructed from the tones of indefinite pitch or tones fluctuating in pitch values in repetitions of the same musical pattern. Kholopov rebranded this term from the Ancient Greek treatises on music theory by Cleonides, Gaudentius, and Ptolemy, who used it to refer to non-melodious, disproportionate, and unclear sounds—in opposition to the term “emmelic,” introduced by Aristoxenus in relation to melodious and well-tuned musical tones (Lippman 1964 p. 160). [↑](#footnote-ref-3)
4. “Frequency change between the words” applies to the change in pitch between the end of one word and the beginning of another—not between the beginnings of two adjacent words. [↑](#footnote-ref-4)
5. Thus, G# in a harmonic A-minor mode constitutes a characteristic modal degree due to its dissonant tritone relation to D, which requires resolution of tension, and a weird sounding gap of the augmented 2^nd^ in relation to the neighboring F. The ending of the antecedent phrase on F-E-G# and of the consequent phrase on F-E-A would define the A Minor key, because G# executes the role of the “leading tone”—a characteristic degree for the harmonic A minor. [↑](#footnote-ref-5)
6. Each of these anchoring levels corresponds to its own preferential method of TO: 1) – to ekmelic, 2) – to emmelic oligotonal, and 3) – to mesotonal, multitonal and diatonic modes (Nikolsky 2015). The ongoing alternation of two tonic anchors usually engages I–VI or I–II degrees in a heptatonic and I–V degrees in a pentatonic mode, and is known as “double-tonic” in Western musicology (Gelbart 2013) and “modal mutability” (“ladovaya peremennost”) in Russian musicology (Kholopov 1988)—first explained by Boleslav Yavorsky in 1908 (Yavorskii 1908). [↑](#footnote-ref-6)
7. It is also plausible that the emergence of musical ascent followed the emergence of musical arch. The impulse to ascend naturally suits the very beginning of the breathing cycle, when oxygen is plentiful, and is replaced by the impulse to descend during the expiration phase. The earliest forms of TO might have started with this physiologically driven scheme and thereafter segment the ascending opening to use it as a stand-alone phrasal model. However, Alekseyev considers this scenario less likely, since in order for the semiotic use of inclinations to emerge, music users must have discovered the activating properties of the ascending shape and associated them with the inspiration of it as an opposition to the relaxing properties of the descending shape and expiration. Such a discovery is unlikely in an arch-shaped phrase that abides by the physiological instincts of breathing and therefore is experienced as something “normal”: the very normalcy of binding the inspiration with activation, and the expiration with relaxation, should be expected to preclude the discovery of inherent capacity of ascending inclination to increase, and of descending inclination to reduce tonal tension. [↑](#footnote-ref-7)
8. If necessary, the audio can be slowed down (good software editors like iZotope RX allow for up to 10x slowing while preserving the timing of the transients rather well) or scrubbed to establish the precise onset of a glissando in order to select the most tonally stable part of a sound for frequency analysis. Then, the use of vibrato, embellishments, and tremolos can be accurately identified, and their average pitch value can be adequately estimated. [↑](#footnote-ref-8)
9. Sámi yoiks present an example of devaluation of melodic dissonance, where the threshold of a leap is pushed higher than a 3^rd^ because of the overall importance of leaps for the genre of yoik (Krumhansl *et al.* 2000). Commonality of long leaps for yoiks might have the environmental origin: large leaps performed with harsh and loud voice characterize not yoiks of Eastern valleys but yoiks of mountainous Western Eanodat region that has been less accessible and therefore less influenced by the Lutheran style singing (Jouste 2009). Contribution of echo in wide open space to shamanic enchanting that must have underlain yoiking (Stockmann 1994) required sharpening of contrasts between the tones of yoik’s pitch set—to make the melodic contour more clear over longer distance. However, such “emancipation” of melodic dissonance (essentially similar to that of Schoenberg’s idea of emancipation of dissonance in harmony) must be statistically rare enough to consider it an exception rather than a rule for the genesis of TO. [↑](#footnote-ref-9)
10. For the distinction between modality and tonality, see the chapters: “Investigation of Melodic Harmony: Mode and Intonation” and “Modality vs. Tonality” (Nikolsky 2016). [↑](#footnote-ref-10)
11. The term “premelic” here refers to such musical mode that *integrates sounds by means of their tonal similarity in timbre, register, loudness and articulation—without reliance on the frequency aspect of organization*. Such mode is hypothesized to precede “melodic” organization in strict sense of the term “melody”—as progression of well-matched “harmonious” changes in frequency. The earliest forms of musical vocalizations by infants and certain forms of timbre-based indigenous traditional music can be qualified as “premelic” TO. [↑](#footnote-ref-11)
12. See Appendix 6: “Comparison of the principal features in types of tonal organization” for more details on transposition (Nikolsky 2016). [↑](#footnote-ref-12)
13. Roitershtein used a black box to fill up the cell of the same vertical and horizontal nomination (e.g., 1x1, 2x2 and 3x3). Goshovsky employed a dash for the same purpose. However, there is no need in doing these, since the intersection would indicate an interval of a melodic unison (ƒ-anc) at any rate. [↑](#footnote-ref-13)
14. In English musicology, it is not uncommon to find definitions of the “leading tone” as a chromatic alteration of the VII degree, accidental in its nature (Clough 1957). In contrary, Russian modal theory generally sides with German musicology in adopting the arguments of Riemann (Riemann 1916 pp. 1–16) and Helmholtz (Helmholtz 1877 pp. 299–300) that modification of the VII degree in minor is not “accidental,” but is caused by general systemic relations between the degrees of a key. From a melodic perspective, even within the classical music repertoire, there are compositions entirely sustained in harmonic minor (Schubert's song “Der Leiermann”) or natural minor (the choir "Why have you forsaken us, our father" [Na kogo ty nas pokidaesh] from Prolog to the opera “Boris Godunov” by Mussorgsky), which clearly should not be regarded as a mere collection of accidental alterations of the VII degree but rather as different musical modes. Thus, the use of the natural minor mode makes two compositions sound alike (e.g., compare Mussorgsky’s choir with Israeli Hatikva). [↑](#footnote-ref-14)
