# Supplementary material for: The Pastoral Origin of Semiotically Functional Tonal Organization of Music
Source: Front Psychol. 2020 Jul 23;11:1358. doi: 10.3389/fpsyg.2020.01358 (PMC7396614; doi:10.3389/fpsyg.2020.01358)
Supplement: DATA SHEET S2 — Appendix 2 – A comparative structural analysis of musograms used in Figures 3, 4, 7 of this article. This document contains a comprehensive analysis of the characteristic traits of tonal organization in the examples of human musical communication, animal vocal communication, and bi-specific communication between domestic animals and their human guardians. [file Data_Sheet_2.docx]

# **Comparative structural analysis of musograms.**

## Abstract

This document presents a full multi-factorial analysis of tonal organization in the audio examples that were used for Figures 3, 4 and 7 in the article “The pastoral origin of semiotically functional tonal organization of music.” All musicological analysis there did not go beyond the most basic entry level—sufficient to identify the patterns of expression, characteristic to each of the aspects of expression (AEs) in pretonal forms of human music, animal calls, and human-to-animal communication. Appendix-1 “Method of modal multifactorial analysis of tonal organization in music” covers the explanation of the principles of modal analysis, its procedure and its ways to quantify the acoustic measurements for the identification of the musical mode in a studied piece of music. Appendix-1 uses the single example of the traditional Yakut lyrical song “Sae Dyige” (in 2 versions, the first of which was used as Figure 2 in the article “The pastoral origin of semiotically functional tonal organization of music”). Three other audio examples, analyzed in this appendix, do not involve modal analysis, since the article focuses on the interaction of structural patterns of multiple EAs rather than on problems of inferring specific frequency modes. Therefore, in Appendix-2, I skip the generation of tables and matrix, necessary for identifying the modal degrees and their functionality. Below, I will characterize the multi-factorial patterns, evident in musograms of the above-mentioned 3 audio examples, and show how the conclusions stated in the main article were actually derived from the musogram analysis of these 3 examples.

Table of Contents:

[1. Expression of anger versus expression of love in Yakut traditional solo songs 1](#_Toc40825073)

[2. Expression of anger in a Yakut traditional song versus gorilla’s calls 3](#_Toc40825074)

[3. The genesis of musical mode through changes in distance in vocalizations of kulning 5](#_Toc40825075)

[Medium-distance kulning (A) 5](#_Toc40825076)

[Close-distance kulning (B) 7](#_Toc40825077)

[Longer-distance kulning (C) 8](#_Toc40825078)

[Maximal-distance kulning (D) 10](#_Toc40825079)

[The interaction of all 4 kulning styles 15](#_Toc40825080)

#### Expression of anger versus expression of love in Yakut traditional solo songs

I will start with the musicological analysis of the expression of anger in the traditional epic song from the Yakut olonkho **“**Djiribina Djirilatta**”** (Fig.1) and compare it to the expression of love in “Sae Dyige” (Fig.2). For the conventions of visualization in a musogram representation of an audio clip, see the legend for Figure-1 in the main article “The pastoral origin of semiotically functional tonal organization of music,” and Chapter 6 “Graphic melodic chart” in Appendix-1 “Method of modal multifactorial analysis of tonal organization in music.”


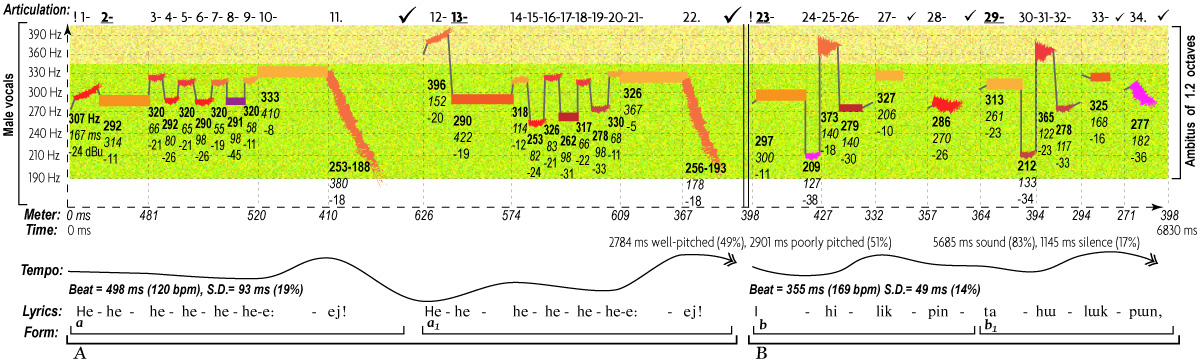


**Figure-1**. Structural analysis of 10 AEs in the expression of aggression and anger in a traditional Yakut epic song of the underworld virgin (<http://chirb.it/sCq02k>).


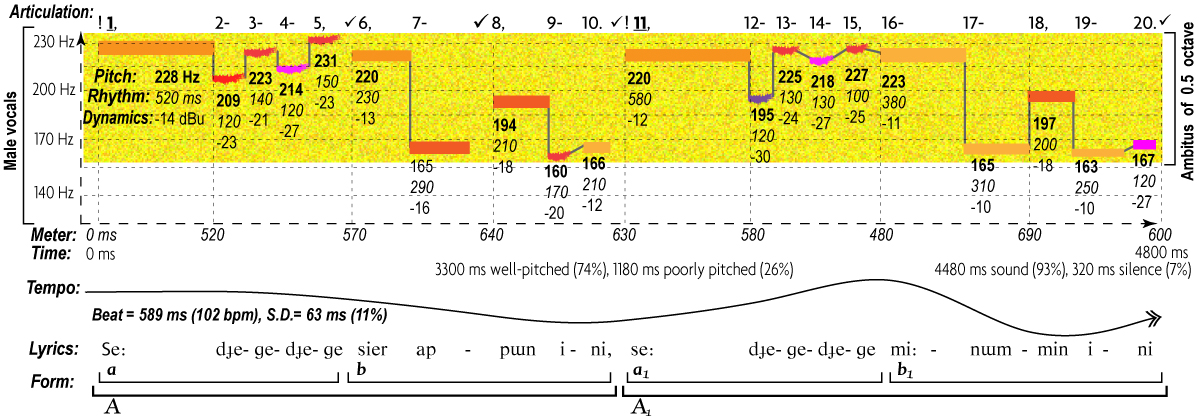


**Figure-2**. Structural analysis of 10 AEs in the expression of love in a traditional Yakut lyrical song “Sae Dyige” (<http://chirb.it/sNegG1>).

Each AE in the expression of anger (Fig.1) is characterized by patterns that are quite different from the expression of love (Fig.2). The ambitus of the melody is more than twice wider (the interval of a 9^th^ rather than a tritone, as in the love song). There are 2 registers instead of just 1 as for the love song. The upper register in the expression of anger is narrower (333–396 Hz, or 300 c, i.e., minor 3^rd^) and more homogenous in its inharmonious “shouting” quality. The lower register is more harmonious yet distinctive in timbral contrast between the long well-sustained pitches, short pitches, and the cadential glissando pitches that slide through a much wider range (188–333 Hz, or 990 c, i.e., minor 7^th^). Both registers are pushed significantly higher than in the love song (most pitches here fall within the range of 253–333 Hz of the male voice vs. 160–230 Hz of Fig.2—about an octave higher). Higher position introduces tension, harshness and “thinness” to the sound quality.

Of all the sounds of the melody, only 49% is well-pitched (i.e., manifest clear sustained pitch value)—in contrast to the love song (Fig.2), where 74% of the sounds were well-pitched. Of the well-pitched tones, 23% belong to the tonic anchor at the beginning of each motif of the angry song versus 35% for the love song. The share of staccato articulation is increased for the expression of anger: 83% of the clip is made of sounds and 17% of silence (vs. the respective values of 93% to 7% ratio for the love song), with 7 pauses (versus only 5 pauses in Fig.2). Two last tones in the “B” material (Fig.1) are cut staccato. Frequent repetition of “B” as a part of melodic formula further emphasizes staccato in expression of anger. Tones here are overall shorter and more diverse in their time values, readily forming contrasts between rhythmic groups: the angry song uses 6 rhythmic values—a 50% increase over 4 values of the love song.

The tempo in Fig.1 is overall faster: on average, 120 bpm for the slower introduction and 169 bpm for the section based on the melodic formula—versus 102 bpm for the love song. The tempo of the angry song is also less stable: 19% of fluctuation vs. 11% for the love song, plus clear presence of 2 contrasting tempi in the expression of anger (marked in the musogram by the double-bar vertical line at the 4 sec mark). As the angry song progresses, the tempo accelerates even further (especially, after 33 sec—not reflected by this chart, but audible in the sound clip). High volatility of tempo is evident if to compare the tempo curve in Fig.1 to Fig.2.

Intonations of the angry song feature wide leaps: they descend by 539, 608, 503, 675, and 471 cents and ascend even more, by 1003, and 941 cents—which makes the mean of 677 cents, or almost a 5^th^. These leaps on average are 70% wider than in the love song: there, we had only the descending leaps of 498, 334, 521, 307, and 328 cents—which gave the mean of 398 cents, or major 3^rd^. Angry expression also increases the variability of leaps by about twice—the S.D. of 197 cents versus 92 cents of the love song. Greater variety of intervals (from 4^th^ to major 6^th^) make angry leaps less predictable and more impulsive. The share of ascending leaps keeps increasing towards the end of the angry song, since the “B” material is sustained for the most of this song. A very active character is projected by strong contrasts between the animated stepwise motion and elongated descending slides at the end of the “A” phrases, including tremolo with a rate of 396 cents (the interval of a major 3^rd^).

Thematically, the angry music is more diverse and complex than the love music: it uses two contrasting materials, melodically smooth yet rhythmically active “A” and extremely zigzagged “B” (the love song had only one homogenous material “A” that was gentle swinging). Singing this song requires harsh timbre, heightened larynx position, and forceful delivery (Alekseyev and Nikolayeva, 1981, 35). Structural descriptors of most aspects of this song fall in the category of “angry” music (Juslin, 2005)—in contradistinction to the humoristic “love song” of Fig.6 (Alekseyev and Nikolayeva, 1981, 86).

- Noteworthy, aspects of rhythm, meter, tempo, articulation and pitch contour change altogether towards a *greater activity* in the transition from “A” to “B”: speeding of tempo is accompanied by shortening of the longest tones, stabilization of meter, introduction of staccato-gaps at the end of the formula, and switching from the apex-tremolo-nadir shape of “A” to the zigzagging down-up-down-up shape of “B.”

#### Expression of anger in a Yakut traditional song versus gorilla’s calls

Evidently, expression of anger engages very different patterns of TO for each of the AEs than does the expression of love does. Even greater differences separate human (Fig.1) and animal expression of anger (Fig.3). The most drastic difference is in the use of registration. Human vocalizations, as a rule, differentiate sounds in register by adopting rather strict frequency threshold tones whose FF exceeds a certain pitch level fall into a different register. Therefore, register in music can be defined by its bandwidth. As long as a tone’s FF fits into the band of a given width, this tone retains timbral similarity to other tones of the same bandwidth. Unlike such band-driven registration, gorilla’s calls break into different registers based on the differences in sound production rather than threshold FF values. Sounds that are produced by different mechanisms of vocalization become registrally different despite falling within the same frequency range. This is more similar to some of human musical instruments rather than to human vocals: e.g., strings can produce pizzicato (i.e., plucking) and arco (i.e., bowing) sounds that registrally differ not in pitch but in sound-production technique. Thus, plucking a string versus bowing it generates a distinctly different tonal quality while maintaining exactly the same pitch value; moreover, the very same division of ambitus in registers that applies to arco, applies to pizzicato as well (Turetzky, 1974, 6).


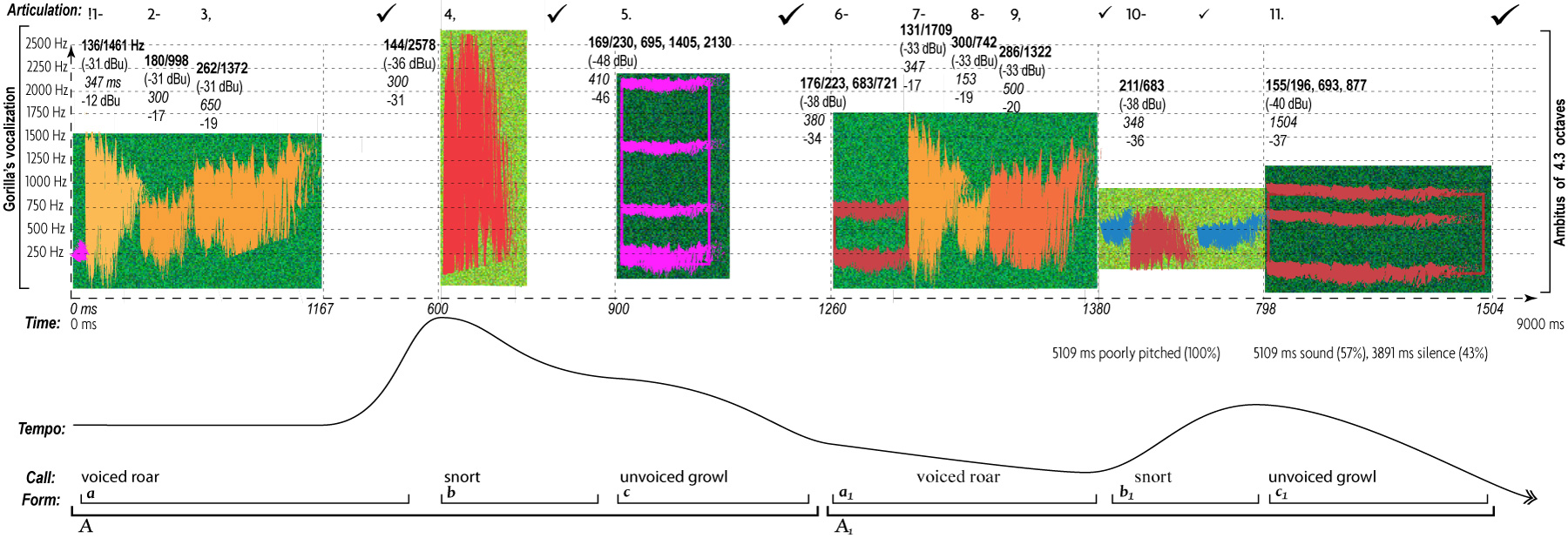


**Figure-3**. Structural analysis of angry gorilla’s calls according to the principles of TO in human music (<http://chirb.it/72g63y>). Multifactorial analysis of primate’s vocalizations reveals important differences in mapping the expression of a similar affective state to structural patterns of each of the AEs.

Gorilla’s vocalization consists of 3 types of calls: voiced roar, non-voiced growl and snort. None of these possess salient pitch contours due to their extremely broad bandwidth. Perhaps, it would be more appropriate here to talk about “band envelops” in reference to the little changes in the melodic shape of the voiced sounds, because the melodic contour of the highest portion of their spectrum does not perfectly align with the contour of the lowest portion (e.g., the upper end of “A” makes a convex shape, whereas its lower end has an ascending shape). Contrasts in size of “band envelops” play a key role in distinguishing one call type from another. Voiced roar starts by narrowing extremely broad signal (136 to 1461 Hz, i.e., 3.4 octaves wide) by about twice (trimming it to 2.5 octaves) and transposing it higher (by about a 5^th^ up) while keeping the same bandwidth (2.4 octaves). The bandwidth here is calculated by taking measurements of the frequency of that portion of a spectrum which stands out from the rest of the signal, as evident by auditioning the material and by examining the frequency analysis graph generated by the software RX Pro by iZotope. Thus, the complex broad band of roaring reaches -31 dBu. Snorting is more dynamically and temporally dispersed: it is 24% broader (143-2578 Hz, i.e., 4.2 octaves)—but nearly quadruple briefer (300 ms rather than 1300 ms of the opening roar) and 14% quieter (-36 dBu). On the other hand, growling is dynamically dispersed even to a greater extent (-46 dBu, or 33% softer), while being harmonically “condensed.” It differs from roaring and snorting by selectively amplifying a handful of partials, which makes it more harmonious—although still without defining a clear FF, due to a rather broad base (169–230 Hz, i.e., 0.5 octaves) and quite obvious de-emphasis of numerous harmonics in a harmonic row.

Each of the call-types forms its own register so wide that there is simply no place left for a frequency threshold within the critical bandwidth of hearing. Instead, calls are distinguished by differences in their tonal quality:

- prolonged deep, rich and full-bodied loud roar;
- breathy and explosive but brief and quiet snort;
- noisy yet more clearly pitched growl.

In effect, their contrasts resemble more of *timbre-classes of timbral music* (Nikolsky et al., 2020) than pitch-classes of human music. However, each call-type here can be regarded not only as a degree in a timbral mode, but also as *a specific thematic material*. The same succession of roar-snort-growl is repeated over and over with variations in bandwidth and occasional insertion of brief breathing noises in between. In essence, conservation of such patterns of succession is no different from assembling motifs from melodic intonations—except that, unlike motifs and intonations of music, such calls neither merge together to form new compound structures nor generate distinct variations in pitch or rhythmic increments. In regard to the aspect of form (i.e., complexity of the thematic material), each of the gorilla’s elementary calls remains isolated in reproductions of a well-conserved “a,” “b,” and “c” (at the absence of lyrics, punctuation in numbering becomes the primary means of indicating articulation in a musogram: dash shows connectedness, comma—disconnectedness, dot—end of the thematic pattern). The overall prevalence of disconnectedness in gorilla’s vocalizations is most obvious from estimating the share of silence—43% (vs. meager 7% of Fig.2 and 17% of Fig.1)—as well as the exceedingly low rate of pitch changes—one change per 818 ms (vs. 240 ms for Fig.2 and 200 ms for Fig.1).

Rhythm and meter in gorilla’s calls are characterized by the same tendency for isolation of discrete elements, which is as pronounced as thematic and registral isolation of elements. Onsets of each of the calls generates a pulse, functionally equivalent to human meter. However, the rate of this pulse is about twice slower than in the angry human music (Fig.1), and its deviation from regular pulse is nearly twice greater—exceeding even a slow and flexible “loving music” (Fig.2). In gorilla’s vocalizations, neither temporal organization, nor pitch (100% of tones are poorly pitched) participate in TO—in complete opposite to human music.

- *Each gorilla’s call remains a “thing-in-itself”, mechanically juxtaposed with other calls and contrasting them along a single axis of pitch* (specifically, in one of the most common pair of frequency-related parameters: bandwidth and direction of “spectral envelop”).

#### The genesis of musical mode through changes in distance in vocalizations of kulning

Finally, we can proceed to the analysis of the human-to-cattle communication in kulning. Since kulning fundamentally lacks metric organization, following the prototype of animal communication in its disregard for any concerns for regularity and proportionality of rhythmic values, its musograms omit the tempo curve, and the vertical dash line indicates not metric stresses but the onset of motifs. This is to facilitate the comparative analysis of all audio clips, even if they have ametric design (all 4 clips representing kulning are trimmed at about 13 sec for the ease of their cross-examination).

*Changes of thematic material remains the principal means of perceiving the regularity/irregularity of temporal TO in vocal kulning*. Therefore, tempo is calculated according to the rate of change in elementary thematic units. Numbers in italics below the vertical dash lines display the time value (in msec) of each motif. The succession of motifs determines the phrasal pulse which projects the overall sense of tempo. Average motif duration reflects how fast or slow each of 4 main types of vocal kulning proceeds, whereas its standard deviation shows how regular that tempo stays. Average tone duration reflects by how much motifs differ in regard to their prevalent rhythmic value (i.e., a motif can be long and therefore less active than a shorter motif, yet feature shorter rhythm to compensate for its extra-length and relaxation). The standard deviation of tone duration reflects how diverse the rhythmic changes within each motif are.

Lyrics are also omitted, because words are used in communication with the cattle only in recitative-like motifs during close distance interaction with the individual animals before letting the herd leave for grazing. So, the articulation aspect (indicated by the numbers above the chart) reflects only connectedness (dash sign) and disconnectedness (comma for inter-phrasal pauses and period for intra-phrasal pauses) between the herder’s syllables. Musograms of kulning display additional, more refined, information about the harmonicity or inharmonicity of each articulated sound. In addition to previously used rectangular bars, fuzzy blots, and thin grey lines, kulning musograms use new arc symbol to represent a common embellishment—a melismatic ornamental shake (usually about a step above the FF).

##### Medium-distance kulning (A)

We will start with the medium-distance kulning, because the principles of bispecific communication between humans and a species of domestic animals were probably forged at the middle distance. Wild and partially domesticated animals are not likely to let humans come close enough to engage in close-distance communication. And long-distance communication relies on auditory signals, excluding the visual and tactile clues that are accessible only in face-to-face close contacts. In order for conventions of long-distance communication to arise, humans need a long history of combined auditory, visual, and tactile stimulation of the animals, so that more obvious visual and tactile displays of human attitude would direct and promote the formation of Pavlovian style reflexes in the animal’s response to specific acoustic traits of human auditory commands. In all likelihood, mid-distance communication provided the reference for the communication at other distances in the practice of coding specific messages to domestic animals.

Medium-distance kulning (Fig.4) engages 3 types of motifs: parlando, exclamations and onomatopoeic imitations—to direct the movement of the entire herd, and occasionally to command a specific animal that does not follow the herding directions (<http://chirb.it/ntIxfM>).

Its **parlando** (motif “a”) can be recognized by a short staccato leap up and an immediate slide down that is detached from the prolonged sustained pitch (zigzags of ≈0.3 octave up and ≈1.5 octave down). The initial shorter tones are placed in the “head register,” whereas the single long tone usually belongs to the low “chest register.” Parlando often contains a simple rhythmically stressed *melisma* of syllables (usually, a single auxiliary step up, like an embellishment known in Western classical music as “shake”) in a manner that prompted the choice of the term “parlando” due to the surprising similarity of this kulning style to the “parlando” device of Western classical music. Parlando (i.e., recitativo, or, *Sprechgesang*) emerged in the monodies of the Florentine Camerata at the very end of the 16^th^ century and became popularized in secco recitatives, especially during the international spread of opera buffa in the 18^th^ century (Adler, 1965, 212). Like operatic parlando, kulning parlando aims at *animating* the vocalization in order to project the affective state necessary to motivate a specific animal to act in a particular way and highlight those acoustic features that correspond to such a way.


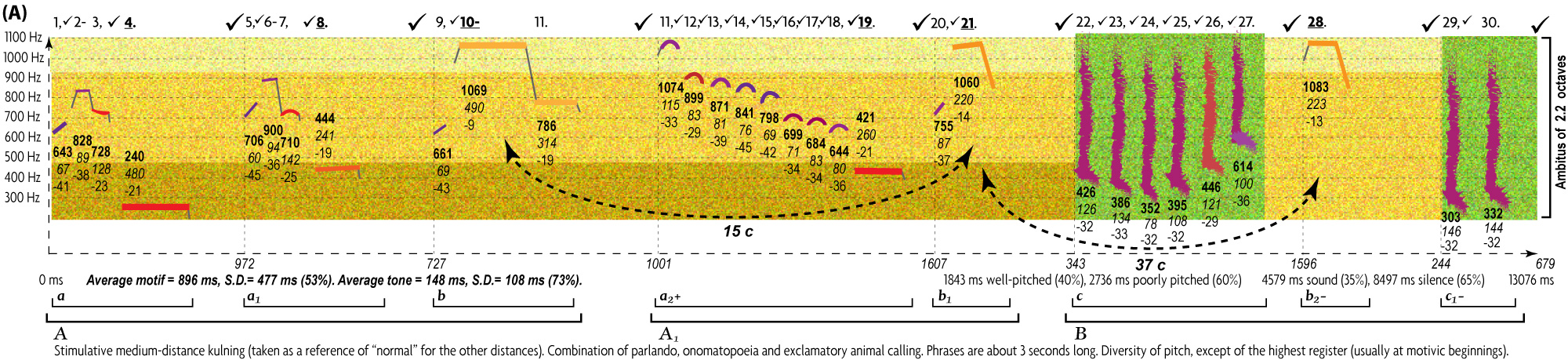


**Figure-4**. Medium distance vocalizations combine motifs of 3 types: parlando, exclamation, and onomatopoeia—employed as means to stimulate and to motivate the entire herd to follow the herder’s commands.

Medium-distance **exclamations** (motif “b”) can be recognized by a different implementation of the same zigzag shape. An ascending staccato leap, similar to “a,” leans on a long sustained pitch at the highest “shrieking” register, followed by the immediate return to the “head register” and a smaller legato leap down (0.7 octave up, 0.4 down—the reversal of the typical zigzag size in “a”). This zigzag emphasizes not the final tone in a motif, as the “normal” voice does in the parlando zigzag, but the second, “*shrieking*,” tone, thereby providing *much stronger stimulation at the opening of a motif*. Furthermore, the “shrieking” peak-tones of such exclamation motifs tend to share the same pitch value due to maximization of pitch while engaging the “closure tones” (Narmour, 1992). Equalization in pitch is reflected by the dotted double-arrows with the numbers next to them, indicating the pitch discrepancy between different occurrences of “peak-tones” in cents. Thus, b, b_1_ and b_2_ vary only by 15 and 37 cents (barely audible difference). This turns all “shrieking” tones into the *single “monotone” degree that provides a pitch reference for medium-distance kulning*.

**Onomatopoeic** imitations (motif “c”) often reproduce dog’s barking. This is a very reactive stimulus for the cattle, usually *motivating an animal to move or not move in a specific direction*. Imitation of the typical sound of an animal to whom that imitation is directed is also quite common for stirring an animal or a group of animals. Such imitations are always “timbre-oriented,” wideband, mostly nonperiodic and monoregistral.

All three types of the mid-distance motifs are strongly stimulating not only by their timbre/registration and pitch contours, which engage multiple registers and rather wide ambitus (2.2 octaves), but also by their temporal organization. Medium distance motifs are brief (mean 896 msec), use short rhythmic values (mean 148 msec) that are the least variable amongst all 4 kulning vocalization styles (the S.D. of tone duration constitutes 73% of average rhythmic value), and combine staccato articulation (24 of 30 tones of a clip are staccato, so that 65% of the clip is made of silence) with quite loud dynamics (mean -31 dBu, S.D. 9 dB).

Stimulating function is also manifested in the overall prevalence of staccato (24 of 30 tones of a clip, so that 65% of the clip is made of silence) in conjunction with the medium loud dynamics (mean -31 dBu, S.D. 9 dB), exuberant wide leaps, brief motifs (mean 896 msec) and tones (mean 148 msec), as well as the minimal rhythmic variety (S.D. of tone duration constitutes 73% of the average rhythmic value). Noteworthy, the motifs “a2” and “c” employ repetition of short staccato tones, resembling the “stimulation signal” that is universal across the animal kingdom (McConnell and Baylis, 1985). Such short repetitions constitute mostly poorly pitched tones (mean duration of 99 ms with S. D. of 28 ms) in contrast to longer tones that tend to be well-pitched (53% of the total duration of tones in this clip).

##### Close-distance kulning (B)

Close-distance kulning (Fig.5) engages 2 types of motifs: recitative and motherese-like talking—directed primarily at individual animals and supported with gestures and mimics (<http://chirb.it/8K3Lqg>). The audio clip selected to represent this type was actually recorded when the kulerska was sending the cattle off to the nearby meadow in the early morning (Wallin, 1991, 398–400). Compared to mid-distance, close-distance kulning is dynamically softer (by about 9 dB), more diverse and individualized (varying in its stimulating properties, depending on the addressee), and is more “verbal” than “musical.” In fact, close-distance kulning is the most verbal of all the kulning styles as it relies on verbal prosody to selectively approach a specific animal or a group of animals with an appropriate “suggestion.” *Close distance promotes exaggerations of melodic, rhythmic, timbral and dynamic contrasts between the consecutive sounds*.


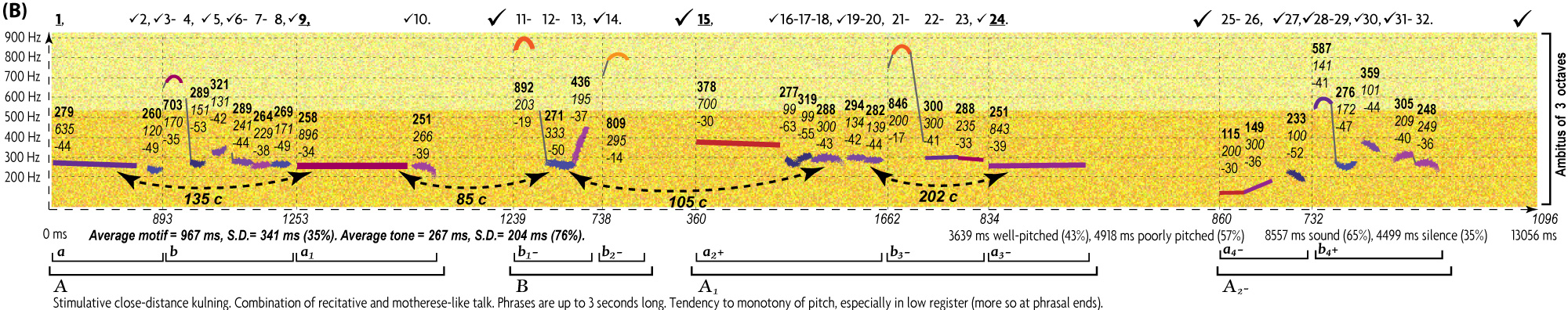


**Figure-5**. Close distance calls combine recitative and motherese-like motifs in stimulating and motivating the movement of individual animals.

**Recitative** (motif “a”) is characterized by using one long sustained pitch level (“recitative tone”) that can exceed the surrounding tones in duration by up to 5 times. It is usually separated from syllables of ordinary speech by a little caesura. This “recitative tone” is *as important for TO of close-distance kulning as the “shrieking tone” is for mid-distance kulning*. The dashed arrows show that the “recitative tone” fluctuates over 5 times more than shrieking (85-202 cents), while remaining *stable enough—within the range of a “step”*—to reference the pitch level specific for the anchor tone that constitutes the principal musical element for TO in close range kulning. Its anchor tone clearly stands out due to its super-long duration and timbre: it is explicitly sung out with a “chest voice.” The regularity of this pitch class is most pronounced at phrasal ends (e.g., A, A_1_), resembling parlando motifs of the mid-distance kulning, but less musically contrasting in articulation, register, and pitch contour—more like casual talking to oneself while being busy doing something.

**Motherese** (motif “b”) closely resembles the mid-distance parlando due to its registral contrast between the “head voice” and the “chest voice” within the same motif and its enormous melodic exaggerations, such as humongous leaps up to 1.7 octaves. Motherese motifs differ from parlando by employing many high register shake-like embellishments (represented by a colored arc shape) of a single brief opening syllable, followed by a deep slide to the lowest register of a talking voice. Not only that these slides are longer than those in parlando, but they do not involve rhythmic contrasts—all motherese tones tend to engage short non-contrasting rhythms (mean duration 211 ms, S.D. 58 ms). Most of the motherese-like material is poorly pitched: for this style, poorly pitched tones make 57% versus 43% for the well-pitched tones.

The overall articulation in close-distance kulning tends to be less disconnected than in mid-distance: of 32 tones, only 11 (34%) are staccato, and only 35% of the entire clip is made up of silence. Correspondingly, mean motif duration is 8% longer than in (A)—967 msec—and even longer for the mean tone duration (267 ms versus 148 ms—an 80% increase) due to the generous use of “recitative tones.” However, the multitude of brief rhythms makes the close-distance style similar to the mid-distance style. All in all, the former *turns out to be less sonically stimulating* than the latter: greater reliance on phonetic contrasts in words than on contrasting changes of purely musical parameters (softer dynamics, longer rhythm and phrases, fewer rhythmized repetitions of short sounds and of contrasting registers).

Closer distance removes the need in shrieking and loud barking, which could potentially startle the cattle and trigger panicky behavior. As a result, close-distance kulning shares only 2 registers (200-500 Hz “chest” and 500-900 Hz “head” voice) in common with the mid-distance kulning. So, the ambitus of close-distance kulning exceeds that of mid-distance kulning by 36% (3 octaves instead of 2.2 octaves) mainly because closer distance makes very low and quiet sounds better audible. It is within this narrow low range of 251–279 Hz (B3-C#4) that the “recitative tones” exhibit their monotony and *promote tonicity of the close-distance communication*.

The herder’s switch from close-distance to mid-distance communication, as the herd departs for the meadow grazing, inevitably causes a very obvious sonic “*modulation*” from the anchoring “recitative tones” of the low baritone-like “chest voice” to the highest “shrieking tones” that are even narrower in their range of 1060–1083 Hz (C6-C#6) of the soprano-like “head voice.” These 3-octave switches between marginal registers establish the frame of reference for the genesis of modal organization.

- *The herder, the herd and the close-by villagers become accustomed to recognizing the modal function of an anchored pitch-class according to its registral placement: the lower anchor is associated with stability, whereas higher anchor—with instability*. “Shrieking” nearly always is used to attract attention, and therefore is followed by a more important signal which prototypes modal resolution: an unstable tone is followed by a stable tone that terminates the melodic motion or the harmonic progression. On the other hand, “recitation tones” are often used to terminate a phrase/motif, which ascribes the power of modal resolution to these tones.

##### Longer-distance kulning (C)

Longer-distance kulning (Fig.6) engages 3 types of motifs: simple kula, exclamations, and parlando—directed at the entire herd to keep all the animals confident in the herder’s control over the situation while signalling that they stop grazing and come back to the herder (<http://chirb.it/n6f0sv>). This vocalization style is functionally opposite to the inherently positive mid- and close-distance communication in suggesting an inhibitive “negative” attitude to the herd: that it is no longer safe for the animals to stay outdoors. The need to secure a constructive response to the inhibitive commands while keeping animals calm to prevent panicky behavior charges the motifs of long-distance kulning with *contrasting semantics*.


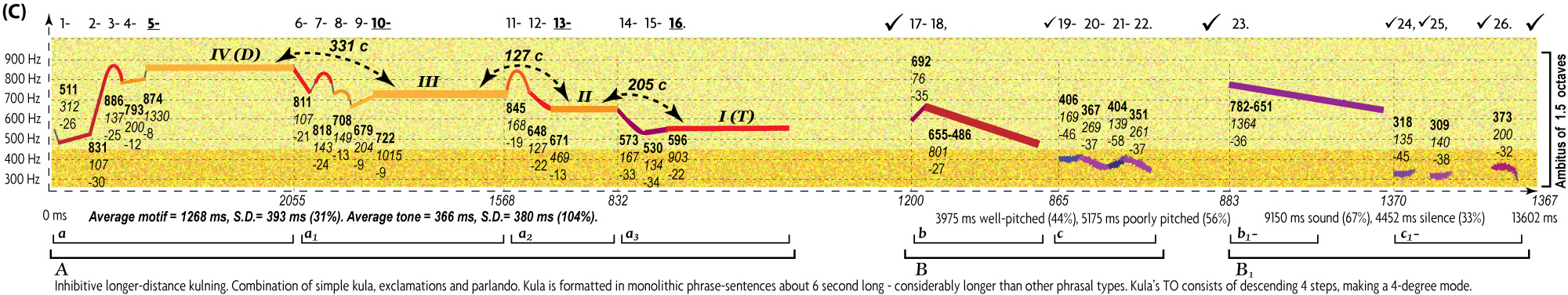


**Figure-6**. Longer distance calls combine simple kula, exclamation and parlando motifs to control the movement of the entire herd by inhibiting its further moving away from the herder, while suggesting that the herder maintains full control over the situation of the herd.

**Kula** (motif “a”) is designed to project *confidence* and *pleasure* in order to compensate for the negative connotations of the inhibitive commands delivered through exclamations and parlando. Clip (C) of longer distance kulning opens with an exemplary representation of a “kula” formula (phrase “A”). Simple “kula” typically reproduces multiple repetitions of the same formula that usually fits in a single breathing cycle: 3–5 motifs are stitched together to form a characteristic melodic shape of ascending to the crest point in the beginning of a phrase and thereafter declining towards the last phrasal tone. Therefore, each motif usually slightly varies in its melodic shape depending on its position within a phrase: the opening motif as a rule contains an ascending leap, the second motif most often steps down, but can contain the phrasal climax point in a longer kula, and the following motifs step down even lower, eventually terminating the phrase either with a slide down or a step up. In Fig.6, the motif “a” jumps a minor 7^th^ up, marking a climactic point, “a_1_” makes a little leap down (minor 3^rd^), “a_2_” steps a bit lower (minor 2^nd^) and “a_3_” provides a cadential ending by leaping a major 3^rd^ down and then bending the pitch up. This effectively generates 4 degrees. They are marked by Roman numerals. IV adopts the “dominant” function (indicated by letter “D”)—serving as an unstable anchor that requires further resolution. III and II become the supporting degrees: III provides an alternative leaning point for the melodic motion towards the “dominant,” whereas II helps the “tonic” (marked by letter “T”) to terminate the phrase.

Kula motifs are united not only by the superimposition of the *stereotypical pitch contour* over them but also by the *dynamic shape*. The envelop of a pitch contour is usually reproduced by the dynamic contour that places the greatest intensity on the climactic pitch and progressively weakens the dynamics towards the terminal longest pitch. The breathing cycle secures the stereotypicity of this dynamic wave by expanding the expiration toward its end and intensifying the inspiration at the very beginning. Such dynamic envelop is implemented rather strictly in medium-long distances, when kulerska does not have to deliver every motif at maximum volume. The resulting kula has a simple structure: a single sentence-phrase made of similar motifs. Simple kula often varies the very same motif, made up of a crop of brief shake-like embellished tones stopped by long sustained pitches (motif “a”). However, the same thematic material receives a slightly different treatment depending on its position within a phrase: “a” constitutes the only ascending motif in this phrase, “a_1_” leaps down, “a_2_” steps down, and “a_3_” leaps down and then bends up.

The ongoing reproductions of the same pitch and dynamic contour take over the verbal and visual signs of herder’s gestures and mimics as primary means of communication to the remote herd. *As a result of this purely musical organization, the entire kula phrase receives a genuine modal TO*. Its highest long-sustained anchor at the beginning of a wave forms the most unstable degree, usually a 5^th^ above the cadential anchor that makes the most stable degree. The interval of perfect 5^th^ most likely constitutes the preferred norm because it is second only to octave in its capacity to provide a “natural” guide in tuning. Listeners routinely mistake the dubbing of a melodic line in perfect 5^th^ for parallel octaves (Bukofzer, 1940) due to the highest fusion of the constituent tones in harmonic (vertical) octaves and fifths (Huron, 2001). That is why *perfect intervals* (octave, 5^th^ and 4^th^) have been indispensable throughout the entire history of tuning as opposed to imperfect intervals (2^nd^, 3^rd^, 6^th^ and 7^th^) (Barbour, 2004). Perfect intervals are identified by listeners with the highest resolution (Shackford, 1962).

The other modal degrees in kula are defined by breaking the 5^th^ in two unequal parts, producing *imperfect intervals* of a 3^rd^. The lower part is narrower because its leaning anchor on the II degree has to be closer to the terminating “tonic” in phrasal cadences to secure smooth resolution at the very end of the breathing cycle, when the expiration complicates the clarity of intonation, precluding descending leaps that are difficult to intone. The wider part of the 5^th^ (major 3^rd^) receives greater salience immediately after the climax point, which prompts the use of a more noticeable descending leap. Both thirds, major and minor, defined in this way, become the most important sources for the genesis of semantic values within the modal music system of human-to-animal communication.

- Major 3^rd^ obtains the specialization in marking climax points in kula.
- Minor 3^rd^ becomes characteristic for cadential endings of kula’s sentences.

Imperfect intervals, in general, are more diverse and expressive in their tonal qualia than perfect intervals (Cooke, 1959). And the opposition of major and minor constitutes the foundation for semantic conventions of Western European, and more generally, Indo-European musical cultures (Rice et al., 2000). The opposition of major versus minor thirds in kula probably *prototypes their cross-cultural affiliation with respectively happy versus sad affective states—via the associations set by the breathing cycle*. The point of highest physical capacity for action at the peak of oxygenation becomes associated with happiness due to the liveliness of this state, whereas the point of the lowest capacity for action at the end of the breathing cycle becomes associated with sadness due to its decline in liveliness.

Long-distance **exclamation** (“b” motif) differs from the stimulating medium-distance exclamations of the mid-distance kulning by carrying an inhibitive function. It is characterized not by an intense zigzag leaping at its onset and by a shrieking contrast, but by a gradual sliding down (about a perfect 4^th^). It is important to note that its melodic shape and soft dynamics closely resemble the “inhibition whistle,” universally employed by dog trainers (McConnell, 2002, 49–63), and most probably a common signal for many animal species (McConnell and Baylis, 1985).

Long-distance **parlando** (“c” motif), like “b,” also differs from the stimulating medium-distance parlando. It is confined to a *single register* just slightly higher than the typical range of the “recitative tones” of close-distance communication: 309–406 Hz (473 c, or D#4-G#4). This parlando stays very homogenous in its speech-like delivery, lacking melodic and rhythmic contrasts, while providing absolutely *no pitch reference*. Such simplification and homogeneity reflect the increased need for greater articulation clarity as the distance of communication increases, as well as the eagerness of a herder to beautify the vocalization in order to compensate for the negativeness of inhibitive long-distance commands. Beautification is evident in generous use of shake-like embellishments dispersed all over the kula motifs, prevalence of legato articulation (just 3 of 26 tones are staccato, and silence constitutes only 33% of the entire clip), plus the general elongation of motifs (1268 msec total—31% longer than close-distance motifs and 42% longer than mid-distance motifs).

*Longer distance forces the herder to vocalize clearer, longer, and louder*: dynamically, long distance kulning exceeds mid-distance kulning by 13 dB (-27 dBu), features the greatest dynamic diversity (S.D.=12 dB), and the longest mean tone duration (366 msec—a 37% increase over close- and 147% over mid-distance). The bulk of the melodic motion is confined to the “head register” of 500-900 Hz, mutual for all 3 distances that we have reviewed so far. The modal genesis in long-distance kulning becomes *limited to this register alone*: high “shrieking” and low “chest-voice” monotones practically disappear from this style. And all modal functionality of I, II, III, and IV degrees is exercised within a relatively narrow range of 511–874 Hz (929 cents, or C5–A5, the interval of a minor 6^th^). Together with the “chest” register, reserved for parlando, the ambitus of long-distance kulning makes a mere 1.5 octaves. This reflects a clear trend of *progressive narrowing of the ambitus with the increase of the distance of communication*.

- Moving further apart from the herd forces the herder to squeeze the ambitus and thereby consolidate the degrees of the musical mode within the tetrachord/pentachord frame. Closer intervallic proximity of degrees transpires into a greater interaction of steps with leaps, promoting the crystallization of *definite-in-pitch intervallic typology* and *specific modal functionality for each of the degrees*.

##### Maximal-distance kulning (D)

Maximal-distance kulning (Fig.7) is limited to the use of a single type of thematic material—kula—arranging its motifs in *complex* structures, directed at the entire herd and at the nearby villagers who are owners of the herded animals (<http://chirb.it/gpyC7t>). This type of vocalization gave kulning its name—maximal-distance kula is archetypical for kulning. Broadcasting signals over a kilometer is impossible without maximizing its loudness, which, in turn, requires taking multiple short caesuras throughout the span of the kula formula. This distinguishes maximal-distance kula (Fig.7) from long-distance kula (Fig.6) by making kula structures *hierarchic*: different intonations make up motifs, different motifs—phrases, and different phrases—sentences, so that a particular motif acquires greater importance than other motifs, subordinating them. Long-distance kula was missing the higher level grouping of sentences, and therefore did not distinguish between different types of cadences.

Since maximal distance kulning is restricted to just a single type of musical material that is mostly suited for the distant sound transmission, its motifs often contrast one another to provide musical versatility. This is of a special value for human listeners of kulning, because they appreciate it aesthetically (Ivarsdotter, 1986). Hence, a maximal distance kula differs from its shorter-distance cousin by heavier use of embellishments, legato, elongation of phrases and sentences, combined with complete exclusion of non-musical speech-like vocalizations. All of these makes singing appear quite virtuosic and aesthetically impressive, especially if to take into consideration that kulerska sings everything as loud as possible (mean -20 dBu, S.D. 7 dB—the smallest dynamic variability of all kulning styles) and as high as possible (625–1281 Hz, or 1 octave, D#5–D#6, which is the piccolo range that corresponds to the coloratura—a very rare and much appreciated type of voice).


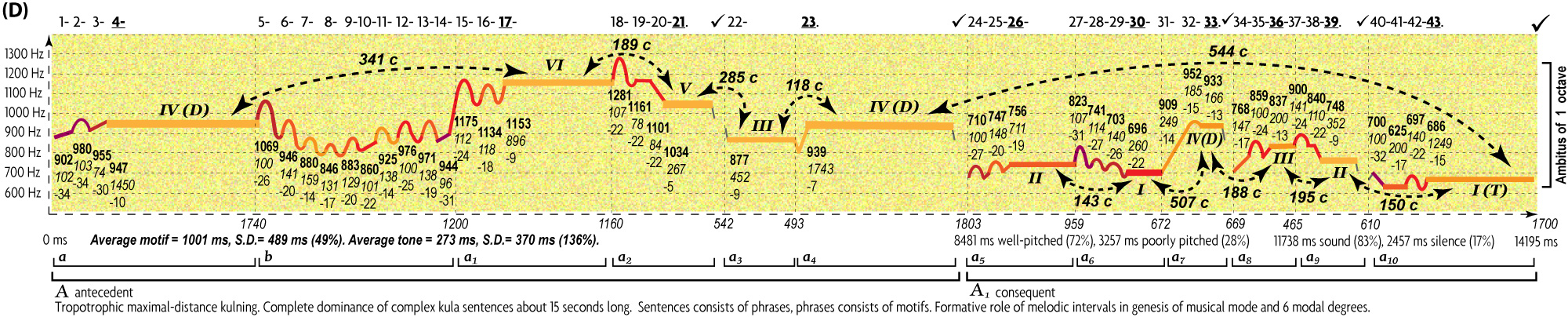


**Figure-7.** Longest distance calls use nothing but kula phrases, grouped into complex sentences, to project pleasant and relaxing impression on the animals in the herd (to motivate the herd to keep the distance in order to keep hearing kula) as well as animals’ owners in a nearby village.

Beautification and complication of kula open doors to its diversification. Thus, “a” and “b” motifs show thematic contrast: unlike “a,” “b” contains a batch of shake-like embellishments, assembled into something like a free-floating tremolo. In complex kula, each motif as a rule adopts a different phrasal function, designated primarily by melodic shape. This is because *the dynamic contour stops assisting the pitch contour in integrating motifs and phrases into a single stereotypical wave-shape*, as it did in long-distance kula. Instead, maximum-distance kula sacrifices dynamic shaping on a phrasal level in sake of reproduction of the same dynamic contour on a motivic level: the last long tone in a motif is almost always the loudest. This leaves thematic elaboration as the main means of diversifying a complex kula. Motifs pass through greater transformations than shorter-distance kula: a long tone can ascend or descend by a step or a leap, and it can be preceded by a different number of short tones that can be sustained, embellished, or slid portamento. The intensity of such thematic elaborations is much greater: 14 seconds of maximal-distance kula contain 10 variations of “a” (Fig.7) whereas 13.5 seconds of long-distance kula—only 3 (Fig.6).

Each motif in the maximum-distance kula is shaped into a characteristic melodic shape by its position within a phrase: e.g., “a” initiates it, “b” contrasts the initiation, “a_1_” marks a phrasal climax point, “a_2_” provides a decaying variation, “a_3_” interrupts the phrase, “a_4_” insists by stepping higher to mark the cadence of the antecedent sentence, “a_5_” opens the consequent sentence with the initiation that is slightly longer than “a,” “a_6_” diversifies “a_5_”, “a_7_” generates the secondary climax point, “a_8_” interrupts the phrase, “a_9_” varies “a_8_” similar to the way in which “a_6_” varied “a_5_,” and finally, “a_10_” terminates the consequent sentence with a female cadence that contrasts a male cadence of the antecedent sentence. As a result, motifs of complex kula turn out to be more diverse and functionally specific than motifs of shorter-distance kulas.

Structural complexity brings modal complexity: anchor tones form intervallic relations that define degrees within a mode based on the *opposition of tetrachords or pentachords*, when the *antecedent sentence is confined to a higher tetrachord/pentachord* of the same or very similar structure as the *lower tetrachord/pentachord that forms the base for the consequent sentence*. The contrast of “imperfect” and “perfect” cadences tonally “rhymes” both sentences into a single “verse” analogous to the musical form of a “period” that makes an elementary unit for construction of music within the Western classical tradition (Ratner, 2001).

The similarities in intervallic structure between those degrees that occupy the same position within a sentence (e.g., the initiation of the antecedent sentence versus the initiation of the consequent sentence) secure the breaking of the entire musical mode into 2 equal parts (usually, tetrachords) of functionally identical or very similar degrees and the hierarchic relationship between both tetrachords. Constructing a musical mode by duplicating a tetrachord above or below the opening tetrachord is exceedingly common across different musical cultures of the world (Beliayev, 1990b). And chaining of two tetrachords into a single octave-equivalent (disjunct connection) or non-octave-equivalent (conjunct connection) heptatonic mode constitutes the most common form of modal genesis (Beliaev, 1963).^[[1]](#footnote-1)^ Curt Sachs (1960) called it the “chain principle.” A singer usually bases a phrase on the kernel of 3–4 pitch levels, forming degrees in a tetrachord (or, a trichord) and thereafter expanding that kernel by building the same structure above/below the kernel’s margin, whenever the melody calls for extra excitement or relaxation. Such “chaining” of tetrachords (trichords) usually disregards octave equivalence, since the singer tends to focus his/her attention only on nearby pitches engaged within the same tetrachord, whereas octave equivalence requires matching of the lowest degree of one tetrachord to the highest degree of the neighboring tetrachord.

Fig.7 features 6 degrees, broken into 2 tetrachords and connected in a *double-conjunct* manner. The earliest form of joining tetrachords was most certainly single conjunct (Sachs, 1962, 159): when a tetrachord of exactly the same structure was built on the marginal degree of the original tetrachord (e.g., C-D-E-F + F-G-A-Bb), producing a non-octave heptatonic mode (C-D-E-F-G-A-Bb). It turns out to lack octave equivalence, because the upper Bb often coexists with the lower B natural, added below the anchor “C” of the lower tetrachord in the same way as “E” of the lower tetrachord leads to the “F” anchor of the upper tetrachord. This, in effect, turns the tetrachordal base of this non-octave mode into the pentachordal base, preparing the ground for conceptualization of the disjunct connection: B-C-D-E-F + F-G-A-Bb.

Disjunct connection must have been discovered after the adoption of conjunct connection, after tetrachord earned its recognition as the basic unit of TO, and the lowest degree of a tetrachord acquired the default status of a permanent stable function. Disjunct connection, as a rule, eventually produces octave equivalence of the tonic: e.g., C-D-E-F + G-A-B-C makes I=VIII, generating the “C-chroma” and opposing tonic “C” to dominant “G” (Beliayev, 1990a). Disjunct construction enables the chaining of tetrachords of different structures, unleashing modal creativity within a musical culture. However, this development requires numerical-based music theory, usually formulated within the palace or temple culture of some urban civilization (Beliaev, 1965).

The same principles of mode-making apply to trichord-based music, such as different forms of pentatonic music systems: thus, C-D-F + F-G-Bb produces “archaic” non-octave pentatonic modes, whereas C-D-F + G-A-C generates more modern octave-equivalent modes (Beliayev, 1990a, 301). Double conjunct connection occurs when not one, but two degrees of both tetrachords overlap. This is what we observe in Fig.7: the consequent sentence features I (686–696 Hz, or 691 the 25 cents range circa F5), II (748–756 Hz, or mean 752 the 18 cents range circa Gb5), III (837–877 Hz, 857 or the 81 cents range circa Ab5) and IV degrees (933–947 Hz, or 940 the 26 cents range circa Bb5); whereas the antecedent sentence shares III and IV degrees with the consequent sentence, while adding two more degrees—V (1034 Hz, or C6) and VI (1153 Hz, or D6).

Double conjunct connection does not adhere strictly to the “chain principle” in keeping both tetrachords exactly the same in structure, because the functional relation between the conjunct pair of degrees inevitably changes within each of the tetrachords. The analysis of Fig.7 discloses this fundamental limitation. The degrees in the analyzed clip are separated by the following mean intervallic distances: 146 c between I–II, 226 c II–III, 160 c III–IV (double-conjunction), 165 c IV–V, and 189 c V–VI degrees. The rule of the smallest interval (rule No. 26 in Appendix-1 “Method of modal multi-factorial analysis) makes the II degree “lead” to the I degree, the III degree—to the IV degree, and the V degree—to the same IV degree. Then, the functional relation III–>IV in the upper tetrachord (its base pair) simply cannot be equivalent to the pairing of I–>II of the lower tetrachord (its base pair), since it is the II degree that “leads” to the I, and not the other way around. The asymmetry of both tetrachords is also evident in relation to their intervallic size: the lowest tetrachord (686–933 Hz) is 558 c wide, which is a step wider than the upper tetrachord (877–1153 Hz, i.e., 474 c wide). Because the middle degrees of the lower tetrachord (II–III) feature the largest interval in a mode, they must be neutral to each other, specializing in supporting the melodic motion toward the marginal anchoring degrees of that tetrachord.

The middle degrees of the upper tetrachord (IV–V) are 15% closer than its upper degrees (V–VI), which indicates that its middle degrees are connected by a descending “leading intonation” V–>IV. Most likely, double-conjunction is generated not by the reproduction of an entire tetrachord on top of or below another tetrachord, but by the accurate reproduction of intervallic and functional characteristics of the *upper pair of degrees* of the lower tetrachord by the lower pair of degrees of the upper tetrachord. This makes both tetrachords decidedly *asymmetric*: the lower tetrachord is defined by the characteristic modal intonations II–>I and III–>IV, whereas the upper tetrachord—by III–>IV and V–>IV. Clearly, the III–>IV intonation is mutual for both tetrachords, while V–>IV is asymmetric.

*Complex hierarchic relations between the degrees of both tetrachords are responsible for integrating different tetrachords into a single mode*. Therefore, it is plausible that the double-conjunction followed the simple conjunction but preceded the disjunction of tetrachords. Modal genesis by means of summing tetrachords, in all likelihood, involved a kind of “chunking” of a group of degrees. Hierarchic TO implies multi-level chunking:

1. in a melody, salient tones of the same distinct pitch level are grouped into a degree of a musical mode;
2. salient consecutive connected tones that represent different degrees form modal intonations;
3. degrees that house multiple modal intonations become grouped into trichords or tetrachords;
4. trichords and tetrachords become the building units in generation of new musical modes;
5. different strategies in connecting trichords and tetrachords lead to their expansion into respectively tetrachords and pentachords, grouped by pairing them into musical modes of different intervallic structures.

The progressive order of chunking most likely reflects the path in evolution of TO. And the connection of modal subsets (trichords, tetrachords, pentachords) secures the structural and functional uniformity of those modal degrees that are shared between different subsets: e.g., the upper degree of the lower tetrachord becomes identical to the lower degree of the upper tetrachord. This is the most important transformation—it marks the transition from *simple* tritonic and tetratonic modes (in Russian taxonomy they are considered “oligotonal”)^[[2]](#footnote-2)^ to *complex* pentatonic (conjunct trichord + trichord), hexatonic (disjunct trichord + trichord and conjunct tetrachord + tetrachord), and heptatonic modes (disjunct tetrachord + tetrachord and conjunct pentachord + tetrachord). The marginal degrees of each of the subsets here lose much of their modal autonomy and unique structural and functional features in sake of obtaining the uniformed membership in a greater and more complex musical mode.

In the end, the usage of characteristic modal intonations within each of the tetrachords causes the “conjunction pair” of degrees to generate functions that are mutual for both tetrachords and thereby establish the characteristic modal intonation(s) for the entire mode. If shorter-distance kula promotes a modal genesis within a tetrachord that houses a simple phrase/sentence, maximum-distance kula necessarily initiates the integration of tetrachords into a single mode, because it has to maximize the loudness of each phrase in order to make it audible over a kilometer or so. This deprives a kulerska of the most important means for pretonal organization—binding tones together with the help of the dynamic contour—which leaves *the stability* (i.e., minimal variability) *of a certain pitch level within the stereotypical wave-like melodic contour* as the principal means of modal genesis.

Stability of a particular tuning value turns it into an anchor, which in turn generates a modal degree and turns the intonations characteristic for that degree into characteristic modal intonations for the entire mode. This determines the integrity of that mode. So, a motif that falls on a specific part of the stereotypical melodic contour can effectively secure a particular expression of the entire mode. Thus, the IV degree of the antecedent sentence “A” supports both, the initiation and the cadence in that sentence, which makes it the most stable amongst all degrees of the corresponding tetrachord (III-IV-V-VI). The III degree provides the “leading tone” to the IV degree (since it features the smallest interval in that tetrachord: 118 c versus 168 c between the IV and V degrees). Then, III->IV is the characteristic modal intonation of this tetrachord. The V degree supports the passing melodic motion to the climactic VI degree. The consequent sentence does not use this tetrachord and instead engages the lower tetrachord (I-II-III-IV). In this tetrachord the IV degree is no longer the most stable anchor that it used to be in the higher tetrachord. Instead, it turns into the climactic degree—the most unstable anchor that requires diminuendo and resolution. The most stable degree now is the I, and its “leading tone” is supplied by the II degree (143 and 150 c—the shortest distance amongst the degrees of this tetrachord/sentence). The III degree becomes altered (flattened), so that instead of supporting the ascending intonation III–>IV (as it did in the antecedent sentence) it supports the descending intonation III–>II.

Hence, the reuse of the same pitch levels (III-IV) between two different sentences and two corresponding tetrachords causes a “modulation” from the IV degree as the temporary “tonic” for the antecedent sentence to the temporary “dominant” for the consequent sentence. The alteration of the III degree supports this modulation: this degree stays sharp in the higher tetrachord yet flattens in the lower tetrachord—changing in accordance to the melodic direction. Hence, the entire mode obtains the following modal rule: an ascending motion sharpens the III degree to bring it closer to the IV degree, whenever the latter becomes stable (temporary “tonic”), whereas a descending motion flattens the III degree, whenever it proceeds to the II degree on the way to the I degree (permanent tonic). *The double-functionality of the IV and III degrees defines this kulning mode*.

- The key role in modal genesis belongs to the reuse of the same pitch level between the sentences that are connected by their antecedent and consequent functionality within the stereotypical melodic contour.

Any kula is distinguished by the shape of a wave that receives its most prominent climax on the antecedent sentence, after which the wave flattens, marking yet a secondary climax, more modest than the first one (Wallin, 1991). *It is the stability of this shape that secures the stability of modal functions for any degree, whether they have to do with coordination or subordination of the constituent motifs/degrees*. That is why kula ends up generating the octave-equivalent frame of a pentachord which is divided in upper major 3^rd^ and lower minor 3^rd^ (Johnson, 1979). Pentachord provides a more prominent climax for a wave shape, as compared to a tetrachord, and promotes triadic tonicity, where all the odd degrees become stable as opposed to all the even degrees that become unstable, enabling the auxiliary and passing melodic motion to stable degrees (Mazel, 1952). By the same token, the emergence of pentachord turns a chained upper tetrachord into the modal inversion of the lower pentachord that maintains the same stable tones: the pentachord base of I–V generates the tetrachord base of V–VIII (I), whose enclosed degrees (VI and VII) are always unstable—just as in the enclosed even degrees of the pentachord (Beliayev, 1990a).

Kula seems to harness melodic motion between the “dominant” and “tonic” anchors with its antecedent-consequent segmentation of the stereotypical melodic contour. And it is the interaction of dominant and tonic that establishes the “tonic” pentachord I–V and the “tonic” tetrachord V-VIII(I) on the V degree, which usually alternates with the “dominant” pentachord V-IX(II)—cemented by the genesis of the tonic triad I–III–V–VIII(I) that turns into the tonic axis for the entire mode. This “triadic induction,” universal for many musical cultures, contracts the distances between the enclosed degrees in both, dominant pentachord and tonic tetrachord, eventually getting rid of the “gapped” modes (e.g., oligotonal or pentatonic) in favor of heptatonic octave-equivalent TO (Mazel, 1952). For kula, the process of modal contraction must have been triggered by the changes in distance between the herder and the herd during kulning. The contraction of ambitus is most obvious across 4 principal styles of kulning: 3 octaves for close distance (Fig.5), 2.2 octaves for mid-distance (Fig.4), 1.5 octave for long distance (Fig.6) and 1 octave for maximal-distance kulning (Fig.7). This contraction is directly related to the practice of sustaining pitch levels and turning them into modal degrees within the musical sentence/phrase structures.

Kula motifs most probably generated musical modes in a way analogous to how the continuous reuse of the same incipital, climactic and cadential intonations across different lyrics shaped the modal system of Gregorian chant (Helmer, 1975). In musicology, such spontaneous genesis of compositional conventions for TO, based on the reuse of pre-existing melodic figures and formulas has received recognition under the term of “**centonization**,” borrowed from the versification theory and adapted for understanding of the compositional practices of plainchant (Ferretti, 1934). Based on centonization, Leo Treitler has reconstructed a comprehensive theory of composition that seems to have been widespread across the world’s temple, palace, and village cultures, constituting a standard for orally transmitted forms of music (Treitler, 2007). The root “cento” (from Lat. “patchwork”) reflects the idea of *composing the entire musical or literary work by stitching together an array of pre-existing units according to the existing conventions, which over time forges syntactic rules of expressing certain semantic content* (Chew and McKinnon, 2001). Most likely, kulning constitutes one of the domains for cultivation of the centonate chant. The existing musicological studies of kulning leave no doubt that kulning puts into use a limited assortment of phonemes, syllables, melodic formulas, ornaments and rules of their configuration (Ahlbäck, 2007; Ivarsdotter, 1986; Johnson, 1979; Rosenberg, 2003, 2014; Wallin, 1991).

##### The interaction of all 4 kulning styles

Four styles of kulning all engage the same repertories of motifs, styles, onomatopoeic imitations, melodic and dynamic contours, only reconfiguring elements from each of these repertories differently, depending on the distance of communication. As evident by the cross-comparison of Fig.4-7, the increase in distance causes 9 important transformations in TO.

1. A *general increase in amplitude* (respectively, -40, -31, -27 and -20 dBu) causes the *great reduction in intra-phrasal dynamic shaping* in the *maximal* distance kula (it features the smallest variability of the dynamics for all forms of kulning, at 7 dB S.D., in contrast to 12 dB of the shorter-distance kula that features the most explicit dynamic shaping), making *intra-phrasal melodic* shaping the primary means of tonal integration at the longest distances.
2. *Intra-phrasal melodic contours* become more prominent as the distance of kulning increases, restricted to a single stereotypical wave-like shape in maximal-distance kula (if close-distance motherese and recitative are the freest in distribution of various melodic contours, long-distance kula imposes one wave-shape on a *single* kula sentence, as in the phrase “A” in Fig.6, whereas maximal-distance kula imposes one wave shape on the pitch values of *multiple* kula sentences, as in the phrases “A–A_1_” in Fig.7).
3. TO shifts from regulating primarily phonemic and timbral (e.g., registral and subharmonic) aspects, common for *speech*, in *closer*-distance kulning, to characteristically *musical* aspects of melody, thematic material and rhythm at *longest* distances. The number of registers reduces from four in Fig.4 to just one in Fig.7. The number of tones with clear subharmonic structure, in contrary, increases by distance, so that for Fig.5, 19 of 32 tones are non-periodic, whereas for Fig.7 they are all gone.
4. The *longer* the distance, the least important are the visual cues (kulerska’s gestures and mimics) and *the more abstracted are purely musical means of expression* (a melodic shape, a style of articulation, a rhythmic figure, a melodic interval) that require memorization and auditory recognition on the part of both, music-maker and listener (thus, Fig.5 requires memorization of just *one* pitch level, whereas Fig.7—*six*). Therefore, the formative power of conventions to forge idiomatic patterns for each of the aspects of musical expression is the greatest at maximal distances.
5. The *longer* the distance, the *better pitched are the tones of kulning*: for Fig.5, poorly pitched tones (i.e., non-periodic, noisy, harsh, onomatopoeic, and glissando sounds) constituted 57% of the clip and were mostly short (on average, 189 ms long, with S.D. of 68 ms); for Fig.4, the corresponding figures were 60% of even shorter duration (mean 124 ms, but of greater S.D. of 130 ms); for Fig.6, these figures were 56% of much longer duration (259 ms mean and 294 ms S.D.); and for Fig.7, the share of poorly pitched tones drastically dropped to 28% (shortening down to mean 125 ms and 35 ms S.D.). Evidently, *maximal* distance promotes *longer tones* of *well-sustained pitch levels*, limiting a poorly tuned pitch to *short embellishments* and *portamento* glides, as opposed to *closer* distances, at which many poorly pitched tones originate from *longer spans of onomatopoeia, recitative, and/or pitch-bending*.
6. *Longest*-distance kulas *maximize the span of the musical motifs and phrases*, thereby *increasing the importance of pitch-related AEs*: for Fig.5, 32 pitch changes occupy 67% of the clip (2.1% per change) with the mean tone duration of 267 ms and the motif duration of 967 ms; for Fig.4, 30 changes—35% (1.2% per change) of the mean 148 ms tone and 896 ms motif durations; for Fig.6, 26 changes—67% (2.6% per change) of the mean 366 ms tone and 1268 ms motif durations; for Fig.7, 43 changes—68% (1.6% per change) of the mean 273 ms tone and 1001 ms motif durations. Evidently, compared to close distance, mid-distance kulning reduces the density of pitch changes by shrinking the average tone duration while increasing the motif span. Long distance maximizes the density of pitch changes, the average tone, and motif duration. And the maximal-distance kula maximizes the number of pitch changes and sentences (about twice longer than in long-distance kulnning), while reducing the tone/motif span due to significant increase in the share of embellishments. At the same time, the *rhythmo-metric organization grows looser and looser as the kulning distance increases* (the rhythmic diversity of maximal-distance kula is 136% greater than in long-distance kula).
7. The *longer* the distance, the *greater the share in expression given to pitch changes* over the other AEs. Maximally long kula features the greatest versatility of rhythm and fundamental ametricity in conjunction with the longest melodic span. The free unveiling of the maximal-distance kula as though negates any structural restrictions and contrasts. This is the only style of kulning that does not use multiple registers and contrasts in timbre, pitch, rhythm and dynamics. Such maximal homogeneity corresponds to the function of “safe call,” designed to reassure the grazing animals in their safety. Subsequently, the maximal-distance kula features the most melodically dense legato style— with merely 17% of the clip given to silence and all pitch changes squeezed within the narrowest ambitus of 1 octave, where stepwise motion is completely overwhelming.
8. The *longer* the distance, the *higher in register* the melodic motion is pushed up: close kulning is mostly active melodically in the range of 115-378 Hz, mid-distance kulning—300–900 Hz, long-distance kula—500–900 Hz, and maximal distance kula—625–1281 Hz. The higher the registration, *the greater number of higher degrees become prominent in modal genesis*. Close distance features 1 degree (low stable anchor with the mean value of 273 Hz and the variability within 85-202 c). Middle distance also features 1 degree (high unstable anchor at 1072 Hz and the variability of 15-37 c). Long distance features 4 degrees (low stable I anchor at 596 Hz, high unstable IV anchor at 874 Hz, high auxiliary unstable degree at 722 Hz and low auxiliary unstable degree at 671 Hz). Maximal distance features 6 degrees (3 anchors: low stable “tonic” I at 686 Hz, middle medium-unstable “dominant” IV at 933–947 Hz, and high maximally-unstable VI at 1153 Hz; and 3 auxiliary unstable degrees: V at 1034 Hz, III at 837–877 Hz, and II at 748–756 Hz—with the variabilities of 26 c for IV, 81 c for III, and 18 c for II degrees). With the exception of III, which constitutes a modal alteration, the variability of pitch values per degree decreases by increase in distance, as the number of degrees increases.
9. The *longer* the distance, the more important the *aesthetic* evaluation of kulning becomes comparing to the *utilitarian* importance of its signals (thus, maximum distance kula features 21 shake-like embellishments, whereas long-distance kula and motherese—only 4). Stereotypicity of phrases must be responsible for the increased ornamentation of the maximal-distance kula, called to diversify the ongoing repetitions of the same melodic/dynamic formula. Taking many small caesuras allows the singer to substantially diversify the melodic shape of the constituent motifs, thereby emphasizing the artistic aspect of kulning and enabling aesthetic competition between different kulerskas, judged and encouraged by human witnesses of their singing.

All in all, cross-examination of 4 styles of kulning, categorized by distance, and of 5 types of motifs, categorized by their semantic and structural typologies, strongly indicates that the increase in distance during the same session of kulning generates incremental differentiation of pitch, defining modal degrees of different melodic functionality and integrating them into a single complex hierarchic system of TO (Table-1).

**Table-1**. **Acoustic traits of main motif types and their semantic values** **in kulning.** Nine AEs (in rows) are used in 5 types of phrases (in columns), each of which also consists of subtypes. Kula has 4 subtypes, whereas the other 4 types feature just a couple (onomatopoeic imitations usually imitate either dog-barking or the typical vocalization of an animal to which the imitation is directed). Each type/subtype is characterized by a unique combination of AE patterns, the most distinctive of which are pitch, rhythm, articulation, dynamics, and register. Each type also is distinguished by its semantic specialization and distance: kula—tropotrophic (long distance), exclamation calls—phatic (long and medium distance), onomatopoeia—ludic (medium distance), parlando—imperative (medium and close distance), motherese—endearing and trusting (close distance).

| **Acoustic domains** | **AEs**  **of music** | **Kula singing phrases & sentences** | **Exclamation calls** | **Onomatopoeic imitations** | **Parlando singing & calling** | **Motherese recitative** |
| --- | --- | --- | --- | --- | --- | --- |
| Frequency | 1.Melodic pitch (consecutive) | Prevalence of long descending stepwise motion, 4 subtypes: 1) overall descending contour, 2) climactic stepping up (in longer kulas), 3) optional wide opening gliding leap up, 4) optional termination by an ascending mid-size leap. Narrow ambitus of a 5^th^ breaks in two portions: upper maj.3^rd^ & lower min.3^rd^ (with possible infrafix). Well-defined pitches, many melismas. The strongest melodic coherence. “Safety call” function –calming yet keeping alert, under control. Subject to aesthetic evaluation. | Prevalence of a short zigzag shape with a very short start, 2 subtypes: 1) stressed ascending leap (<4^th^) followed by descending leap (<tritone), 2) stressed ascending step & a long gradual fall (sliding FM as in “stop whistle”). Poorly defined pitches. Subtype 1) has huge ambitus (1.6 octave), can use shakes as melismas and is used for stimulation. Subtype 2) is much narrower (0.5 octave), without any melismas, used for inhibition. Exclusively phatic function (no aesthetic value). | Prevalence of short repetitions of the same sound (usually of the herded animals or dog’s barking). Non-pitched or poorly-pitched (with possible FM) sounds of very broad bandwidth. No melismas at all. Melodic coherence is completely absent. The primary function is to stimulate animals by triggering energetic response – usually to make the herd move in the necessary direction. Most likely, this is a derivative of a “fetch whistle” command. Utilitarian application (no aesthetic value). | Prevalence of a zigzag shape, similar to exclamation, but often longer & stressing a huge descending leap (up to 1.6 octave), greater than an ascending leap (<7^th^). Both leaps exceed those of exclamation – but relax towards the end. Stimulating function. An alternative subtype is less intense, like motherese subtype-1, but is sung-out & smoothened. Inhibitive function. Most pitches are quite clear, without melismas. Utilitarian application with aesthetic value. | Prevalence of drastic contrasts in pitch, 2 subtypes: 1) very flat, monotonous (<2^nd^) phrases of pitched talking, 2) marginal zigzag leaps (about 1.7 octave) of a single syllable in a word/phrase. Stimulating & motivating functions, with strong use of gestures, mimics & touch to support the acoustic expression in directing the herd. Only a few pitches are clear. The peak tones often receive a melismatic shake. Mostly utilitarian application. |
|  | 2.Harmony | n/a | n/a | n/a | n/a | n/a |
|  | 3.Form (complexity) | Greatest complexity | Simplicity | Greatest simplicity | Low complexity | Medium complexity |
| Time | 4.Tempo | Slow, frequent ritenuto towards the phrasal end. | Moderate subtype-1, slow subtype-2, possible ritenuto. | Moderate, possible accelerando. | Moderate or slow, frequent rubato. | Moderate and lively, with moderate rubato. |
|  | 5.Rhythm | Sharp contrasts of melismatic & anchor tones, the greatest rhythmic diversity, yet lack of increments. | Contrast of long peak & short initiation in subtype-1, totally arrhythmic subtype-2. | Prevalence of the same relatively short rhythm, grouped by pauses, clear increments. | Contrast of short upper & long lower tones in subtype-1, mostly short equal rhythm in subtype-2. | Contrast of long initial & short last tones in subtype-1, free “verbal” rhythm in subtype-2. |
|  | 6.Meter | Most irregular, often totally “ametric,” a dragging feel. | Can contain regular fragments, usually iambic. | Mostly regular (spondaic). | Usually irregular (loose, free). | Irregular, as if always changing iambic-trochaic. |
|  | 7.  Articulation | Absolute dominance of legato. Frequent & possibly very long caesuras between phrases. Phrases usually end on long tenuto tone with a descending glide. | Contrasted groups of staccato on ascending leaps & legato on descending steps. Subtype-1 often ends with a tenuto glide. | Prevalent non legato provides ease & clarity of recognition for each of the imitations. | Contrasted groups of staccato for ascending leaps & legato for steps & descending medium leaps. Subtype-1 can end on tenuto. | Syllables within a word usually receive legato treatment, while words or vocables are separated by pauses. |
| Amplitude | 8.Dynamics | The loudest type imposes a holistic diminuendo on the entire mid-distance kula & a wavelike shape on each of the phrases in the long-distance kula. | Rather intense dynamics: subtype-1 stresses the high tones & destresses the low tones, whereas subtype-2 engages a wavelike shape. | Only minor dynamics changes within mostly loud levels, copying the typical dynamics envelop of a typical animal call. | Moderate contrast of softer high tones & louder lower tones in subtype-1, overall soft dynamics in subtype-2. | The softest type, yet with sudden accents, falling on a single syllable in words marked by a zigzag leap. Frequent strong attacks. |
| Timbre | 9.Register | Single, very high register - the longer the distance, the brighter the tonal quality (piercing or shrilling). Fixed high larynx position, constant brightness. | Multiple registers: shrieking for the highest tones, shrilling (kula-like) for high, “casual” for low tones. Variable larynx position. | Single register for each onomatopoeic imitation, usually broadband – in contrast to the narrow-band kula. Usually high larynx. Overall dullness. | Contrast of 2 registers: head-voice (kula-like) for high tones & throat or chest singing voice for low tones. Variable larynx position. | Contrast of 2 registers: head-voice (kula-like) for one syllable & normal speaking voice for the rest. Variable larynx position. |
|  | 10.  Harmonicity | Clear harmonics (akin to pure tones), portamento attack & termination (in longer kulas), minimal vibrato (only to embellish a tone such as a trill). | Clear harmonics (akin to kula) for the highest & longest tones only, ascending & descending portamento, no vibrato. | Prevalence of non-periodic spectrum, harsh, noisy sound, little voicing (only if present in the imitated model), no portamento or vibrato. | Clear & rich harmonics for the lowest & longest tones only (some vibrato), ascending & descending portamento for leaps only. | Prevalence of non-periodic spectrum, as in speech, with much FM & noise. No vibrato. Leaps engage portamento. |

Except for the long-distance kula (whose sentences can reach up to 15 seconds), all other types are quite brief (usually, 0.3-2 sec) and are intermixed with the same or shorter-distance vocalizations (i.e., kula phrases can be included in motherese recitative, but motherese cannot be included in kula). The maximal distance squeezes the ambitus into an octave confined to a single highest register. This compresses degrees into steps of a smaller or a longer size, depending on their phrasal position. Climactic step tends to be “major,” whereas cadential step—“minor,” to facilitate resolution. Mid-distance kula transposes heptatonic structures to lower registers, fitting them into a tetrachord (pentachord, if a climactic motif is added). Octave equivalence secures heptatony. Closer distances enable alterations, flattening/sharpening the degrees, and timbral recoloring.

- Stacking phrases of *contrasting TO and semantic values*, learnable by *humans* *and* *domesticated animals*, generates the SFTO.

The *transition from long-distance kula to maximal-distance kula* is especially instrumental in promoting the *multi-tetrachordal (pentachordal) melopoeia* with the conjunct degrees that manifest different functionality in different tetrachords. This establishes the hierarchic relations between all the degrees within a musical mode, causing certain degrees to pair with others by forming the characteristic modal intonations and subdividing degrees into stable and unstable, of various gradations, and enabling alterations and modulations.

As a result of reuse of the same melodic formulas at different distances, TO of kulning becomes oriented primarily toward a pitch-related AEs rather than time-, timbre- and amplitude-related AEs. TO acquires a heptatonic structure with octave equivalence and dominant-tonic dichotomy in integrating antecedent and consequential sentences into a single musical period that engages the same stereotypical wave-like pitch contour over a few musical sentences/phrases of similar thematic material. The resultant musical mode tends to consist of a “tonic” pentachord base with the conjunct “tonic” tetrachord placed on top of it, where the odd degrees mark the tonic triad as modal anchors (I-III-V), offsetting the unstable even degrees (II-IV-VI) and reserving the VII degree as the “leading tone” for the ascending resolution VII-VIII (I). In this process, *the pitch classes bring to life interval classes*: pentachord marks a perfect 5^th^, tetrachord—a perfect 4^th^, the tonic triad—major and minor 3^rd^, neutral neighboring degrees—major 2^nd^, and “leading tone(s)”—minor 2^nd^. The interaction of the imperfect interval classes paves the way for the development of major and minor typologies of TO. In effect, practicing kulning on a day-to-day basis leads to crystallization of tonality with its major and minor keys, very close to how we use them today in making and consuming music.

BIBLIOGRAPHY

Adler, K. (1965). *The Art of Accompanying and Coaching*. Minneapolis, MN: University of Minnesota Press.

Ahlbäck, S. (2007). *The Tonality of Older Swedish Folk Music*. Stockholm, Sweden: Udda Toner.

Alekseyev, E. Y., and Nikolayeva, N. (1981). *Models of Yakut vocal folklore [Образцы якутского песенного фольклора]*. Yakutsk: Academy of Sciences of the USSR.

Barbour, J. M. (2004). *Tuning and Temperament: A Historical Survey*. New York: Dover Publications.

Beliaev, V. (1963). The Formation of folk modal systems. *Journal of the International Folk Music Council* 15, 4–9. doi:10.2307/836227.

Beliaev, V. (1965). Folk Music and the History of Music. *Akademiai Kiado* 7, 19–23. doi:10.2307/901408.

Beliayev, V. M. (1990a). “Modal systems in the traditional music of the USSR [Ладовые системы в музыке народов СССР],” in *Viktor Mikhailovich Beliayev [Виктор Михайлович Беляев]*, ed. I. Travkina (Moscow: Soviet Composer), 223–377.

Beliayev, V. M. (1990b). *Viktor Mikhailovich Beliayev [Виктор Михайлович Беляев]*. Moscow: Soviet Composer.

Bukofzer, M. F. (1940). Popular Polyphony in the Middle Ages. *The Musical Quarterly* XXVI, 31–49. doi:10.1093/mq/XXVI.1.31.

Chew, G., and McKinnon, J. W. (2001). Centonization. *The New Grove Dictionary of Music and Musicians*. doi:10.1093/gmo/9781561592630.article.05279.

Cooke, D. (1959). *The language of music*. London: Oxford University Press.

Ferretti, P. M. (1934). *Estetica gregoriana ossia Trattato delle forme musicali del canto gregoriano*. Rome: Pontificio Istituto di Musica Sacra.

Helmer, R. H. P. (1975). *European Pastoral Calls and Their Possible Influence on Western Liturgical Chant*. New York, NY: Columbia University Press.

Huron, D. (2001). Tone and Voice: A Derivation of the Rules of Voice-Leading from Perceptual Principles. *Music Perception* 19, 1–64. doi:10.1525/mp.2001.19.1.1.

Ivarsdotter, A. (1986). Sången i skogen. Studier kring den svenska fäbodmusiken.

Johnson, A. (1979). Fäbodmusik i Förvandling: Rapport Från En Exkursion Sommaren 1977. *Svensk Tidskrift för Musikforskning* 60, 5–39.

Juslin, P. N. (2005). “From mimesis to catharsis: expression, perception, and induction of emotion in music,” in *Musical communication*, eds. D. Miell, R. MacDonald, and D. J. Hargreaves (Oxford, UK: Oxford University Press), 85–116.

Kvitka, K. V. (1971). *Selected Works [Избранные труды]*, ed. V. L. Goshovsky Moscow: Soviet Composer.

Mazel, L. (1952). *On melody [О мелодии]*. Moscow: Gos Muz Izdat [State Musical Publishing].

McConnell, P. B. (2002). *The Other End of the Leash: Why We Do What We Do Around Dogs*. New York: Ballantine Books.

McConnell, P. B., and Baylis, J. R. (1985). Interspecific Communication in Cooperative Herding: Acoustic and Visual Signals from Human Shepherds and Herding Dogs. *Zeitschrift für Tierpsychologie* 67, 302–328. doi:10.1111/j.1439-0310.1985.tb01396.x.

Narmour, E. (1992). *The Analysis and Cognition of Melodic Complexity: The Implication-Realization Model*. Chicago, IL: Chicago Review Press.

Nikolsky, A. (2016). Evolution of Tonal Organization in Music Optimizes Neural Mechanisms in Symbolic Encoding of Perceptual Reality. Part-2: Ancient to Seventeenth Century. *Frontiers in Psychology*. doi:10.3389/fpsyg.2016.00211.

Nikolsky, A., Alekseyev, E. Y., Alekseev, I. Y., and Dyakonova, V. E. (2020). The overlooked tradition of ‘personal music’ and its place in the evolution of music. *Frontiers in Psychology* 10, 3051. doi:10.3389/fpsyg.2019.03051.

Ratner, L. G. (2001). Period. *The New Grove Dictionary of Music and Musicians*. doi:10.1093/gmo/9781561592630.article.21337.

Rice, T., Porter, J., and Goertzen, C. (2000). *The Garland Encyclopedia of World Music: Europe*. New York: Garland Publishing.

Rosenberg, S. (2003). *Kulning. Musiken och metoden*. Stockholm, Sweden: Udda Toner.

Rosenberg, S. (2014). Kulning – an ornamentation of the surrounding emptiness: about the unique Scandinavian herding calls. *Voice and Speech Review* 8, 100–105. doi:10.1080/23268263.2013.829712.

Sachs, C. (1960). Primitive and medieval music: a parallel. *Journal of the American Musicological Society* 13, 43–49. Available at: http://www.jstor.org/stable/830245 [Accessed December 10, 2014].

Sachs, C. (1962). *The wellsprings of music*, ed. J. Kunst The Hague, Netherlands: Martinus Nijhoff.

Shackford, C. (1962). Some Aspects of Perception III: Addenda. *Journal of Music Theory* 6, 295–303.

Treitler, L. (2007). “‘Centonate’ Chant: übles Flickwerk or e pluribus unus?,” in *With Voice and Pen*, ed. L. Treitler (Oxford: Oxford University Press), 186–201. doi:10.1093/acprof:oso/9780199214761.003.0007.

Turetzky, B. (1974). *The contemporary contrabass.* Berkeley, CA: University of California Press.

Wallin, N. L. (1991). *Biomusicology: Neurophysiological, Neuropsychological, and Evolutionary Perspectives on the Origins and Purposes of Music*. Hillsdale, NY: Pendragon Press.

1. For the discussion of special importance of the interval of a 4^th^ and the tetrachord as the principal unit of modal organization see the chapter “Genesis of modal family and the role of tetrachord“ (Nikolsky, 2016). [↑](#footnote-ref-1)
2. The term "oligotonal" (from Greek "oligo"—few) was introduced by Kliment Kvitka (1971, 1:285) in 1927 to refer to modes that feature fewer than 7 degrees, but are not organized by the pentatonic principle—so that the tones do not conform to the circle of 5^ths^. Kvitka was addressing the issue that anhemitonic mode can originate from multiple principles of organization, where diatonic principle is only one possibility. Many archaic folk modes contain 3-4 stepwise degrees, comprising the nucleus (2-3 degrees) and an infrafix or suprafix (1 degree), set apart by the interval of a 4^th^ or a 3^rd^ (e.g., C-E-F-G – the nucleus is underlined). Qualifying such modes as “incomplete pentatonic” would be inaccurate, since it would imply some kind of incompletion and therefore defectiveness, whereas in many cases songs created in such modes possess their own inherent logic of tonal unity (1971, 1:310). [↑](#footnote-ref-2)
